# Nephroprotective Plant Species Used in Brazilian Traditional Medicine for Renal Diseases: Ethnomedical, Pharmacological, and Chemical Insights

**DOI:** 10.3390/plants14050648

**Published:** 2025-02-20

**Authors:** Rodrigo Moraes Carlesso, Yasmin Louise Ramos Cappellari, Daiana Daniele Boeff, Alícia da Costa Pereira, Elisa Schmitt Rusch, Thiago de Souza Claudino, Mara Rejane Ritter, Eduardo Luis Konrath

**Affiliations:** 1 Laboratory of Pharmacognosy, Faculty of Pharmacy, Universidade Federal do Rio Grande do Sul (UFRGS), Porto Alegre 90010-150, Brazil; rodrigo288987@gmail.com (R.M.C.); louise.yasmin314@gmail.com (Y.L.R.C.); dd.boeff@gmail.com (D.D.B.); aliciacosta2610@gmail.com (A.d.C.P.); 00337869@ufrgs.br (E.S.R.); 2 Pos-Graduate Program in Pharmaceutical Sciences, Faculty of Pharmacy, Universidade Federal do Rio Grande do Sul (PPGCF-UFRGS), Porto Alegre 90010-150, Brazil; 3 Irati Campus, Federal Institute of Education, Science and Technology of Paraná (IFPR), Irati 84500-000, Brazil; thiago_claudino@yahoo.com.br; 4 Department of Botany, Biosciences Institute, Universidade Federal do Rio Grande do Sul (UFRGS), Porto Alegre 90010-150, Brazil; mara.ritter@ufrgs.br

**Keywords:** nephroprotection, kidney disorders, ethnomedicine, secondary metabolites, plant extracts

## Abstract

The prevalence of kidney-related diseases has been increasing and has emerged globally as a leading cause of mortality, especially in developing countries where they are considered a neglected public health problem. Renal diseases are commonly progressive and may cause irreversible loss of organ function, eventually necessitating renal replacement therapy. Although different pharmaceuticals are considered for the treatment of these pathologies, the uncertain effectiveness and presence of adverse effects have generated a growing need for the development of novel nephroprotective compounds. Because many medicinal herbs are typically used in Brazilian folk medicine to prevent and cure kidney ailments, ethnomedicine may play a promising and strategic role in identifying and adding new potential molecules to the pharmacological arsenal. This review focuses on the use of plants and secondary metabolites belonging to different classes to treat renal diseases, associating the screened plant extracts with the bioactive components present in each species. Flavonoids and triterpenes are notable metabolites that have therapeutic potential. The putative pharmacological mechanisms related to nephroprotective properties are also discussed in in vitro and in vivo models, when available.

## 1. Introduction

Renal diseases are common global causes of morbidity and mortality that cause an increasing socioeconomic burden on public health programs, particularly in low- and middle-income countries [1]. These conditions may impair both kidney structure and function and are associated with significant adverse clinical outcomes [2]. In addition to their high prevalence, kidney diseases are typically associated with comorbidities, including diabetes, hypertension, and coronary heart disease [3,4]. Currently, the management of renal diseases remains a challenge because of their multifold impact, with more than two million people globally receiving renal replacement therapy in the form of continuous dialysis methods or, eventually, kidney transplantation [5]. Based on the cause, duration, and severity, abnormalities in kidney function can be classified as acute kidney disease (AKD) or chronic kidney disease (CKD) [6,7].

AKD is a life-threatening decline in kidney function that affects the glomerular filtration rate and is more reversible than CKD [8]. Glomerular or tubular injury, trauma, and exposure to nephrotoxic compounds, including medicines and mycotoxins, are associated with this condition [9]. Importantly, persistent and unresolved episodes of acute renal injury are implicated in the progression to CKD and end-stage kidney disease (ESKD), particularly in the elderly [10]. Serum creatine, proteinuria, and microalbuminuria are the current clinical markers for the detection of kidney disease progression; however, these markers are neither specific nor sensitive [8]. Repeated renal injuries may result in glomerulosclerosis, vascular rarefaction, and fibrosis, all of which are associated with CKD and ESRD [11].

**Figure 1:**
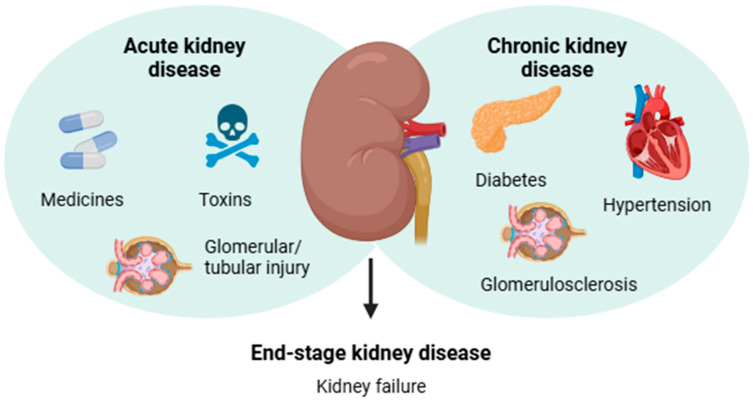
Infographic summarizing the main causes related to the etiology of acute and chronic kidney diseases, which may lead to end-stage kidney disease.

In contrast, CKD gradually develops as a systemic kidney disorder associated with renal pathological features or secondary complications of chronic diseases including diabetes and hypertension [3,4]. The diagnosis of CKD is mainly based on the presence of albuminuria for a period or a glomerular filtration rate < 60 mL/min per 1.73 m^2^ for ≥3 months, which indicates decreased kidney function [12]. According to the World Health Organization (WHO) estimates, approximately 850 million people worldwide are affected by some form of kidney disease, and CKD is projected to become the 5th most common chronic disease by 2040 [6]. Notably, deterioration of renal function is a silent process due to the lack of physical signs, and most affected individuals are asymptomatic until CKD advances to kidney failure [4,13]. The main causes related to the development of AKD and CKD are shown in Figure 1.

Patients with type 2 diabetes and hypertension are more closely affected and experience a substantial reduction in the quality of life with progressive loss of renal function, resulting in kidney failure and high mortality rates. Some non-pharmacological approaches, including a range of lifestyle and dietary strategies (weight loss, protein restriction, blood pressure, and glucose control), can adequately preserve kidney function [14,15]. Traditionally available pharmacological interventions are rarely curative and are classified into three main targets: (i) agents that slow the progression of the disease (angiotensin-converting enzyme inhibitors, angiotensin receptor blockers, and sodium-glucose transport protein inhibitors, known as SGLT-2 inhibitors or gliflozins); (ii) agents that reduce cardiovascular risk (lipid, glucose, and blood pressure-lowering drugs); and (iii) agents that manage uremia and associated symptoms (veverimer, loop or thiazide diuretics, and uric acid-lowering drugs) [16,17]. Further clinical evidence shows that newer nonsteroidal mineralocorticoid receptor antagonists play an important role in preventing and managing AKD-CKD transition, acting as antifibrotic and anti-inflammatory agents [18].

In addition to the limited number of nephroprotective therapies available in current clinics, studies have associated an increased risk of adverse outcomes with the use of some agents, including hyperkalemia or uncertain effectiveness of multi-agent renin–angiotensin–aldosterone system blockade and euglycemic ketoacidosis for SGLT-2 inhibitors [16]. Some drug combinations have not shown additional benefits and may induce serious adverse effects including acute kidney injury and renal dysfunction [19]. Moreover, current pharmacological approaches have been shown to mitigate the risk of CKD progression, particularly in patients with diabetes type 2, but are unlikely to slow disease progression, prevent the development of ESRD and cardiovascular disease, or ensure a better health-related quality of life for these patients [20].

Therefore, we considered the research of plant species that may have nephroprotective activity because many of these plants are widely used as folk medicines in Brazil. In this review, we discuss how plants and their active compounds are used to treat conditions (in vitro and in vivo effects) that are consistent with kidney-related diseases. Furthermore, we review and summarize the empirical use of medicinal plants for the treatment of renal symptoms and provide scientific evidence obtained from preclinical studies.

## 2. Plant Species Used in Brazilian Ethnomedicine with Nephroprotective and Kidney-Related Properties

The primary source of human healthcare has been linked to the millennial use of medicinal plants. The World Health Organization has estimated that four billion people worldwide still rely on traditional remedies, representing 80% of the world’s population [21,22]. Ethnomedicine plays a pivotal role as a validated strategy to identify new natural chemical entities with important pharmacological activities [23,24]. Plant-derived metabolites are often regarded as chemically complex molecules with outstanding and versatile scaffold diversity compared with typical synthetic compounds [25,26]. Screening and identification of novel therapeutic leads are routinely performed using analytical and computational techniques, including high-throughput screening, medicinal chemistry, molecular docking, and omics [27]. The importance of plants in discovering new drug entities is still growing, as approximately half of the new therapeutic agents approved between 1981 and 2019 are recognized as natural products or their synthetic variants based on natural pharmacophoric groups [28].

Brazil contains approximately 45,000 plant species, corresponding to 20–22% of the global flora, many of which are used for medicinal purposes [29]. Various bioactive molecules have been isolated from native Brazilian plants; however, few studies have investigated their mechanisms of action [30]. Global estimates show that only a small percentage of higher plants on the planet have been chemically (15%) or pharmacologically (6%) investigated [31]. In Brazil, a significant number of plant species have historically been used to normalize kidney function, including medicinal allegations as nephroprotective and diuretic agents, stone elimination, and blood depuration. The properties of plant species used in traditional medicine for these purposes compiled from books about medicinal plants and folk medicine published in Brazil (Table 1) are listed in Table 2.

## 3. Methodology

A comprehensive review of the medicinal plants used in Brazilian traditional medicine as remedies against kidney-related disorders and their associated bioactive compounds was conducted. To perform bibliographic research, ethnobotanical books containing primary surveys and compilations conducted by authors linked to the national scientific academy were searched (Table 1). The literature used in the review covered plants native or exotic to the Brazilian flora present in all biomes in the country using specific keywords in Portuguese such as “diuretic”, “kidney stones”, “blood purifier”, and “kidney disease”, among others. The following information was collected: plant species, vernacular names, parts of the plant used, and related folk medicinal uses (Table 2). All valid scientific names of species, authors, botanical families, and origins were checked using The World Flora Online “http://www.worldfloraonline.org (accessed on 12 October 2024)” and Flora e Funga do Brasil “http://floradobrasil.jbrj.gov.br (accessed on 15 October 2024)”.

The review process was continued by consulting peer-reviewed journal articles available in electronic databases such as Scopus, PubMed, SciFinder, and SciELO and using keywords associated with the scientific names of plants with in vitro, ex vivo, and in vivo nephroprotective activities. Next, the secondary metabolites present in related plant species used in folk medicine were reviewed using the databases mentioned above, and a comprehensive review was further refined by combining keywords related to the in vitro, ex vivo, and in vivo nephroprotective activities of each compound, resulting in a list of bioactive metabolites useful for treating kidney-related disorders (Tables S1 and S2 of the Supporting Information). For all searches, the inclusion criteria were based on the credibility of the sources from which data were collected. No specific restrictions were considered, and all preclinical studies were included and investigated.

The inclusion criteria for the ethnobotanical compilation were as follows: (i) plants species traditionally used in traditional Brazilian medicine specifically for the treatment of kidney-related diseases, (ii) books edited in Brazil based on ethnobotanical studies or compilations of folk medicinal reports published by scientists, and (iii) reports containing the specific botanical epithet. The inclusion criteria for pharmacological evidence were as follows: (i) Brazilian species with traditional reports of nephroprotection, (ii) well-designed studies of pharmacological evaluation (in vitro, in vivo, or in silico), and (iii) bioactive components present in the organs of the species traditionally used for renal diseases. The exclusion criteria applied for medicinal plants were if they were (i) identified only to the genus level, (ii) cited only by their popular names, and (iii) exotic, non-acclimatized plants were excluded, resulting in a complete database final list.

## 4. Nephroprotective Strategies in Acute Kidney Disease and Chronic Kidney Disease

One potential reason for plant-derived phytochemicals being suggested as promising strategies to prevent or treat kidney-related diseases is their complex pleiotropic effects based on potential interactions with different pharmacological sites. Considering that kidney-related diseases involve multiple pathological pathways, the presence of different nephroprotective natural compounds in plant extracts represents a multitarget approach, as they can act synergistically, thus modulating important proteins involved in renal pathogenesis [52].

**Figure 2:**
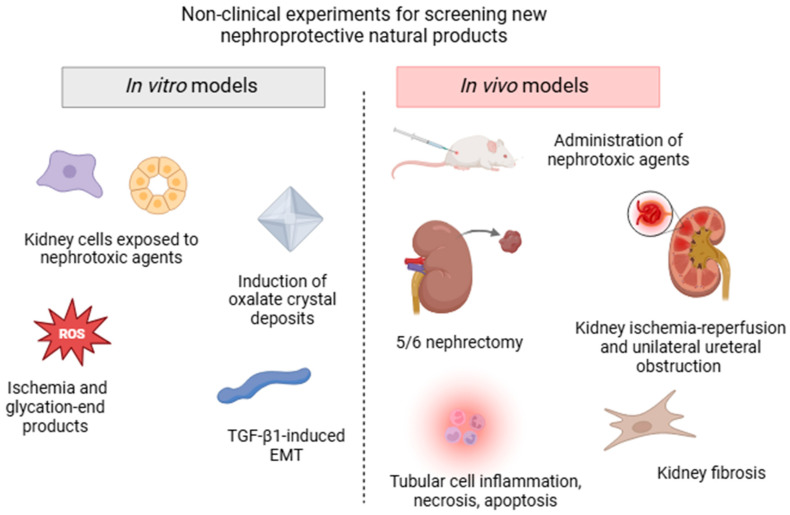
Schematic illustration of the main non-clinical models (in vitro and in vivo assays) commonly used for screening nephroprotective plant extracts and/or isolated compounds.

Diverse experimental non-clinical approaches are currently used to screen the bioactive components of plants traditionally used for their pharmacological activity in kidney disorders, all of which mimic the different processes involved in AKD and CKD (summarized in Figure 2). In vitro models can be quickly performed in batteries and often use human, rat, porcine, or canine renal cell lines, such as glomerular mesangial cells, tubular epithelial cells, interstitial fibroblast cells, podocytes, or renal structures exposed to drug-induced nephrotoxicity. Strategies to target the pharmacological mechanisms in these cells also include incubation with high glucose and glycation end products, chemical ischemia, transforming growth factor β1 (TGF-β1)-induced epithelial to mesenchymal transition, and induction of kidney oxalate crystal deposits [53,54,55,56].

However, in vivo experimental models that expose rats or mice to different etiological factors and conditions are more extensively used to assay molecules or extracts that can potentially block or reverse the progression of kidney diseases. Classically, AKI is induced by the administration of various noxious stimuli including antibiotics, chemotherapeutic agents, aristolochic acid, heavy metals, pesticides, and endogenous toxins (sepsis-induced AKD) [52]. Surgical strategies, such as kidney ischemia-reperfusion and unilateral ureteral obstruction (UUO) models, are low-cost and often used to affect specific physiological components, triggering tubular cell necrosis, apoptosis, inflammation, and oxidative stress damage. Some CKD models include diabetic nephropathy, hypertension-induced renal damage, glomerular injury, and renal mass reduction (5/6 nephrectomy) [57]. Although these models do not fully reproduce human clinical features, they share some common specific cell-signaling pathways and are usually accompanied by a decline in renal function, represented by elevated serum creatinine and urea levels, decreased glomerular filtration rate, tubular lesions, proteinuria, and fibrosis [52].

Considering that CKD may ultimately result in epithelial-to-mesenchymal transition (EMT), renal fibrosis, and nephron loss, novel pharmacological targets focus on the modulation of molecular and cellular cascades related to the deposition of extracellular matrix proteins or fibrinogenesis [58]. Most of these studies have suggested that classical oxidative stress and inflammation biomarkers as well as the activation of epidermal growth factor receptor (EGFR), regulation of cell differentiation and migration pathways (such as Notch, Wnt, Hedgehog, and SOX9), myofibroblast-targeting strategies, TGFβ1 signaling blockers, and inhibition of the Wnt/β-catenin pathway are promising therapeutic approaches [59,60,61].

## 5. Plant Extracts Characterized in In Vitro and In Vivo Studies

According to the collected data, 398 plant species were identified as folk nephroprotective agents, most of which were native to Brazilian flora, resulting in 741 citations. Table 2 provides an overview of their taxonomic distribution, morphological structures, and folk indications. Among them, 235 genera belonging to 91 botanical families were identified. The most cited families with traditional use were Fabaceae, followed by Asteraceae and Rubiaceae (65, 50, and 33 citations, respectively). As for the number of species, the most common families were Fabaceae, followed by Asteraceae and Bignoniaceae (42, 25, and 18 plant species, respectively). These results agree with previous studies indicating that Fabaceae and Asteraceae are among the largest botanical families found in Brazilian territory, thus reinforcing their importance for medicinal use in the country [62].

Roots were the predominant morphological structure of nephroprotective drugs used in household remedies (28.7%), followed by leaves (28.2%), and whole plants (9.6%). The species most commonly cited as useful for the treatment of renal complaints in the context of Brazilian traditional medicine were *Persea americana* (13 citations), *Phyllanthus niruri*, and *Casearia sylvestris* (both with 10 citations). Among these, *P. niruri* appears to be the most widely investigated plant for kidney diseases, sharing scientific evidence of its biological and pharmacological efficacy attributed to lignans, which are regarded as putative bioactive components (Table 3, Table 4 and Tables S1 and S2). Extracts obtained from *P. americana* have demonstrated important effects, particularly after in vivo assays. However, pharmacological studies to confirm the nephroprotective therapeutic properties of *C. sylvestris* are lacking. Notably, *P. americana* is a plant that shares the highest number of use reports, but is an exotic (non-native) species. This fact is consistent with the higher availability of some species that are cultivated in domestic gardens and also points to the fact that the Brazilian population tends to fill the medicinal gaps not met by native species [63,64].

A detailed list of plant extracts with nephroprotective activity in vitro and in vivo is provided in Table 3 and Table 4. Although these studies reported the effectiveness of the extracts using distinct models, the minimal concentration at which significant effects were achieved, and the half-maximal inhibitory concentration (IC_50_) were the measurements presented. The most potent extract (Table 3) was the aqueous leaf extract of *Guazuma ulmifolia*, which displayed an in vitro protective effect at a concentration of 3.125 μg/mL in mouse mesangial cells (SV40MES13) exposed to high glucose concentrations, simulating diabetic glomerulosclerosis [65]. *G. ulmifolia* is a Brazilian native tree widely found in the country, with edible fruits and known locally as “mutamba”. Decoctions of the leaves, fruits, and stem bark are traditionally used to treat kidney stones and digestive and cardiovascular disorders [66]. Polar extracts obtained from the bark of *G. ulmifolia* are especially rich in procyanidin oligomers, consisting mainly of [4β → 8]-(−)-epicatechin units, including procyanidin B1 (epicatechin-(4β → 8)-catechin), procyanidin B2 (epicatechin-(4β → 8)-epicatechin), procyanidin B5 (epicatechin-(4β → 6)-epicatechin), procyanidin C1 ([epicatechin-(4β → 8)]2-epicatechin), cinnamtannin A2 ([epicatechin-(4β → 8)]3-epicatechin), and epiphyllocoumarin derivatives [67,68]. The cardioprotective and antihypertensive activities of this species have been demonstrated both in vitro and in vivo and are mainly attributed to the presence of proanthocyanidins and flavonoids [69]. Bioassay-guided fractionation was conducted with a *G. ulmifolia* bark acetone extract to inhibit angiotensin II binding to the hAT1 receptor, leading to the isolation of a number of bioactive condensed tannins; the most promising compounds are highly polymerized pentamer and hexamer proanthocyanidins [70].

Other potent extracts include *P. niruri*, a medicinal herb known in Brazil as “stone-breaker” and widely used in household remedies to eliminate renal and urinary calculi, and as diuretic [71]. A hot aqueous plant extract at 5 µg/mL significantly inhibited the endocytic response observed in Madin-Darby canine kidney cells (MDCK) exposed to calcium oxalate (CaOx) crystals without impairing cell viability [53]. Another study demonstrated that the infusion of *P. niruri* extract inhibited the in vitro growth of CaOx crystals and reduced their aggregation in human urine, which could be useful for the treatment of urolithiasis [72]. Oral administration of an aqueous plant extract (1.25 mg/mL/day) over 42 days in rats induced relaxation, elimination, and dissolution of bladder CaOx stones, possibly by preventing crystal aggregation and promoting glycosaminoglycan adsorption [73]. An important dose-dependent reversion of plasma uric acid in hyperuricemic rats was displayed by the extract and apolar fractions of *P. niruri* leaves at 50 mg/kg, leading to the isolation of phyllanthin (**42**), a lignan with anti-hyperuricemic properties comparable to that of allopurinol at a dose of 23.9 µmol/kg [74]. A mechanistic investigation showed that the anti-hyperuricemic activity of the *P. niruri* methanol extract in animals was associated with the induction of uric acid excretion and in vitro (IC_50_ = 39.4 µg/mL) and in vivo inhibition of xanthine oxidase activity after intraperitoneal administration.

Studies have also shown that an ethanolic fraction of *Phyllanthus amarus* decoction induces urinary excretion and produces natriuretic effects at doses ranging from 5 to 80 mg/kg in rats, with evidence that the prostaglandin E2 pathway mediates the diuretic response [75]. The main active constituents of *P. amarus* are lignans such as phyllanthin and hypophyllanthin (**28**), which are also related to the pharmacological and biological activities of this species [76].

The hydroethanolic extract of *Costus spiralis* leaves, traditionally used in Brazilian folk medicine to treat pyelonephritis and kidney stones, significantly reversed renal function after cisplatin-induced nephrotoxic damage in rats at an oral dose of 5 mg/kg [77]. These effects were related to the presence of flavonoids, mainly apigenin glycosides, which partially validated the popular use of this plant. The infusion of *Euphorbia serpens* aerial parts increased urine volume in rats concomitantly with the loss of electrolytes in a dose-dependent manner, similar to that of the standard drug furosemide. D-mannitol was found to be the main constituent of the plant extract and was primarily associated with its diuretic activity [78]. *Bredemeyera floribunda* is a medicinal liana used as a remedy to control nephrolithiasis symptoms. A series of in vivo studies have been conducted using the root ethanolic extract, which resulted in a hypotensive response and a direct effect on the glomerular filtration rate after intravenous administration in antidiuretic or water diuretic rats [79,80]. The diuretic and saluretic responses of the extract were suggested to possibly occur via detergent-like interactions with Na^+^/K^+^-ATPase in proximal tubular cells, attributed to its triterpenoid saponins known as bredemeyerosides [81].

**Table 3:** Nephroprotective activities of extracts from Brazilian plants evaluated through in vitro and ex vivo assays.

Family	Specie	Morph. Struc.	Extract	Model	Effective Concentration(s)	Effects/Mechanisms	Reference
Asteraceae	*Ageratum conyzoides*	Leaf	EtOH	CaOx crystallization	10 mg	Antilithiatic effect	[82]
	*Lychnophora pinaster*	Aerial part	EtOH	Enzymatic activity	IC_50_ = 73.9 µg/mL	Inhibition of xanthine oxidase activity	[83]
			Hex				
			EtOAc		IC_50_ = 43.2 µg/mL		
			EtOH				
Bignoniaceae	*Sparattosperma leucanthum*	Leaf	EtOAc	Enzymatic activity	IC_50_ = 107 µg/mL	Inhibition of xanthine oxidase activity	[84]
			MeOH				
			AQ				
Costaceae	*Costus arabicus*	Whole plant	AQ	CaOx crystallization-induced MDCK-I cells	10, 100 µg/mL	Antilithiatic effect	[85]
Euphorbiaceae	*Euphorbia hirta*	Leaf	AQ, EtOAc, HEX, MeOH	CaOx crystallization	1 mg/mL	Antilithiatic effect	[86]
Fabaceae	*Copaifera langsdorffii*	Leaf	HA	CaOx crystallization	0.3, 0.7, 1 mg/mL	Antilithiatic effect	[87]
Lauraceae	*Persea americana*	Fruit, seed	Oil	Rotenone-induced VERO cells	1, 3, 10, 30, 100, 300, 600, 1000 µg/mL	Cytoprotective effect	[88]
		Leaf	Extract (*n.d.*)				
Malvaceae	*Guazuma ulmifolia*	Leaf	AQ	Glu-induced glomerulosclerosis in HRM cells	3.25, 6.25 μg/mL	Reduction in fibronectin levels	[65]
	*Theobroma grandiflorum*	Fruit	Pulp	High glucose-induced mouse immortalized mesangial cells	10, 50, 100 μg/mL	Antiproliferative and anti-inflammatory effects	[89]
Phyllanthaceae	*Phyllanthus niruri*	Whole plant	AQ	CaOx crystallization	0.0625, 0.125, 0.25, 0.5, 1 mg/mL	Inhibition of CaOx crystals growth and aggregation	[72]
		Whole plant	AQ	CaOx crystallization-induced MDCK	5, 10, 50, 100, 500, 1000 μg/mL	Inhibitory effect on the CaOx crystal internalization	[53]
		Leaf	MeOH	Enzymatic activity	IC_50_ = 39.4 µg/mL	Inhibition of xanthine oxidase activity	[90]
			F1		IC_50_ = 427.7 µg/mL		
			F2		IC_50_ = 86.9 µg/mL		
			F3		IC_50_ = 28.6 µg/mL		
			F4		IC_50_ = 22.7 µg/mL		
		Leaf	MeOH	CaOx crystallization	50 mg/mL	Antilithiatic effect	[91]
			AQ		50 mg/mL		

AQ, Aqueous, CaOx, Calcium oxalate, EtOAc, Ethyl acetate, EtOH, Ethanol, F, Fraction, Glu, Glucose, HEX, Hexane, HRM, Human renal mesangial cells, MDCK, Madin-Darby canine kidney cells, MeOH, Methanol, Morph. Struc., Morphological structure, n.d., not described, VERO, Monkey kidney epithelial cells line.

**Table 4:** Nephroprotective activities of extracts obtained from Brazilian plants evaluated through in vivo assays.

Family	Species	Morph. Struc.	Extract	Experimental Model	Route of Administration	Effective Dose (s)	Pharmacological Activities	Reference
Alismataceae	*Aquarius grandiflorus*	Leaf	AQ (EtOH-F)	Male Wistar rats	p.o.	300 mg/kg	Diuretic and hypotensive effects	[92]
	*Aquarius macrophyllus*	Leaf	HA	Gentamicin- induced kidney injury in male Wistar rats	p.o.	30 mg/kg	Antidiuretic and nephroprotective effects	[93]
Apiaceae	*Centella asiatica*	Leaf	EtOH	STZ-induced diabetic nephropathy in male Wistar rats	p.o.	400 mg/kg	Reduction in glomerular and vascular injuries	[94]
		Leaf	EtOH	Subtotal nephrectomy-induced nephropathy in male Swiss mice	p.o.	840 mg/kg	Reduction in kidney fibrosis and renal injury	[95]
Aquifoliaceae	*Ilex paraguariensis*	Leaf and stem	AQ	K_2_Cr_2_O_7_-induced nephropathy in Wistar rats	p.o.	540 to 600 mg	Improvement of glomerular filtration rate and nephroprotective effect	[96]
Araceae	*Pistia stratiotes*	Leaf	EtOH	Male Wistar rats	p.o.	200, 400 mg/kg	Diuretic effect	[97]
		Whole plant	HA	Renal ischemia and reperfusion-induced damage in diabetic male Sprague Dawley rats	p.o.	100 mg/kg	Nephroprotective, antiapoptotic, and anti-inflammatory effects	[98]
Arecaceae	*Acrocomia aculeata*	Ripe fruit	Pulp oil	Male Wistar rats	p.o.	300, 700 mg/kg	Diuretic effect	[99]
Asteraceae	*Acanthospermum hispidum*	Aerial part	AQ (EtOH-F)	Male Wistar rats	p.o., i.d.	30, 100, 300 mg/kg	Acute hypotensive and absence of diuretic effect	[100]
		Aerial part	AQ (EtOH-F)	Female ovariectomized Wistar rats	p.o.	30, 100, 300 mg/kg	Reduction in renovascular hypertension. Saluretic effect	[101]
	*Ageratum conyzoides*	Leaf	AQ	Gentamicin and diet-induced hyperoxaluria and CaOx deposition in male Wistar rats	p.o.	400 mg/kg	Antiurolithiatic effect	[102]
	*Baccharis trimera*	Aerial parts	AQ (EtOH-F)	Female Wistar rats exposed to cholesterol, diabetes and tobacco cigarettes	p.o.,	30, 100, 300 mg/kg	Nephroprotective effect in glomeruli, tubules, interstitium, and vessels	[103]
		Aerial parts	AQ (EtOH-F)	Male Wistar rats exposed to hookah, alcohol, and energy drink	p.o.	30, 100, 300 mg/kg	Nephroprotective effect	[104]
		Aerial parts	AQ (EtOH-F)	Male Wistar rats exposed to cholesterol, diabetes and tobacco cigarettes	p.o.	30, 100, 300 mg/kg	Nephroprotective effect	[105]
	*Eclipta prostrata*	Leaf	HA	Cisplatin-induced kidney injury in male Wistar rats	p.o.	400, 600 mg/kg	Nephroprotective effect	[106]
		Leaf	MeOH	Gentamicin- induced kidney injury in female Sprague Dawley rats	p.o.	300, 600 mg/kg	Nephroprotective effect	[107]
		Leaf	HA	Male Sprague Dawley rats	p.o.	14, 28 mg/kg	Nephroprotective effect. Increase in renal 11β- hydroxysteroid dehydrogenase activity	[108]
	*Lychnophora pinaster*	Aerial part	EtOH	Oxonate-induced hyperuricemia in male Swiss mice	p.o.	40, 125, 375 mg/kg	Hypouricemic and anti-inflammatory effects	[109]
	*Sonchus oleraceus*	Aerial part	EtOH	Renal ischemia and reperfusion-induced damage in male Wistar rats	p.o.	300 mg/kg	Nephroprotective effect	[110]
Bignoniaceae	*Bignonia binata*	Leaf	EtOH	CCl_4_-induced nephrotoxicity in male albino rats	p.o.	300 mg/kg	*n.e.*	[111]
			PE				*n.e.*	
			EtOAc				Nephroprotective effect	
			AQ				Nephroprotective effect	
	*Sparattosperma leucanthum*	Leaf	EtOAc	Oxonate-induced hyperuricemia in male Swiss mice	p.o.	125, 250, 500 mg/kg	Hypouricemic and anti-inflammatory effects	[84]
			MeOH					
			AQ					
Bromeliaceae	*Ananas comosus*	Ripe fruit	EtOH	Oxalate-induced urolithiasis in male Wistar rats	p.o.	500, 750, 1000 mg/kg	Antilithiatic and diuretic effects	[112]
Celastraceae	*Monteverdia ilicifolia*	Leaf	EtOAc-F	Male Wistar rats	p.o.	30, 100 mg/kg	Diuretic effect. Increase in urinary excretion of Na^+^ and sparing effect of K^+^ and Cl^−^	[113]
Costaceae	*Costus spiralis*	Leaf	HA	Cisplatin-induced kidney injury in Wistar rats	p.o.	5, 15, 30 mg/kg	Nephroprotective effect	[77]
Equisetaceae	*Equisetum giganteum*	Whole plant	CHCl_3_	CD1 strain mice	p.o.	50 mg/kg	Diuretic effect. Increase in urinary excretion of Na^+^, Cl^−^ and K^+^	[114]
Euphorbiaceae	*Euphorbia hirta*	Whole plant	EtOH	Nitrobenzene- induced nephrotoxicity in female Wistar rats	p.o.	400 mg/kg	Nephroprotective effect	[115]
	*Euphorbia serpens*	Aerial parts	AQ	Male Wistar rats	p.o.	5, 10, 20 mg/mL	Diuretic activity, increase in urinary excretion of Na^+^	[78]
	*Euphorbia thymifolia*	Whole plant	EtOH	Ethylene glycol- induced urolithiasis in male Wistar rats	p.o.	250, 500 mg/kg	Antilithiatic and diuretic effects	[116]
Fabaceae	*Abrus precatorius*	Stem bark	MeOH	Gentamicin- induced kidney injury in male Wistar rats	p.o.	100, 200 mg/kg	Nephroprotective, antiapoptotic and anti-inflammatory effects	[117]
	*Bauhinia forficata*	Leaf	AQ	Normotensive male Wistar rats	p.o.	300 mg/kg	Diuretic, anti-natriuretic, and antikaliuretic effects	[118]
			MeOH			100, 300 mg/kg	Diuretic effect	
			EtOAc- BuOH-F			30, 100 mg/kg	Diuretic, anti-natriuretic, and antikaliuretic effects	
			CHCl_3_-F			100 mg/kg	Diuretic, anti-natriuretic, and antikaliuretic effects	
	*Copaifera langsdorffii*	Leaf	HA	Ethylene glycol- induced nephrolithiasis in male Wistar rats	p.o.	40, 80, 160 mg/kg	Nephroprotective and antilithiatic effects	[87]
	*Mucuna pruriens*	Leaf	HA	CCl_4_ and rifampicin-induced nephrotoxicity in male Wistar rats	p.o.	50, 100 mg/kg	Nephroprotective effect	[119]
		Seed	AQ	Arsenic-induced nephrotoxicity in male Wistar rats	p.o.	350, 530, 700 mg/kg	Nephroprotective effect	[120]
	*Senna alata*	Leaf	AQ	Acetaminophen- induced nephrotoxicity in male Sprague Dawley rats	p.o.	200 mg/kg	Nephroprotective effect	[121]
		Leaf	HA	STZ-induced diabetic nephropathy in Wistar rats	p.o.	400 mg/kg	Nephroprotective effect	[122]
	*Senna occidentalis*	Leaf	AQ	Male and female Wistar rats	p.o.	240, 320, 400 mg/kg	Diuretic and saluretic effects	[123]
Lamiaceae	*Vitex megapotamica*	Leaf	MeOH: H_2_O (7:3)	High fat diet- induced nephropathy in C57BL/6 LDLr-null mice	p.o.	300 mg/kg	Nephroprotective effect	[124]
Lauraceae	*Persea americana*	Seed	AQ	Cadmium-induced nephrotoxicity in male Wistar rats	p.o.	400 mg/kg	Nephroprotective, antioxidant and anti-inflammatory effects	[125]
		Leaf	AQ	Nicotinamide and STZ-induced diabetic nephropathy in male Wistar rats	p.o.	100 mg/kg	Nephroprotective effect	[126]
			MeOH					
			EtOH					
		Fruit pulp	Oil	Male diabetic Goto- Kakizaki rats	p.o.	1 mL/250 g	Modulation of the redox state of kidney mitochondrial glutathione	[127]
		Fruit pulp	AQ	Cadmium-induced nephrotoxicity in male Wistar rats	p.o.	10% of the diet	Nephroprotective effect. Restoration of memory and learning disabilities caused by cadmium	[128]
		Leaf	HA	Ethylene glycol and NH_4_Cl-induced urolithiasis in male Sprague Dawley rats	p.o.	100, 300 mg/kg	Antiurolithic and nephroprotective effects	[129]
Lythraceae	*Cuphea carthagenensis*	Leaf	AQ (EtOH-F)	Ovariectomized hypertensive female Wistar rats	p.o.	30, 100, 300 mg/kg	Cardiorenal protective effect, preservation of renal function	[130]
Malvaceae	*Ceiba pentandra*	Aerial part	EtOAc-F	Methotrexate- induced kidney injury in male Wistar rats	p.o.	400 mg/kg	Nephroprotective, antiapoptotic and anti-inflammatory effects	[131]
	*Sida rhombifolia*	Aerial part	HA	STZ-induced diabetic nephropathy in male Wistar rats	*n.d.*	100, 200 mg/kg	Nephroprotective effect	[132]
	*Theobroma cacao*		Polyphenol -F	CCl_4_-induced nephrotoxicity in male F344 rats	p.o.	500 mg/kg	Nephroprotective effect	[133]
	*Theobroma grandiflorum*	Fruit	Pulp	STZ-induced diabetic nephropathy in male Wistar rats	p.o.	1 g/mL	Nephroprotective and anti-inflammatory effects	[89]
Menispermaceae	*Cissampelos pareira*	Root	AQ	Ethylene glycol and NH_4_Cl-induced urolithiasis in male albino rats	p.o.	100, 200, 400 mg/kg	Antiurolithic and nephroprotective effects	[134]
		Whole plant	HA	Acetaminophen- induced nephrotoxicity in male albino rats	p.o.	200, 400 mg/kg	Nephroprotective effect	[135]
Myrtaceae	*Eugenia uniflora*	Leaf	HA	Renal ischemia and reperfusion-induced acute kidney injury in male Wistar rats	p.o.	200 mg/kg	Stabilization of glomerular filtration rate, renal blood flow and renal vascular resistance parameters	[136]
		Leaf	AQ	Normotensive male Wistar rats	p.o.	120 mg d.L./kg	Diuretic effect. Reduction in Na^+^ excretion.	[137]
Passifloraceae	*Passiflora edulis*	Fruit peel	AQ	STZ-induced diabetic nephropathy in male Wistar rats	p.o.	400 mg/kg	Nephroprotective effect	[138]
		Fruit peel	EtOH	Gentamicin- induced kidney injury in male albino rats	p.o.	250, 500 mg/kg	Nephroprotective effect	[139]
Phyllanthaceae	*Phyllanthus amarus*	Whole plant	EtOH	High salt diet- induced renal metabolic derangement in male Wistar rats	p.o.	75, 100, 150 mg/kg	Nephroprotective effect	[140]
		Leaf	HA	Rifampicin-induced nephrotoxicity in male Wistar rats	p.o.	50, 100 mg/kg	Nephroprotective effect	[141]
		Leaf	AQ	Glycolate-induced hyperoxaluria in male Wistar rats	p.o.	3.5 mg	Diuretic effect. Reduction in oxalate crystal deposition of Ca^2+^ kidney content	[142]
		Aerial part	AQ	Gentamicin and acetaminophen- induced kidney injury in male Wistar rats	p.o.	100, 200, 400 mg/kg	Nephroprotective effect	[143]
		Whole plant	AQ (EtOH-F)	Male Wistar rats	i.p.	5, 10, 20, 40, 80 mg/kg	Diuretic and natriuretic effects	[75]
	*Phyllanthus niruri*	Whole plant	AQ	CaOx-induced urolithiasis in male albino rats	p.o.	1.25 mg/mL	Inhibitory effect of crystal growth	[73]
		Whole plant	AQ	CaOx-induced urolithiasis in male albino rats	p.o.	5 mg	Inhibitory effect of crystal deposition	[144]
		Leaf	MeOH	Oxonate and uric acid-induced hyperuricemia in male Sprague Dawley rats	i.p.	50 mg/kg	Uricosuric effect, inhibition of xanthine oxidase activity	[90]
			F 1				*n.e.*	
			F 2					
			F 3				Inhibition of xanthine oxidase activity	
			F 4				Inhibition of xanthine oxidase activity	
		Leaf	MeOH	Oxonate and uric acid-induced hyperuricemia in male Sprague Dawley rats	i.p.	50, 100, 200, 500, 1000 mg/kg	Antihyperuricemic effect	[74]
			F 1			50 mg/kg	*n.e.*	
			F 2			50 mg/kg		
			F 3			50 mg/kg		
			F 4			50 mg/kg	Antihyperuricemic effect	
		Leaf	AQ	STZ-induced diabetic nephropathy in male Wistar rats	p.o.	200, 400 mg/kg	Nephroprotective effect	[145]
		Leaf	AQ	STZ/nicotinamide-induced diabetic nephropathy in male Wistar rats	p.o.	200, 400 mg/kg	Nephroprotective, anti-inflammatory, antiapoptotic and antifibrotic effects	[146]
	*Phyllanthus sellowianus*	Stem bark	AQ	Female Sprague Dawley rats	p.o.	400 mg/kg	Diuretic effect	[147]
	*Phyllanthus tenellus*	Aerial parts	AQ (EtOH-F)	Male Sprague Dawley rats	p.o.	30, 100, 300 mg/kg	Absence of diuretic effect	[148]
Piperaceae	*Piper peltatum*	Root	HA	Female albino rats	p.o.	25, 100, 200 mg/kg	Diuretic and saluretic effect	[149]
	*Piper umbellatum*	Leaf	MeOH	Atherogenic diet- induced renal injury in male Syrian Golden hamsters	p.o.	0.25 and 1 g/kg	Nephroprotective effect	[150]
Polygalaceae	*Bredemeyera floribunda*	Root	EtOH	Male Wistar rats	i.v.	15, 30 mg/kg	Diuretic, natriuretic, and kaliuretic effects	[79]
						0.05 mg/100 g	Increase in glomerular filtration rate, fractional water and sodium excretion and solute clearance	[80]
Portulacaceae	*Portulaca pilosa*	Leaf, stem	HA	Male Wistar rats	p.o.	400 mg/kg	Kaliuretic effects	[151]
Pteridaceae	*Adiantum capillus-veneris*	Aerial part	HA	Ethylene glycol and NH_4_Cl-induced urolithiasis in male Sprague Dawley rats	p.o.	127.6, 255.2 mg/kg	Antiurolithic and nephroprotective effects	[152]
Rhamnaceae	*Ampelozizyphus amazonicus*	Root	HA (n-BuOH- F)	Furosemide-induced diuresis in male Wistar rats	p.o.	50 mg/kg	Antidiuretic effect	[153]
		Root	EtOH	Male Wistar rats	p.o.	100 mg/kg	Diuretic effect	[154]
			Saponin-F			50, 100, 200, 1000 mg/kg	Antidiuretic effect	
			Saponin- free F			50, 100, 200 mg/kg	Diuretic effect	
Rubiaceae	*Alibertia edulis*	Leaf	AQ	Male Wistar rats	i.d.	200 mg/kg	Diuretic effect. Increased excretion of Na^+^, Cl^−^, K^+^, Ca^2+^	[155]
	*Palicourea coriacea*	Aerial part	HA	Male Wistar rats	p.o.	20, 40, 80 mg/kg	Diuretic, natriuretic, and kaliuretic effects	[156]
	*Rudgea viburnoides*	Leaf	AQ	2K1C-hypertensive male Wistar rats	p.o.	30, 100, 300 mg/kg	Preservation of urine excretion and electrolyte levels. Reduction in the progression of cardiorenal disease	[157]
		Leaf	HA	Gentamicin- induced kidney injury in male Wistar rats	p.o.	50, 200 mg/kg	Nephroprotective effect	[158]
Sapindaceae	*Cardiospermum halicacabum*	Whole plant	MeOH, PE	Acetaminophen- induced nephrotoxicity in Wistar rats	p.o.	400 mg/kg	Nephroprotective effect	[159]
		Whole plant	AQ	Gentamicin- induced kidney injury in albino rats	p.o.	200, 400 mg/kg	Nephroprotective effect	[160]
Talinaceae	*Talinum paniculatum*	Leaf, stem	AQ (EtOH-F)	Male Wistar rats	p.o.	30, 100, 300 mg/kg	Diuretic and saluretic effect	[161]
		Leaf	HA	2K1C-hypertensive male Wistar rats	p.o.	100, 300 mg/kg	Diuretic and nephroprotective effects	[162]
Urticaceae	*Cecropia pachystachya*	Leaf	EtOH (AQ-F)	Subtotal nephrectomy- induced nephropathy in male Wistar rats	p.o.	0.5 g/kg	Nephroprotective and anti-inflammatory effects	[163]
		Leaf	AQ	Subtotal nephrectomy- induced nephropathy in male Wistar rats	p.o.	0.6 g/kg	Reduction in glomerulosclerosis and of the urinary excretion of MCP-1 and TGF-β	[164]
	*Laportea aestuans*	Leaf	MeOH	Arsenite and STZ-induced nephrotoxicity in Wistar rats	p.o.	200 mg/kg	Nephroprotective effect	[165]
	*Urera baccifera*	Root, leaf, stem	AQ	Male Wistar rats	p.o.	400 mg/kg	Diuretic effect	[166]
Violaceae	*Anchietea pyrifolia*	Leaf	AQ (EtOH-F)	Male Wistar rats	p.o.	30, 100, 300 mg/kg	Absence of diuretic effect. Decreased urine excretion of Na^+^, K^+^, and Cl^−^	[167]

2K1C 2-Kidney, 1-clip surgery, AQ aqueous, CaOx calcium oxalate, CCl_4_ carbon tetrachloride, CHCl_3_ chloroform, EtOAc ethyl acetate, EtOH ethanol, F fraction, HA hydroalcoholic, i.d. intraduodenal administration, i.v. intravenous, K_2_Cr_2_O_7_ potassium dichromate, MeOH methanol, n-BuOH n-butanol, n.e. no effect, NH_4_Cl ammonium chloride, PE petroleum ether, STZ streptozotocin, mg/kg milligram/kilogram, Morph. Struc. morphological structure, n.d. not described, n.e. no effect, p.o. oral administration.

## 6. In Vitro and In Vivo Studies with Plant Secondary Metabolites

Although successful, natural product-based drug discovery for kidney-related diseases has some drawbacks. It is not uncommon to observe a reduction or loss of biological activity throughout the bioactivity-guided fractionation process, possibly because of synergistic interactions among the compounds in unrefined phytocomplex mixtures [168]. Nevertheless, in contrast to extracts and synthetic compounds, single nephroprotective molecules may offer some special features to the limited available therapeutic arsenal, including their diverse chemical diversity and reduction in active doses/concentrations due to the purification of extracts [169]. Tables S1 and S2 of Supporting Information show 74 secondary metabolites belonging to the classes of alkaloids, anthraquinones, coumarins, flavonoids, lignans, phenylpropanoids, saponins, and triterpenes with in vitro and in vivo nephroprotective activities, respectively. The structures of bioactive secondary metabolites are shown in Figure S1. Likewise, as the studies reported the effectiveness of the isolated compounds using distinct models, the minimal concentration at which significant effects were achieved, and the half-maximal inhibitory concentration (IC_50_) were the measurements presented.

Surprisingly, phytochemical studies of some plant species based on ethnomedical information have resulted in the identification of potent nephrotoxins. For example, aristolochic acid, a compound commonly found in the roots of *Aristolochia* species and used medicinally for kidney ailments, is associated with nephropathy, renal interstitial fibrosis, and urothelial cancer [170]. Aristolochic acid is currently used in models to induce CKD and screen for new nephroprotective drugs [171]. Controversially, five species from the *Aristolochia* genus are used as folk blood depuratives and diuretics in Brazil, as shown in Table 2. This fact points to the relevant risks associated with their use of self-care as home remedies.

Polyphenols are among the most potent nephroprotective compounds that have been assayed in vitro. For example, scopoletin (**51**), a coumarin found in a number of medicinal plants, such as *Persea americana* and *Sida rhombifolia*, displayed significant protection in a model of diabetic glomerulosclerosis in rat glomerular mesangial cells exposed to high glucose [172]. At a concentration of 0.1 µM, this compound reduced cell proliferation, inhibited the overexpression of ECM proteins, and reduced connective tissue growth factor and TGF-ꞵ expression, proving that scopoletin could be a potential antifibrotic agent against diabetes-induced nephropathy. Another highlight is that the anthraquinone emodin (**16**), which is found in *Handroanthus impetiginosus* and *Senna occidentalis*, displayed an in vitro protective effect at 0.5 µM against cisplatin-induced damage in human kidney (HEK 293) cells, mostly because of its antioxidant properties [173]. Delphinidin (**14**), an anthocyanidin commonly found in medicinal edible fruit trees such as *Euterpe precatoria* and *Plinia peruviana*, reduced the oxidative injury caused by antimycin a, patulin and insulin in epithelial rat kidney (NRK) cells at 1 µM [174]. Ferulic acid (**20**), quercetin (**48**), kaempferol (**32**), epicatechin (**17**), and wedelolactone (**57**) all displayed significant protective effects against nephrotoxic agents at a concentration of 1 µM in renal cell lines [175,176,177,178,179]. The triterpenes betulinic acid (**6**) and ursolic acid (**55**) were also effective in mitigating the damage induced by different toxins at concentrations of 0.25 and 1 µM, respectively [180,181].

Several secondary metabolites present in the selected medicinal plants have undergone in vitro to in vivo tests, and their nephroprotective properties have been characterized. For example, it has been suggested that the beneficial properties against nephrolithiasis symptoms described for *P. niruri* preparations can be attributed to lignans, a class of compounds that are particularly found in other medicinal herbs used to treat kidney-related disorders belonging to the *Phyllanthus* genus, such as *P. amarus*, *P. tenellus,* and *P. sellowianus* [182]. As previously mentioned, bio-guided fractionation of *P. niruri* methanol extract and fractions that displayed potent anti-hyperuricemic oral effects in animals afforded three lignans, of which phyllanthin (**42**) at 10 mg/kg displayed significant and dose-dependent uricosuric action [74]. Although phyllanthin, hypophyllanthine (**28**), and phyltetralin (**43**) alone showed no appreciable effect owing to possible synergistic interactions, the first significantly induced changes in urine output and uric acid content in hyperuricemic rats [90]. Therefore, the uricosuric and antiurolithic properties of lignans from *Phyllanthus* spp. may provide attractive therapeutic alternatives for the management of hyperuricemia and urinary stones.

The flavonoids afzelin (**1**) and kaempferitrin (**31**), both kaempferol glucosides isolated from the medicinal plant *Bauhinia forficata*, demonstrated significant diuretic and saluretic effects after oral administration to rats at low concentrations (0.1 mg/kg) [118,183]. Moreover, afzelin presented acute and subchronic Ca^2+^-sparing and renoprotective effects in normotensive and hypertensive rats, as well as antiurolithiatic effects in synthetic and rat urine [183], which may support the ethnopharmacological use of this herb for kidney ailments. In a type-1 diabetic model, subcutaneous administration of 0.78 mg/kg/day of apigenin (**4**) for 10 days attenuated nephropathy features in rats by decreasing the overexpression of dynamin-related protein 1 (Drp1) in kidney tissues [184]. Similarly, epicatechin (**17**) counteracted the progression of renal damage in rats subjected to a subtotal nephrectomy at a dose of 0.01 mg/kg administered orally for 14 days, preserving renal function and systolic blood pressure [185].

In addition to the in vitro results, oral in vivo pretreatment with betulinic acid (**6**) mitigated the damage induced by nephrotoxic T-2 mycotoxins in mice [186]. At 0.25 mg/kg, this triterpene reduced the inflammatory response and renal oxidative damage via Nrf2 signaling pathway activation. These results suggest the possibility of conducting clinical trials using betulinic acid to limit the progression of renal disease in humans.

## 7. Conclusions

According to the literature review, there is a great diversity of medicinal plants popularly used for the treatment of kidney disorders in Brazil, but only a few of them have been preclinically tested for their potential nephroprotective effects. In contrast, the number of isolated secondary metabolites found in the aforementioned plants was higher, which demonstrates a greater interest in validating the pharmacological potential of single molecules, particularly polyphenols and triterpenes. New nephroprotective agents are needed for the therapeutic arsenal because the drugs currently available are not entirely satisfactory. Flavonoids appeared to be the leading class of compounds investigated for renal disorders, highlighting quercetin, rutin, kaempferol, apigenin, fisetin, and luteolin. Phenylpropanoids, including caffeic, chlorogenic, ellagic, gallic and rosmarinic acids, also shared a large number of scientific reports. Finally, asiatic acid, betulinic acid, lupeol, and ursolic acid were identified as important bioactive nephroprotective triterpenes in this study. Considering that many of these molecules may occur concomitantly in the compiled folk medicinal species, more vigorous efforts directed towards isolation (bioguided assay), as well as clinical assays for potent extracts and/or isolated compounds, are required to establish effectiveness and toxicological data and to ensure their potential use.

## 8. Future Prospects

A significant number of medicinal plants are commonly used in traditional Brazilian medicine to relieve the symptoms associated with renal diseases. Additionally, available biological and pharmacological reports suggest that several species have their use examined and pre-validated and are considered safe in toxicological studies. This study provides a comprehensive overview of the diverse secondary metabolites belonging to different chemical classes found in the most cited plants, in vitro and in vivo investigations, and their efficacy, presenting the most common mechanisms and active doses/concentrations required to reduce kidney damage. Future research efforts should be directed towards the identification of active compounds for plant species, given the still limited data available on isolated nephroprotective phytochemicals. Complementary information on revealing and comparing the pharmacological potencies of extracts and their derived isolated molecules and clinical trials are also relevant future challenges for the development of new drugs. Experimental findings published over the last few years have confirmed their high potency, offering unique and safe multitarget approaches for the modulation of different signaling pathways involved in kidney diseases. Based on the search conducted, alkaloids, anthraquinones, coumarins, flavonoids, lignans, phenylpropanoids, saponins, and triterpenes provided satisfactory preclinical efficacy and could be recommended for use alone or concomitantly with available nephroprotective synthetic drugs. A drug discovery program to provide nephroprotective products in biodiverse regions is essential for expediting the development of novel products for the benefit of humanity, which has an important impact in underdeveloped countries.

## Figures and Tables

**Table 1:** List of books consulted in the process of survey and of the main Brazilian biomes involved (Amazon Forest, Cerrado, Atlantic Forest, Caatinga, Pantanal, and Pampa).

Brazilian Bioma	References
Amazon Forest	[32,33,34,35,36,37,38,39,40,41,42]
Atlantic Forest	[32,33,34,35,38,39,40,42,43,44,45,46]
Caatinga	[33,34,35,38,39,40,42,43,44,47,48]
Cerrado	[33,34,35,36,37,38,39,40,42,44,49,50]
Pampa	[33,34,35,38,39,40,42,46]
Pantanal	[33,34,35,36,37,38,39,40,42,51]

**Table 2:** Plant species used in Brazilian ethnomedicine as nephroprotective agents.

Family	Scientific Name	Vernacular Name	Origin	Traditional Use	Morphological Structure Used	Reference
Alismataceae	*Aquarius grandiflorus* (Cham. & Schltr.) (synonym of *Echinodorus grandiflorus* (Cham. & Schltr.) Micheli)	Chapéu-de-couro	N	Kidney disorders, diuretic, blood depurative	Leaf	[32]
				Diuretic, kidney disorders	Leaf	[43]
				Diuretic, blood depurative, kidney disorders	Leaf	[33]
	*Aquarius macrophyllus* (Kunth) Christenh. and Bing. (synonym of *Echinodorus macrophyllus* (Kunth) Micheli)	Chapéu-de-couro	N	Kidney disorders	Whole plant	[34]
				Blood impurities, nephritis, diuretic	Leaf	[35]
				Diuretic, blood depurative, kidney disorders	Leaf	[36]
				Kidney disorders	Leaf	[37]
				Diuretic, blood depurative, kidney diseases	Leaf	[38]
				Diuretic	Leaf	[39]
				Diuretic, blood depurative	Leaf	[44]
Amaranthaceae	*Alternanthera brasiliana* (L.) Kuntze	Sempre-viva	N	Diuretic, blood depurative	Leaf	[33]
	*Alternanthera ficoidea* (L.) P.Beauv (synonym of *A. tenella* Colla)	Perpétua-branca	N	Diuretic, blood depurative	Whole plant	[44]
	*Chamissoa altissima* (Jacq.) Kunth	Fumo-bravo	N	Diuretic	Root	[47]
	*Iresine diffusa* Humb. and Bonpl. ex Willd.	Bredinho	N	Diuretic	Leaf	[47]
	*Pfaffia jubata* Mart.	Lã-de-carneiro	N	Blood depurative	Whole plant	[37]
Amaryllidaceae	*Hymenocallis tubiflora* Salisb.	Cebola-brava-do-Pará	N	Diuretic	n.d.	[35]
Anacardiaceae	*Anacardium occidentale* L.	Cajueiro	N	Blood depurative, diuretic	Resin, fruit stalk, fruit	[32]
				Blood depurative	Bark, gum	[33]
				Diuretic	Fruit	[40]
	*Astronium fraxinifolium* Schott	Gonçalo-alves	N	Kidney disorders	Bark	[48]
	*Schinus molle* L.	Aroeira	N	Diuretic	n.d.	[33]
	*Schinus terebinthifolia* Raddi	Aroeira-vermelha	N	Blood depurative, diuretic	Bark	[32]
				Diuretic	Leaf, bark, fruit	[41]
				Blood depurative, diuretic	Bark, fruit	[45]
	*Spondias mombin* L.	Cajazeira	N	Kidney diseases	Bark, fruit peel	[48]
Annonaceae	*Annona coriacea* Mart.	Araticum	N	Diuretic	Leaf	[48]
	*Annona spinescens* Mart.	Araticum-de-espinho	N	Blood depurative	Fruit	[35]
	*Xylopia aromatica* (Lam.) Mart.	Árvore-de-espinho	N	Diuretic	Leaf	[49]
	*Xylopia frutescens* Aubl.	Breu	N	Diuretic	Leaf	[32]
Apiaceae	*Centella asiatica* (L.) Urb.	Centela	E	Diuretic	Whole plant	[44]
				Blood depurative, diuretic	Leaf	[32]
				Blood depurative	Stalk	[34]
				Blood depurative, diuretic	n.d.	[39]
	*Eryngium foetidum* L.	Coentro-fedorento	N	Diuretic	Whole plant	[47]
				Diuretic	Root	[44]
				Diuretic	n.d.	[35]
	*Eryngium pristis* Cham. and Schltdl.	Língua-de-tucano	N	Diuretic	Leaf	[50]
Apocynaceae	*Allamanda cathartica* L.	Alamanda	N	Kidney disorders	Leaf	[44]
	*Aspidosperma quebracho-blanco* Schltdl.	Quebracho	N	Diuretic	Bark	[39]
	*Himatanthus obovatus* (Müll. Arg.) Woodson	Angélica	N	Blood depurative	Leaf	[51]
				Blood depurative	Leaf	[37]
	*Mandevilla illustris* (Vell.) Woodson	Jalapa	N	Renal dropsy	n.d.	[49]
	*Mandevilla velame* (A.St.-Hil.) Pichon	Velame-branco	N	Blood depurative	Root	[49]
				Blood depurative	Root	[35]
				Diuretic, blood depurative, kidney disorders	Leaf, root	[36]
				Blood depurative	Root	[37]
				Blood depurative	Root, whole plant	[50]
				Blood depurative	Root	[40]
				Blood depurative	n.d.	[34]
				Blood depurative	Root	[44]
	*Ruehssia amylacea* (Barb.Rodr.) F.Esp.Santo and Rapini	Condurango	N	Blood depurative	n.d.	[34]
Aquifoliaceae	*Ilex conocarpa* Reissek	Congonha	N	Diuretic	n.d.	[34]
	*Ilex diuretica* Mart. ex Reissek	Congonha	N	Diuretic	Leaf, stem	[44]
	*Ilex paraguariensis* A.St.-Hil.	Erva-mate	N	Kidney disorders	n.d.	[34]
				Kidney colic	Leaf	[39]
				Diuretic	n.d.	[45]
Araceae	*Philodendron bipinnatifidum* Schott ex Endl. (synonym of *Thaumatophyllum bipinnatifidum* (Schott ex Endl.) Sakur., Calazans & Mayo)	Cipó-imbé	N	Diuretic	Leaf	[45]
	*Pistia stratiotes* L.	Aguapé	N	Diuretic, kidney disorders	Leaf	[35]
				Diuretic	Leaf	[33]
Araliaceae	*Hydrocotyle bonariensis* Comm. ex Lam.	Açariçoba	N	Diuretic	Root	[35]
				Diuretic	Rhizome	[33]
				Diuretic	Rhizome	[44]
	*Hydrocotyle leucocephala* Cham. and Schltdl.	Acariçoba	N	Diuretic, renal indisposition	Rhizome	[45]
Arecaceae	*Acrocomia aculeata* (Jacq.) Lodd. ex Mart.	Bocaiúva	N	Diuretic	Root	[51]
	*Copernicia alba* Morong	Palmeira-carandá	N	Diuretic	Root	[35]
				Diuretic	Root	[51]
	*Copernicia prunifera* (Mill.) H.E.Moore	Carnaúba-branca	N	Blood depurative	Root	[48]
				Diuretic	Root	[35]
				Diuretic	Root	[34]
				Diuretic	Root	[39]
	*Euterpe precatoria* Mart.	Açaí	N	Kidney disorders, blood depurative	Root	[41]
	*Syagrus comosa* (Mart.) Mart.	Catolé	N	Kidney stones	Root	[48]
Aristolochiaceae	*Aristolochia cymbifera* Mart. and Zucc.	Angelicó	N	Diuretic	n.d.	[33]
				Diuretic, blood depurative	Root	[35]
				Diuretic	Root	[47]
				Diuretic	Root	[40]
	*Aristolochia esperanzae* Kuntze	Milhomem	N	Blood depurative	n.d.	[36]
				Kidney disorders	n.d.	[51]
	*Aristolochia labiata* Willd.	Papo-de-peru	N	Diuretic	Root	[39]
				Diuretic	Root	[47]
				Diuretic	Root	[40]
	*Aristolochia triangularis* Cham.	Cipó-mil-homens	N	Blood depurative, diuretic	n.d.	[45]
	*Aristolochia trilobata* L.	Mil-homens	N	Diuretic	Root	[32]
Asparagaceae	*Herreria salsaparilha* Mart.	Salsaparrilha-verdadeira	N	Blood depurative	Root	[50]
				Blood depurative	Root	[44]
Asteraceae	*Acanthospermum australe* (Loefl.) Kuntze	Carrapicho-rasteiro	N	Kidney disorders, diuretic	Root	[32]
				Diuretic	Whole plant	[39]
	*Acanthospermum hispidum* DC.	Maroto	N	Kidney disorders	Whole plant	[43]
				Kidney stones	Leaf, root	[36]
	*Ageratum conyzoides* L.	Erva-de-São-João	N	Blood depurative	Root, leaf	[48]
				Diuretic	Whole plant	[39]
				Diuretic	Whole plant	[44]
	*Baccharis crispa* Spreng.	Carqueja	N	Kidney disorders, diuretic	Leaf	[32]
				Diuretic	Aerial parts	[46]
				Kidney disorders, diuretic, blood depurative	Whole plant	[35]
				Diuretic	Whole plant	[44]
				Diuretic, kidney disorders, blood depurative	Whole plant	[40]
	*Bidens gardneri* Baker	Picão	N	Diuretic	n.d.	[51]
	*Blainvillea acmella* (L.) Philipson	Agrião-bravo	E	Blood depurative	Flower, leaf, branch, whole plant	[48]
	*Chaptalia nutans* (L.) Pol.	Língua-de-vaca	N	Blood depurative	Root	[48]
	*Conyza bonariensis* (L.) Cronquist)	Voadeira	N	Kidney disorders	Leaf, root	[36]
				Diuretic		[51]
	*Eclipta prostrata* (L.) L.	Surucuína	N	Blood depurative	n.d.	[35]
	*Egletes viscosa* (L.) Less.	Macela	N	Kidney disorders	Inflorescence, leaf, seed	[48]
	*Elephantopus hirtiflorus* DC.	Língua-de-vaca	N	Diuretic, urinary stones	Root, leaf	[40]
	*Elephantopus mollis* Kunth	Erva-grossa	N	Diuretic	Root	[47]
				Diuretic, kidney stones	Root, leaf	[33]
				Diuretic, urinary stones	Root, leaf	[40]
	*Lychnophora pinaster* Mart.	Arnica-da-montanha-mineira	N	Kidney disorders	Stem, leaf, flower	[44]
	*Melampodium divaricatum* (Rich.) DC.	Picão-da-praia	N	Diuretic	Leaf	[35]
	*Mikania cordifolia* (L.f.) Willd.	Erva-de-cobra	N	Diuretic	Whole plant	[39]
				Diuretic	Leaf	[47]
	*Mikania glomerata* Spreng.	Guaco	N	Blood depurative	Leaf	[33]
				Blood depurative	Leaf	[44]
	*Mikania guaco* Kunth	Guaco	N	Diuretic	Whole plant	[35]
	*Mikania hirsutissima* DC.	Cipó-cabeludo	N	Diuretic, nephritis	n.d.	[33]
				Nephritis, diuretic	Whole plant	[50]
				Nephritis	n.d.	[34]
				Diuretic, nephritis	Flower	[38]
				Diuretic, kidney disorders	Whole plant	[44]
	*Mikania setigera* Sch. Bip. ex Baker	Orelha-de-cachorro	N	Diuretic, nephritis	Stem, leaf	[39]
				Blood depurative	Leaf, root	[44]
	*Orthopappus angustifolius* (Sw.) Gleason	Língua-de-vaca	N	Blood depurative	n.d.	[36]
	*Pluchea sagittalis* (Lam.) Cabrera	Lucera	N	Kidney inflammation	Aerial parts	[33]
	*Solidago chilensis* Meyen	Arnica	N	Blood depurative	n.d.	[51]
	*Sonchus oleraceus* L.	Serralha	N	Blood depurative	n.d.	[35]
				Blood depurative	n.d.	[34]
				Diuretic	Whole plant	[33]
				Blood depurative	n.d.	[36]
	*Vernonanthura ferruginea* (Less.) H.Rob.	Assa-peixe	N	Diuretic, blood depurative	Leaf	[49]
				Diuretic, blood depurative	Root	[51]
				Diuretic, blood depurative	Root	[50]
				Diuretic, blood depurative	Leaf	[37]
	*Vernonanthura polyanthes* (Sprengel) Vega and Dematt.	Assa-peixe	N	Diuretic, kidney stones	Leaf, root	[33]
				Diuretic	Whole plant	[44]
Begoniaceae	*Begonia bidentata* Raddi	Erva-de-sapo	N	Diuretic	n.d.	[35]
	*Begonia riedelii* A.DC.	Saracura	N	Diuretic	n.d.	[35]
	*Begonia sanguinea* Raddi	Erva-de-sapo-da-vermelha	N	Diuretic	n.d.	[35]
Bignoniaceae	*Anemopaegma arvense* (Vell.) Stellfeld ex de Souza	Alecrim-do-campo	N	Blood depurative	n.d.	[36]
	*Bignonia binata* Thunb.	Cipó-camarão	N	Blood depurative	n.d.	[34]
	*Cybistax antisyphilitica* (Mart.) Mart.	Caroba-de-flor-verde	N	Blood depurative	Bark	[41]
				Diuretic, nephroprotective	Leaf, bark, root	[34]
	*Dolichandra unguis-cati* (L.) L.G.Lohmann	Unha-de-gato	N	Diuretic	Leaf, bark	[35]
				Diuretic	Leaf, bark	[51]
	*Handroanthus heptaphyllus* (Vell.) Mattos	Piúva	N	Blood depurative	Bark	[51]
	*Handroanthus impetiginosus* (Mart. ex DC.) Mattos	Cinco-folhas	N	Kidney disorders	Stem bark, flower	[48]
				Kidney stones	Leaf, stem	[43]
				Diuretic	Bark	[33]
	*Handroanthus ochraceus* (Cham.) Mattos	Piúva-cascuda	N	Nephroprotective	Leaf	[51]
	*Handroanthus serratifolius* (Vahl) S.O.Grose	Ipê	N	Diuretic, blood depurative	Bark	[39]
	*Jacaranda brasiliana* (Lam.) J. St.-Hil.	Caroba	N	Blood depurative	Stem bark, flower	[48]
	*Jacaranda caroba* (Vell.) DC. (synonym of *J. oxyphylla* Cham.)	Caroba	N	Blood depurative	Leaf	[32]
				Diuretic	Stem bark	[50]
				Diuretic	Leaf, stem	[44]
				Blood depurative	Leaf	[47]
	*Jacaranda cuspidifolia* Mart.	Carobão	N	Blood depurative	Stem bark	[36]
				Blood depurative	*n.d.*	[51]
	*Jacaranda decurrens* Cham.	Carobinha-branca	N	Blood depurative	Leaf, root, stem bark	[36]
				Blood depurative	Stem bark, leaf	[50]
	*Jacaranda micrantha* Cham.	Caroba	N	Diuretic, blood depurative	n.d.	[40]
	*Jacaranda puberula* Cham.	Carobinha-do-campo	N	Blood depurative	Leaf, root, stem bark	[36]
	*Pyrostegia venusta* (Ker Gawl.) Miers	Cipó-de-São-João	N	Kidney disorders	Flower, branch	[44]
	*Sparattosperma leucanthum* (Vell.) K.Schum.	Ipê-batata	N	Diuretic, blood depurative	Leaf	[35]
	*Tabebuia aurea* (Silva Manso) Benth. and Hook.f. ex S.Moore	Paratudo	N	Diuretic, blood depurative	Bark, sprouts	[49]
				Diuretic	n.d.	[51]
	*Zeyheria montana* Mart.	Bolsa-de-pastor	N	Blood depurative	Root bark	[34]
Bixaceae	*Bixa arborea* Huber	Urucum	N	Diuretic	n.d.	[32]
	*Bixa orellana* L.	Urucum	N	Diuretic	Root	[47]
				Diuretic, kidney disorders	Leaf, seed, root	[38]
				Kidney disorders	Leaf, seed	[44]
				Diuretic	Root	[40]
	*Cochlospermum regium* (Mart. ex Schrank) Pilg.	Algodão-do-campo	N	Blood depurative	Root	[36]
				Blood depurative	Bark	[37]
Boraginaceae	*Cordia ecalyculata* Vell.	Café-de-bugre	N	Renal edema	Leaf	[35]
				Diuretic	n.d.	[33]
				Diuretic	n.d.	[34]
	*Cordia rufescens* A.DC.	Baba-de-boi	N	Kidney disorders	Leaf, stem	[43]
	*Heliotropium elongatum* (Lehm.) Gürke	Crista-de-galo	N	Diuretic	Root, flower, leaf	[47]
				Diuretic	Root, flower, leaf	[40]
	*Heliotropium indicum* L.	Aguaraciunha	N	Diuretic	Leaf, root, flower	[33]
				Diuretic		[51]
	*Myriopus paniculatus* (Cham.) Feuillet	Marmelinho-do-campo	N	Diuretic, kidney disorders, kidney stones	Leaf, stem	[44]
Brassicaceae	*Lepidium bonariense* L.	Mastruço-de-Buenos-Aires	N	Diuretic	n.d.	[35]
				Diuretic	Leaf	[34]
Bromeliaceae	*Ananas ananassoides* (Baker) L.B.Sm.	Ananás	N	Diuretic	Fruit	[50]
	*Ananas comosus* (L.) Merr.	Abacaxi	N	Kidney disorders	Fruit, leaf	[43]
				Diuretic	Fruit	[47]
				Diuretic	Fruit	[33]
				Kidney stones	Fruit	[34]
				Diuretic	Fruit	[44]
	*Bromelia antiacantha* Bertol.	Caraguatá	N	Diuretic, kidney stones	Fruit	[33]
	*Tillandsia aeranthos* (Loisel.) L.B.Sm.	Cravo-do-mato	N	Diuretic	Whole plant	[46]
Burseraceae	*Commiphora leptophloeos* (Mart.) J.B.Gillett	Imburana-de-espinho	N	Nephroprotective	Stem bark	[48]
	*Crescentia cujete* L.	Coité	E	Diuretic	Leaf	[48]
Cactaceae	*Cereus jamacaru* DC.	Mandacaru	N	Kidney disorders	Root	[33]
	*Opuntia monacanthos* (Willd.) Haw.	Palmatória	N	Diuretic	Fruit	[35]
Calophyllaceae	*Caraipa grandifolia* Mart.	Tamaquaré	N	Blood depurative	Bark	[35]
Cannaceae	*Canna glauca* L.	Embira	N	Diuretic	Whole plant	[47]
				Diuretic	n.d.	[34]
				Diuretic	Whole plant	[35]
	*Canna indica* L.	Bananeirinha	N	Diuretic	Whole plant	[47]
				Diuretic	Fresh leaf	[35]
	*Canna paniculata* Ruiz and Pav.	Bananeira-do-mato	N	Diuretic	Root	[35]
Capparaceae	*Morisonia hastata* (Jacq.) Christenh. and Byng (synonym of *Cynophalla hastata* (Jacq.) J.Presl)	Sapotaia	N	Diuretic	Root bark	[35]
Cardiopteridaceae	*Citronella gongonha* (Mart.) R.A.Howard	Congonha-de-bugre	N	Nephroprotective	n.d.	[34]
Caryocaraceae	*Caryocar villosum* (Aubl.) Pers.	Pirquiá	N	Diuretic	Bark	[35]
Celastraceae	*Monteverdia ilicifolia* (Mart. ex Reissek) Biral (synonym of *Maytenus ilicifolia* Mart. ex Reissek)	Espinheira-santa	N	Kidney disorders, blood depurative	Leaf, root	[36]
				Diuretic	Leaf	[46]
				Kidney disorders, diuretic	Leaf	[35]
				Diuretic	Leaf	[38]
				Kidney disorders	Leaf	[39]
				Kidney disorders, diuretic	Leaf	[40]
	*Monteverdia rigida* (Mart.) Biral (synonym of *Maytenus rigida* Mart.)	Bom-nome	N	Nephroprotective	n.d.	[40]
Chloranthaceae	*Hedyosmum brasiliense* Mart. ex Miq.	Chá-de-soldado	N	Diuretic	Leaf	[44]
Commelinaceae	*Commelina erecta* L.	Erva-mijona	N	Diuretic	Leaf	[47]
	*Tripogandra serrulata* (Vahl) Handlos	Trapoeiraba	N	Diuretic	Whole plant	[44]
Convolvulaceae	*Cuscuta racemosa* Mart.	Cipó-chumbo	N	Diuretic	Whole plant	[44]
	*Cuscuta umbellata* Kunth	Cipó-chumbo	N	Diuretic	Whole plant	[35]
	*Distimake tomentosus* (Choisy) Petrongari and Sim.-Bianch.	Velame-do-campo	N	Blood depurative	Branch, leaf, flower	[50]
	*Ipomoea imperati* (Vahl) Griseb.	Salsa-branca	N	Diuretic	Root, seed	[47]
	*Ipomoea pes-caprae* (L.) R.Br.	Salsa-da-praia	N	Diuretic, blood depurative	Root	[47]
				Diuretic	Root	[33]
	*Operculina hamiltonii* (G.Don) D.F.Austin and Staples	Batata-de-tiú	N	Blood depurative	Tuber	[35]
				Blood depurative	n.d.	[36]
	*Operculina macrocarpa* (L.) Urb.	Batata-de-purga	N	Blood depurative	Tuber, root	[48]
				Blood depurative	Root	[43]
				Blood depurative	Root	[33]
				Blood depurative	n.d.	[36]
				Blood depurative	Tuber	[44]
Costaceae	*Costus arabicus* L.	Cana-do-brejo	N	Diuretic, kidney disorders	Leaf, stem	[36]
	*Costus scaber* Ruiz and Pav.	Cana-de-macaco	N	Kidney disorders	Stem	[43]
	*Costus spiralis* (Jacq.) Roscoe	Cana-do-brejo	N	Diuretic	Leaf	[32]
				Diuretic, kidney disorders	Stem	[35]
				Diuretic, kidney disorders	Whole plant	[40]
Cucurbitaceae	*Apodanthera smilacifolia* Cogn. (synonym of *Melothrianthus smilacifolius* (Cogn.) Mart.Crov.)	Cipó-azougue	N	Blood depurative	Root	[35]
				Blood depurative	n.d.	[34]
				Blood depurative	Whole plant	[39]
				Blood depurative	Root, fruit	[44]
	*Cayaponia bonariensis* (Mill.) Mart.Crov.	Taiuiá	N	Blood depurative	Fruit	[35]
	*Cayaponia espelina* (Silva Manso) Cogn.	Espelina	N	Blood depurative	Root	[44]
	*Cayaponia podantha* Cogn.	Abóbora-d’anta	N	Blood depurative	Root	[44]
	*Cayaponia tayuya* (Vell.) Cogn.	Taiuiá	N	Blood depurative	Tuber, bark, root, aerial parts	[48]
				Blood depurative	Root	[47]
				Blood depurative	Tuber	[33]
				Blood depurative	Root	[39]
				Blood depurative	Root	[50]
				Blood depurative	Root	[44]
	*Cayaponia trilobata* Cogn.	Abóbora-d’anta	N	Blood depurative	Bark, root	[34]
	*Siolmatra brasiliensis* (Cogn.) Baill.	Taiuiá	N	Blood depurative	Root	[51]
Cyperaceae	*Cyperus aggregatus* (Willd.) Endl.	Tiririca	N	Diuretic	Root	[47]
	*Cyperus brevifolius* (Rottb.) Endl. ex Hassk.	Capim-cheiroso	N	Diuretic	Root	[47]
	*Cyperus compressus* L.	Paraturá	N	Diuretic	Root	[40]
	*Cyperus hermaphroditus* (Jacq.) Standl.		N	Diuretic	Root	[47]
	*Cyperus ligularis* L.	Capim-açu	N	Diuretic	Root	[47]
	*Cyperus pedunculatus* (R.Br.) J.Kern	Paraturá	E	Diuretic	n.d.	[47]
				Diuretic	Root	[40]
	*Cyperus sesquiflorus* (Torr.) Mattf. and Kük.	Capim-cidreira	N	Diuretic	Whole plant	[35]
				Diuretic	Root	[47]
Dilleniaceae	*Curatella americana* L.	Lixeira	N	Blood depurative	Flower	[35]
	*Davilla aspera* (Aubl.) Benoist (synonym of *Tetracera tigarea* DC.)	Cipó-vermelho-de-caiena	N	Blood depurative, diuretic	Leaf	[35]
	*Davilla rugosa* Poir.	Cipó-caboclo	N	Diuretic	Leaf, root	[44]
				Blood depurative	n.d.	[47]
				Diuretic	Branch	[50]
	*Doliocarpus dentatus* (Aubl.) Standl.	Cipó-caboclo-vermelho	N	Diuretic	Branch, root	[50]
	*Doliocarpus major* J.F.Gmel.	Cipó-d’água	N	Diuretic	Root, sap	[35]
Equisetaceae	*Equisetum giganteum* L.	Cavalinha	N	Kidney disorders, diuretic, generalized edema,	Sprout	[35]
				Diuretic, kidney infection, kidney disorders	Stem	[33]
Euphorbiaceae	*Cnidoscolus urens* (L.) Janti	Cansanção-de-leite	N	Diuretic	Root	[40]
	*Croton antisyphiliticus* Mart.	Pé-de-perdiz	N	Blood depurative	Leaf, root	[35]
				Blood depurative	Whole plant	[50]
				Diuretic	Whole plant	[39]
				Diuretic, blood depurative	Leaf, root, whole plant	[44]
	*Croton cajucara* Benth.	Sacaca	N	Kidney inflammation	Leaf	[33]
	*Croton campestris* A.St.-Hil.	Velame-do-campo	N	Blood depurative	Root	[47]
				Blood depurative	Root, leaf	[44]
				Blood depurative	Leaf, root	[39]
				Blood depurative	n.d.	[34]
				Diuretic	Leaf	[40]
	*Croton echioideus* Baill.	Quebra-faca	N	Kidney disorders	Whole plant, stem bark	[48]
	*Croton fulvus* Mart.	Velame-do-mato	N	Blood depurative	Leaf, root	[35]
	*Croton goyazensis* Müll.Arg.	Alcanforeira	N	Diuretic	Whole plant	[45]
	*Croton heliotropiifolius* Kunth	Velame	N	Blood depurative	Root	[48]
	*Croton salutaris* Casar.	Sangue-de-pau	N	Blood depurative	Bark	[35]
	*Euphorbia hirta* L.	Erva-andorinha	N	Diuretic	Leaf	[35]
				Diuretic	Leaf	[40]
	*Euphorbia hyssopifolia* L.	Sete-sangrias	N	Blood depurative	Whole plant	[36]
	*Euphorbia prostrata* Aiton	Quebra-pedra	N	Diuretic, kidney disorders	n.d.	[46]
	*Euphorbia serpens* Kunth	Caá-cambuí	N	Diuretic	Leaf	[35]
	*Euphorbia thymifolia* L.	Leite-de-Nossa-Senhora	N	Diuretic	n.d.	[51]
	*Jatropha elliptica* (Pohl) Oken	Purga-de-lagarto	N	Blood depurative	Root	[37]
				Diuretic	Leaf	[40]
				Blood depurative	Root	[51]
	*Jatropha gossypiifolia* L.	Pinhão-roxo	N	Diuretic	Root	[33]
	*Joannesia princeps* Vell.	Anda-assú	N	Diuretic	Seed	[39]
Fabaceae	*Abrus precatorius* L.	Jequiriti	E	Diuretic	Root	[33]
	*Anadenanthera colubrina* (Vell.) Brenan	Angico	N	Blood depurative	Bark	[33]
				Blood depurative	Bark	[51]
				Blood depurative	Bark	[39]
	*Anadenanthera macrocarpa* (Benth.) Brenan	Angico	N	Blood depurative	Stem bark	[48]
				Blood depurative	Bark	[47]
	*Bauhinia dubia* G.Don	Pata-de-vaca	N	Kidney disorders	Leaf	[36]
	*Bauhinia forficata* Link	Pata-de-vaca	N	Diuretic	Leaf	[38]
				Diuretic, kidney stones	n.d.	[45]
				Diuretic	Leaf	[32]
				Diuretic, kidney stones	Leaf	[33]
				Diuretic, kidney disorders	Leaf	[46]
				Kidney disorders, depurative	Whole plant	[41]
				Diuretic	Leaf, root, flower	[47]
				Diuretic, blood depurative	Leaf	[40]
	*Bauhinia holophylla* (Bong.) Steud.	Unha-de-vaca	N	Diuretic	Whole plant	[50]
	*Bauhinia pentandra* (Bong.) Vogel ex Steud.	Mororó-de-espinho	N	Kidney disorders	Stem bark, leaf	[48]
	*Bauhinia rufa* (Bong.) Steud.	Unha-de-vaca	N	Diuretic	Whole plant	[50]
	*Bowdichia nitida* Spruce ex Benth.	Sapupira-da-mata	N	Blood depurative	Root, seed	[35]
	*Bowdichia virgilioides* Kunth	Sucupira	N	Blood depurative	Root, seed	[35]
				Blood depurative	Stem bark, seed	[36]
				Blood depurative	Tuber, root bark, seed	[39]
				Blood depurative	Root, seed	[51]
				Blood depurative	Tuber, seed	[34]
				Blood depurative	Seed	[50]
				Blood depurative	Bark	[40]
	*Centrosema angustifolium* (Kunth) Benth.	Espia-caminho	N	Diuretic	n.d.	[40]
	*Centrosema brasilianum* (L.) Benth.	Espia-caminho	N	Diuretic	n.d.	[40]
	*Centrosema pascuorum* Mart. ex Benth.	Espia-caminho	N	Diuretic	n.d.	[40]
	*Centrosema plumieri* (Turpin ex Pers.) Benth.	Espia-caminho	N	Diuretic	n.d.	[40]
	*Chamaecrista rotundifolia* (Pers.) Greene	Quebra-pedra	N	Diuretic, kidney disorders	Whole plant	[50]
	*Clitoria guianensis* (Aubl.) Benth.	Catuaba-falsa	N	Diuretic	Root, seed	[44]
				Diuretic	Root	[50]
	*Copaifera langsdorffii* Desf.	Pau-d‘óleo	N	Kidney disorders	Stem bark	[48]
				Kidney inflammation	Resin	[49]
				Diuretic	n.d.	[33]
	*Copaifera multijuga* Hayne	Copaíba	N	Kidney inflammation	Bark	[41]
	*Enterolobium contortisiliquum* (Vell.) Morong	Ximbuva	N	Nephroprotective	Bark	[51]
	*Grona barbata* (L.) H.Ohasi and K.Ohasi (synonym of *Desmodium barbatum* (L.) Benth.)	Trevinho	N	Diuretic	n.d.	[51]
	*Guilandina bonduc* L.	Carnica	N	Diuretic	Seed	[47]
	*Hymenaea courbaril* L.	Jatobá	N	Blood depurative	Stem bark, fruit, barks	[48]
				Kidney disorders	Stem, seed	[40]
				Kidney disorders	n.d.	[41]
	*Indigofera lespedezioides* Kunth	Purgueiro	N	Diuretic	n.d.	[51]
	*Indigofera suffruticosa* Mill.	Anileira	N	Diuretic	Root, leaf	[47]
				Diuretic	Root, leaf	[40]
				Diuretic	Whole plant	[50]
	*Libidibia ferrea* (Mart. ex Tul.) L.P.Queiroz	Pau-ferro	N	Kidney disorders	Pod	[32]
				Blood depurative	Stem bark, fruit, root, flower	[48]
				Diuretic	Stem bark	[36]
	*Machaerium acutifolium* Vogel	Bico-de-pato	N	Diuretic	Fruit	[49]
	*Mimosa candollei* R.Grether	Malícia-roxa	N	Diuretic	Root	[47]
	*Mucuna pruriens* (L.) DC.	Pó-de-mico	E	Diuretic	Root	[35]
	*Myroxylon balsamum* (L.) Harms	Pau-bálsamo	N	Diuretic	n.d.	[35]
				Diuretic	n.d.	[39]
	*Myroxylon peruiferum* L.f.	Bálsamo-do-Peru	N	Diuretic	n.d.	[39]
	*Periandra mediterranea* (Vell.) Taub.	Alcaçuz	N	Diuretic	Root	[35]
				Diuretic	Root	[34]
				Diuretic	n.d.	[36]
	*Pterodon emarginatus* Vogel	Sucupira-branca	N	Blood depurative	Bark	[40]
	*Pterodon pubescens* (Benth.) Benth.	Sucupira-branca	N	Blood depurative	Bark	[37]
				Blood depurative	Stem bark, seed	[36]
	*Schnella splendens* (Kunth) Benth.	Pata-de-vaca	N	Kidney disorders	n.d.	[36]
	*Senna alata* (L.) Roxb.	Mata-pasto	N	Blood depurative	n.d.	[51]
				Blood depurative	Leaf	[35]
				Diuretic	n.d.	[40]
	*Senna occidentalis* (L.) Link	Fedegoso	N	Diuretic	Root bark	[33]
				Diuretic	Root	[51]
				Diuretic	Root	[50]
				Diuretic, kidney diseases	Root	[32]
				Blood depurative	Seed, fruit, root	[48]
				Diuretic	Root bark	[35]
				Diuretic	Root, seed	[47]
				Diuretic	Leaf, root bark, root, seed	[38]
				Diuretic	Root bark, seed, leaf	[39]
	*Senna reticulata* (Willd.) H.S.Irwin and Barneby	Pé-de-São-João	N	Diuretic, kidney diseases	Root	[32]
	*Senna splendida* (Vogel) H.S.Irwin and Barneby	Fedegoso-grande	N	Diuretic	Root	[50]
	*Senna uniflora* (Mill.) H.S.Irwin and Barneby	Mata-pasto	N	Diuretic	n.d.	[40]
	*Stryphnodendron adstringens* (Mart.) Coville	Barbatimão	N	Blood depurative	Bark	[40]
	*Zornia latifolia* Sm.	Orelha-de-caxinguelê	N	Diuretic	n.d.	[51]
Francoaceae	*Viviania albiflora* (Cambess.) Reiche	Anavinga	N	Diuretic	Fruit	[35]
Gentianaceae	*Schultesia guianensis* (Aubl.) Malme	Mata-zombando	N	Blood depurative	n.d.	[47]
				Blood depurative	Leaf, root	[51]
Humiriaceae	*Humiria balsamifera* (Aubl.) A.St.-Hil.	Umiri	N	Diuretic	Bark	[35]
Iridaceae	*Eleutherine bulbosa* (Mill.) Urb.	Marupá	N	Diuretic	n.d.	[41]
	*Sisyrinchium vaginatum* Spreng.	Canchalágua	N	Diuretic	Whole plant	[46]
	*Trimezia martinicensis* (Jacq.) Herb.	Maririçó	N	Blood depurative	Rhizome, root	[35]
Lamiaceae	*Hyptis radicans* (Pohl) Harley and J.F.B.Pastore	Paracari	N	Diuretic	Stall, leaf	[35]
				Kidney stones	n.d.	[34]
				Diuretic, kidney inflammation	Branch, leaf	[50]
				Diuretic, kidney inflammation	Flower, whole plant	[44]
	*Ocimum campechianum* Mill.	Alfavaca	N	Diuretic	n.d.	[32]
	*Vitex cymosa* Bertero ex Spreng.	Tarumã	N	Kidney infection	Leaf, stem bark	[36]
				Blood depurative	Leaf	[51]
	*Vitex megapotamica* (Spreng.) Moldenke	Tarumã	N	Diuretic, blood depurative	n.d.	[35]
				Diuretic, blood depurative	Bark, fruit, leaf	[46]
	*Vitex polygama* Cham.	Azeitona-do-mato	N	Kidney disorders, diuretic	Leaf	[50]
				Blood depurative	Leaf	[44]
	*Vitex triflora* Vahl	Tarumã-da-mata	N	Blood depurative, diuretic	Leaf, root, fruit	[35]
Lauraceae	*Cassytha filiformis* L.	Cipó-chumbo	N	Kidney disorders	n.d.	[51]
	*Mespilodaphne cymbarum* (Kunth) Trofimov (synonym of *Ocotea cymbarum* Kunth)	Sassafrás	N	Blood depurative	n.d.	[40]
	*Nectandra mollis* (Kunth) Nees (synonym of *N. reticulata* Mez)	Louro-preto	N	Diuretic	Bark	[35]
	*Ocotea aurantiodora* (Ruiz and Pav.) Mez (synonym of *O. longifolia* Kunth)	Caneleira-de-cheiro	N	Diuretic	Root	[35]
	*Ocotea odorifera* (Vell.) Rohwer	Sassafrás	N	Blood depurative, diuretic	Bark, flower, oil	[33]
				Blood depurative	Bark	[37]
				Blood depurative	Stem bark, root	[44]
				Blood depurative	n.d.	[40]
	*Persea americana* Mill.	Abacateiro	E	Kidney stones	Fruit	[44]
				Diuretic, kidney disorders	Leaf	[39]
				Diuretic	Leaf	[38]
				Kidney disorders	Leaf	[37]
				Diuretic	Leaf	[34]
				Diuretic, kidney disorders	Leaf	[36]
				Diuretic, anti-infective for the kidneys	Stem bark, seed, leaf	[33]
				Diuretic	Leaf, sprout	[47]
				Diuretic	Stem bark, leaf, seed	[41]
				Diuretic	Leaf	[42]
				Kidney stones, diuretic, kidney disorders	Leaf, seed, fruit	[48]
				Kidney stones, diuretic, kidney disorders	Leaf	[32]
				Diuretic	Leaf, sprout	[40]
Lecythidaceae	*Lecythis pisonis* Cambess.	Sapucaia	N	Diuretic	n.d.	[35]
Linderniaceae	*Torenia crustacea* (L.) Cham. and Schltdl. (synonym of *Lindernia crustacea* (L.) F.Muell.)	Douradinha-do-Pará	N	Diuretic	Leaf	[35]
				Diuretic	Leaf	[40]
	*Vandellia diffusa* L. (synonym of *Lindernia diffusa* (L.) Wettst.)	Douradinha-do-campo	E	Diuretic	Leaf	[35]
				Diuretic, blood depurative	n.d.	[40]
				Diuretic	n.d.	[34]
Loganiaceae	*Strychnos pseudoquina* A.St.-Hil.	Quina	N	Blood depurative	Bark, wood	[51]
Lycopodiaceae	*Lycopodium clavatum* L.	Licopódio	N	Diuretic	Spore, whole plant	[44]
Lythraceae	*Cuphea carthagenensis* (Jacq.) J.F.Macbr.	Sete-sangrias	N	Diuretic, blood depurative	Whole plant	[33]
				Blood depurative	Leaf, flower, stem, branch, root	[38]
				Blood depurative	Whole plant	[50]
				Blood depurative, diuretic	Whole plant, root	[48]
	*Cuphea ingrata* Cham. and Schltdl.	Sete-sangrias	N	Blood depurative	n.d.	[35]
Malpighiaceae	*Banisteriopsis argyrophylla* (A.Juss.) B.Gates	Cipó-prata	N	Diuretic, kidney disorders, kidney stones, nephritis	Root, branch, leaf, flower	[50]
				Kidney diseases	Root, leaf, stem	[44]
	*Banisteriopsis campestris* (A.Juss.) Little	Cipó-prata	N	Diuretic, kidney disorders, kidney stones, nephritis	Root, branch, leaf, flower	[50]
	*Banisteriopsis laevifolia* (A.Juss.) B.Gates	Cipó-prata	N	Diuretic, kidney disorders, kidney stones, nephritis	Root, branch, leaf, flower	[50]
	*Banisteriopsis megaphylla* (A.Juss.) B.Gates	Cipó-prata	N	Diuretic, kidney disorders, kidney stones, nephritis	Root, branch, leaf, flower	[50]
	*Byrsonima intermedia* A.Juss.	Saratudo	N	Diuretic	n.d.	[41]
	*Byrsonima pachyphylla* A.Juss.	Muruci	N	Diuretic	Branch, leaf	[50]
	*Byrsonima verbascifolia* (L.) DC.	Douradinha-falsa	N	Diuretic at higher doses	n.d.	[49]
				Diuretic	Whole plant	[35]
				Diuretic	Leaf, stem	[50]
	*Heteropterys argyrophaea* A.Juss.	Cipó-prata	N	Diuretic, kidney diseases	n.d.	[34]
	*Heteropterys banksiifolia* Griseb.	Guiné-do-campo	N	Diuretic	Leaf	[50]
	*Heteropterys tomentosa* A.Juss.	Nó-de-cachorro	N	Blood depurative	Root	[37]
				Blood depurative	Root	[36]
				Blood depurative	n.d.	[51]
Malvaceae	*Ayenia melastomifolia* (A.St.-Hil) Christenh and Byng (synonym of *Byttneria melastomifolia* A.St.-Hil.)	Raiz-de-bugre	N	Blood depurative	Leaf, root	[36]
				Blood depurative	Root	[37]
	*Ceiba pentandra* (L.) Gaertn.	Sumaúma-da-várzea	N	Diuretic	Bark	[35]
	*Guazuma ulmifolia* Lam.	Mutamba	N	Blood depurative	Bark	[39]
				Blood depurative	Bark	[35]
	*Helicteres baruensis* Jacq.	Umbigo-de-bezerro	N	Renal insufficiency	Fruit	[48]
	*Herissantia crispa* (L.) Brizicky	Malva	N	Diuretic	Leaf	[47]
	*Luehea paniculata* Mart.	Açoita-cavalo	N	Blood depurative	Root	[49]
				Blood depurative	n.d.	[36]
	*Sida rhombifolia* L.	Guanxuma	E	Kidney stones	Whole plant	[32]
				Diuretic	Seed	[46]
				Diuretic	Leaf	[44]
	*Theobroma cacao* L.	Cacaueiro	N	Diuretic	Seed	[39]
				Diuretic	Seed	[44]
	*Theobroma grandiflorum* (Willd. ex Spreng.) K.Schum.	Cupuaçu	N	Kidney infections	Leaf	[32]
	*Triumfetta rhomboidea* Jacq. (synonym of *T. bartramia* L.)	Carrapichão	E	Diuretic	Whole plant	[50]
	*Triumfetta semitriloba* Jacq.	Carrapicho-de-calçada	N	Blood depurative	Root	[48]
				Diuretic	Whole plant	[39]
				Diuretic	Whole plant	[44]
	*Urena lobata* L.	Urena	N	Diuretic	Root	[50]
				Diuretic	Root	[32]
				Diuretic	Leaf	[35]
				Diuretic, kidney colic	Root, flower	[44]
	*Waltheria communis* A.St.-Hil.	Douradinha	N	Diuretic	n.d.	[51]
				Diuretic	Bark, leaf	[33]
				Blood depurative	n.d.	[34]
Melastomataceae	*Mouriri elliptica* Mart.	Coroa-de-frade	N	Nephroprotective	Fruit	[51]
	*Pleroma asperius* (Cham.) Triana	Douradinha	N	Diuretic	n.d.	[46]
Meliaceae	*Trichilia elegans* A.Juss.	Cachuá	N	Diuretic	Bark	[51]
Menispermaceae	*Chondrodendron platyphyllum* (A.St.-Hil.) Miers	Abatua	N	Diuretic	Root	[39]
	*Chondrodendron tomentosum* Ruiz and Pav.	Parreira-brava	N	Nephrolithiasis	n.d.	[35]
	*Cissampelos glaberrima* A.St.-Hil.	Abútua	N	Diuretic	Root	[35]
				Diuretic	Root	[50]
				Diuretic	Leaf, bark, root	[44]
	*Cissampelos ovalifolia* DC.	Orelha-de-onça	N	Diuretic	Root	[50]
	*Cissampelos pareira* L.	Buta	N	Diuretic, kidney stones	Root, stem bark	[35]
				Diuretic	Root	[51]
	*Cissampelos sympodialis* Eichler	Angelicó	N	Blood depurative	Aerial parts	[48]
				Diuretic	Root	[47]
	*Cissampelos tropaeolifolia* DC.	Parreira-brava-do-rio	N	Nephrolithiasis	n.d.	[35]
Moraceae	*Brosimum acutifolium* Huber	Mururé	N	Blood depurative	Latex	[35]
	*Brosimum gaudichaudii* Trécul	Mamacadela	N	Blood depurative	Leaf, root	[33]
				Diuretic	n.d.	[51]
				Blood depurative	Branch, leaf	[50]
				Blood depurative	Leaf, stem, root	[44]
				Diuretic, blood depurative	Root, bark, root	[49]
				Blood depurative	n.d.	[36]
	*Dorstenia brasiliensis* Lam.	Figueirilha	N	Diuretic	Whole plant	[46]
				Diuretic	Root	[47]
				Diuretic	n.d.	[51]
				Diuretic	Root	[40]
	*Dorstenia cayapia* Vell.	Caapiá	N	Diuretic	Rhizome	[35]
				Diuretic	Leaf, root, rhizome, fruit	[33]
	*Dorstenia cayapia* subsp. *asaroides* (synonym of *D. asaroides* Gardner)	Carapiá	N	Blood depurative	n.d.	[36]
	*Ficus crocata* (Miq.) Mart. ex Miq.	Oiti	N	Blood depurative	Bark	[34]
	*Ficus gomelleira* Kunth	Figueira	N	Blood depurative	n.d.	[51]
	*Pseudolmedia macrophylla* Trécul	Mururé	N	Blood depurative	Bark	[34]
				Blood depurative	Bark	[39]
Myrtaceae	*Eugenia biflora* (L.) DC.	Rosário-de-jambu	N	Diuretic	Root	[35]
	*Eugenia involucrata* DC.	Pitanga-do-mato	N	Diuretic	Leaf	[50]
	*Eugenia punicifolia* (Kunth) DC.	Pitanga-de-folha-fina	N	Diuretic	Stem bark, leaf	[50]
	*Eugenia uniflora* L.	Pitangueira	N	Diuretic	Leaf	[46]
				Kidney infection	Leaf	[36]
				Diuretic	Leaf, fruit	[47]
	*Myrcia vellozoi* Mazine	Uoapurama	N	Diuretic	Root, seed, bark	[35]
	*Plinia peruviana* (Poir.) Govaerts (synonym of *P. cauliflora* (Mart.) Kausel)	Jaboticaba	N	Kidney stones	Stem, fruit	[43]
				Diuretic	Fruit, bark	[44]
	*Psidium guineense* Sw.	Araçá-do-campo	N	Diuretic	Root	[51]
				Diuretic	Root, stem bark	[50]
Nyctaginaceae	*Neea theifera* Oerst.	Caparrosa-do-campo	N	Kidney disorders	n.d.	[34]
Ochnaceae	*Sauvagesia erecta* L.	Erva-de-São-Martinho	N	Diuretic	n.d.	[35]
				Diuretic	n.d.	[51]
Olacaceae	*Ximenia americana* L.	Limão-bravo	N	Blood depurative	Bark	[51]
Oxalidaceae	*Oxalis hirsutissima* Mart. and Zucc.	Azedinha	N	Kidney infection	Leaf	[36]
Passifloraceae	*Passiflora alata* Curtis	Maracujá	N	Diuretic	Leaf	[38]
	*Passiflora edulis* Sims	Maracujá	N	Diuretic	Leaf	[35]
			N	Diuretic	Leaf	[34]
			N	Kidney disorders	Bark	[37]
			N	Diuretic	Leaf	[40]
	*Passiflora quadrangularis* L.	Maracujá-açu	N	Diuretic, blood depurative	Fruit	[35]
	*Piriqueta duarteana* (Cambess.) Urb.	Chanana	N	Renal lithiasis, pyelonephritis	Leaf, root	[48]
	*Turnera diffusa* Willd. ex. Schult	Damiana	N	Diuretic	n.d.	[35]
				Diuretic, nephroprotective	Leaf	[39]
Phyllanthaceae	*Phyllanthus acutifolius* Poir. ex Spreng.	Erva-pombinha	N	Diuretic, kidney colic	n.d.	[34]
	*Phyllanthus amarus* Schumach. and Thonn.	Quebra-pedra	N	Kidney disorders	Whole plant	[43]
				Diuretic, kidney stones	n.d.	[51]
	*Phyllanthus brasiliensis* (Aubl.) Poir.	Conami	N	Diuretic	Root	[35]
	*Phyllanthus niruri* L.	Quebra-pedra	N	Kidney stones, diuretic, kidney colic	Whole plant	[35]
				Diuretic	n.d.	[41]
				Diuretic, kidney stones	Whole plant	[44]
				Diuretic, kidney stones, kidney colic	Whole plant	[40]
				Kidney stones, nephroprotective	Whole plant, root, leaf	[48]
				Kidney stones, kidney pain	n.d.	[42]
				Kidney stones, diuretic	Whole plant	[33]
				Diuretic, kidney disorders, kidney stones	Whole plant	[36]
				Kidney disorders	Leaf	[37]
				Diuretic, kidney stones, nephritis	Aerial parts, flower, root, seed	[38]
	*Phyllanthus niruri* subsp. *lathyroides* (Kunth) G.L.Webster (synonym of *P. lathyroides* Kunth)	Quebra-pedra	N	Kidney stones	Root	[47]
	*Phyllanthus orbiculatus* Rich.	Quebra-pedra	N	Diuretic, kidney stones	n.d.	[51]
	*Phyllanthus sellowianus* (Klotzsch) Müll.Arg.	Quebra-pedra	N	Diuretic, kidney stones, kidney colic	Whole plant	[40]
	*Phyllanthus tenellus* Roxb.	Quebra-pedra-falso	E	Diuretic, lithiasis	Whole plant	[44]
	*Phyllanthus tenellus* Roxb. var. *tenellus* (synonym of *P. corcovadensis* Müll.Arg.)	Quebra-pedra	E	Diuretic, kidney stones	Leaf, root	[32]
Petiveriaceae	*Seguieria americana* L.	Tapiá	N	Diuretic	n.d.	[34]
	*Gallesia integrifolia* (Spreng.) Harms	Pau-alho	N	Nephroprotective	n.d.	[51]
Piperaceae	*Peperomia pellucida* (L.) Kunth	Erva-jaboti	N	Diuretic	n.d.	[41]
				Diuretic	Whole plant	[33]
	*Piper aduncum* L.	Pimenta-do-mato	N	Diuretic	Fruit	[51]
	*Piper bartlingianum* (Miq.) C.DC.	Nhambi	N	Diuretic	n.d.	[34]
	*Piper cernuum* Vell.	Pariparoba	N	Kidney disorders	Leaf	[32]
	*Piper marginatum* Jacq.	Pariparoba	N	Diuretic	Root	[32]
				Diuretic	Root	[35]
				Diuretic, kidney diseases	Whole plant	[47]
				Blood depurative, diuretic	n.d.	[45]
	*Piper peltatum* L.	Caapeba	N	Diuretic	Root, leaf	[32]
				Diuretic, kidney stones	Whole plant	[47]
				Diuretic	Root, leaf	[40]
	*Piper regnellii* (Miq.) C.DC.	Capeba-do-Brasil	N	Kidney disorders	Leaf	[44]
	*Piper tuberculatum* Jacq.	Pimenta-de-macaco	N	Renal insufficiency	Flower, fruit	[48]
	*Piper umbellatum* L.	Capéba	N	Kidney disorders, diuretic, blood depurative	Leaf, root, bark	[44]
				Diuretic	Leaf, root	[39]
				Diuretic	Root	[48]
				Diuretic, blood depurative, kidney disorders	Leaf, root	[43]
				Diuretic	Leaf, root	[35]
				Diuretic, kidney disorders	Whole plant	[47]
				Diuretic	Root	[33]
				Diuretic	Leaf, root	[36]
				Diuretic	Root, leaf	[40]
Plantaginaceae	*Scoparia dulcis* L.	Vassourinha	N	Blood depurative	Whole plant	[32]
				Blood depurative, hematuria, diuretic	Root, leaf, whole plant	[48]
Poaceae	*Guadua paniculata* Munro	Taquara	N	Diuretic	n.d.	[50]
	*Imperata brasiliensis* Trin.	Capim-sapê	N	Diuretic	Whole plant	[47]
	*Schizachyrium condensatum* (Kunth) Nees	Rabo-de-burro	N	Diuretic	Root	[50]
				Diuretic	Root	[35]
Polygalaceae	*Asemeia acuminata* (Willd.) J.F.B.Pastore and J.R.Abbott		N	Diuretic	Root	[35]
				Diuretic	n.d.	[34]
	*Bredemeyera floribunda* Willd.	Botica-inteira	N	Kidney diseases	Whole plant	[44]
	*Bredemeyera laurifolia* (A.St.-Hil. and Moq.) Klotzsch ex A.W.Benn.	João-da-Costa	N	Kidney disorders	Root bark	[50]
	*Senega lancifolia* (A.St.-Hil. and Moq.) J.F.B.Pastore (synonym of *Polygala lancifolia* A.St.-Hil. & Moq.)	Polígala	N	Diuretic, kidney disorders	Root	[44]
	*Senega paniculata* (L.) J.F.B.Pastore and J.R. Abbott (synonym of *Polygala paniculata* L.)	Barba-de-São-Pedro	N	Diuretic	Root	[35]
				Diuretic	Leaf	[33]
				Diuretic, kidney disorders	Root	[44]
	*Senega timoutou* (Aubl.) J.F.B.Pastore (synonym of *Polygala timoutou* Aubl.)	Timutu	N	Diuretic	Root	[35]
Polygonaceae	*Persicaria hydropiperoides* (Michx.) Small (synonym of *Polygonum hydropiperoides* Michx.)	Acataia	N	Diuretic	Leaf	[33]
	*Persicaria punctata* (Elliott) Small (synonym of *Polygonum punctatum* Elliott)	Erva-de-bicho	N	Diuretic	n.d.	[47]
				Diuretic, blood depurative	Whole plant, leaf,	[38]
				Diuretic	Whole plant	[39]
				Diuretic	Whole plant	[44]
Pontederiaceae	*Pontederia crassipes* Mart. (synonym of *Eichhornia crassipes* (Mart.) Solms)	Aguapé	N	Diuretic, blood depurative	Leaf	[44]
Portulacaceae	*Portulaca pilosa* L.	Amor-crescido	N	Kidney colic, diuretic	Leaf	[32]
Primulaceae	*Myrsine coriacea* (Sw.) R.Br. ex Roem. and Schult.	Azeitona-do-mato	N	Diuretic, blood depurative	Fruit	[34]
Pteridaceae	*Adiantum capillus-veneris* L.	Avenca	N	Diuretic	Leaf	[38]
Rhamnaceae	*Ampelozizyphus amazonicus* Ducke	Saracura-mirá	N	Blood depurative	Root	[40]
				Blood depurative	Root	[35]
Rosaceae	*Rubus brasiliensis* Mart.	Amoreira	N	Diuretic	Leaf, root	[50]
				Diuretic	Leaf, root	[46]
				Diuretic	n.d.	[45]
	*Rubus rosifolius* Sm.	Morango-silvestre	E	Diuretic, kidney disorders, blood depurative	Fruit, sprout, root	[44]
	*Rubus sellowii* Cham. and Schltdl.	Amora-brava	N	Diuretic	Root	[33]
Rubiaceae	*Alibertia edulis* (Rich.) A.Rich.	Marmelada-bola	N	Kidney disorders	Leaf	[36]
	*Spermacoce verticillata* L. (synonym of *Borreria verticillata* (L.) G.Mey.)	Vassourinha-de-botão	N	Diuretic	Root	[33]
	*Chiococca alba* (L.) Hitchc.	Cainca	N	Diuretic	Root bark	[33]
				Blood depurative	Root	[36]
				Diuretic, blood depurative	Root	[51]
				Diuretic	Root bark	[35]
				Diuretic	Root	[39]
				Diuretic, blood depurative	Root	[40]
	*Galianthe centranthoides* (Cham. and Schltdl.) E.L.Cabral	Sabugueirinho-do-campo	N	Diuretic	Whole plant	[39]
	*Genipa americana* L.	Jenipapeiro	N	Diuretic	Stem bark	[36]
				Diuretic	Fruit	[51]
				Kidney disorders, diuretic	Leaf, fruit, stem bark, root, seed	[44]
				Diuretic	Fruit	[40]
				Diuretic	n.d.	[35]
				Diuretic	n.d.	[34]
				Diuretic	Fruit, bark	[39]
	*Guettarda viburnoides* Cham. and Schltdl.	Veludo-branco	N	Kidney disorders	Leaf, root, bark	[36]
	*Palicourea coriacea* (Cham.) K.Schum.	Douradinha	N	Kidney disorders	Leaf	[37]
	*Palicourea crocea* (Sw.) Schult.	Roxinha	N	Kidney pain	Whole plant	[50]
	*Palicourea longiflora* DC.	Douradinha	N	Diuretic, blood depurative, kidney infection	Leaf	[36]
	*Palicourea rigida* Kunth	Douradão	N	Kidney infection	Leaf	[36]
				Blood depurative, kidney diseases	Leaf, stem bark	[44]
				Diuretic	Leaf	[37]
				Blood depurative, kidney diseases	Root, stem bark, leaf	[50]
				Diuretic	n.d.	[47]
	*Palicourea tetraphylla* Cham. and Schltdl.	Dom-Bernardo	N	Diuretic, kidney disorders	Leaf	[34]
	*Randia armata* (Sw.) DC. subsp. *armata*	Espinho-de-carneiro	N	Blood depurative	Root	[47]
	*Rudgea viburnoides* (Cham.) Benth.	Congonha-de-bugre	N	Diuretic, blood depurative, kidney infection	Leaf	[36]
				Diuretic, kidney diseases	Leaf	[50]
				Blood depurative	Root, bark	[35]
				Diuretic, kidney diseases	Leaf, root	[44]
				Blood depurative	Bark	[39]
	*Uncaria guianensis* (Aubl.) J.F.Gmel.	Unha-de-gato	N	Blood depurative	n.d.	[33]
Rutaceae	*Ertela trifolia* (L.) Kuntze	Alfavaca-brava	N	Diuretic, kidney disorders	Whole plant	[35]
				Diuretic	Whole plant	[33]
	*Pilocarpus giganteus* Engl.	Jaborandi-de-fruto-grande	N	Diuretic	n.d.	[35]
	*Pilocarpus jaborandi* Holmes	Jaborandi	N	Diuretic, nephritis	n.d.	[40]
	*Pilocarpus microphyllus* Stapf ex Wardlew.	Jaborandi	N	Diuretic, nephritis	n.d.	[40]
	*Pilocarpus pennatifolius* Lem.	Jaborandi	N	Diuretic, nephritis	n.d.	[40]
				Kidney diseases	n.d.	[33]
	*Spiranthera odoratissima* A.St.-Hil.	Manacá	N	Diuretic, blood depurative, kidney infection	Leaf, root	[36]
Salicaceae	*Casearia sylvestris* Sw.	Erva-de-bugre	N	Blood depurative	Leaf, bark	[33]
				Diuretic, blood depurative	Leaf	[38]
				Blood depurative, kidney diseases	Leaf, stem bark	[50]
				Blood depurative	Leaf, root	[40]
				Blood depurative	Leaf	[39]
				Diuretic	Bark, leaf	[46]
				Blood depurative	Leaf, bark, root	[49]
				Blood depurative	Leaf, root	[35]
				Blood depurative	n.d.	[51]
				Blood depurative	Leaf	[44]
	*Salix humboldtiana* Willd.	Sarã	N	Kidney stones	Bark	[51]
Sapindaceae	*Allophylus edulis* (A.St.-Hil. A.Juss and Cambess.) Radlk.	Vacum	N	Blood depurative	n.d.	[45]
	*Cardiospermum halicacabum* L.	Poca	N	Diuretic	Root	[51]
	*Cupania oblongifolia* Mart.	Pau-magro	N	Diuretic	Leaf	[44]
	*Sapindus saponaria* L.	Saboneteira	N	Nephroprotective	Fruit	[51]
	*Serjania erecta* Radlk.	Cinco-folhas	N	Nephroprotective	Leaf, root	[36]
	*Talisia esculenta* (A.St.-Hil. A.Juss and Cambess.) Radlk.	Pitombeira	N	Kidney disorders	Leaf	[37]
Selaginellaceae	*Selaginella erythropus* (Mart.) Spring	Palminha-das-pedras	N	Diuretic	n.d.	[47]
Simaroubaceae	*Quassia amara* L.	Quássia	N	Kidney stones	Bark, root, wood	[35]
	*Simarouba versicolor* A.St.-Hil.	Pé-de-perdiz	N	Blood depurative	Root	[36]
Siparunaceae	*Siparuna guianensis* Aubl.	Negramina	N	Kidney pain	Leaf	[43]
				Diuretic	Leaf, flower	[33]
Smilacaceae	*Smilax brasiliensis* Spreng.	Japecanga	N	Blood depurative	Root	[50]
	*Smilax campestris* Griseb.	Japecanga	N	Blood depurative	Root	[50]
	*Smilax cissoides* M.Martens and Galeotti	Japecanga	N	Blood depurative	Root	[50]
	*Smilax fluminensis* Steud.	Japecanga	N	Kidney diseases, blood depurative, diuretic	Root	[51]
				Diuretic, blood depurative	n.d.	[34]
	*Smilax goyazana* A.DC.	Japecanga	N	Blood depurative, diuretic	Leaf, root, rhizome	[49]
	*Smilax japicanga* Griseb.	Japecanga	N	Diuretic, blood depurative	n.d.	[33]
				Diuretic, blood depurative	n.d.	[34]
				Blood depurative	Root	[48]
				Blood depurative	Root	[35]
				Diuretic, blood depurative	Root	[47]
				Blood depurative	Root	[36]
				Diuretic, blood depurative	Root	[39]
	*Smilax longifolia* Rich.	Salsaparrilha	N	Blood depurative	Root	[48]
				Blood depurative	Root	[35]
	*Smilax quinquenervia* Vell.	Salsaparrilha	N	Diuretic	Root	[39]
				Diuretic, blood depurative	Root	[44]
Solanaceae	*Brunfelsia uniflora* (Pohl) D.Don	Manacá	N	Blood depurative	Root, branch, leaf	[44]
				Blood depurative	Root	[48]
				Diuretic	Root	[35]
				Blood depurative	Root	[47]
				Diuretic	n.d.	[33]
				Diuretic	n.d.	[34]
				Diuretic	Root	[39]
	*Physalis pubescens* L.	Camapu	N	Diuretic	Leaf, fruit	[35]
				Diuretic	Whole plant	[47]
				Diuretic	Leaf, fruit	[51]
	*Solanum alternatopinnatum* Steud.	Jequiri	N	Blood depurative	Leaf	[44]
	*Solanum americanum* Mill.	Maria-preta	N	Blood depurative	Whole plant	[50]
				Diuretic, blood depurative	n.d.	[33]
				Blood depurative	Leaf, root	[36]
				Diuretic	n.d.	[34]
				Diuretic	Whole plant	[39]
	*Solanum cernuum* Vell.	Panaceia	N	Diuretic	Leaf, flower	[50]
				Diuretic, blood depurative	Leaf, root	[44]
				Diuretic, blood depurative	Leaf	[33]
				Diuretic	Root, leaf	[39]
	*Solanum lycocarpum* A.St.-Hil.	Lobeira	N	Kidney disorders	Flower, fruit, root	[44]
				Diuretic, kidney colic	Leaf	[33]
	*Solanum paludosum* Moric.	Jurubeba-roxa	N	Blood depurative	Root, leaf	[48]
	*Solanum paniculatum* L.	Jurubeba	N	Diuretic	Fruit, root	[48]
				Diuretic	Leaf, fruit, root	[35]
				Diuretic	n.d.	[47]
				Diuretic, nephroprotective	Root, flower	[51]
				Diuretic	Leaf, root	[38]
	*Solanum viarum* Dunal	Joá	N	Kidney pain	Fruit	[51]
Talinaceae	*Talinum paniculatum* (Jacq.) Gaertn.	Maria-gondó	N	Diuretic	Root	[33]
				Diuretic	Leaf, root	[44]
Urticaceae	*Cecropia concolor* Willd.	Embaúba	N	Diuretic	n.d.	[41]
	*Cecropia pachystachya* Trécul	Embaúba	N	Diuretic	Leaf	[45]
				Diuretic	Leaf	[50]
				Diuretic, nephroprotective	Leaf, stem, root	[48]
				Diuretic	Leaf	[47]
				Diuretic	Leaf	[33]
				Diuretic, blood depurative, kidney infection	Leaf	[36]
	*Cecropia palmata* Willd.	Torém	N	Diuretic	Spout	[39]
				Diuretic	Leaf	[47]
	*Cecropia peltata* L.	Embaúba	N	Nephroprotective	Leaf	[32]
				Diuretic, nephroprotective	n.d.	[34]
	*Laportea aestuans* (L.) Chew	Cansanção	N	Diuretic	Root	[47]
	*Urera aurantiaca* Wedd.	Urtiga	N	Diuretic, kidney disorders	Whole plant	[36]
				Diuretic	n.d.	[51]
	*Urera baccifera* (L.) Gaudich. ex Wedd.	Urtiga	N	Blood depurative, diuretic	Root	[41]
	*Urera caracasana* (Jacq.) Griseb.	Urtigão	N	Diuretic	Root	[35]
Verbenaceae	*Stachytarpheta cayennensis* (Rich.) Vahl	Gervão-azul	N	Diuretic	Leaf, aerial parts	[33]
				Diuretic	Branch, leaf	[46]
				Diuretic	Leaf	[35]
				Diuretic	n.d.	[40]
	*Stachytarpheta indica* (L.) Vahl	Gervão	N	Blood depurative	Whole plant	[36]
				Kidney disorders	Root	[37]
Viburnaceae	*Sambucus australis* Cham. and Schltdl.	Sabugueiro	N	Diuretic	Flower	[47]
				Diuretic, nephritis, kidney stones	Bark	[33]
				Diuretic	Flower, leaf	[44]
				Diuretic	Flower	[40]
Violaceae	*Anchietea pyrifolia* (Mart.) G.Don	Cipó-suma	N	Blood depurative	Stem, root bark	[39]
				Blood depurative	n.d.	[34]
				Blood depurative	Stem, root	[44]
	*Pombalia calceolaria* (L.) Paula-Souza	Ipepacuanha	N	Blood depurative	Root	[48]
Vitaceae	*Cissus erosa* Rich.	Cipó-de-arraia-liso	N	Diuretic	Root	[51]
Vochysiaceae	*Vochysia rufa* Mart.	Pau-doce	N	Diuretic	Leaf, bark, root	[36]
Winteraceae	*Drimys brasiliensis* Miers	Casca-d’anta	N	Diuretic	Stem bark	[44]

E, exotic to Brazilian flora, N, native to Brazilian flora, n.d., not described.

## Data Availability

No new data were created or analyzed in this study. Data sharing is not applicable to this article.

## Supplementary Materials

The following supporting information can be downloaded at: https://www.mdpi.com/article/10.3390/plants14050648/s1, Table S1: Nephroprotective activities of secondary metabolites found in Brazilian plants evaluated through in vitro assays; Table S2: Nephroprotective activities of secondary metabolites found in native Brazilian plants extracts evaluated through in vivo assays; Figure S1: Molecular structures of secondary metabolites with nephroprotective activity evaluated in vitro and in vivo, shown in Tables S1 and S2 of the supporting information.
