# Supplementary material for: Nephroprotective Plant Species Used in Brazilian Traditional Medicine for Renal Diseases: Ethnomedical, Pharmacological, and Chemical Insights
Source: Plants (Basel). 2025 Feb 20;14(5):648. doi: 10.3390/plants14050648 (PMC11901925; doi:10.3390/plants14050648)
Supplement: Supplementary file 1 [file plants-14-00648-s001.zip › plants-3476960-supplementary.pdf]

Table S1: Nephroprotective activities of secondary metabolites found in Brazilian plants evaluated through *in vitro* assays

Compound	Source(s)	Model	Effective concentrations	Biological activities	Reference
Afzelin (**1**)	*C. langsdorffii, B. forficata, E. hirta, H. bonariensis, H. leucocephala, S. cernuum, S. chilensis*	CaOx crystallization	0.01, 0.1 mg/mL	Antilithic effect	[1]
Aloe-emodin (**2**)	*P. crassipes, S. alata, S. occidentalis, T. crustacea*	TGF-β1-induced fibrosis in HK-2 cells	40 μM	Cytoprotective effects and reduction of fibrosis-related proteins levels by the inhibition of PI3K/Akt/mTOR pathway	[2]
α/β- amyrin (**3**)	*A. edulis, A. cathartica, A. colubrina, B. intermedia, C. halicacabum, C. pachystachya, E. prostrata, E. contortisiliquum, E. hirta, E. thymifolia, H. radicans, L. pinaster, O. monacantha, P. umbellatum, S. dulcis, S. rhombifolia, S. verticillata*	High glucose-induced injury in HK-2 cells	20 μM	Anti-apoptotic and anti-inflammatory effects by the miR-181b-5p/HMGB2 axis	[3]
Apigenin (**4**)	*A. precatorius, A. australe, A. colubrina, B. gardneri, B. acmella, C. halicacabum, C. palmata, C. regium, C. erecta, C. spiralis, C. cujete, D. unguis-cati, E. prostrata, E. bonariensis, E. prostrata, E. precatoria, H. impetiginosus, N. theifera, P. edulis, P. tenellus, P. aduncum, P. umbellatum, P. crassipes, S. dulcis, S. occidentalis, S. paniculatum, S. oleraceus, T. cacao, T. crustacea, T. diffusa, U. aurantiaca, U. baccifera, V. polyanthes, V. megapotamica*	Doxorubicin-induced injury in NRK-52E and MPC-5 cells	20 μM	Cytoprotective and anti-apoptotic effects	[4]
		Doxorubicin-induced injury in HEK-293 cells	12.5 μg/mL	Cytoprotective effect by the regulation of NGAL protein expression	[5]
		TGF-β1-induced fibrosis in NRK-49F cells	5, 10, 20 μM	Antifibrotic effect by AMPK activation and reduction of ERK1/2 phosphorylation	[6]
		Hypoxia-induced injury in HK-2 cells	20 μM	Anti-apoptotic effect by PI3K/Akt mediated mitochondria-dependent apoptosis signaling pathway.	[7]

		Indoxyl sulfate-induced injury in HK-2 cells	10 μM	Reduction of endoplasmic reticulum stress, inhibition of PI3K/Akt pathway	[8]
		High glucose-induced injury in HK-2 cells	100, 200 μM	Cytoprotective, anti-apoptotic and antioxidant effects	[9]
		Inhibition of URAT1 in HEK-293T cells	IC_{50}= 0.64 μM	Inhibitory effect of urate transporters	[10]
		Inhibition of GLUT9 in HEK-293T cells	IC_{50}= 2.63 μM		
		Imipenem-induced injury in rabbit primary proximal tubule cells	10 μM	Inhibition of intracellular accumulation and renal transport of imipenem by the inhibition of OAT1/3	[11]
Astragalin (**5**)	*A. occidentale, G. ulmifolia, P. amarus, P. niruri, S. cernuum*	High glucose and lipids-induced injury in HK-2 cells	20 μM	Regulation of mitochondrial function via AMPK agonism	[12]
Betulinic acid (**6**)	*A. amazonicus, A. colubrina, B. intermedia, C. erosa, D. dentatus, E. prostrata, E. contortisiliquum, E. uniflora, H. impetiginosus, P. rigida, S. dulcis, S. alata*	T-2 toxin-induced injury in PK15 cells	0.25, 0.5, 1 μM	Modulation of oxidative stress and endoplasmic reticulum stress	[13]
		High glucose-induced injury in glomerular mesangial cells	20, 40 μM	Cytoprotective, antiproliferative and antifibrotic effects	[14]
Bixin (**7**)	*B. orellana*	TGF-β1-induced fibrosis in HK-2 cells	40 μM	Inhibition of cell fibrotic markers and related protein expression	[15]
				Suppression of EMT and extracellular matrix production	[16]
Caffeic acid (**8**)	*A. conyzoides, A. edulis, A. comosus, A. fraxinifolium, C. indica, C. halicacabum, C.*	AGE-induced injury in HK-2 cells	2.5, 5, 10, 20 μM	Antifibrotic effect	[17]

	palmata, C. erecta, C. antisyphiliticus, C. ingrata, C. americana, D. unguis-cati, E. viscosa, E. bonariensis, E. crassipes, E. giganteum, E. foetidum, E. hirta, E. precatoria, G. ulmifolia, H. impetiginosus, I. paraguariensis, I. pes-caprae, J. gossypiifolia, L. pinaster, M. elliptica, O. monacantha, P. niruri, P. tenellus, R. viburnoides, S. alata, S. reticulata, S. rhombifolia, S. fluminensis, S. verticillata, S. lycocarpum, S. paniculatum, S. viarum	CaOx crystallization in synthetic urine	0.1, 0.3 mg/mL	Antilithic effect	[18]
Caffeine (**9**)	*I. paraguariensis, T. cacao, T. diffusa*	CaOx crystallization-stimulated MDCK cells	1, 10, 100 μM	Antilithic effect	[19]
		Hypoxia-induced renal fibroblast activation in BHK-21 cells	6.25 mM	Antifibrotic activity	[20]
		NaOx-stimulated EMT in MDCK cells	100 μM	Nephroprotective effect	[21]
Catechin (**10**)	*A. edulis, A. occidentale, A. colubrina, A. comosus, B. argyrophylla, B. laevifolia, B. intermedia, C. pachystachya, C. palmata, C. cajucara, C. heliotropiifolius, C. americana, E. biflora, E. involucrata, E. precatoria, G. ulmifolia, H. tomentosa, L. pisonis, L. ferrea, M. ilicifolia, M. elliptica, O. monacantha, P. alata, P. americana, P. hydropiperoides, P. brasiliensis, P. niruri, P. tenellus, S. humboldtiana, S. molle, S. terebinthifolia, S. dulcis, S. reticulata, S. campestris, S. mombin, S. adstringens, T. esculenta, T. cacao, T. grandiflorum, V. megapotamica, X. americana*	Melamine and cyanuric acid crystallization	100 μM	Antilithic effect	[22]
		CaOx-induced injury in NRK-52E cells	0.4 μL/mL	Cytoprotective effect against mitochondrial membrane potential, antioxidant and caspase cleavage alterations	[23]
		Hypoxia-induced injury in HKC-8 cells	100 μM	Cytoprotective effect by the modulation of TXNRD1 expression	[24]
		Enzymatic activity	IC50 = 5.47 μM		

Chlorogenic acid (**11**)	*A. hispidum, A. edulis, A. brasiliana, A. tenella, A. colubrina, A. comosus, B. intermedia, C. halicacabum, C. halicacabum, C. pachystachya, C. palmata, C. peltata, C. americana, D. unguis-cati, D. brasiliensis, E. viscosa, E. foetidum, E. precatoria, G. ulmifolia, H. impetiginosus, H. tomentosa, I. paraguariensis, I. imperati, I. pes-caprae, L. paniculata, L. pinaster, M. elliptica, O. campechianum, P. americana, P. tenellus, P. crassipes, P. pubescens, R. viburnoides, S. australis, S. alata, S. rhombifolia, S. lycocarpum, S. paniculatum, S. chilensis, S. oleraceus, S. mombin, T. paniculatum, T. esculenta, T. cacao, X. aromatica*			Inhibition of rat kidney aldose reductase activity	[25]
		CaOx-induced injury in HK-2 cells	300, 400, 500 μg/mL	Cell membrane protection and antioxidant activity	[26]
Chrysophanol (1**2**)	*S. occidentalis*	Hypoxia-reoxygenation-induced injury in HK-2 cells	30 μM	Cytoprotective effect by the regulation of apoptosis, endoplasmic reticulum stress, and ferroptosis	[27]
		TGF-β1-induced fibrosis in HK-2 cells	25, 50, 100 μM	Anti-inflammatory and antifibrotic effects by the inhibition of NKD2/NF-κB pathway	[28]
		Cisplatin-induced injury in HK-2 cells	1.25, 5, 20 μM	Cytoprotective, anti-apoptotic and antioxidant effects	[29]
		TGF-β1-induced fibrosis in HK-2 cells	50 μM	Cytoprotective and antifibrotic effects	[30]
		Canine parvovirus-induced injury in MDCK cells	40 μM	Cytoprotective, anti-inflammatory, antioxidant and anti-apoptotic effects	[31]
Cyanidin-3-*O*-glucoside (**13**)	*A. occidentale, E. punicifolia, E. hirta, E. precatoria, P. niruri, P. tenellus, P. peruviana*	Hypoxia-reoxygenation-induced injury in HK-2 and NRK-52E cells	50 μM	Cytoprotective effect against ferroptosis by AMPK activation	[32]
		Hypoxia-induced injury in HK-2 cells	200 μM	Cytoprotective and anti-apoptotic effects	[33]

		Cisplatin-induced injury in HK-2 cells	40 µg/mL	Anti-apoptotic effect by the modulation of ERK and AKT pathways	[34]
		High glucose-induced injury in HK-2 cells	50 µM	Anti-apoptotic effect, suppression of the phosphorylation of p38 MAPK and ERK1/2 oxidase activity	[35]
		High glucose-induced injury in HK-2 cells	50 µM	Blockade of cholesterol deposition and inhibition of the LXRα pathway-induced inflammatory response	[36]
Delphinidin (**14**)	*A. occidentale, E. punicifolia, E. precatoria, P. peruviana, P. crassipes*	Antimycin a, patulin and insulin-induced injury in NRK cells	1, 3.2, 10 µM	Cytoprotective effect, increase in HO-1 expression	[37]
		High glucose-induced injury in CRL-1927 cells	50 µM	Cytoprotective effects and prevention of collagen synthesis by inhibition of NOX-1 and mitochondrial superoxide	[38]
Ellagic acid (**15**)	*A. comosus, C. villosum, C. antisyphiliticus, C. heliotropiifolius, C. ingrata, E. biflora, E. uniflora, G. ulmifolia, L. pisonis, L. ferrea, M. velame, P. amarus, P. niruri, P. peruviana, R. rosifolius, S. mombin, T. diffusa, X. americana*	CaOx-induced injury in HK-2 cells	20 µM	Cytoprotective effect	[39]
		Uric acid-induced injury in SV40MES13 cells	5 µM	Cytoprotective, anti-inflammatory and antifibrotic effects	[40]
		Rat kidney aldose reductase activity	IC50 = 5.7 µg/mL	Inhibition of aldose reductase activity	[41]
Emodin (**16**)	*H. impetiginosus, S. occidentalis*	TGF-β1-induced fibrosis in NRK-52E cells	100 µM	Antifibrotic effect. Regulation of lipid accumulation, and mitochondrial homeostasis by the modulation of the PGC-1α pathway	[42]
		TGF-β1-induced fibrosis in NRK-49F	20 µM	Antifibrotic effect by the inhibition of EZH2, H3K27me3 and fibronectin expressions	[43]

		High glucose-induced injury in MPC-5 cells	4 μM	Anti-apoptotic effect by the modulation of AMPK/ mTOR pathway	[44]
		Chemical hypoxia-induced injury in HK-2 cells	3 μM	Cytoprotective effect by improving mitochondrial dysfunction	[45]
		Chemical hypoxia and vancomycin-induced injury in HK-2 cells	30 μM	Anti-apoptotic by the regulation of p53/Caspase-9/Caspase-3, p53/Bcl-2 and HIF-1α/VEGF signaling pathway	[46]
		IgG-type anti-dsDNA antibody-induced injury in mouse mesangial cells	40 μM	Inhibition of cellular transdifferentiation to a fibroblast-like phenotype. Reduction of TNF-α, ICAM-1 and fibronectin levels	[47]
		Cisplatin-induced injury in HEK-293 cells	0.5 μM	Cytoprotective and antioxidant effects	[48]
		TGF-β1-induced fibrosis in HK-2 cells	20 μM	Antifibrotic effect by the regulation of fibronectin, α-SMA and Smad2 expression	[49]
		TGF-β1-induced EMT in HK-2 cells	100 μM	Attenuation of EMT by the activation of autophagy and BMP-7 protein regulation	[50]
		High glucose-induced injury in mouse podocyte cells	20, 40 μM	Anti-apoptotic effect by the inhibition of PERK-eIF2α signaling pathway	[51]
		High glucose-induced injury in mouse podocytes	30 μM	EMT suppressive effect by the inhibition of ILK and desmin and nephrin upregulation	[52]
		Cisplatin-induced injury in HEK-293 cells	10, 100 μM	Anti-apoptotic effect by the modulation of AMPK/mTOR signaling pathway and activation of autophagy	[53]
		Hypoxia/reoxygenation-induced injury in HK-2 cells	10, 30, 50 μM	Anti-apoptotic effect by the regulation of cellular oxidative stress, MAPK activation and restoration of Bax/Bcl-2 ratio.	[54]

		High glucose-induced injury in human peritoneal mesothelial cells	20 μM	Cytoprotective effect by the reduction of TGF-β1 bioactivity and matrix synthesis, inhibition of PKCα activation and CREB phosphorylation	[55]
		High glucose-induced injury in HBZY-1 cells	30, 60 μM	Suppression of cell proliferation and fibronectin expression by the inhibition of p38MAPK signaling pathway	[56]
		High glucose-induced injury in rat mesangial cells	20, 40 μM	Cytoprotective effect by the suppression of TGF-β1 and fibronectin overexpression through inhibition of NF-κB activation	[57]
Epicatechin (**17**)	*A. cathartica, A. occidentale, A. comosus, A. spinescens, B. laevifolia, B. intermedia, C. pachystachya, C. cajucara, D. rugosa, E. involucrata, E. precatoria, G. ulmifolia, H. courbaril, L. pisonis, M. ilicifolia, P. alata, P. americana, P. hydropiperoides, P. brasiliensis, S. reticulata, S. mombin, S. adstringens, T. esculenta, T. esculenta, T. cacao, T. grandiflorum, V. megapotamica*	Hypoxia-induced injury in HKC-8 cells	100 μM	Cytoprotective effect by the maintenance of TXNRD1 expression	[24]
		Cisplatin-induced injury in mouse proximal tubular cells	1 μM	Cytoprotective effect, reduction of ERK activity	[58]
		NRK-52E cells	5, 10 μM	Regulation of the renal glucose homeostasis by the modulation of both glucose uptake and production	[59]
Epicatechin gallate (**18**)	*E. hirta, L. pisonis, P. niruri*	SIN1-induced injury in LLC-PK1 cells	25, 125 μM	Anti-apoptotic effect, regulation of the cell cycle by enhancing G2/M phase arrest	[60]
		Ochratoxin A-induced injury in LLC-PK1 cells	60 μM	Cytoprotective, antioxidant effects and reduction of DNA fragmentation	[61]
Epigallocatechin-3-gallate (**19**)	*M. ilicifolia, P. niruri, S. adstringens*	Ochratoxin A-induced injury in LLC-PK1 cells	30 μM	Cytoprotective, antioxidant effects and reduction of DNA fragmentation	[61]

		AGE-induced injury in HEK-293 and human mesangial cells	10 μM	Cytoprotective and anti-apoptotic effects	[62]
		Cisplatin-induced injury in HK-2 cells	10 μM	Anti-apoptotic effect and reduction of the mitochondrial reactive oxygen species generation	[63]
		H_2O_2-induced injury in HEK-293 cells	40, 80 μM	Cytoprotective and anti-apoptotic effects	[64]
		TGF-β1-induced fibrosis in NRK-52E cells	2, 5 μM	Anti-apoptotic effect by the MAPK phosphorylation inhibition	[65]
Ferulic acid (**20**)	*A. edulis, A. brasiliana, A. tenella, A. colubrina, A. comosus, C. indica, C. halicacabum, C. palmata, C. antisyphiliticus, C. ingrata, D. unguis-cati, E. giganteum, E. foetidum, E. hirta, E. precatoria, G. ulmifolia, L. pisonis, M. elliptica, O. monacantha, P. americana, P. aduncum, P. crassipes, S. alata, S. reticulata, S. rhombifolia, S. alternatopinnatum, S. viarum, T. esculenta, T. cacao, T. crustacea, V. megapotamica*	High glucose-induced injury in NRK-52E cells	75 μM	Cytoprotective effect. Blockade of autophagy	[66]
		LPS-induced injury in NRK-52E cells	75 μM	Anti-apoptotic and anti-inflammatory effects by the upregulation of AMPKα1 expression and phosphorylation	[67]
		TGF-β1-induced fibrosis in NRK-52E cells	25, 50, 100, 200 μM	Antifibrotic effect. Suppression of EMT process	[68]
		Cisplatin-induced injury in HK-2 cells	1, 10, 50 μM	Cytoprotective effect by the reduction of ECM deposition and anti-apoptotic effect	[69]
Fisetin (**21**)	*B. pentandra, H. courbaril, M. peruiferum*	Adenine and TGF-β1-induced fibrosis in TCMK-1 cells	20 μM	Anti-inflammatory and antifibrotic effects. Suppression of ferroptosis	[70]
		Uric acid-induced injury in TCMK-1 cells	5, 10, 20 μM	Cytoprotective, anti-inflammatory and antifibrotic effects by the modulation of the TGF-β/Smad3 signaling pathway	[71]
		High glucose-induced injury in mouse podocytes	5, 25, 50 μM	Cytoprotective effect by the restoration of autophagy-mediated CDKN1B/P70S6K pathway and inhibition of NLRP3 inflammasome	[72]

		TGF-β1-induced fibrosis in human proximal tubular cells	40 μM	Antifibrotic effect by the inhibition of TGF-β1–induced phosphorylation of SMAD3 and SMAD2	[73]
		TGF-β1-induced fibrosis in NRK-52E and NRK-49F	5, 10, 20 μM	Anti-senescent and antiproliferative effects	[74]
		Palmitate-induced injury in HK-2 cells	20, 40 μM	Cytoprotective and anti-inflammatory effects by the suppression of RIP3 expression and NLRP3 inflammasome	[75]
Formononetin (**22**)	*A. precatorius, B. nitida, M. balsamum, S. paniculatum*	Ferroptosis-induced injury in primary mouse renal tubular epithelial cells	20, 40, 80 μM	Inhibition of ferroptosis by the suppression of Smad3 and ATF3 translocation from cytoplasm to nucleus	[76]
		Cisplatin-induced injury in HK-2 cells	10 μM	Anti-apoptotic and cytoprotective effects by the inhibition of OCT2 and increasing Mrp2 and Mrp4 functions	[77]
		High glucose-induced injury in HK-2 cells	10, 20 μM	Anti-apoptotic effect and alleviation of mitochondrial membrane potential loss by the regulation of Sirt1/PGC-1α pathway	[78]
		Cisplatin-induced injury in LLC-PK1 cells	10, 25 μM	Cytoprotective effect by the inhibition of intracellular ROS accumulation	[79]
Gallic acid (**23**)	*A. edulis, A. cathartica, A. occidentale, A. comosus, B. intermedia, B. verbascifolia, C. halicacabum, C. villosum, C. regium, C. antisyphiliticus, C. heliotropiifolius, C. ingrata, C. americana, D. rugosa, E. involucrata, E. punicifolia, E. uniflora, E. hirta, E. prostrata, G. ulmifolia, J. gossypiifolia, J. princeps, L. pisonis, L.*	CaOx crystal-induced cell deaths and crystal adhesion in HK-2 cells	100 μM	Decrease of crystal deposition and of renal oxidative and inflammatory injuries	[80]
		H_2O_2-induced injury in HEK-293 cells	25 μM	Cytoprotective effect	[81]
		Glyoxal-induced injury in rat kidney proximal tubular cells	10 μM	Cytoprotective effect	[82]

	ferrea, L. paniculata, M. elliptica, O. monacantha, P. americana, P. hydropiperoides, P. amarus, P. niruri, P. sellowianus, P. tenellus, P. peruviana, P. crassipes, P. pilosa, P. guineense, R. rosifolius, S. molle, S. terebinthifolia, S. alata, S. reticulata, S. viarum, S. verticillata, S. mombin, S. adstringens, T. esculenta, T. cacao	CaOx crystallization	0.003, 0.01, 0.03 mg/mL	Antilithic effect	[83]
		High glucose-induced injury in rat NRK 52E cells	10 μM	Inhibition of the p38 MAPK and NF-κB activation and of proinflammatory cytokine synthesis	[84]
Gentisic acid (**24**)	*G. ulmifolia, P. crassipes*	CaOx crystallization	1.5 mM	Antilithic effect	[85]
Hederagenin (**25**)	*A. edulis, E. serpens, P. mediterranea, P. emarginatus, S. saponaria*	TGF-β1- induced fibrosis in TCMK1 cells	10, 20, 30 μg/mL	Antifibrotic effects by inhibiting the JAK/STAT signaling pathway	[86]
		LPS-induced injury in pTEC cells	10, 20, 30 μg/mL	Cytoprotective and anti-inflammatory effects by the inhibition of Axin2/β-catenin signaling pathway	[87]
		TGF-β1-induced fibrosis in NRK-49F cells	5, 10, 15 μM	Antifibrotic effect by targeting muscarinic acetylcholine receptor	[88]
Hispidulin (**26**)	*A. precatorius, S. dulcis, S. rhombifolia*	High glucose-induced injury in MPC-5 cells	2, 5 μM	Anti-apoptotic effect and induction of autophagy by the regulation of Pim1-p21-mTOR signaling axis	[89]
Hyperoside (**27**)	*A. occidentale, A. colubrina, C. racemosa, G. ulmifolia, H. bonariensis, H. leucocephala, P. americana, S. australis, T. cacao*	Albumin-induced injury in HK-2 cells	10 μM	Cytoprotective effect and alleviation of proteinuria-induced pyroptosis	[90]
		Cadmium-induced injury in RPTEC cells	25, 50 μM	Cytoprotective effect by the inhibition of MAPK/NF-κB pathway stimulation	[91]
		Oxalic acid induced injury in- HK-2 cells	50, 100, 200 μM	Anthilithiac and cytoprotective effect by the regulation of Nrf2/HO-1/NQO1 pathway	[92]

		High glucose-induced injury in mouse podocytes	2 mmol/L	Suppression of extracellular matrix accumulation, inflammation, and apoptosis via the miR-499–5p/APC axis	[93]
		High glucose-induced injury in HK-2 cells	50 μM	Anti-inflammatory and anti-apoptotic effects by the modulation of the miR-499a-5p/NRIP1 axis	[94]
		D-galactose-induced injury in NRK-52E cells	5, 10 μg/mL	Cytoprotective and anti-apoptotic effects	[95]
		High glucose-induced injury in mouse mesangial cells	100 μM	Cytoprotective effect by the upregulation of MMP-9 expression	[96]
		High glucose-induced injury in immortalized mouse podocytes	30, 100 μg/ml	Cytoprotective effect by decreasing the heparanase expression induced by ROS	[97]
		Chemical hypoxia-induced injury in HK-2 cells		Inhibition of mitochondrial fission, anti-apoptotic and antioxidant effects	[98]
		CaOx crystallization-induced injury in HK-2 cells	100 μM	Cytoprotective, antilithic and anti-inflammatory effects by the modulation of the AMPK/Nrf2 signaling pathway	[99]
		Cisplatin-induced injury in HK-2 cells	10, 25, 50 μM	Cytoprotective effect by the activation of Nrf2 signaling pathways	[100]
		AGE-induced injury in immortalized murine podocytes	50, 200 μg/mL	Cytoprotective and anti-apoptotic effects by the inhibition of caspases expression	[101]
Hypophyllanthin (**28**)	*E. hirta, P. amarus, P. niruri, P. tenellus*	Enzymatic activity	$IC_{50} > 40$ μg/mL	Inhibition of xanthine oxidase activity	[102]
Isorhamnetin (**29**)	*A. colubrina, A. coriacea, B. forficata, C. sylvestris, E. biflora, E. hirta, G. integrifolia, G. ulmifolia, H. leucocephala, J.*	Cisplatin-induced injury in HK-2 cells	2.5 μM	Anti-apoptotic and anti-inflammatory effects by PGC-1α mediated fatty acid oxidation	[103]

	caroba, O. monacantha, P. rigida, P. hydropiperoides, S. molle, S. oleraceus, S. pseudoquina, T. cacao, V. polyanthes	LPS-induced injury in rat glomerular mesangial cells	5, 10 μM	Antioxidant and anti-inflammatory effects. Inhibition of the NF-κB signaling activity	[104]
Isovitexin (**30**)	*A. grandiflorus, C. tayuya, C. erosa, C. leptophloeos, C. cajucara, E. precatoria, I. imperati, J. princeps, L. ferrea, L. pinaster, N. theifera, P. alata, P. edulis, P. pelucida, P. crassipes, S. erecta, S. alternatopinnatum, T. cacao*	LPS-induced injury in SV40-MES 13 cells	2.5, 5 μM	Cytoprotective effect by the modulation of the mitochondrial membrane potential, downregulation of inflammation and pyroptosis factors	[105]
Kaempferitrin (**31**)	*B. forficata*	AGE-induced injury in glomerular mesangial cells	10, 20, 35 μM	Anti-apoptotic, antifibrotic and antioxidant effects	[106]
Kaempferol (**32**)	*A. capillus-veneris, A. conyzoides, A. cathartica, A. brasiliana, A. tenella, A. occidentale, A. coriacea, A. spinescens, B. argyrophylla, B. forficata, C. halicacabum, C. sylvestris, C. pareira, C. sympodialis, C. gongonha, C. cajucara, C. ingrata, C. americana, C. racemosa, D. rugosa, D. dentatus, E. viscosa, E. giganteum, E. foetidum, E. uniflora, E. hirta, E. thymifolia, E. precatoria, G. integrifolia, G. ulmifolia, H. impetiginosus, H. crispa, H. balsamifera, H. bonariensis, I. paraguariensis, I. imperati, J. caroba, L. pisonis, M. acutifolium, M. ilicifolia, O. monacantha, P. amarus, P. brasiliensis, P. niruri, P. tenellus, P. crassipes, P. guineense, R. rosifolius, S. australis, S. terebinthifolia, S. dulcis, S. alata, S. erecta,*	TGF-β1-induced EMT in NRK-52E cells	20 μM	Cytoprotective effect	[107]
		Doxorubicin- induced injury in NRK-52E cells	100 μM	Cytoprotective and antioxidant effects. Inhibition of MAPK signaling pathway	[108]
		TGF-β1-induced fibrosis in HK-2 cells	10, 20, 40 μM	Antifibrotic effect. Inhibition of EMT process	[109]
		CaOx crystal-induced cell deaths and crystal adhesion in HK-2 cells	10, 20, 40 μM	Decrease of crystal deposition and of renal oxidative and inflammatory injuries	[110]
		D-ribose-induced injury in SV40-MES 13 cells	1, 2, 5 μM	Anti-apoptotic and anti-autophagic effects	[111]
		High glucose-induced injury in NRK-52E and RPTEC cells	10, 50 μM	Antifibrotic, antioxidant, anti-inflammatory effects	[112]
		Cisplatin-induced injury in HEK-293 cells	25 μM	Anti-apoptotic and antioxidant effects	[113]

	S. rhombifolia, S. versicolor, S. guianensis, S. brasiliensis, S. cernuum, S. lycocarpum, S. paludosum, S. paniculatum, S. oleraceus, S. mombin, S. adstringens, T. aurea, T. paniculatum, T. cacao, T. grandiflorum, U. lobata, V. ferruginea, V. megapotamica, X. americana	High glucose-induced injury in MPC-5 cells	4, 8 μM	Anti-apoptotic and anti-inflammatory effects	[114]
Liquiritigenin (**33**)	*B. pentandra, B. acutifolium*	Erastin-induced ferroptosis in HK-2 cells	25 μM	Cytoprotective and antiferroptosis effects by the upregulation of VKORC1	[115]
		Cisplatin-induced injury in HK-2 cells	25 μM	Anti-apoptotic effect and improvement of mitochondrial function by the modulation of the Nrf2 activity	[116]
		High glucose-induced injury in HZBY-1 cells	20, 40 μM	Anti-inflammatory, antioxidant and reduction in ECM accumulation by the suppression of the NF-κB and NLRP3 inflammasome pathways	[117]
Luteolin (**34**)	*A. occidentale, A. pyrifolia, B. gardneri, B. acutifolium, B. gaudichaudii, C. halicacabum, C. palmata, C. erosa, C. regium, C. erecta, C. cujete, C. antisyphiliticus, D. unguis-cati, E. prostrata, E. bonariensis, E. foetidum, E. involucrata, E. prostrata, E. precatoria, G. ulmifolia, H. impetiginosus, J. decurrens, L. paniculata, M. velame, N. theifera, O. campechianum, P. edulis, P. brasiliensis, P.*	H_2O_2-induced injury in 293T cells	5, 10, 20 μM	Suppression of NF-κB and HIF-1α transcriptional activity	[118]
		Methamphetamine-induced injury in mouse podocytes	50 μM	Cytoprotective effect, inhibition of podocyte protein loss	[119]
		High glucose-induced injury in MPC-5 cells	25, 50, 100 μM	Anti-apoptotic and anti-inflammatory effect by reducing the NLRP3 inflammasome formation	[120]

	niruri, P. stratiotes, P. crassipes, P. emarginatus, S. dulcis, S. fluminensis, S. oleraceus, T. aurea, T. paniculatum, T. esculenta, T. cacao, T. crustacea, T. diffusa, U. baccifera, V. ferruginea, V. polyanthes, X. aromatica	Ochratoxin A-induced injury in NRK-52E cells	50, 100, 200 μM	Cytoprotective effect by the activation of Nrf2 pathway and modulation of HIF-1α and VEGF pathways	[121]
Madecassoside (**35**)	*C. asiatica*	Doxorubicin-induced injury in HK-2 cells	5, 10, 30, 60, 100 μM	Anti-apoptotic and anti-inflammatory effects	[122]
Magnoflorine (**36**)	*C. glaberrima, C. pareira*	High glucose-induced injury in SV40-MES 13 cells	100 μg/mL	Reduction of abnormal proliferation, antifibrotic and anti-inflammatory effects by the inhibition of TGF-β1/Smad2/3 signaling pathway	[123]
		Peroxynitrite-induced injury in LLC-PK1 cells	10, 25 μg/mL	Cytoprotective effect by the control of cell cycle and DNA damage	[124]
Morin (**37**)	*A. colubrina, M. velame, S. dulcis*	H_2O_2-induced injury in MDCK cells	20, 40, 60, 80, 100 μM	Cytoprotective, antioxidant and anti-apoptotic effects	[125]
		Tunicamycin-induced injury in HK-2 cells	10, 50, 100 μM	Reduction of oxidative stress and anti-apoptotic effect	[126]
		High glucose-induced injury in glomerular mesangial cells	25, 50 μM	Antiproliferative and inhibition of ECM expression by the suppression of p38 MAPK and JNK signaling pathways.	[127]
		Cisplatin-induced injury in HEK-293 cells	10, 20 μM	Cytoprotective, anti-inflammatory, and anti-autophagic effects by the regulation of PARP-1	[128]
		Imipenem-induced injury in MDCK cells	100 μM	Cytoprotective effect by the inhibition of OAT3-mediated cellular uptake	[129]
		CaOx crystallization	200 μg/mL	Antilithic effect by the alteration of crystal morphology to unstable forms	[130]

		Xanthine oxidase/hypoxanthine and menadione-induced injury in rat glomerular mesangial cells	0.5, 1 mM	Cytoprotective and antioxidant effects	[131]
Myricetin (**38**)	*A. occidentale, A. colubrina, B. forficata, C. regium, D. rugosa, E. involucrata, E. punicifolia, E. uniflora, J. princeps, L. pisonis, M. velame, P. mediterranea, P. hydropiperoides, P. niruri, P. tenellus, P. crassipes, P. emarginatus, S. paludosum, S. adstringens, T. esculenta*	Cisplatin-induced injury in HK-2 cells	2.5 μM	Antiproliferative effect, reduction of the mitochondrial membrane potential and DNA damage	[132]
		High glucose-induced injury in HRM cells	5, 10, 15 μM	Antiproliferative and antifibrotic effects by the modulation of the ROCK1/ERK/P38 pathway	[133]
Naringenin (**39**)	*A. precatorius, A. cathartica, A. colubrina, B. pentandra, B. acutifolium, C. guianensis, C. cujete, C. antisyphiliticus, E. bonariensis, E. involucrata, G. ulmifolia, H. impetiginosus, L. pisonis, M. velame, P. niruri, P. crassipes, S. humboldtiana, S. terebinthifolia, S. adstringens, T. esculenta, T. cacao, V. megapotamica*	Chemical hypoxia/reperfusion - induced injury in HK-2 cells	200 μM	Inhibition of pyroptosis and apoptosis by activating Nrf2/HO-1 signaling pathway	[134]
		High glucose-induced injury in mouse glomerular mesangial cells	600, 1000 μmol/L	Attenuation in the extracellular matrix protein deposition. Regulation of let-7a/TGFBR1 signaling.	[135]
		Uric acid-induced disordered expression of urate transporters in HK-2 cells	40 μM	Anti-inflammatory effect. Reduction of the expression of GLUT9 by the inhibition of PI3K/AKT signaling pathway	[136]
		High glucose-induced injury in NRK-52E cells	25 μM	Cytoprotective and anti-apoptotic effects	[137]
Niranthin (**40**)	*E. hirta, P. amarus, P. niruri, P. tenellus*	Enzymatic activity	IC50 > 40 μg/mL	Inhibition of xanthine oxidase activity	[102]
Orientin (**41**)	*A. fraxinifolium, C. pachystachya, C. erosa, C. leptophloeos, E. precatoria, J. princeps, L. ferrea, N. theifera, P. alata, P. quadrangularis, P. aduncum, P. stratiotes, P. crassipes, T. cacao, V. polygama*	High glucose-induced injury in MPC-5 cells	120 μM	Anti-apoptosis and autophagy effects by protecting mitochondria	[138]

Phyllanthin (**42**)	*E. hirta, P. amarus, P. niruri*	Enzymatic activity	IC50 > 40 μg/mL	Inhibition of xanthine oxidase activity	[102]
Phyltetralin (**43**)	*E. hirta, P. amarus, P. niruri, P. tenellus*	Enzymatic activity	IC50 > 40 μg/mL	Inhibition of xanthine oxidase activity	[102]
Pinocembrin (**44**)	*C. cujete, E. hirta, P. aduncum, S. leucanthum, T. diffusa*	LPS-induced injury in HK-2 cells	50, 100, 200 μg/mL	Anti-inflammatory, anti-apoptotic and antioxidant effects	[139]
Procyanidin B2 (**45**)	*C. pachystachya, G. ulmifolia*	High glucose-induced injury in SV40-Mes13 cells	10, 25 μM	Inhibitory effect on cell proliferation, oxidative stress, ECM accumulation and cellular inflammation by CAV-1 regulation	[140]
		High glucose-induced injury in HK-2 cells	10 μM	Suppression of EMT by the inhibition of TGF-β1/Smad and MAPK/P38 signaling pathways	[141]
		High glucosamine-induced injury in rat mesangial cells	10 μg/mL	Inhibition of mitochondrial dysfunction and anti-apoptotic effects by the activation of the AMPK–SIRT1–PGC-1α axis	[142]
		High glucose-induced injury in MPC-5 cells	10 μg/mL	Inhibition of mitochondrial dysfunction and anti-apoptotic effects by the activation of the AMPK–SIRT1–PGC-1α axis	[143]
Protocatechuic acid (**46**)	*A. cathartica, C. halicacabum, C. ingrata, E. precatoria, G. ulmifolia, L. pisonis, O. monacantha, P. niruri, S. reticulata, S. adstringens, V. polyanthes*	High glucose-induced injury in human mesangial cells	5, 10 μM	Antiproliferative effect, reduction on extracellular matrix accumulation and inhibition of the p38 MAPK signaling pathway	[144]
Psoralen (**47**)	*B. gaudichaudii, D. brasiliensis, D. cayapia* subsp. *asaroide*	High glucose-induced in injury in HK-2 cells	10 μM	Anti-inflammatory effect and reduction of ECM accumulation by the upregulation of miR-874	[145]
Quercetin (**48**)	*A. hispidum, A. capillus-veneris, A. conyzoides, A. edulis, A. cathartica, A. occidentale, A. colubrina, A. pyrifolia, A. coriacea, A. fraxinifolium, B. argyrophylla, B. laevifolia, B. forficata, B. holophylla, B. gaudichaudii, B. intermedia, B.*	H_2O_2-induced injury in HEK-293 cells	165.4 μM	Cytoprotective effect	[81]
		High glucose-induced injury in HK-2 cells	25 μM	Inhibition of the ferroptosis via the Nrf2/HO-1 signaling pathway regulation	[146]

	verbascifolia, C. halicacabum, C. sylvestris, C. palmata, C. nutans, C. pareira, C. sympodialis, C. erecta, C. leptophloeos, C. antisyphiliticus, C. cajucara, C. heliotropiifolius, C. carthagenensis, C. ingrata, C.americana, C. racemosa, D. rugosa, D. unguis-cati, D. brasiliensis, E. prostrata, E. giganteum, E. bonariensis, E. foetidum, E. involucrate, E. uniflora, E. hirta, E. thymifolia, E. precatoria, G. integrifolia, G. ulmifolia, G. viburnoides, H. impetiginosus, H. balsamifera, H. leucocephala, I. paraguariensis, I. suffruticosa, I. imperati, I. pes-caprae, J. caroba, J. decurrens, J. princeps, L. pisonis, L. pinaster, M. acutifolium, M. velame, M. elliptica, O. monacantha, P. rigida, P. edulis, P. mediterranea, P. americana, P. hydropiperoides, P. amarus, P. brasiliensis, P. niruri, P. sellowianus, P. tenellus, P. pubescens, P. peruviana, P. crassipes, P. guineense, R. rosifolius, R. viburnoides, S. humboldtiana, S. australis, S. molle, S. dulcis, S. occidentalis, S. rhombifolia, S. guianensis, S.brasiliensis, S. campestris, S. fluminensis, S. paniculatum, S. viarum, S. chilensis, S. verticillata, S. mombin, S. pseudoquina, S. adstringens, T. esculenta, T. cacao, T. grandiflorum, T. rhomboidea, T. diffusa, V. ferruginea, V. polyanthes, V. megapotamica, X. aromatica	AGE- injury in HK-2 cells overexpressing SARS-CoV-2 N protein	64 μM	Blockade of SARS-CoV-2 N-Smad3-mediated cell death pathway	[147]
		TGF-β1-induced fibrosis in HK-2 cells	20 μM	Antifibrotic effects by the inhibition of AREG/EGFR signaling. EMT suppressive effect.	[148]
		Iohexol-induced injury in HK-2 cells	20 μM	Anti-inflammatory and anti-apoptotic effects by the attenuation of HIF-1α expression	[149]
		High glucose-induced injury in SV40MES13 cells	5, 10, 50 μg/mL	Anti-inflammatory, antioxidant and antifibrotic effects	[150]
		TGF-β1-induced fibrosis in NRK-49F cells	10, 20 μM	Cytoprotective effects by the inhibition of α-SMA and fibronectin expression, and mTOR and β-catenin signaling modulation	[151]
		TGF-β1-induced EMT in NRK-52E cells	10, 20, 40 μM	Attenuation of EMT and of ECM deposition	[152]
		High glucose-induced injury in HEK-293 cells	3, 6, 12 μM	Reduction of methylglyoxal levels and of SMP30 expression	[153]
		LPS-induced injury in HK-2 cells	20 μM	Anti-inflammatory and anti-apoptotic effects	[154]
		High glucose-induced injury in SV40MES13	20, 40 μM	Antiproliferative effect by the modulation of Hippo pathway	[155]
		Radiocontrast medium-induced injury in HK-2 cells	100 μM	Cytoprotective and antioxidant effects	[156]
		TGF-β1-induced fibrosis in HK-2 cells	20 μM	Anti-apoptotic effect and attenuation of EMT	[157]
		Zearalenone-induced injury in PK15 cells	30 μM	Anti-apoptotic and anti-necrotic effects	[158]

		Chemical anoxia-induced injury in LLC-PK1 cells	10, 30 μM	Cytoprotective effect by the upregulation of AMPK phosphorylation, downregulation of mTOR phosphorylation and autophagy activation	[159]
		Ischemia/reperfusion-induced damage in LLC-MK2 cells	100, 200 μM	Cytoprotective and antioxidant effects. Reduction of KIM-1 levels	[160]
		High glucose-induced injury in HRM cells	40 μM	Cytoprotective effect by the suppression of NF-κB and MCP-1 expression	[161]
		High glucose-induced injury in HK-2 and NRK-52E cells	10, 20, 40 μM	Attenuation of EMT. Inhibition of the mTOR/p70S6 kinase activation	[162]
		High glucose-induced injury in rat mesangial cells	100 μg/mL	Antiproliferative and anti-autophagic effects. Reduction of the TGF-β1 expression	[163]
		Zearalenone-induced injury in HEK-293 cells	5 μM	Anti-apoptotic and antioxidant effects	[164]
		Valproic acid-induced injury in rat kidney post nuclear supernatant	0.05 mM	Antioxidant effect	[165]
		Cadmium-induced injury in RPTEC cells	1.0 μM	Cytoprotective and anti-apoptotic effects	[166]
		High glucose-induced injury in rat mesangial cells	5, 10, 20 μg/mL	Inhibition of cell hypertrophy and of ECM accumulation	[167]
		Hypoxanthine/xanthine oxidase system, H_2O_2, 1-chloro-2,4- dinitrobenzene and aminotriazole- induced damage in LLC-PK1 cells	100 mM	Cytoprotective and antioxidant effects	[168]
		H_2O_2-induced injury in rat mesangial, LLC-PK1, and NRK-49F cells	20, 50 μM	Anti-apoptotic effect by the modulation of c-Jun/AP-1 pathway	[169]

		NaOx-induced injury in MDCK cells	100 μM	Decrease of crystal deposit formation and antioxidant effects	[170]
		Cisplatin-induced LLC-PK1 cells	100, 200, 500 μM	Cytoprotective and antioxidant effects	[171]
		IL1β-induced injury in SM43 rat mesangial cells	50 μM	Suppression of NF-κB and AP-1 activity, and MCP-1 expression	[172]
		High glucose-induced injury in HK2 cells	10, 25, 50, 100 μM	Attenuation of EMT by the inhibition of NOX/ROS/ ERK pathway	[173]
Rosmarinic acid (**49**)	*A. capillus-veneris, C. indica, C. nutans, D. unguis-cati, J. gossypiifolia, L. paniculata, O. campechianum, T. rhomboidea*	CaOx crystallization in synthetic urine	0.3 mg/mL	Antilithic effect	[18]
		Cadmium-induced injury in mouse proximal tubular epithelial cells	40 μM	Antioxidant and antifibrotic effect by the modulation of TGF-β/SMAD/collagen VI signaling pathway	[174]
		High glucose-induced injury in HK-2 cells	4, 8 μM	Antifibrotic effect by the modulation of ERK signaling pathway	[175]
		PDGF- and TNF-α-induced murine mesangial cells	1, 5 μg/mL	Antiproliferative effect by the suppression of PDGF and c-myc mRNA expression	[176]
		Indoxyl sulfate-induced fibrosis in NRK-52E cells	20, 40 μM	Antifibrotic effect by inhibiting the NLRP3 inflammasome signaling pathway	[177]
Rutin (**50**)	*A. edulis, A. cathartica, A. brasiliana, A. tenella, A. colubrina, A. coriacea, B. laevifolia, B. floribunda, B. floribunda, C. sylvestris, C. pachystachya, C. palmata, C. erosa, C. erecta, C. antisyphiliticus, C. cajucara, C. carthagenensis, C. americana, D. rugosa, D. unguis-cati, E. viscosa, E. bonariensis, E. involucrata, E. uniflora, E.*	High glucose-induced injury in glomerular endothelial cells	12.5 μM	Cytoprotective effect by the modulation of PI3K/AKT/mTOR-HDAC1 signaling pathway	[178]
		Vancomycin-induced injury in LLC-PK1 cells	5, 10, 20 μM	Cytoprotective and anti-apoptotic effects	[179]
		Hyperglycemia-induced barrier dysfunction in HRGE cells.	25 μM	Nephroprotective effect	[180]

	hirta, G. integrifolia, G. ulmifolia, H. tomentosa, H. bonariensis, H. leucocephala, I. paraguariensis, I. suffruticosa, I. imperati, J. decurrens, L. paniculata, L. pinaster, M. velame, M. elliptica, O. campechianum, P. alata, P. amarus, P. niruri, P. sellowianus, P. tenellus, P. aduncum, P. peruviana, P. crassipes, P. guineense, S. australis, S. dulcis, S. paniculata, S. rhombifolia, S. guianensis, S. brasiliensis, S. campestris, S. paniculatum, S. viarum, S. chilensis, S. oleraceus, S. mombin, S. adstringens, T. aurea, T. esculenta, T. cacao, X. americana, X. aromatica	Cisplatin-induced injury in HRM cells	12.5, 25 μM	Cytoprotective and anti-apoptotic effects	[181]
Scopoletin (**51**)	*B. uniflora, H. brasiliense, P. rigida, P. americana, R. rosifolius, S. rhombifolia*	High glucose-induced injury in rat glomerular mesangial cells	0.1, 1 μM	Inhibition of cell proliferation, hypertrophy and ECM proliferation. Reduction in the expression of fibronectin, TGF-β1, and connective tissue growth factor	[182]
		High glucose-induced injury in HK-2 cells	5, 10, 20 μg/mL	Anti-apoptotic effect. Reduction in EMT transition, deposition of extracellular matrix components and TGF-β levels	[183]
Scutellarein (**52**)	*A. conyzoides, S. dulcis*	Ischemia/reperfusion-induced damage in HK-2 cells	2.5, 5, 10 μM	Cytoprotective, anti-inflammatory and anti-apoptotic effects	[184]
Taxifolin (**53**)	*D. brasiliensis, E. precatoria, H. tomentosa, L. pisonis*	High glucose-induced injury in HBZY-1 and HK-2cells	20 μM	Antiproliferative and anti-fibrotic effects, suppression of the NLRP3 inflammasome	[185]
		High glucose and salt-induced damage in NRK-52E	10 mM	Anti-apoptotic and anti-inflammatory effects by modulating PI3K/AKT signaling pathway	[186]

		TGF-β1- induced fibrosis in HK-2 cells	80 μM	Cytoprotective effect by preventing the TGF-β1-induced fibroblast activation and collagen synthesis	[187]
Theobromine (**54**)	*I. paraguariensis, T. cacao*	Dissolution of uric acid renal calculi in hermetic flow capsules	40 mg/L	Antilithic effect by inhibiting the formation of new stones and the increase of stones dissolution	[188]
Ursolic acid (**55**)	*A. edulis, A. cathartica, E. prostrata, E. contortisiliquum, G. viburnoides, H. radicans, J. decurrens, P. coriacea, P. amarus, P. guineense, S. rhombifolia, S. verticillata*	High glucose-induced injury in SV40MES13 cells	2.5 μM	Anti-inflammatory and antifibrotic effects	[189]
		CaOx crystals-induced injury in HK-2 cells	2.5, 5 μM	Anti-inflammatory and anti-apoptotic effects	[190]
		TGF-β1-induced fibrosis in HK-2 cells	10, 50 μM	EMT suppressive effect decreasing profibrotic factors	[191]
		High glucose-induced injury in immortalized murine podocytes cells	5 μM	Increase of autophagy by the modulation of PI3K/Akt/mTOR pathway	[192]
		High glucose-induced injury in rat glomerular mesangial cells	2.5 μM	Increase of autophagy by modulating the PI3K/Akt/mTOR pathway	[193]
		Ochratoxin A-induced injury in HEK-293 cells	1 μM	Cytoprotective effect by the modulation of the Lonp1 expression	[194]
		High glucose-induced injury in human mesangial cells	1 mM	Antiproliferative and antioxidant effect by the inhibition of PI3K/Akt/mTOR pathway activation	[195]

Vitexin (**56**)	*A. precatorius, A. brasiliana, A. tenella, A. colubrina, C. pachystachya, C. erosa, C. cajucara, H. balsamifera, L. ferrea, L. paniculata, L. pinaster, N. theifera, P. alata, P. edulis, P. quadrangularis, P. pelucida, P. aduncum, P. marginatum, P. stratiotes, S. dulcis, S. erecta, T. grandiflorum*	CaOx crystals-induced injury in HK-2 cells	10, 20 μM	EMT suppressive effect	[196]
Wedelolactone (**57**)	*E. prostrata*	LPS-induced cell proliferation in HRMC cells	2.5, 5, 10, 20 μM	Antiproliferative effect	[197]
		Cisplatin-induced injury in HEK-293 cells	1 μM	Cytoprotective effect. Reduction of cisplatin accumulation in renal cells by inhibiting the OCT2 uptake activity	[198]
		Doxorubicin-induced injury in MPC-5 cells	1.25, 5, 20 μM	Anti-inflammatory and antioxidant effects	[199]
		LPS-induced injury in HK-2 cells	10 μM	Anti-inflammatory and anti-apoptotic effects	[200]

293T Human embryonic kidney cells, *786-O* Renal cell carcinoma cells, *A498* Human kidney cancer cell, *ACHN* Metastatic renal adenocarcinoma cells, *AGE* Advanced glycation end products, *AKT* Protein kinase B, *AMPK* AMP-activated protein kinase, *AP-1* Activator protein-1, *APC* Activated protein C, *AREG* Amphiregulin, *Bcl2* B-cell lymphoma-2, *BHK-21* Baby hamster kidney cells, *BMP-7* Bone morphogenic protein-7, *CaOx* = Calcium oxalate, *CAV-1* Caveolin-1, *CDKN1B* Cyclin dependent kinase inhibitor 1B, *CREB* CRE-binding protein, *CRL-1927* Mouse mesangial cells, *ECM* Extracellular matrix, *EGFR* Epidermal growth factor receptor, *EMT* Epithelial-to-mesenchymal transition, *ERK* Phosphorylation-extracellular signal-regulated kinase, *EZH2* Enhancer of zeste 2 polycomb repressive complex 2 subunit, *GLUT9* Glucose transporter 9, H_2O_2 Hydrogen peroxide, *HBZY-1* Rat mesangial cell, *HDAC1* Histone deacetylase 1, *HEK-293* Human embryonic kidney cell, *HIF-1α* Hypoxia-inducible factors 1α, *HK-2* Human normal kidney cells, *HKC-8* Human kidney proximal tubular cells, *HMGB2* High-mobility group protein 2, *HRGE* Human renal glomerular endothelial cells, *HRM* Human renal mesangial cells, *ICAM-1* Intercellular adhesion molecule 1, *ILK* Integrin-linked kinase, *JAK* Janus kinases, *KIM-1* Kidney injury molecule-1, *LLC-MK2* Rhesus monkey kidney epithelial cells, *LLC-PK1* Porcine renal tubular cells, *Lonp1* Lon peptidase 1, *LPS* Lipopolysaccharide, *LXRα* Liver X receptor alpha, *MAPK* Mitogen-activated protein kinase, *MCP-1* Monocyte chemoattractant protein-1, *MDCK* Madin-Darby canine kidney cells, *MMP-9* Matrix metalloproteinase-9, *MPC-5* Mouse podocyte clone 5 cells, *MRP* Multidrug resistance-associated protein, *mTOR* Mammalian target of rapamycin, *NaOx* Sodium oxalate, *NF-κB* Factor nuclear kappa B, *NGAL* Lipocalin 2, *NKD2* Naked cuticle homolog 2, *NLRP3* NOD-like receptor protein 3, *NOX* Nicotinamide adenine dinucleotide phosphate oxidase, *NQO1* Quinone oxidoreductase 1, *Nrf2/HO-1* Nuclear factor erythroid-related factor 2/ heme oxygenase-1, *NRIP1* Nuclear receptor interacting protein 1, *NRK-49F* Rat proximal tubular epithelial cells, *NRK-52E* Renal epithelial cells, *OAT* Organic

anion transporter, *OCT2* Organic cation transporter 2, *PARP-1* Poly (ADP-ribose) polymerase 1, *PDGF* Platelet-derived growth factor, *PERK* Protein kinase RNA-like endoplasmic reticulum kinase, *PGC-1α* Peroxisome proliferator-activated receptor-gamma coactivator-1 alpha, *PI3K* Phosphoinositide 3 kinase, *PK15* Porcine kidney cells, *PKCα* Protein kinase C alpha, *pTEC* Renal proximal tubular epithelial cells, *RIP3* Receptor-interacting protein kinase 3, *ROCK* Rho-associated protein kinase, *ROS* Reactive oxygen species, *RPTEC* Primary human renal proximal tubule epithelial cells, *SARS-CoV-2* Severe acute respiratory syndrome coronavirus 2, *SIN1* Stress-activated protein kinase interacting protein 1, *SIRT1* Sirtuin-1, *SMP30* Senescence marker protein30, *STAT* Signal transducer and activator of transcription proteins, *SV40-MES 13* Mouse renal glomerular mesangial cells, *TCMK-1* Transformed C3H mouse kidney-1 cells, *TGFBR1* Transforming growth factor beta receptor 1, *TGF-β1* Transforming growth factor beta 1, *TNF-α* Tumor necrosis factor alpha, *TXNRD1* Thioredoxin reductase 1, *URAT* Urate transporter, *VEGF* vascular endothelial-derived growth factor, *VKORC1* Vitamin K epoxide reductase complex subunit 1, *α-SMA* Alpha smooth muscle actin

Table S2. Nephroprotective activities of secondary metabolites found in native Brazilian plants extracts evaluated through *in vivo* assays

Compound	Source(s)	Experimental model	Effective doses, route of administration	Pharmacological effect	Reference
Acacetin (**58**)	*I. imperati, P. aduncum, P. umbellatum, P. venusta, S. dulcis, S. oleraceus, T. esculenta*	High fat diet and STZ-induced diabetic nephropathy in male Sprague-Dawley rats	25, 50 mg/kg; p.o.	Nephroprotective and anti-inflammatory effects. Improvement of the antioxidant status	[201]
		Renal ischemia and reperfusion-induced damage in male Balb/C mice	50 mg/kg; n.d.	Nephroprotective and anti-inflammatory effects	[202]
		Renal ischemia and reperfusion-induced damage in male Balb/C mice	50 mg/kg; i.p.	Nephroprotective, anti-apoptotic and anti-inflammatory effects	[203]
Afzelin (**1**)	*C. langsdorffii, B. forficata, E. hirta, H. bonariensis, H. leucocephala, S. cernuum, S. chilensis*	Female normotensive and Wistar SHR	0.1, 1 mg/kg; p.o and i.p.	Diuretic, saluretic, Ca^{2+}-sparing effects. Antiurolithiatic and nephroprotective effects	[1]
Aloe-emodin (**2**)	*P. crassipes, S. alata, S. occidentalis, T. crustacea*	UUO-induced nephropathy in male C57BL6 mice	20 mg/kg; p.o.	Antifibrotic effect and reduction of TGF-β1 and fibronectin expressions by the suppression of the PI3K/Akt/mTOR pathway	[2]
Amentoflavone (**59**)	*A. occidentale, B. verbascifolia, C. pareira, T. cacao*	Gentamicin-induced kidney injury in male Wistar rats	40 mg/kg; p.o.	Nephroprotective and anti-inflammatory effects. Improvement of the antioxidant status	[204]

α/β- amyrin (**3**)	*A. edulis, A. cathartica, A. colubrina, B. intermedia, C. halicacabum, C. pachystachya, E. prostrata, E. contortisiliquum, E. hirta, E. thymifolia, H. radicans, L. pinaster, O. monacantha, P. umbellatum, S. dulcis, S. rhombifolia, S. verticillata*	STZ-induced diabetic nephropathy in C57BL/6 mice	50 µg/kg, p.o.	Nephroprotective effect by the restoration of urine β-NAG levels and downregulation of HMGB2	[3]
		Glycerol-induced kidney injury in female Sprague-Dawley rats	50 mg/kg; p.o.	Nephroprotective effect, restoration of biochemical kidney markers. Downregulation of the expression of nestin	[205]
Apigenin (**4**)	*A. precatorius, A. australe, A. colubrina, B. gardneri, B. acmella, C. halicacabum, C. palmata, C. regium, C. erecta, C. spiralis, C. cujete, D. unguis-cati, E. prostrata, E. bonariensis, E. prostrata, E. precatoria, H. impetiginosus, N. theifera, P. edulis, P. tenellus, P. aduncum, P. umbellatum, P. crassipes, S. dulcis, S. occidentalis, S. paniculatum, S. oleraceus, T. cacao, T. crustacea, T. diffusa, U. aurantiaca, U. baccifera, V. polyanthes, V. megapotamica*	Doxorubicin-induced kidney injury in male BALB/c mice	125 mg/kg; p.o.	Nephroprotective and anti-inflammatory effects, reduction of NLRP3, caspase-1 levels	[4]
		Renal ischemia and reperfusion-induced damage in male Sprague-Dawley rats	20 mg/kg; i.p.	Nephroprotective and anti-apoptotic effects, by the regulation of PI3K/Akt mediated mitochondria-dependent apoptosis signaling pathway	[7]
		Oxonate-induced hyperuricemia in male Kunming mice	50, 100 mg/kg; p.o.	Nephroprotective, anti-inflammatory and antifibrotic effects by the suppression of Wnt/β-catenin pathway, promotion of urinary uric acid excretion and inhibition of URAT1	[10]
		Imipenem-induced kidney injury in male New Zealand white rabbits	10 mg/kg; i.p.	Decrease in the renal exposure of imipenem by the inhibition of OAT1/3	[11]
		Methotrexate-induced kidney injury in male CD-1 mice	3 mg/kg; i.p.	Nephroprotective, anti-apoptotic and anti-inflammatory effects. Improvement of the antioxidant status	[206]

		Hypercholesterolemic diet-induced kidney injury in male Wistar rats	50 mg/kg; p.o.	Nephroprotective effect by the modulation of the KIM-1, fibronectin, and Nrf2 signaling pathways	[207]
		Gentamicin-induced kidney injury in male rats	20 mg/kg; p.o.	Nephroprotective, antioxidant and anti-inflammatory effects. Modulation of KIM-1, NGAL, and cystatin C levels	[208]
		Lead-induced nephrotoxicity in male Wistar rats	20 mg/kg; p.o.	Nephroprotective, antioxidant and anti-inflammatory effects. Upregulation of Nrf2 and HO-1 levels	[209]
		Cisplatin-induced kidney injury in male BALB/c mice	20 mg/kg; i.p.	Nephroprotective, antioxidant and anti-inflammatory effects	[210]
		Mesoporous silica nanoparticles-induced kidney injury in male BALB/c mice	40 mg/kg; i.p.	Suppression of oxidative stress and inflammation via the FOXO3a/NF-κB pathway	[211]
		Oxazinate/hypoxanthine-induced hyperuricemia in male Kunming mice	100 mg/kg; p.o.	Nephroprotective effect by the inhibition of uric acid production, and suppression of JAK2/STAT3 signaling pathway	[212]
		Deoxycorticosterone acetate salt-induced hypertension in male Sprague-Dawley rats	0.2% of the diet; p.o.	Antifibrotic effect by the inhibition of TGF-β1/Smad2/3 signaling pathway	[213]
		Ethylene glycol and NH_4Cl-induced urolithiasis in male Wistar rats	0.005, 0.01, and 0.02 g/kg; p.o.	Antilithic effect, improvement of the antioxidant status and inhibition of TGF-β1 signaling pathway	[214]
		Cisplatin-induced kidney injury in female Wistar rats	3 mg/kg; i.p.	Nephroprotective, anti-inflammatory and antioxidant effects	[215]
		Nickel oxide nanoparticles-induced kidney injury in male Wistar rats	25 mg/kg; p.o.	Nephroprotective effect by the improvement of the antioxidant status	[216]

		Ethylene glycol and NH_4Cl-induced urolithiasis in male Wistar rats	1.2, 2.4, and 4.8 mg/kg; p.o.	Decrease of calcium levels in the kidneys	[217]
		STZ-induced diabetic nephropathy in male Wistar rats	20 mg/kg; p.o.	Nephroprotective and anti-inflammatory effects, reduction of collagen deposition, MAPK activation and glomerulosclerosis in the renal tissue	[218]
		3-MCPD-induced kidney toxicity in male Sprague-Dawley rats	20, 40 mg/kg; p.o.	Nephroprotective effect by the modulation of mitochondria dependent caspase cascade pathway	[219]
		Edifenphos-induced nephrotoxicity in male Wistar rats	10 mg/kg; p.o.	Nephroprotective and anti-apoptotic effects. Improvement of the antioxidant status	[220]
		STZ-induced diabetic nephropathy in male Wistar rats	0.78 mg/kg; s.c.	Reduction of kidney and urinary lesions	[221]
		Methotrexate-induced kidney injury in male Wistar rats	20 mg/kg; p.o.	Nephroprotective, anti-inflammatory and autophagic effects by modulation of NOD-2 and p-NF-κB p65/NLRIP3/IL-1β trajectory	[222]
Asiatic acid (**60**)	*C. asiatica*	UUO-induced nephropathy in male C57BL6 mice	4, 16 mg/kg; p.o	Reduction of tubulointerstitial fibrosis and tubular injury	[223]
		Doxorubicin-induced kidney toxicity in Wistar rats	5, 10, 20 mg/kg; p.o.	Nephroprotective effect	[224]
		STZ-induced diabetic nephropathy in male Sprague-Dawley rats	10, 20, 40 mg/kg; p.o.	Nephroprotective effect	[225]

		Cisplatin-induced kidney injury in male C57BL/6 mice	50, 100 mg/kg; i.p.	Nephroprotective, anti-inflammatory and anti-apoptotic effects	[226]
		UUO-induced nephropathy in male Sprague-Dawley rats and ICR mice	10, 15 mg/kg; p.o.	Antifibrotic and neuroprotective effects	[227]
		LPS-induced kidney injury in broilers	15, 30, 60 mg/kg; p.o.	Nephroprotective effect	[228]
		STZ-induced diabetic nephropathy in male Sprague-Dawley rats	10, 30 mg/kg; p.o.	Nephroprotective effect	[229]
		STZ and NAD-induced diabetic nephropathy in male rats	20 mg/kg; p.o.	Nephroprotective effect	[230]
		Cisplatin-induced kidney fibrosis in male mice	50, 100 mg/kg; i.p.	Nephroprotective effect	[231]
Asiaticoside (**61**)	*C. asiatica*	Adriamycin-induced nephropathy in SD male rats	8, 16, 32 mg/kg; p.o.	Nephroprotective effect	[232]
		Renal ischemia and reperfusion-induced damage in male C57BL/6 mice	20, 40, 60 mg/kg; i.p.	Nephroprotective effect	[233]
		Renal ischemia and reperfusion-induced damage in male C57BL/6 mice	40 mg/kg; p.o.	Nephroprotective effect	[234]
Astragalin (**5**)	*A. occidentale, G. ulmifolia, P. amarus, P. niruri, S. cernuum*	STZ-induced diabetic nephropathy in male C57BL/6 mice	5, 10 mg/kg; p.o.	Nephroprotective and anti-apoptotic effects by the reduction of proteinuria, oxidative stress and highly activated renal aldose reductase activity	[12]

Barbinervic acid (**62**)	*E. punicifolia*	Noradrenaline-induced increase of renal circulation in Sprague Dawley rats	IC_{50} = 30 μM; *ex vivo*	NO-dependent reduction of the renal tonus	[235]
β-sitosterol (**63**)	*A. conyzoides, A. cathartica, A. occidentale, A. colubrina, A. comosus, B. pentandra, B. acutifolium, C. halicacabum, C. sylvestris, C. filiformis, C. pachystachya, C. erosa, C. rufescens, C. cajucara, D. unguis-cati, D. dentatus, D. brasiliensis, E. prostrata, E. uniflora, E. hirta, E. thymifolia, F. crocata, G. americana, G. viburnoides, H. brasiliense, H. crispa, H. radicans, I. suffruticosa, I. diffusa, L. pinaster, M. glomerata, M. balsamum, P. pelucida, P. americana, P. sellowianus, P. umbellatum, P. stratiotes, P. crassipes, P. guineense, S. dulcis, S. occidentalis, S. rhombifolia, S. versicolor, S. guianensis, S. verticillata, S. odoratissima, T. rhomboidea, T. semitriloba, T. diffusa*	Renal ischemia and reperfusion-induced damage in female Wistar rats	150 mg/kg; p.o.	Nephroprotective, anti-necrotic and anti-apoptotic effects	[236]
		DEN and Fe-NTA-induced kidney injury in male Wistar rats	20 mg/kg; p.o.	Nephroprotective effect by the upregulation of Nrf2 gene expression	[237]
		DEN and Fe-NTA-induced renal carcinogenesis in male Wistar rats	20 mg/kg; p.o.	Prevention of cellular proliferation and apoptotic effect	[238]
Bergapten (**64**)	*B. gaudichaudii, D. brasiliensis, D. cayapia subsp. asaroide, P. pennatifolius*	Cyclophosphamide-induced nephrotoxicity in male Wistar rats	10 mg/kg; i.p.	Nephroprotective, antifibrotic, antioxidant and anti-inflammatory effects	[239]

Betulinic acid (**6**)	*A. amazonicus, A. colubrina, B. intermedia, C. erosa, D. dentatus, E. prostrata, E. contortisiliquum, E. uniflora, H. impetiginosus, P. rigida, S. dulcis, S. alata*	STZ-induced diabetic nephropathy in male C57BL/6 mice	20 mg/kg; p.o.	Nephroprotective and anti-inflammatory effects	[240]
		Adenine-induced kidney injury in male Wistar rats	30 mg/kg; p.o.	Nephroprotective and antifibrotic effects	[241]
		NDMA-induced kidney injury in male Wistar rats	25 mg/kg; p.o.	Nephroprotective, antioxidant and anti-inflammatory effects	[242]
		Cisplatin-induced kidney injury in male Wistar rats	30, 50 mg/kg; p.o.	Nephroprotective, antioxidant, autophagic and anti-inflammatory effects	[243]
		Cadmium-induced kidney injury in male Wistar rats	3, 10, 30 mg/kg; p.o.	Nephroprotective and anti-apoptotic effects	[244]
		T-2 toxin-induced kidney injury in male Kunming mice	0.25, 0.5, 1 mg/kg; p.o.	Nephroprotective and anti-inflammatory effects by the activation of Nrf2 pathway	[245]
		Cyclophosphamide-induced nephrotoxicity in male rats	20, 40 mg/kg; p.o.	Nephroprotective, antioxidant and anti-inflammatory effects	[246]
		Renal ischemia and reperfusion-induced damage in Wistar rats	250 mg/kg; i.p.	Nephroprotective effect by the regulation of apoptotic function of leukocytes and inhibition of neutrophil infiltration	[247]
		Cecal ligation and puncture injury-induced sepsis and nephropathy in Swiss mice	10, 30 mg/kg; i.p.	Nephroprotective, antioxidant and anti-inflammatory effects	[248]
		PHN in female Sprague-Dawley rats	25, 50 mg/kg; p.o.	Nephroprotective and anti-inflammatory effects by the regulation of Nrf2/NF-κB pathway	[249]
		STZ-induced diabetic nephropathy in male Sprague-Dawley mice	20 mg/kg; p.o.	Antifibrotic effect by the inhibition of NF-κB activation	[250]

		NDMA-induced kidney injury in male Wistar rats	25 mg/kg; p.o.	Nephroprotective, antioxidant and anti-apoptotic effects	[251]
		Cyclophosphamide-induced nephrotoxicity in male rats	0.5, 1 mg/kg; p.o.	Nephroprotective, anti-inflammatory and antioxidant effects by the modulation of NF-κB and ERK-mediated mitochondrial apoptotic pathway	[252]
		STZ-induced diabetic nephropathy in male Sprague-Dawley rats	20, 40 mg/kg; p.o.	Nephroprotective, anti-inflammatory and antioxidant effects by the regulation of AMPK/NF-κB/Nrf2 signaling pathway	[253]
		STZ and NAD-induced diabetic nephropathy in male NMRI mice	10, 20, 40 mg/kg; p.o.	Nephroprotective by the regulation of albumin, plasma urea nitrogen and creatinine levels	[254]
Bixin (**7**)	*B. orellana*	UUO-induced nephropathy in male C57BL/6 mice	100 mg/kg; i.p.	Prevention of renal interstitial fibrosis	[15]
		UUO-induced nephropathy in STAT6 mice	100 mg/kg; i.p.	Prevention of renal interstitial fibrosis	[16]
		CCl_4-induced nephrotoxicity in male ICR mice	100, 200 mg/kg; p.o.	Anti-inflammatory and antifibrotic effects	[255]
Caffeic acid (**8**)	*A. conyzoides, A. edulis, A. comosus, A. fraxinifolium, C. indica, C. halicacabum, C. palmata, C. erecta, C. antisyphiliticus, C. ingrata, C. americana, D. unguis-cati, E. viscosa, E. bonariensis, E. crassipes, E. giganteum, E. foetidum, E. hirta, E. precatoria, G. ulmifolia, H.*	Oxonate-induced hyperuricemia in male Swiss mice	15 mg/kg; p.o.	Hypouricemic and anti-inflammatory effects	[256]
		Male Wistar rats	10 mg/kg; p.o.	Diuretic effect. Increase in urinary excretion of Na^+, K^+ and Ca^{2+}	[18]
		Ethylene glycol- induced urolithiasis in Male Wistar rats	20, 40 mg/kg; p.o.	Antilithic effect, upregulation of bikunin gene	[257]
		STZ and high fat diet-induced diabetic nephropathy in male Wistar rats	40 mg/kg; p.o.	Nephroprotective effect by modulating the autophagy pathway	[258]

	impetiginosus, I. paraguariensis, I. pes-caprae, J. gossypiifolia, L. pinaster, M. elliptica, O. monacantha, P. niruri, P. tenellus, R. viburnoides, S. alata, S. reticulata, S. rhombifolia, S. fluminensis, S. verticillata, S. lycocarpum, S. paniculatum, S. viarum	STZ-induced diabetic nephropathy in male Balb/c mice	2.5% of the diet; p.o.	Anti-glycative and anti-inflammatory renal effects	[259]
		Renal ischemia and reperfusion-induced damage in Wistar rats	100 mg/kg; p.o.	Nephroprotective and anti-inflammatory effects	[260]
		Capecitabine-induced kidney injury in male Wistar rats	100 mg/kg; p.o.	Nephroprotective and antioxidant effects	[261]
		Aflatoxin B1-induced nephrotoxicity in male Wistar rats	20, 40 mg/kg; p.o.	Nephroprotective effect. Reduction of DNA damage, oxidative and inflammatory mediated toxicities	[261]
		UUO-induced nephropathy in male C57BL/6J mice	25 mg/kg; p.o.	Renal tubulointerstitial antifibrotic effect	[263]
		Valproate-induced kidney injury in male Sprague–Dawley rats	50 mg/kg; p.o.	Nephroprotective, anti-inflammatory, and anti-apoptotic effects	[264]
Caffeine (**9**)	*I. paraguariensis, T. cacao, T. diffusa*	A1 receptor +/+ and -/- mice	45 mg/kg; p.o.	Diuretic and natriuretic effects	[265]
		Tubulus-specific NHE3 knockout mice	45 mg/kg; p.o.	Diuretic and natriuretic effects	[266]
		Male Pkd1-deficient cystic mice	3 mg/day; p.o.	Deterioration of kidney function	[267]
		Diclofenac-induced kidney injury in male albino rats	15 mg/kg; p.o.	Nephroprotective effect	[268]
		Cisplatin-induced kidney injury in male Balb/c mice	0.1, 0.5, 1 % of the diet; p.o.	Nephroprotective effect	[269]

Catechin (**10**)	*A. edulis, A. occidentale, A. colubrina, A. comosus, B. argyrophylla, B. laevifolia, B. intermedia, C. pachystachya, C. palmata, C. cajucara, C. heliotropiifolius, C. americana, E. biflora, E. involucrata, E. precatoria, G. ulmifolia, H. tomentosa, L. pisonis, L. ferrea, M. ilicifolia, M. elliptica, O. monacantha, P. alata, P. americana, P. hydropiperoides, P. brasiliensis, P. niruri, P. tenellus, S. humboldtiana, S. molle, S. terebinthifolia, S. dulcis, S. reticulata, S. campestris, S. mombin, S. adstringens, T. esculenta, T. cacao, T. grandiflorum, V. megapotamica, X. americana*	Melamine and cyanuric acid-induced urolithiasis in male Sprague-Dawley rats	2.5, 10 mg/kg; p.o.	Nephroprotective, antilithic and anti-apoptotic effects	[22]
		Ethylene glycol- induced urolithiasis in male Sprague-Dawley rats	2.5, 10 mg/kg; p.o.	Antilithic effect, improvement of the antioxidant status	[23]
		Cadmium-induced nephrotoxicity in male Wistar rats	25, 50, and 100 mg/kg; p.o.	Nephroprotective and anti-inflammatory effects. Kidney mitochondrial protection	[270]
		Benzo [a] pyrene-induced kidney injury in male Wistar rats	20 mg/kg; p.o.	Nephroprotective and anti-apoptotic effects	[271]
		Melamine and cyanuric acid-induced urolithiasis in male Sprague-Dawley rats	50 mg/kg; p.o.	Nephroprotective and antilithic effects	[272]
		Gentamicin-induced kidney injury in male Wistar rats	50 mg/kg; p.o.	Nephroprotective and antioxidant effects	[273]
		Diabetic male C57BL/KsJ db/db mice	15, 30, 60 mg/kg; p.o.	Nephroprotective and anti-inflammatory effects, inhibition of AGE formation	[274]
		Glycerol-induced myoglobinuric kidney damage in male Wistar rats	40 mg/kg; p.o.	Nephroprotective effect, improvement of the antioxidant status	[275]
		Fe-NTA-induced kidney injury in male Wistar rats	40 mg/kg; p.o.	Nephroprotective effect, improvement of the antioxidant status	[276]

		Cyclosporine-induced kidney injury in Wistar rats	100 mg/kg; p.o.	Nephroprotective effect, improvement of the antioxidant status	[277]
		Ethylene glycol- induced urolithiasis in male Wistar rats	100 mg/L in drinking water; p.o.	Nephroprotective and antioxidant effects by the induction of PON1 activity	[278]
Chlorogenic acid (**11**)	*A. hispidum, A. edulis, A. brasiliana, A. tenella, A. colubrina, A. comosus, B. intermedia, C. halicacabum, C. halicacabum, C. pachystachya, C. palmata, C. peltata, C. americana, D. unguis-cati, D. brasiliensis, E. viscosa, E. foetidum, E. precatoria, G. ulmifolia, H. impetiginosus, H. tomentosa, I. paraguariensis, I. imperati, I. pes-caprae, L. paniculata, L. pinaster, M. elliptica, O. campechianum, P. americana, P. tenellus, P. crassipes, P. pubescens, R. viburnoides, S. australis, S. alata, S. rhombifolia, S. lycocarpum, S. paniculatum, S. chilensis, S. oleraceus, S. mombin, T. paniculatum, T. esculenta, T. cacao, X. aromatica*	Oxonate-induced hyperuricemia in male Swiss mice	15 mg/kg; p.o.	Hypouricemic and anti-inflammatory effects	[256]
		UUO-induced nephropathy in male Swiss Webster mice	14 mg/kg; i.p.	Antifibrotic effect	[279]
		Renal ischemia and reperfusion-induced damage in male Swiss mice	3.5, 7, 14 mg/kg; i.p.	Nephroprotective and anti-inflammatory effects	[280]
		Tamoxifen-induced kidney injury in female Wistar rats	25, 50 mg/kg; p.o.	Nephroprotective effect	[281]
		D-galactose-induced kidney injury in male Kunming mice	200 mg/kg of the diet; p.o.	Nephroprotective and anti-inflammatory effects	[282]
		LPS-induced kidney injury in female C57BL/6 mice	5, 10, 20 mg/kg; i.p.	Nephroprotective and anti-inflammatory effects	[283]
		Cisplatin-induced kidney injury in male Balb/c mice	3, 10, 30 mg/kg; i.p.	Nephroprotective, anti-inflammatory and anti-apoptotic effects	[284]
		Lead-induced nephrotoxicity in male C57BL/6 mice	10, 20, 40 mg/kg; i.p.	Nephroprotective and anti-apoptotic effects	[285]

		Vancomycin-induced kidney injury in male Wistar mice	150 mg/kg; p.o.	Nephroprotective, anti-inflammatory and anti-apoptotic effects	[286]
		Adenine-induced hyperuricemic nephropathy in Sprague-Dawley rats	40 mg/kg; p.o.	Nephroprotective and antifibrotic effects	[287]
		Hypoxanthine and oxonate-induced hyperuricemia in male Kunming mice	30, 60 mg/kg; p.o.	Nephroprotective and anti-inflammatory effects	[288]
		Arsenite-induced nephrotoxicity in male Swiss mice	100, 200 mg/kg; p.o.	Nephroprotective, anti-inflammatory and anti-apoptotic effects	[289]
		STZ-induced diabetic nephropathy in male Sprague-Dawley rats	5, 10, 20 mg/kg; i.p.	Nephroprotective and anti-apoptotic effects	[290]
		STZ-induced diabetic nephropathy in male Sprague-Dawley rats	10 mg/kg; i.p.	Nephroprotective and anti-inflammatory effects	[291]
		Lead-induced nephrotoxicity in male Kunming mice	30 mg/kg; p.o.	Nephroprotective effect	[292]
		Renal ischemia and reperfusion-induced damage in male Sprague-Dawley rats	20 mg/kg; i.p.	Nephroprotective effect	[293]
		Cadmium-induced nephrotoxicity in male Kunming mice	30 mg/kg; p.o. and 500 mg/kg; i.p.	Nephroprotective effect	[294]
		Ochratoxin-induced nephrotoxicity in male Wistar rats	5 mg/kg; p.o.	Nephroprotective effect	[295]

Chrysophanol (**12**)	*S. occidentalis*	UUO-induced nephropathy in male BALB/c mice	20, 40 mg/kg; p.o.	Nephroprotective and antifibrotic effects by the inhibition of NKD2/NF-κB pathway	[28]
		Cisplatin-induced kidney injury in male C57BL6/J mice	20, 40 mg/kg; p.o.	Nephroprotective, antioxidant, anti-inflammatory and anti-apoptotic effects. Inhibition of IKKβ/IκBα/p65/NF-κB signaling pathway	[29]
		UUO-induced nephropathy in male C57BL/6 mice	10, 40 mg/kg; p.o.	Nephroprotective and antifibrotic effects by downregulating TGF-β1 and phospho-Smad3 expressions	[30]
		High sugar and fat diet/STZ-induced diabetic nephropathy in male C57BL/6 mice	2.5, 5, 10 mg/kg; p.o.	Nephroprotective and anti-inflammatory effects. Reduced of kidney oxidative stress and pyroptosis by activating the Keap1/Nrf2 pathway	[296]
Cinnamic acid (**65**)	*A. edulis, A. colubrina, C. sylvestris, C. cujete, C. heliotropiifolius, L. pinaster*	Oxonate-induced hyperuricemia in male Swiss mice	15 mg/kg; p.o.	Hypouricemic and anti-inflammatory effects	[256]
		Gentamicin-induced kidney injury in male Wistar rats	50 mg/kg; i.p.	Nephroprotective effect	[297]
		Cisplatin-induced kidney injury in male Wistar rats	50 mg/kg; p.o.	Nephroprotective effect	[298]
p-coumaric acid (**66**)	*A. capillus-veneris, A. edulis, A. colubrina, A. comosus, A. fraxinifolium, C. halicacabum, C. palmata, C. ingrata, D. unguis-cati, E. precatoria, G. ulmifolia, H. impetiginosus, L. pisonis, M. elliptica, O.*	STZ-induced diabetic nephropathy in male Sprague-Dawley rats	100 mg/kg; p.o.	Nephroprotective, anti-inflammatory, antifibrotic effects. Downregulation of TLR-4 activation	[299]
		Cadmium-induced nephrotoxicity in male Wistar rats	100 mg/kg; p.o.	Nephroprotective effect by the restoration of kidney biochemical markers and antioxidant status	[300]

	monacantha, P. americana, P. niruri, P. crassipes, S. reticulata, S. rhombifolia, T. esculenta, T. crustacea, V. megapotamica	Cisplatin-induced kidney injury in male Wistar rats	100 mg/kg; i.p.	Nephroprotective effect by the restoration of kidney antioxidant status	[301]
		Doxorubicin-induced kidney injury in male Wistar rats	100 mg/kg; p.o.	Nephroprotective, antiinflammatory and anti-apoptotic effects	[302]
		Renal ischemia and reperfusion-induced damage in male Sprague– Dawley rats	100 mg/kg; p.o.	Nephroprotective and anti-inflammatory effects. Restoration of kidney antioxidant status	[303]
		Fipronil-induced renal injury in male Swiss mice	100 mg/kg; i.p.	Nephroprotective and anti-inflammatory effects. Restoration of kidney antioxidant status	[304]
Cyanidin-3-*O*-glucoside (**13**)	*A. occidentale, E. punicifolia, E. hirta, E. precatoria, P. niruri, P. tenellus, P. peruviana*	Renal ischemia and reperfusion-induced damage in male C57BL/6 mice	10 mg/kg; i.p.	Nephroprotective effect against ferroptosis by AMPK activation	[32]
		Renal ischemia and reperfusion-induced damage in male C57BL/6 mice	200 mg/kg; p.o.	Nephroprotective and antifibrotic effects. Reduction of the endoplasmic reticulum stress	[33]
		Diabetic male C57BL/KsJ db/db mice	10 mg/kg; p.o.	Nephroprotective effect by the regulation of aminoacids metabolism	[305]
		Diabetic male C57BL/KsJ db/db mice	10, 20 mg/kg; i.p.	Nephroprotective, antifibrotic and anti-inflammatory effects. Reduction of kidney lipid accumulation	[306]
		Ochratoxin A-induced nephrotoxicity in male Sprague–Dawley rats	1 g/kg of the diet; p.o.	Nephroprotective effect by the reduction of the DDAH/NOS pathway expression	[307]
		STZ-induced diabetic nephropathy in male Sprague–Dawley rats	10, 20 mg/kg; p.o.	Nephroprotective and anti-inflammatory effects by the regulation of TGF-β1/Smad2/3 pathway	[308]

		Ochratoxin A-induced nephrotoxicity in male Sprague–Dawley rats	1 g/kg of the diet; p.o.	Nephroprotective effect by the antioxidant and HO-1-inducing properties.	[309]
Ellagic acid (**15**)	*A. comosus, C. villosum, C. villosum, C. antisyphiliticus, C. heliotropiifolius, C. ingrata, E. biflora, E. uniflora, G. ulmifolia, L. pisonis, L. ferrea, M. velame, P. amarus, P. niruri, P. peruviana, S. mombin, T. esculenta, T. diffusa, X. americana*	Glyoxylate-induced renal injury in male C57BL/6 mice	20 mg/kg; p.o.	Nephroprotective effect	[39]
		Adenine-induced hyperuricemic nephropathy in C57BL/6J mice	0.1% of the diet; p.o.	Nephroprotective and antifibrotic effects	[40]
		STZ-induced diabetic nephropathy in male Balb/c mice	5% of the diet; p.o.	Nephroprotective, anti-glycative and anti-inflammatory effects	[259]
		$K_2Cr_2O_7$-induced renal injury in male Wistar rats	30 mg/kg; p.o.	Nephroprotective and anti-inflammatory effects	[310]
		D-GalN- induced kidney damage in male Sprague– Dawley rats	20 mg/kg; p.o.	Nephroprotective effect	[311]
		Nicotine- induced kidney injury during the fetal period in Sprague– Dawley rats	60 mg/kg; i.p.	Nephroprotective and anti-apoptotic effects	[312]
		Subtotal nephrectomy- induced male Sprague– Dawley rats	20, 40 mg/kg; p.o.	Nephroprotective effect	[313]
		Renal ischemia and reperfusion-induced damage in male Sprague-Dawley rats	50, 100, 150 mg/kg; p.o.	Nephroprotective effect	[314]
		Gentamicin- induced kidney injury in male Sprague– Dawley rats	10 mg/kg; p.o.	Nephroprotective and anti-apoptotic effects	[315]

		Aging- induced kidney damage in male Wistar rats	30 mg/kg; p.o.	Nephroprotective effect by the activation of SIRT1 and NRF2 signaling	[316]
		Oxonate-induced hyperuricemia in male ICR mice	50, 100 mg/kg; i.p.	Antihyperuricemic and anti-inflammatory effects	[317]
		Lead-induced nephrotoxicity in male Wistar rats	25, 50 mg/kg; p.o.	Nephroprotective and anti-apoptotic effects	[318]
		Cisplatin-induced kidney injury in male Sprague– Dawley rats	10, 30 mg/kg; p.o.	Nephroprotective, anti-inflammatory and anti-apoptotic effects	[319]
		Cisplatin-induced kidney injury in male Sprague– Dawley rats	10 mg/kg; p.o.	Nephroprotective effect	[320]
		TCDD-induced kidney injury in male Wistar rats	10 mg/kg; p.o.	Nephroprotective effect	[321]
		STZ-induced diabetic nephropathy in male ICR mice	50, 100, 150 mg/kg; p.o.	Nephroprotective and anti-inflammatory effects	[322]
		Cyclosporine-induced kidney injury in male Sprague– Dawley rats	10 mg/kg; p.o.	Nephroprotective effect	[323]
		Ifosfamide-induced kidney injury in male Wistar rats	25 mg/kg; i.p.	Nephroprotective effect	[324]
		Gentamicin- induced kidney injury in male Wistar rats	10 mg/kg; p.o.	Nephroprotective effect	[325]
		Cyclophosphamide- induced nephrotoxicity in male Swiss mice	50, 100 mg/kg; p.o.	Nephroprotective effect	[326]

Emodin (**16**)	*H. impetiginosus, S. occidentalis*	UUO-induced nephropathy in male Sprague– Dawley rats	30 mg/kg; n.d.	Nephroprotective and antifibrotic effects by the regulation of PGC-1α pathway	[42]
		UUO-induced nephropathy in male Sprague– Dawley rats	26 mg/kg; p.o.	Antifibrotic effect by the reduction of extracellular collagen deposition and downregulation of Smad3, CTGF and EZH2 signaling pathways	[43]
		Renal ischemia and reperfusion-induced damage in C57BL/6 male mice	3, 10 mg/kg; i.p.	Nephroprotective and anti-apoptotic effects by the inhibition of CAMKII activation and reduction of DRP1 phosphorylation at Ser616	[45]
		Bilateral renal artery clipping-induced injury in Sprague–Dawley rats	30 mg/kg; p.o.	Anti-apoptotic and angiogenic effect by the regulation of p53/Caspase-9/Caspase-3, p53/Bcl-2 and HIF-1α/VEGF signaling pathway	[46]
		Lupus nephritis-prone male BXSB mice	5, 10, 20 mg/kg; p.o.	Nephroprotective effect by the reduction of TNF-α, ICAM-1 and fibronectin levels. Reduction of urine protein and serum anti-dsDNA antibody levels	[47]
		UUO-induced nephropathy in C57BL/6 mice	50 mg/kg; p.o.	Antifibrotic effect by the inhibition of α-SMA and p-Smad2 expression	[49]
		Diabetic nephropathy in KK-Ay mice	40, 80 mg/kg; p.o.	Nephroprotective effect by the restoration of kidney markers, increase of nephrin expression and inhibition of PERK-eIF2α signaling pathway	[51]
		STZ-induced diabetic nephropathy in male Wistar rats	20 mg/kg; p.o.	Nephroprotective effect by desmin and ILK inhibition and nephrin upregulation	[52]

		High fat diet and STZ- induced nephropathy in male C57BL/6J mice	50 mg/kg; p.o.	Nephroprotective effect by the upregulation of GLP-1R activity	[327]
		Unilateral nephrectomy and STZ-induced nephropathy in male Sprague– Dawley rats	20, 40 mg/kg; p.o.	Anti-apoptotic effect. Enhancement of autophagy of podocytes by the regulation of AMPK/mTOR signaling pathway	[328]
		5/6 nephrectomy- induced kidney injury in male Sprague– Dawley rats	1 mg/day; colonic irrigation	Nephroprotective effect and reduction of uremic toxins production by the alteration in gut microbiota structure	[329]
		Cisplatin-induced kidney injury in male Wistar rats	10 mg/kg; p.o.	Nephroprotective and antinecrotic effects. Reduction in urinary N-acetyl-β-D-glucosaminidase activity, osmolarity and protein concentrations	[330]
		STZ-induced diabetic nephropathy in female adult Wistar rats	100 mg/kg; n.d.	Anti-inflammatory effect, suppression of ICAM-1 and Bax, and activation of the PI3K/Akt/GSK-3β pathway.	[331]
Epicatechin (**17**)	*A. cathartica, A. occidentale, A. comosus, A. spinescens, B. laevifolia, B. intermedia, C. pachystachya, C. cajucara, D. rugosa, E. involucrata, E. precatoria, G. ulmifolia, H. courbaril, L. pisonis, M. ilicifolia, P. alata, P. americana, P. hydropiperoides, P. brasiliensis, S. reticulata, S. mombin, S. adstringens, T.*	Cisplatin-induced kidney injury in male C57BL/6J mice	1 mg/kg; i.p.	Nephroprotective effect, reduction of the renal mitochondrial oxidative stress	[58]
		LPS-induced kidney injury in male Sprague– Dawley rats	80 mg/kg; p.o.	Nephroprotective and anti-inflammatory effects by the inhibition of TLR4 upregulation and NOX activation	[332]
		Fructose-induced kidney injury in male Sprague– Dawley rats	20 mg/kg; p.o.	Nephroprotective, antifibrotic and anti-inflammatory effects	[333]
		Subtotal nephrectomy- induced male C57BL6 mice	0.01, 0.1, 1 mg/kg; p.o.	Nephroprotective effect	[334]

	esculenta, T. esculenta, T. cacao, T. grandiflorum, V. megapotamica	Ethylene glycol and NH_4Cl-induced urolithiasis in male Wistar rats	200 mg/L in drinking water; p.o.	Antilithic effect, improvement of the antioxidant status	[335]
Epicatechin gallate (**18**)	*E. hirta, L. pisonis, P. niruri*	LPS/ischemia-induced kidney injury in male Wistar rats	10, 20 μmol/kg; p.o.	Nephroprotective effect, improvement of the antioxidant status	[60]
		Cisplatin-induced kidney injury in male Wistar rats	5 mg/kg; i.p.	Nephroprotective, anti-apoptotic and anti-inflammatory effects by the downregulation of MAPK pathway	[336]
Epigallocatechin-3-gallate (**19**)	*M. ilicifolia, P. niruri, S. adstringens*	Cisplatin-induced kidney injury in male C57BL/6 mice	100 mg/kg; i.p.	Nephroprotective, antioxidant, anti-apoptotic and anti-inflammatory effects	[63]
		Glycerol-induced myoglobinuric renal damage in male Wistar rats	5, 10 mg/kg; i.p.	Nephroprotective, anti-apoptotic and anti-inflammatory effects. Reduction of mitochondrial apoptotic pathway markers	[64]
		UUO-induced nephropathy in male mice	5 mg/kg; i.p.	Nephroprotective, antifibrotic and anti-apoptotic effects by the inhibition of the TGF-β1-estimulated phosphorylation of MAPK	[65]
		Immune-mediated glomerulonephritis in male 129/svJ mice	50 mg/kg; p.o.	Nephroprotective and anti-inflammatory effects by the modulation of antioxidant status and PPARγ levels	[337]
		Lupus nephritis in NZB/W F1 lupus-prone mice	120 mg/kg; p.o.	Nephroprotective and anti-inflammatory effects by the reversion of proteinuria and modulation of Nrf2 antioxidant and of renal NLRP3 inflammasome activation	[338]

		STZ-induced diabetic nephropathy in male Wistar rats	25, 50, 100 mg/kg; p.o.	Nephroprotective and antioxidant effects, reduction of the renal AGE products accumulation	[339]
		Cisplatin-induced kidney injury in male Wistar rats	100 mg/kg; p.o.	Nephroprotective effect by the modulation of Nrf2/HO-1 signaling pathway and NF-κB levels	[340]
		Immune-mediated glomerulonephritis in male 129/svJ mice	25, 50 mg/kg; p.o.	Nephroprotective and anti-inflammatory effects, modulation of the antioxidant status	[341]
		UUO-induced nephropathy in male Sprague– Dawley rats	2.5, 5, 10 mg/kg; i.p.	Nephroprotective effect by the activation of the Nrf2 signaling pathway	[342]
		UUO-induced nephropathy in male Wistar rats	50 mg/kg; i.p.	Nephroprotective effect by the decrease of KIM-1, NGAL and TNF-α expressions	[343]
		High-fat food feeding and STZ-induced nephropathy in male Sprague-Dawley rats	40, 80 mg/kg; p.o.	Nephroprotective and anti-inflammatory effects mediated by the inhibition of the NLRP3 inflammasome overactivation	[344]
		Cadmium-induced nephrotoxicity in male Wistar rats	100, 200 mg/kg; p.o.	Nephroprotective effect, regulation of the renal TGF-β1 pathway and EMT suppression	[345]
		Adenine-induced hyperuricemic nephropathy in C57BL/6J mice	20, 100, 500 mg/kg; p.o.	Nephroprotective effect, inhibition of methyl guanidine production	[346]
		STZ-induced diabetic nephropathy in male ICR mice	100 mg/kg; s.c.	Nephroprotective effect by the suppression of osteopontin production	[347]
Ferulic acid (**20**)	*A. edulis, A. brasiliana, A. tenella, A. colubrina, A. comosus, C. indica, C.*	STZ-induced diabetic nephropathy in male Wistar rats	50 mg/kg; p.o.	Nephroprotective, anti-apoptotic and anti-inflammatory effects. Modulation of MAPK and NF-κB pathways	[66]

	halicacabum, C. palmata, C. antisyphiliticus, C. ingrata, D. unguis-cati, E. giganteum, E. foetidum, E. hirta, E. precatoria, G. ulmifolia, L. pisonis, M. elliptica, O. monacantha, P. americana, P. aduncum, P. crassipes, S. alata, S. reticulata, S. rhombifolia, S. alternatopinnatum, S. viarum, T. esculenta, T. cacao, T. crustacea, V. megapotamica	LPS-induced kidney injury in male C57BL/6 mice	50 mg/kg; i.p.	Nephroprotective and anti-inflammatory effects by the upregulation of AMPKα1 expression	[67]
		Male SHR	50 mg/kg; p.o.	Nephroprotective effects	[348]
		Renal ischemia and reperfusion-induced damage in male C57/BL6 mice	30, 100 mg/kg; p.o.	Nephroprotective effect by the reduction of apoptosis, inflammation, increase of adenosine generation and upregulation of HIF-1α	[349]
		Gentamicin- induced kidney injury in female Wistar rats	50 mg/kg; p.o.	Nephroprotective, antioxidant and anti-inflammatory effects	[350]
		Cyclosporine- induced kidney injury in Wistar rats	50 mg/kg; p.o.	Nephroprotective and anti-infammatory effects by the activation of Nrf2/HO-1 signaling and suppression of NF-κB/TNF-α axis	[351]
		UUO-induced nephropathy in male mongrel dogs	70 mg/kg; p.o.	Nephroprotective, antioxidant and antifibrotic effects	[352]
		LPS-induced kidney injury in female Balb/c mice	50, 100 mg/kg; i.p.	Nephroprotective and anti-inflammatory effects by the inhibition of TLR-4 mediated NF-κB activation	[353]
		STZ-induced diabetic nephropathy in male C57BL/6J mice	200 mg/kg; p.o.	Nephroprotective effect by the induction of autophagy and reduction of inflammatory response	[354]
		Cisplatin- induced kidney injury in male Wistar rats	50 mg/kg; p.o.	Nephroprotective and anti-apoptotic effects. Reduction of DNA damage	[355]
		Metabolism syndrome-induced hyperuricemia in male Sprague–Dawley rats	0.05 and 0.1% of the diet; p.o.	Nephroprotective and anti-inflammatory effects. Inhibition of uric acid synthesis	[356]

		Glycerol- induced nephrotoxicity in male Wistar rats	15, 20, 25 mg/kg; p.o.	Nephroprotective effect by the increase in the antioxidant status and decrease of NF-kB levels	[357]
Fisetin (**21**)	*B. pentandra, H. courbaril, M. peruiferum*	Adenin-diet and UUO-induced nephropathy in male mice	50, 100 mg/kg; p.o.	Nephroprotective and anti-inflammatory effects. Reduction of the tubulointerstitial fibrosis. Inhibition of ferroptosis	[70]
		Oxonate and adenine-induced hyperuricemic nephropathy in male C57BL/6J mice	100 mg/kg; p.o.	Nephroprotective, anti-inflammatory, antifibrotic and anti-hyperuricemic effects. Modulation of IL-6/JAK2/STAT3 and TGF-β/Smad3 signaling pathways	[71]
		STZ-induced diabetic nephropathy in eNOS−/− mice	5, 10, 20 mg/kg; p.o.	Nephroprotective effect by the restoration of CDKN1B/P70S6K-mediated autophagy and inhibition of NLRP3 inflammasome	[72]
		UUO-induced nephropathy in C57BL/6 female mice	25 mg/kg; i.p.	Antifibrotic, anti-inflammatory, anti-apoptotic effects by the inhibition of phosphorylation of SMAD3 and of accumulation of profibrotic M2 macrophages in the obstructed kidneys	[73]
		Lupus nephritis model in female MRL/lpr mice	100 mg/kg; p.o.	Nephroprotective and antifibrotic effects. Reduction in the number of senescent tubular epithelial cells and myofibroblasts	[74]
		High fat diet -induced diabetic nephropathy in male C57BL/6 mice	40, 80 mg/kg; p.o.	Nephroprotective and anti-inflammatory effects by the inhibition of NF-κB and RIP3-regulated NLRP3 inflammasome	[75]
		LPS-induced sepsis and nephropathy in male C57BL/6 mice	100 mg/kg; p.o.	Nephroprotective, anti-apoptotic and anti-inflammatory effects by the inhibition of Src-mediated NF-κB, p65 and MAPK signaling pathways	[358]

		Oxonate and adenine-induced hyperuricemic nephropathy in male C57BL/6J mice	50, 100 mg/kg; p.o.	Nephroprotective and antifibrotic effects. Modulation of gut microbiota-mediated tryptophan metabolism and AHR activation	[359]
		Renal ischemia and reperfusion-induced damage in male Wistar rats	20 mg/kg; i.p.	Nephroprotective, antioxidant and anti-apoptotic effects, improvement of the mitochondrial function	[360]
		High fat diet -induced diabetic nephropathy in male C57BL/6 mice	40, 80 mg/kg; p.o.	Nephroprotective effect by the regulation of iRhom2/NF-κB and Nrf-2/HO-1 signaling pathways	[361]
		Cisplatin-induced kidney injury in male Sprague– Dawley rats	1.25, 2.5 mg/kg; i.p.	Nephroprotective, antioxidant, anti-apoptotic and anti-inflammatory effects. Inhibition of NF-κB activation	[362]
		Lead-induced induced nephrotoxicity in male Wistar rats	200 mg/kg; p.o.	Nephroprotective and anti-inflammatory effects, restoration of the antioxidant status and biochemical parameters by the upregulation of Nrf2/HO-1 signaling pathway	[363]
Formononetin (**22**)	*A. precatorius, B. nitida, M. balsamum, S. paniculatum*	UUO-induced nephropathy in male C57BL/6 mice	40 mg/kg; p.o.	Nephroprotective effect and inhibition of the ferroptosis-associated fibrosis by impeding Smad3/ATF3/SLC7A11 signaling and promoting the Nrf2 activity	[76]
		Folic acid-induced nephropathy in male C57BL/6 mice			
		Cisplatin- induced kidney injury in male Wistar rats	15, 50, 75 mg/kg; p.o.	Nephroprotective and anti-apoptotic effects by the regulation of the expression of Bax, Bcl-2 and caspase-3. Promotion of the proliferation of surviving renal tubular cells	[77]

		STZ-induced diabetic nephropathy in male Sprague–Dawley rats	20 mg/kg; p.o.	Nephroprotective and anti-apoptotic effects, reduction of mitochondrial fragmentation by the regulation of Sirt1/PGC-1α pathway	[78]
		STZ-induced diabetic nephropathy in male Sprague–Dawley rats	10, 20, 40 mg/kg; p.o.	Nephroprotective effect by the increase in the SIRT1 expression in kidney tissue	[364]
		Cisplatin- induced kidney injury in male Wistar rats	75 mg/kg; p.o.	Nephroprotective effect by the activation of the PPARα/Nrf2/HO-1/NQO1 pathway	[365]
		Methotrexate-induced kidney injury in male Wistar rats	10, 20, 40 mg/kg; p.o.	Nephroprotective and anti-inflammatory effect by the activation of Nrf2/HO-1 signaling and attenuation of oxidative damage	[366]
		5/6 nephrectomy- induced kidney injury in male Sprague– Dawley rats	30, 60 mg/kg; p.o.	Neuroprotective effect. Amelioration of muscle atrophy related to myostatin-mediated PI3K/Akt/FoxO3a pathway	[367]
		Gentamicin-induced kidney injury in male Wistar rats	60 mg/kg; p.o.	Nephroprotective, anti-inflammatory, antioxidant and anti-apoptotic effects. Induction of Nrf2 signaling	[368]
		Diabetic male C57BL/KsJ db/db mice	25, 50 mg/kg; p.o.	Nephroprotective and antifibrotic effects by the suppression of smad3 expression	[369]
Gallic acid (**23**)	*A. edulis, A. cathartica, A. occidentale, A. comosus, B. intermedia, B. verbascifolia, C. halicacabum, C. villosum, C. regium, C. antisyphiliticus, C. heliotropiifolius, C. ingrata, C. americana, D. rugosa, E. involucrata, E. punicifolia, E.*	Glyoxylic acid-induced urolithiasis in male C57BL/6 mice	25, 50 mg/kg; i.p.	Nephroprotective, antilithic, antioxidant and anti-inflammatory effects	[80]
		DnBP-induced kidney injury in male Wistar rats	50 mg/kg; p.o.	Nephroprotective and antioxidant effects	[81]
		High fat diet/STZ- induced diabetic nephropathy in male Wistar rats	25, 50 mg/kg; p.o.	Nephroprotective, anti-inflammatory and antifibrotic effects by the inhibition of the	[84]

	uniflora, E. hirta, E. prostrata, G. ulmifolia, J. gossypiifolia, J. princeps, L. pisonis, L. ferrea, L. paniculata, M. elliptica, O. monacantha, P. americana, P. hydropiperoides, P. amarus, P. niruri, P. sellowianus, P. tenellus, P. peruviana, P. crassipes, P. pilosa, P. guineense, R. rosifolius, S. molle, S. terebinthifolia, S. alata, S. reticulata, S. viarum, S. verticillata, S. mombin, S. adstringens, T. esculenta, T. cacao			renal p38 MAPK, NF-κB and TGF-β1 levels	
		Methotrexate- induced kidney injury in male Wistar rats	100 mg/kg; p.o.	Nephroprotective, antioxidant and anti-inflammatory effects	[370]
		STZ-induced diabetic nephropathy in male Sprague–Dawley rats	20, 40 mg/kg; p.o.	Nephroprotective effect. Reduction of the circulating and tissue levels of TGF-β1	[371]
		Paraquat-induced nephrotoxicity in male Wistar rats	25, 50, 100 mg/kg; p.o.	Nephroprotective, antioxidant and anti-inflammatory effects	[372]
		Methylglyoxal-induced diabetic nephropathy in male NMRI mice	30 mg/kg; p.o.	Nephroprotective and antifibrotic effects. Reduction of the endoplasmic reticulum stress	[373]
		Cisplatin-induced kidney injury in male Wistar rats	8 mg/kg; p.o.	Nephroprotective effect	[374]
		Nickel-induced kidney injury in male Swiss mice	110 mg/kg; i.p.	Nephroprotective, anti-necrotic and anti-inflammatory effects	[375]
		Renal ischemia and reperfusion-induced damage in male Wistar rats	50, 100, 200 mg/kg; p.o.	Nephroprotective effect mediated by the activation of PPAR-γ	[376]
		Arsenite-induced nephrotoxicity in male Wistar rats	10, 30 mg/kg; p.o.	Nephroprotective and antioxidant effects	[377]
		Sodium fluoride-induced kidney injury in male Wistar rats	20 mg/kg; i.p.	Nephroprotective and antioxidant effects. Restoration of kidney biochemical markers	[378]
		Diclofenac-induced kidney injury in male Wistar rats	50, 100 mg/kg; p.o.	Nephroprotective, anti-inflammatory and antioxidant effects	[379]

		Lead-induced nephrotoxicity in male Wistar rats	13.5 mg/kg; p.o.	Reduction of oxidative stress-induced kidney damage	[380]
		Cisplatin-induced kidney injury in male Wistar rats	50 mg/kg; p.o.	Nephroprotective and antioxidant effects	[381]
		Gentamicin-induced kidney injury in male Wistar rats	30 mg/kg; i.p.	Nephroprotective and antioxidant effects. Restoration of kidney biochemical markers	[382]
		Cisplatin-induced kidney injury in male Sprague– Dawley rats	20, 40 mg/kg; p.o.	Nephroprotective, anti-apoptotic, anti-inflammatory and antioxidant effects	[383]
		Aflatoxin B1-induced nephrotoxicity in male Wistar rats	20, 40 mg/kg; p.o.	Nephroprotective, anti-apoptotic, and anti-inflammatory effects	[384]
		$AlCl_3$-induced kidney injury in male Wistar rats	100 mg/kg; p.o.	Nephroprotective effect by the prevention of renal electrolyte homeostasis disruption	[385]
		Bisphenol A-induced kidney injury in male Wistar rats	50, 200 mg/kg; p.o.	Nephroprotective, anti-inflammatory and antifibrotic effects	[386]
		Glyoxal-induced renal fibrosis in male Wistar rats	100 mg/kg; p.o.	Antifibrotic effect by the suppression of collagen, MMP-2, MMP-9 and NOX mRNA expressions	[387]
		Renal ischemia and reperfusion-induced damage in male Sprague– Dawley rats	100 mg/kg; p.o.	Reduction of oxidative stress-induced kidney damage	[388]
		Cobalt- induced nephrotoxicity in male Wistar rats	120 mg/kg; p.o.	Nephroprotective effect by the reduction in NF-κB and KIM-1 expressions	[389]

Gentisic acid (**24**)	*G. ulmifolia, P. crassipes*	Ethylene glycol- induced urolithiasis in male Sprague–Dawley rats	200 mg/kg; p.o.	Antilithic effects	[85]
		Gentamicin-induced kidney injury in male Wistar rats	100 mg/kg; i.p.	Nephroprotective and anti-inflammatory effects by modulating the levels of NGAL, KIM-1, blood urea nitrogen and creatinine in serum	[390]
		STZ and NAD-induced diabetic nephropathy in male Wistar rats	100 mg/kg; p.o.	Nephroprotective and anti-inflammatory effects. Inhibition of albuminuria and of renal oxidative stress	[391]
Hederagenin (**25**)	*A. edulis, E. serpens, P. mediterranea, P. emarginatus, S. saponaria*	Renal ischemia and reperfusion-induced damage in male C57BL/6 mice	25, 50 mg/kg; p.o.	Nephroprotective and antifibrotic effects	[86]
		UUO-induced nephropathy in male C57BL/6 mice		Nephroprotective, and antifibrotic effects by the inhibition of ISG15 and JAK/STAT signaling pathways	
		Cisplatin-induced kidney injury in male c57BL/6 mice	20 mg/kg; n.d.	Nephroprotective and anti-inflammatory effects by the inhibition of Axin2 and β-catenin expressions	[87]
Hesperidin (**67**)	*A. colubrina, M. velame, V. megapotamica*	$AlCl_3$-induced kidney injury in male Wistar rats	100 mg/kg; p.o.	Nephroprotective effect by the prevention of renal electrolyte homeostasis disruption	[385]
		Arsenite-induced nephrotoxicity in male ICR mice	25 mg/kg; i.p.	Nephroprotective, anti-inflammatory and antioxidant effects	[392]
		CCl_4-induced nephrotoxicity in male Wistar rats	100 mg/kg; p.o.	Nephroprotective and anti-inflammatory effects by the suppression of NF-κB immunoexpression	[393]

		CCl_4-induced nephrotoxicity in male Wistar rats	200 mg/kg; p.o.	Nephroprotective effect by the restoration of kidney markers and antioxidant effect	[394]
		Paclitaxel-induced kidney injury in male Wistar rats	10 mg/kg; p.o.	Nephroprotective effect by the restoration of antioxidant status	[395]
		DMBA-induced kidney injury in female Sprague–Dawley rats	30 mg/kg; p.o.	Nephroprotective effect by the restoration of antioxidant status	[396]
		Gentamicin-induced kidney injury in male Wistar rats	200 mg/kg; p.o.	Nephroprotective, anti-necrotic and antioxidant effects	[397]
		Ferric nitrilotriacetate- induced nephrotoxicity in male Wistar rats	100, 200 mg/kg; p.o.	Nephroprotective, anti-apoptotic, antioxidant and anti-inflammatory effects. Inhibition of TNF-α, NF-κB, iNOS expression and upregulation of caspases	[398]
Hispidulin (**26**)	*A. precatorius, S. dulcis, S. rhombifolia*	LPS-induced kidney injury in male C57BL/6 mice	50 mg/kg; i.p.	Nephroprotective, anti-inflammatory and antioxidant effects. Reduction of tubular cell death.	[399]
		Adenine and oxonate-induced hyperuricemic nephropathy in male Sprague– Dawley rats	20, 50 mg/kg; i.p.	Nephroprotective and anti-inflammatory effects by the regulation of NF-κB activation	[400]
Hyperoside (**27**)	*A. occidentale, A. colubrina, C. racemosa, G. ulmifolia, H. bonariensis, H. leucocephala, P. americana, S. australis, T. cacao*	Diabetic male C57BL/KsJ db/db mice	50 mg/kg; p.o.	Attenuation of glomerulosclerosis by the MMP-9 pathway	[90]
		Cadmium-induced nephrotoxicity in female C57BL/6 mice	25, 50 mg/kg; p.o.	Nephroprotective and anti-inflammatory effects by the suppression of NLRP3 inflammasome activation	[91]
		High fat diet and STZ-induced diabetic nephropathy in male C57BL/6 mice	30 mg/kg; p.o.	Mitigation of renal injury and renal fibrosis	[93]

		D-galactose-induced kidney injury in male Sprague– Dawley rats	20 mg/kg; p.o.	Attenuation of renal aging and injury by the inhibition of AMPK-ULK1-mediated autophagy	[95]
		STZ-induced diabetic nephropathy in female C57BL6 mice	10, 30 mg/kg; p.o.	Nephroprotective effect by decreasing the albuminuria, renal damage and podocyte injury	[96]
		STZ-induced diabetic nephropathy in female C57BL6 mice	10, 30 mg/kg; p.o.	Nephroprotective effect by the reduction of proteinuria and improvement of antioxidant status	[97]
		Renal ischemia and reperfusion-induced damage in male C57BL/6 mice	20 mg/kg; i.p.	Nephroprotective effect by the suppression of tubular cells apoptosis, mitochondrial fission and OPA1 proteolysis	[98]
		Ethylene glycol- induced urolithiasis in male Sprague–Dawley rats	50 mg/kg; p.o.	Antilithic, antioxidant and anti-inflammatory effects	[99]
		Cisplatin-induced kidney injury in C57BL6 mice	20, 40, 80 mg/kg; i.p.	Nephroprotective and anti-inflammatory effects by the regulation of Nrf2 and HO-1 expression and NF-κB activation	[100]
		Diabetic male C57BL/KsJ db/db mice	50 mg/kg; via celiac injection	Nephroprotective effect by the regulation of ERK1/2 signaling pathway and restoration of antioxidant status	[401]
		LPS-induced kidney injury in BALB/c mice	25, 50, 100 mg/kg; i.p.	Nephroprotective and anti-inflammatory effects by the modulation of the TLR4 and NLRP3 signaling pathways.	[402]
			10 mg/kg; i.p.	Uricosuric effect	[102]

Hypophyllanthin (**28**)	*E. hirta, P. amarus, P. niruri, P. tenellus*	Oxonate and uric acid-induced hyperuricemia in male Sprague–Dawley rats	10 mg/kg; p.o.	Antihyperuricemic effect	
Isorhamnetin (**29**)	*A. colubrina, A. coriacea, B. forficata, C. sylvestris, E. biflora, E. hirta, G. integrifolia, G. ulmifolia, H. leucocephala, J. caroba, O. monacantha, P. rigida, P. hydropiperoides, S. molle, S. oleraceus, S. pseudoquina, T. cacao, V. polyanthes*	Cisplatin-induced kidney injury in male C57BL/6J mice	50 mg/kg; p.o.	Nephroprotective effect by the induction of PGC-1α-dependent reprogramming of fatty acid oxidation	[103]
		STZ-induced diabetic nephropathy in male Sprague-Dawley mice	50, 100 mg/kg; p.o.	Nephroprotective and anti-inflammatory effects. Reduction of the levels of urinary osteopontin, KIM-1 and albumin.	[104]
		Cisplatin-induced kidney injury in male C57BL/6J mice	50 mg/kg; p.o.	Nephroprotective effect by the upregulation of SLPI expression	[403]
		UUO and PKFB overexpression-induced nephropathy in male C57BL/6 mice	10 mg/kg; p.o.	Nephroprotective and antifibrotic effect. Regulation of renal glycolysis	[404]
		Hypoxanthine and oxonate-induced hyperuricemia in male Kunming mice	50, 100, 150 mg/kg; p.o.	Nephroprotective effect by the reduction of the uric acid levels and inhibition of xanthine oxidase activity	[405]
Isovitexin (**30**)	*A. grandiflorus, C. tayuya, C. erosa, C. leptophloeos, C. cajucara, E. precatoria, I. imperati, J. princeps, L. ferrea, L. pinaster, N. theifera, P. alata, P. edulis, P. pelucida, P. crassipes, S. erecta, S. alternatopinnatum, T. cacao*	LPS-induced kidney injury in male C57BL/6 mice	15 mg/kg; i.p.	Nephroprotective and anti-inflammatory, antioxidant, and anti-pyroptosis effects	[105]
		Cisplatin-induced kidney injury in female BALB/c mice	12.5, 25, 50 mg/kg; i.p.	Nephroprotective and anti-inflammatory effects by the regulation of Nrf2 and HO-1 expression and NF-κB activation	[406]
Kaempferitrin (**31**)	*B. forficata*	Normotensive and male Wistar SHR	0.3, 1 mg/kg; p.o.	Diuretic and natriuretic effects	[407]

Kaempferol (**32**)	*A. capillus-veneris, A. conyzoides, A. cathartica, A. brasiliana, A. tenella, A. occidentale, A. coriacea, A. spinescens, B. argyrophylla, B. forficata, C. halicacabum, C. sylvestris, C. pareira, C. sympodialis, C. gongonha, C. cajucara, C. ingrata, C. americana, C. racemosa, D. rugosa, D. dentatus, E. viscosa, E. giganteum, E. foetidum, E. uniflora, E. hirta, E. thymifolia, E. precatoria, G. integrifolia, G. ulmifolia, H. impetiginosus, H. crispa, H. balsamifera, H. bonariensis, I. paraguariensis, I. imperati, J. caroba, L. pisonis, M. acutifolium, M. ilicifolia, O. monacantha, P. amarus, P. brasiliensis, P. niruri, P. tenellus, P. crassipes, P. guineense, R. rosifolius, S. australis, S. terebinthifolia, S. dulcis, S. alata, S. erecta, S. rhombifolia, S. versicolor, S. guianensis, S. brasiliensis, S. cernuum, S. lycocarpum, S. paludosum, S. paniculatum, S. oleraceus, S. mombin, S. adstringens, T. aurea, T. paniculatum, T. cacao, T. grandiflorum, U. lobata, V.*	Female normotensive and Wistar SHR	0.01, 0.1, 1 mg/kg; p.o and i.p.	Absence of diuretic effect	[1]
		UUO-induced nephropathy in male Sprague– Dawley rats	30, 60, 90 mg/kg; p.o.	Antifibrotic effect	[107]
		Doxorubicin-induced kidney injury in male Balb/c mice	10 mg/kg; p.o.	Nephroprotective and antioxidant effects	[108]
		Chronic hypertension-induced kidney fibrosis in Wistar rats	10, 20, 40 mg/kg; p.o.	Reduction of blood pressure and decrease of the ECM components	[109]
		Glyoxylic acid-induced urolithiasis in male C57BL/6 mice	25, 50 mg/kg; p.o.	Antilithic, antioxidant and anti-inflammatory effects	[110]
		Glucose load-induced diabetic nephropathy in C57BL/6 mice	100, 200 mg/kg; p.o.	Nephroprotective, anti-inflammatory and antifibrotic effects	[113]
		STZ-induced diabetic nephropathy in male C57BL/6 mice	10 mg/kg; p.o.	Nephroprotective, anti-inflammatory and antifibrotic effects	[408]
		Cecal ligation and puncture injury-induced sepsis and nephropathy in ICR mice	1 mg/kg; p.o.	Nephroprotective and anti-inflammatory effects	[409]
		Diabetic male C57BL/KsJ db/db mice	50, 100 mg/kg; p.o.	Nephroprotective and anti-apoptotic effects. Induction of podocytes autophagy	[410]
		Aged Sprague Dawley rats	2, 4 mg/kg; p.o.	Anti-inflammatory and antioxidant effects	[411]
		STZ-induced diabetic nephropathy in male Wistar rats	200 mg/kg; p.o.	Nephroprotective effect by the upregulation of Nrf2/HO-1 axis	[412]

	ferruginea, V. megapotamica, X. americana	Cadmium-induced nephropathy in male Wistar rats	200 mg/kg; p.o.	Nephroprotective, anti-inflammatory and antioxidant effects	[413]
		Cisplatin-induced kidney injury in male Balb/c mice	100, 200 mg/kg; p.o.	Nephroprotective, antioxidant, anti-apoptotic and anti-inflammatory effects	[414]
		Tacrolimus-induced nephrotoxicity in male Wistar rats	10 mg/kg; p.o.	Nephroprotective and antioxidant effects. Inhibition of calcineurin B1 activity	[415]
		Mercury- induced nephrotoxicity in male Wistar rats	100 mg/kg; p.o.	Nephroprotective effects, reversal of elevated kidney markers	[416]
		Mercury- induced nephrotoxicity in male Wistar rats	100 mg/kg; p.o.	Nephroprotective effects, reversal of elevated kidney markers and lipid parameters	[417]
Liquiritigenin (**33**)	*B. pentandra, B. acutifolium*	Folic acid-induced nephropathy in male C57bL/6 mice	15 mg/kg; i.p.	Nephroprotective effect by the inhibition of VKORC1-mediated ferroptosis	[115]
		Cisplatin-induced kidney injury in male BALB/c mice	15 mg/kg; i.p.	Nephroprotective and anti-apoptotic effects, amelioration of mitochondrial dysfunction by the modulation of the NRF2 activity	[116]
		Oxonate-induced hyperuricemia in male Sprague– Dawley rats	20, 40 mg/kg; p.o.	Nephroprotective, antinecrotic and anti-inflammatory effects by the suppression of renal AQP4/NF-κB/IκBα and NLRP3 inflammasome activation	[418]
Lychnophoric acid (**68**)	*L. pinaster*	Oxonate-induced hyperuricemia in male Swiss mice	15 mg/kg; p.o.	Hypouricemic and anti-inflammatory effects	[256]
Lupeol (**69**)	*A. amazonicus, A. colubrina, C. pachystachya, C. echioides, D. unguis-cati, E. contortisiliquum, F. crocata, L.*	Oxonate-induced hyperuricemia in male Swiss mice	15 mg/kg; p.o.	Hypouricemic and anti-inflammatory effects	[256]
		Ethylene glycol- induced urolithiasis in Wistar rats	50, 100 mg/kg; p.o.	Antilithic effect	[419]

	pinaster, M. glomerata, P. rigida, P. amarus, S. dulcis, V. ferruginea	High-fat diet and sucrose-induced diabetic nephropathy in rats	25 mg/kg; p.o.	Nephroprotective effect	[420]
		Cadmium-induced nephrotoxicity in female Wistar rats	40 mg/kg; p.o.	Nephroprotective effect	[421]
		Ethylene glycol- induced urolithiasis in male Wistar rats	50 mg/kg; p.o.	Antiurolithic effect	[422]
		Pyridoxine-deficient hyperoxaluric male Wistar rats	35 mg/kg; p.o.	Antiurolithic effect	[423]
		NH_4Cl and zinc-induced urolithiasis in male albino rats	35 mg/kg; p.o.	Antiurolithic effect	[424]
Luteolin (**34**)	*A. occidentale, A. pyrifolia, B. gardneri, B. acutifolium, B. gaudichaudii, C. halicacabum, C. palmata, C. erosa, C. regium, C. erecta, C. cujete, C. antisyphiliticus, D. unguis-cati, E. prostrata, E. bonariensis, E. foetidum, E. involucrata, E. prostrata, E. precatoria, G. ulmifolia, H. impetiginosus, J. decurrens, L. paniculata, M. velame, N. theifera, O. campechianum, P. edulis, P. brasiliensis, P. niruri, P. stratiotes, P. crassipes, P. emarginatus, S. dulcis, S. fluminensis, S. oleraceus, T. aurea, T. paniculatum, T. esculenta, T. cacao, T.*	Lupus nephritis model in MRL/lpr mice	10, 40 mg/kg; p.o.	Nephroprotective, antioxidant, and anti-inflammatory effects by the regulation of NF-κB/HIF-1α signaling pathway	[118]
		Methamphetamine-induced renal injury in male C57BL/6 J mice	100 mg/kg; p.o.	Nephroprotective effect by the modulation of the GSK3β-p-Tau axis	[119]
		Cobalt- induced nephrotoxicity in male Wistar rats	100 mg/kg; p.o.	Nephroprotective effect by the reduction in NF-κB and Kim-1 expressions	[389]
		Renal ischemia and reperfusion-induced damage in male Swiss mice	100 mg/kg; n.d.	Nephroprotective, anti-apoptotic and anti-inflammatory effects, inhibition of caspase-3 expression	[425]
		Renal ischemia and reperfusion-induced damage in male Sprague– Dawley rats	50 mg/kg; p.o.	Nephroprotective effect by improving the antioxidant status and reducing the expressions of Nrf2 and miR320.	[426]

	crustacea, T. diffusa, U. baccifera, V. ferruginea, V. polyanthes, X. aromatica	LPS-induced renal injury in male ICR mice	40 mg/kg; p.o.	Nephroprotective effect by the improvement of the antioxidant status, decrease of NF-κB activation, of inflammatory and apoptosis factors	[427]
		Glycerol-induced rhabdomyolysis-related kidney injury in male Wistar rats	100, 200 mg/kg; p.o.	Nephroprotective effect by the modulation of Kim-1/ NF-κB /Nrf2 signaling pathway	[428]
		Cisplatin-induced kidney injury in male BALB/cN mice	10 mg/kg; i.p.	Nephroprotective, anti-apoptotic and anti-inflammatory effects. Suppression of oxidative/ nitrosative stress	[429]
		Cisplatin-induced kidney injury in male C57BL/6 mice	50 mg/kg; p.o.	Nephroprotective effect by the downregulation of p53-dependent apoptotic pathway in the kidney	[430]
		Doxorubicin-induced kidney injury in male Wistar rats	50, 100 mg/kg; p.o.	Nephroprotective, anti-apoptotic and anti-inflammatory effects	[431]
		Methotrexate-induced kidney injury in male Wistar rats	50 mg/kg; p.o.	Nephroprotective, anti-apoptotic, antioxidant and anti-inflammatory effects	[432]
		Colistin-induced acute kidney injury in male Wistar rats	10 mg/kg; i.p.	Nephroprotective and anti-apoptotic effects	[433]
		Mercury- induced nephrotoxicity in male Wistar rats	80 mg/kg; p.o.	Nephroprotective, anti-apoptotic and antioxidant effects by the activation of Nrf2 signaling pathway	[434]
		Lead- induced nephrotoxicity in male Wistar rats	50 mg/kg; p.o.	Nephroprotective, antioxidant, anti-inflammatory, and antiapoptotic activities by the activation of Nrf2/ARE signaling pathway	[435]

		$K_2Cr_2O_7$-induced renal injury in male Wistar rats	100, 200 mg/kg; p.o.	Nephroprotective and antioxidant effects. Modulation of KIM-1 and Nrf2 expressions	[436]
		AngII-induced renal damage in apolipoprotein E-deficient ($ApoE^{-/-}$) mice.	100 mg/kg; p.o.	Nephroprotective, anti-apoptotic and anti-inflammatory effects. Attenuation of collagen deposition	[437]
		Oxonate-induced hyperuricemia in male Kunming mice	20, 40, 100 mg/kg; p.o.	Hypouricemic and anti-inflammatory effects	[438]
Madecassoside (**35**)	*C. asiatica*	Doxorubicin-induced kidney injury in male Balb/c mice	12 mg/kg; i.p.	Nephroprotective effect	[122]
		Cisplatin-induced kidney injury in male SPF C57BL/6J mice	20 mg/kg; i.p.	Nephroprotective, anti-apoptotic and anti-inflammatory effects	[439]
Magnoflorine (**36**)	*C. glaberrima, C. pareira*	STZ-induced diabetic nephropathy in male Sprague–Dawley rats	100 mg/kg; p.o.	Nephroprotective, antifibrotic and anti-inflammatory effects	[123]
		High-fat and high-fructose diets-induced nephropathy in male C57BL/6 mice	5, 10 mg/kg; p.o.	Nephroprotective effect by the activation of Parkin/PINK1-dependent mitophagy, inhibition of NLRP3 inflammasome activation and pyroptosis	[440]
Myricetin (**38**)	*A. occidentale, A. colubrina, C. regium, D. rugosa, E. involucrata, E. punicifolia, J. princeps, L. pisonis, M. velame, P. mediterranea, P. hydropiperoides, P. niruri, P. tenellus, P. crassipes, P. emarginatus, S. paludosum, S. adstringens, T. esculenta*	Cisplatin-induced kidney injury in male Kunming mice	100 mg/kg; p.o.	Nephroprotective, antioxidant and anti-inflammatory effects. Reduction of DNA damage	[132]
		STZ-induced diabetic nephropathy in C57BL mice	200, 300 mg/kg; p.o.	Nephroprotective and antifibrotic effect by the modulation of the ROCK1/ERK/P38 pathway	[133]
		Cisplatin-induced kidney injury in female Wistar rats	3 mg/kg; i.p.	Nephroprotective, anti-inflammatory and antioxidant effects	[215]

		Lead-induced nephrotoxicity in male Wistar rats	100, 200 mg/kg; p.o.	Nephroprotective, anti-inflammatory and antioxidant effects by the regulation of the Nrf2/HO-1 signaling pathway	[363]
		STZ-induced diabetic nephropathy in C57BL mice	100 mg/kg; p.o.	Nephroprotective, antifibrotic and anti-inflammatory effects. Modulation of NF-κB signaling pathway	[441]
		Diabetic male C57BL/KsJ db/db mice	50, 100 mg/kg; p.o.	Nephroprotective, antifibrotic and anti-inflammatory effects	[442]
		Ethylene glycol- induced urolithiasis in Wistar rats	25, 50 mg/kg; p.o.	Antiurolithic, anti-inflammatory and antioxidant effects	[443]
		STZ-induced diabetic nephropathy in male Wistar rats	6 mg; i.p.	Reduction of the glomerulosclerosis and restoration of urinary markers	[444]
		Cadmium/STZ-induced kidney injury in male Wistar rats	1 mg/kg; i.p.	Nephroprotective effect by the restoration of renal biochemical markers	[445]
		Cadmium and STZ-induced kidney injury in male Wistar rats	1 mg/kg; i.p.	Nephroprotective effect by the regulation of the SREBP, TGF-β1, PPAR-α and VEGF expression levels	[446]
		Ochratoxin A-induced nephrotoxicity in male Wistar rats	100 mg/kg; p.o.	Nephroprotective effect by the regulation of the p53, PCNA and TGF-β1 levels	[447]
Morin (**37**)	*A. colubrina, M. velame, S. dulcis*	Cisplatin-induced kidney injury in male ICR mice	20, 40 mg/kg; i.p.	Nephroprotective, antioxidant and anti-autophagic effects	[128]
		Imipenem-induced kidney injury in male albino rabbits	12, 25, 50 mg/kg; p.o.	Nephroprotective effect by inhibiting the OAT3-mediated renal excretion of imipenem.	[129]
		Ethylene glycol and NH_4Cl-induced urolithiasis in Wistar rats	50, 100 mg/kg; p.o.	Nephroprotective, diuretic and antilithic effects	[130]

		Aflatoxin B1-induced nephrotoxicity in chicks	20, 40, 80 mg/kg; i.p.	Nephroprotective, anti-inflammatory and antioxidant effects. Inhibition of heterophil extracellular traps release	[448]
		Cisplatin-induced kidney injury in male BALB/cN mice	10, 20, 40 mg/kg; i.p.	Nephroprotective, anti-apoptotic, anti-inflammatory and antioxidant effects	[449]
		LPS-induced kidney injury in male CD-1 mice	50 mg/kg; p.o.	Nephroprotective, anti-inflammatory and antioxidant effects	[450]
		Ifosfamide-induced nephrotoxicity in male Sprague–Dawley rats	100, 200 mg/kg; p.o.	Nephroprotective and anti-inflammatory effects by the modulation of NF-κB/p53 and Bcl-2 signaling pathways	[451]
		Oxonate-induced hyperuricemia in male Kunming mice	20, 40, 80 mg/kg; p.o.	Uricosuric effect by the suppression of urate reabsorption and promotion of urate secretion	[452]
		Adenine-induced hyperuricemic nephropathy in male BALB/c mice	20, 40 mg/kg; p.o.	Nephroprotective, antifibrotic and anti-inflammatory effects by the inactivation of the cathepsin D signaling	[453]
		Acrylamide-induced nephrotoxicity in male Sprague–Dawley rats	100 mg/kg; p.o.	Nephroprotective, anti-apoptotic and anti-inflammatory effects by the regulation of PI3K/Akt/mTOR signaling pathway	[454]
		Doxorubicin-induced kidney injury in male Wistar rats	100 mg/kg; p.o.	Nephroprotective and anti-inflammatory effects. Modulation of the aquaporin-2 and nephrin expression	[455]
Naringenin (**39**)	*A. precatorius, A. cathartica, A. colubrina, B. pentandra, B. acutifolium, C. guianensis, C.*	Renal ischemia and reperfusion-induced damage in male C57Bl/6 mice	50 mg/kg; p.o.	Nephroprotective effect by the attenuation of pyroptosis and apoptosis through the activation of Nrf2/HO-1 signaling pathway	[134]

	cujete, C. antisyphiliticus, E. bonariensis, E. involucrata, G. ulmifolia, H. impetiginosus, L. pisonis, M. velame, P. niruri, P. crassipes, S. humboldtiana, S. terebinthifolia, S. adstringens, T. esculenta, T. cacao, V. megapotamica	STZ-induced diabetic nephropathy in male Sprague–Dawley rats	50 mg/kg; p.o.	Nephroprotective effect by the regulation of let-7a/TGFBR1 signaling.	[135]
		Oxonate and fructose-induced hyperuricemia in male Kunming mice	10, 50 mg/kg; p.o.	Uricosuric effect by the regulation of the PI3K/AKT signaling pathway. Anti-inflammatory effect by the regulation of NF-kB signaling pathway	[136]
		STZ and NAD-induced diabetic nephropathy in male Wistar rats	50, 100 mg/kg; p.o.	Nephroprotective, antioxidant and anti-apoptotic effects by the downregulation of the expression of endoplasmic reticulum stress marker proteins	[137]
		Doxorubicin-induced kidney injury in male Wistar rats	50, 100 mg/kg; p.o.	Nephroprotective and anti-inflammatory effects by the regulation of KIM-1, TNF-α, PGE-2 and NF-kB signaling pathway	[456]
		Mercury-induced nephrotoxicity in male Wistar rats	50, 100 mg/kg; p.o.	Nephroprotective and anti-apoptotic effects. Restoration of kidney antioxidant status	[457]
		Cecal ligation and puncture injury-induced sepsis and nephropathy in Wistar rats	10, 20 mg/kg; n.d.	Nephroprotective, antioxidant and anti-apoptotic effects by the decrease in Bax and increase in Bcl-2 expression. Decrease in urinary angiotensinogen levels	[458]
		L-NAME-induced hypertensive male Wistar rats	50 mg/kg; p.o.	Nephroprotective effect by the inhibition of the expressions of KIM-1, MCR and ACE and restoration of the kidney antioxidant status	[459]
		B6.MRL-Faslpr/J lupus-prone mice	50, 100 mg/kg; p.o.	Nephroprotective and antifibrotic effects. Reduction of autoimmunity by the modulation of T-cell subsets and cytokines profile	[460]

		Gentamicin-induced kidney injury in male Sprague– Dawley rats	50 mg/kg; p.o.	Nephroprotective, antioxidant and anti-inflammatory effects. Reduction of KIM-1, VEGF, iNOS, and caspase-9 expressions.	[461]
		Oxytetracycline-induced kidney injury in male Wistar rats	50 mg/kg; p.o.	Nephroprotective effect by the restoration of the kidney antioxidant status	[462]
		CCl_4-induced nephrotoxicity in male Swiss mice	50 mg/kg; p.o.	Nephroprotective effect by the restoration of the kidney antioxidant status	[463]
		Daunorubicin-induced kidney injury in male Sprague– Dawley rats	20 mg/kg; p.o.	Nephroprotective, antioxidant and anti-inflammatory effects. Attenuation of AT1R, ERK1/2-NFκB p65 signaling pathway	[464]
		2K1C surgery-induced renovascular hypertension in male Sprague– Dawley rats	200 mg/kg; p.o.	Attenuation of renal damage by normalizing the imbalance of renin-angiotensin system activation	[465]
		Lead-induced nephrotoxicity in male Sprague– Dawley rats	50 mg/kg; p.o.	Nephroprotective effect by the restoration of the kidney antioxidant status and biochemical markers	[466]
		Oxonate-induced hyperuricemia in male Wistar rats	100 mg/kg; i.p.	Uricosuric, anti-inflammatory and anti-apoptotic effects	[467]
		STZ-induced diabetic nephropathy in male Sprague–Dawley rats	5, 10 mg/kg; p.o.	Nephroprotective, anti-apoptotic, antioxidant and anti-inflammatory effects.	[468]
		Paclitaxel-induced kidney injury in male Wistar rats	10 mg/kg; p.o.	Nephroprotective effect by the restoration of the kidney antioxidant status and biochemical markers	[469]
		STZ-induced diabetic nephropathy in male BALB/cA mice	2% of the diet; p.o.	Nephroprotective, antifibrotic and anti-inflammatory effects, suppression of NF-kB activity	[470]

		Cadmium-induced nephrotoxicity in male Wistar rats	25, 50 mg/kg; p.o.	Nephroprotective effect by the restoration of the kidney antioxidant status	[471]
		Cisplatin-induced kidney injury in male Wistar rats	20 mg/kg; p.o.	Nephroprotective effect by the restoration of the kidney antioxidant status	[472]
Niranthin (**40**)	*E. hirta, P. amarus, P. niruri, P. tenellus*	Oxonate and uric acid-induced hyperuricemia in male Sprague–Dawley rats	10 mg/kg; i.p.	Uricosuric effect	[102]
Orientin (**41**)	*A. fraxinifolium, C. pachystachya, C. erosa, C. leptophloeos, E. precatoria, J. princeps, L. ferrea, N. theifera, P. alata, P. quadrangularis, P. aduncum, P. stratiotes, P. crassipes, T. cacao, V. polygama*	D-galactose-induced kidney injury in male Kunming mice	40 mg/kg; p.o.	Nephroprotective effect by the restoration of antioxidant status	[473]
		Cisplatin-induced kidney injury in male Wistar rats	40 mg/kg; p.o.	Nephroprotective and anti-inflammatory effects. Restoration of the antioxidant status	[474]
Phyllanthin (**42**)	*E. hirta, P. amarus, P. niruri*	Oxonate and uric acid-induced hyperuricemia in male Sprague–Dawley rats	5, 10, 20 mg/kg; i.p.	Uricosuric effect	[102]
			5, 10, 20 mg/kg; p.o.	Antihyperuricemic effect	[102]
Phyltetralin (**43**)	*E. hirta, P. amarus, P. niruri, P. tenellus*	Oxonate and uric acid-induced hyperuricemia in male Sprague–Dawley rats	10 mg/kg; i.p.	Uricosuric effect	[475]
			10 mg/kg; p.o.	Absence of effects	[102]

Pinocembrin (**44**)	*C. cujete, E. hirta, P. aduncum, S. leucanthum, T. diffusa*	Gentamicin-induced kidney injury in male Sprague– Dawley rats	50 mg/kg; i.p.	Nephroprotective and anti-apoptotic effects by the modulation of the Nrf2, HO-1, and NQO1 pathways	[476]
		Spontaneous polycystic kidney disease model in PCK rats	40 mg/kg; p.o.	Decrease of renal cystic area by the inhibition of renal CFTR protein expression	[477]
Procyanidin B2 (**45**)	*C. pachystachya, G. ulmifolia*	LPS-induced septic acute kidney injury in male C57BL/6 mice	100 mg/kg; p.o.	Nephroprotective effect by the decrease in renal tubular cell vacuolization and oxidative stress by Nrf2 pathway	[478]
		Diabetic male C57BL/KsJ db/db mice	30 mg/kg; p.o.	Nephroprotective effect by the inhibition of MFG-E8, phosphorylation of ERK1/2, Akt, and GSK-3β signaling pathway	[479]
		Lupus nephritis model in female MRL/lpr mice	100 mg/kg; p.o.	Nephroprotective effect by the inhibition of NLRP3 inflammasome activation	[480]
		Aflatoxin-induced nephrotoxicity in male Sprague Dawley rats	30 mg/kg; p.o.	Nephroprotective, antioxidant and anti-inflammatory effects. Modulation of Bcl-2/Bax ratio	[481]
		Spontaneous diabetic male C57BL/KsJ db/db mice	30 mg/kg; p.o.	Nephroprotective, antioxidant and antifibrotic effects	[482]
Protocatechuic acid (**46**)	*A. cathartica, C. halicacabum, C. ingrata, E. precatoria, G. ulmifolia, L. pisonis, O. monacantha, P. niruri, S. reticulata, S. adstringens, V.*	LPS-induced kidney injury in male C57B6J mice	30 mg/kg; p.o.	Nephroprotective and anti-inflammatory effects by the modulation of the IKK/NF-κB/TLR-4 pathway	[483]
		Monosodium glutamate-induced acute kidney injury in male Wistar rats	100 mg/kg; p.o.	Nephroprotective, anti-apoptotic and anti-inflammatory effects by the activation of Nrf2 pathway and suppression of NF-kB signaling pathway	[484]

	polyanthes	Doxorubicin-induced kidney injury in male Wistar rats	20 mg/kg; p.o.	Nephroprotective and anti-inflammatory effects. Restoration of the antioxidant status	[485]
		Deoxycorticosterone acetate salt-induced hypertension in male Wistar rats	50, 100, 200 mg/kg; p.o.	Nephroprotective, anti-hypertensive and anti-inflammatory effects	[486]
		Methotrexate-induced kidney injury in male Wistar rats	25, 50 mg/kg; p.o.	Nephroprotective, antioxidant, anti-apoptotic and anti-inflammatory effects	[487]
Psoralen (**47**)	*B. gaudichaudii, D. brasiliensis, D. cayapia* subsp. *asaroide*	UUO-induced nephropathy in male C57BL/6 mice	20 mg/kg; p.o.	Reduction of tubular injury, interstitial fibrosis by the modulation of NLRP3 and caspases	[488]
Quercetin (**48**)	*A. hispidum, A. capillus-veneris, A. conyzoides, A. edulis, A. cathartica, A. occidentale, A. colubrina, A. pyrifolia, A. coriacea, A. fraxinifolium, B. argyrophylla, B. laevifolia, B. forficata, B. holophylla, B. gaudichaudii, B. intermedia, B. verbascifolia, C. halicacabum, C. sylvestris, C. palmata, C. nutans, C. pareira, C. sympodialis, C. erecta, C. leptophloeos, C. antisyphiliticus, C. cajucara, C. heliotropiifolius, C. carthagenensis, C. ingrata, C.americana, C. racemosa, D. rugosa, D. unguis-cati, D.*	Oxonate-induced hyperuricemia in male Swiss mice	15 mg/kg; p.o.	Hypouricemic effect	[256]
		DnBP-induced kidney injury in male Wistar rats	50 mg/kg; p.o.	Nephroprotective and antioxidant effects	[81]
		Spontaneous diabetic male C57BL/KsJ db/db mice	25, 100 mg/kg; p.o.	Nephroprotective effect by the suppression of ferroptosis	[146]
		SARS-CoV-2 N protein-induced acute kidney injury in spontaneous diabetic db/db mice	150 mg/kg; p.o.	Nephroprotective effect by blocking SARS-CoV-2 N-Smad3-mediated cell death pathway	[147]
		UUO-induced nephropathy in male Sprague– Dawley rats	90 mg/kg; p.o.	Nephroprotective, antifibrotic, anti-apoptotic and anti-inflammatory effects	[148]
		UUO-induced nephropathy in male CD-1 mice	25 mg/kg; i.p.	Antifibrotic and inhibition of fibroblast activation effects by the mTOR and β-catenin signaling transduction inhibition	[151]

	brasiliensis, E. prostrata, E. giganteum, E. bonariensis, E. foetidum, E. involucrata, E. uniflora, E. hirta, E. thymifolia, E. precatoria, G. integrifolia, G. ulmifolia, G. viburnoides, H. impetiginosus, H. balsamifera, H. leucocephala, I. paraguariensis, I. suffruticosa, I. imperati, I. pes-caprae, J. caroba, J. decurrens, J. princeps, L. pisonis, L. pinaster, M. acutifolium, M. velame, M. elliptica, O. monacantha, P. rigida, P. edulis, P. mediterranea, P. americana, P. hydropiperoides, P. amarus, P. brasiliensis, P. niruri, P. sellowianus, P. tenellus, P. pubescens, P. peruviana, P. crassipes, P. guineense, R. rosifolius, R. viburnoides, S. humboldtiana, S. australis, S. molle, S. dulcis, S. occidentalis, S. rhombifolia, S. guianensis, S.brasiliensis, S. campestris, S. fluminensis, S. paniculatum, S. viarum, S. chilensis, S. verticillata, S. mombin, S. pseudoquina, S. adstringens, T. esculenta, T. cacao, T. grandiflorum, T. rhomboidea, T. diffusa, V. ferruginea, V. polyanthes, V. megapotamica, X. aromatica	UUO-induced nephropathy in male Sprague– Dawley rats	100 mg/kg; p.o.	Nephroprotective and antifibrotic effects	[152]
		STZ-induced diabetic nephropathy in male Wistar rats	15, 20 mg/kg; i.p.	Reduction of methylglyoxal levels and of SMP30 expression	[153]
		Spontaneous diabetic C57BL/KSJ db/db mice	50, 100, 150 mg/kg	Nephroprotective effect by the reactivation of Hippo pathway	[155]
		UUO-induced nephropathy in male Sprague– Dawley rats	100 mg/kg; p.o.	Nephroprotective, antifibrotic, anti-apoptotic and anti-inflammatory effects	[157]
		Renal ischemia and reperfusion-induced damage in C57BL/6j mice	5, 10 mg/kg; i.p.	Nephroprotective effect through AMPK-regulated autophagy signaling pathway	[159]
		STZ-induced diabetic nephropathy in male Sprague-Dawley rats	30, 60, 90 mg/kg; p.o.	Nephroprotective and antifibrotic effects	[162]
		High fat diet and STZ-induced diabetic nephropathy in male Sprague Dawley rats	300 mg/kg; p.o.	Nephroprotective, antifibrotic and anti-autophagic effects	[163]
		Oxalate-induced urolithiasis in male Sprague– Dawley rats	10 mg/kg; p.o.	Antilithic effects	[170]
		Ethylene glycol- induced urolithiasis in male Wistar rats	100 mg/L in drinking water; p.o.	Nephroprotective and antioxidant effects by the induction of PON1 activity	[278]
		Gentamicin-induced kidney injury in female Sprague Dawley rats	50 mg/kg; p.o.	Nephroprotective effect	[489]
		UUO-induced nephropathy in ICR/JCL mice	20 mg/kg; p.o.	Nephroprotective and anti-inflammatory effects	[490]

		UUO-induced nephropathy in male Sprague– Dawley rats	20 mg/kg; p.o.	Antifibrotic effect. Attenuation of cellular senescence of renal tubular epithelial cells	[491]
		Cisplatin- induced kidney injury in male C57BL/6 mice	50, 100 mg/kg; i.p.	Nephroprotective and anti-inflammatory effects	[492]
		Doxorubicin- induced kidney injury in male C57BL/6 mice	25, 50 mg/kg; i.p.	Nephroprotective, anti-inflammatory and antifibrotic effects	[493]
		Lead-induced nephrotoxicity in male Wistar rats	25, 50 mg/kg; p.o.	Nephroprotective and anti-inflammatory effects. Modulation of the MAPK and NF-κB signaling pathways	[494]
		STZ-induced diabetic nephropathy in male Sprague–Dawley rats	50, 100 mg/kg; p.o.	Anti-hyperuricemic and anti-dyslipidemic effects	[495]
		TiO_2- induced nephrotoxicity in male Wistar rats	5 mg/kg; i.p.	Nephroprotective and antioxidant effects	[496]
		Adenine-induced hyperuricemic nephropathy in male Sprague–Dawley rats	0.75, 1.5, 3 g/kg; p.o.	Nephroprotective, antifibrotic and anti-apoptotic effects	[497]
		Bisphenol A- induced nephrotoxicity in male Swiss mice	60 mg/kg; p.o.	Nephroprotective and antioxidant effects	[498]
		NaF-induced nephrotoxicity in male Wistar rats	20 mg/kg; i.p.	Nephroprotective and antioxidant effects	[499]
		Dichlorvos- induced nephrotoxicity in male Wistar rats	50 mg/kg; p.o.	Nephroprotective effect	[500]
		Pristane-induced lupus nephritis in female Balb/c mice	50 mg/kg; p.o.	Nephroprotective, anti-inflammatory and antioxidant effects	[501]

		Acrylamide- induced nephrotoxicity in male Wistar rats	50 mg/kg; p.o.	Nephroprotective and antioxidant effects. Regulation of phospholipid, energy and amino acid metabolism	[502]
		Renal ischemia and reperfusion-induced damage in male Wistar rats	50 mg/kg; p.o. and i.p.	Nephroprotective effect	[503]
		D-galactose-induced nephropathy in male Wistar rats	25, 50, 100 mg/kg; p.o.	Nephroprotective, anti-apoptotic, anti-inflammatory, and antioxidant effects	[504]
		Nephrectomy- induced glomerulosclerosis in male Sprague Dawley rats	25, 50, 100 mg/kg; p.o.	Nephroprotective effect through the regulation of TGF-β signaling pathway	[505]
		Gold nanoparticles- induced nephrotoxicity in male Wistar rats	100 mg/kg; i.p.	Nephroprotective, anti-inflammatory and antioxidant effects	[506]
		X-ray irradiation-induced nephropathy in male Sprague Dawley rats	20 mg/kg; i.p.	Nephroprotective, anti-inflammatory and antioxidant effects	[507]
		Iron-induced nephrotoxicity in male Kunming mice	1 % of the diet; p.o.	Nephroprotective and antioxidant effects	[508]
		Gibberellic acid- induced nephrotoxicity in male albino rats	50 mg/kg; i.p.	Anti-inflammatory, anti-apoptotic and antioxidant effects.	[509]
		Ethylene glycol- induced urolithiasis in male Wistar rats	10 mg/kg; p.o.	Nephroprotective and antilithic effects	[510]
		Organophosphate pesticides-induced nephrotoxicity in Wistar rats	50 mg/kg; p.o.	Nephroprotective effect by the inhibition of xanthine oxidase and PLA2 activities	[511]

		Gamma radiation- induced nephrotoxicity in Wistar rats	50 mg/kg; p.o.	Nephroprotective, anti-inflammatory and anti-apoptotic effects	[512]
		Mercury- induced nephrotoxicity in Wistar rats	250 mg/kg; p.o.	Nephroprotective effect by the reduction of KIM-1, TIMP-1, VEGF and urinary MCP-1 levels	[513]
		Hypobaric hypoxia- induced nephropathy in male Sprague–Dawley rats	50 mg/kg; p.o.	Nephroprotective effect by stabilizing the HIF-1α and reducing VEGF protein expressions and oxidative stress	[514]
		Dichloromethane- induced nephrotoxicity in male Sprague–Dawley rats	10, 20, 40 mg/kg; p.o.	Nephroprotective and antioxidant effects	[515]
		Nitrite-induced nephrotoxicity in male Wistar rats	200 mg/kg; i.p.	Nephroprotective, anti-inflammatory and anti-apoptotic effects	[516]
		Alloxan-induced diabetic nephropathy in male Swiss mice	20 mg/kg; p.o.	Nephroprotective and antioxidant effects	[517]
		Cadmium-induced nephrotoxicity in male Wistar rats	50 mg/kg; p.o.	Nephroprotective effect by the modulation of the metabolism of lipids, amino acids, and purines	[518]
		Adenine-induced hyperuricemic nephropathy in male Wistar rats	25 mg/kg; p.o.	Nephroprotective effect by the modulation of the iNOS/p38MAPK pathway	[519]
		Renal ischemia and reperfusion-induced damage in male rats	50 mg/kg; i.p.	Nephroprotective and antioxidant effects	[520]
		UUO-induced nephropathy in male Wistar rats	10, 25, 100 mg/kg; i.p.	Nephroprotective effect by the modulation of Eph/CAV-1 signaling	[521]
			50 mg/kg; i.p.		[522]

		Cadmium- induced nephrotoxicity in male Wistar rats		Nephroprotective effect by the modulation of iNOS, COX-2, and MT expressions	
		Oxonate-induced hyperuricemia in Kun-Ming mice	50, 100 mg/kg; p.o.	Uricosuric and nephroprotective effects mediated by regulating the expression levels of renal organic ion transporters and uromodulin	[523]
		Gentamicin- induced kidney injury in male Wistar rats	50 mg/kg; p.o.	Nephroprotective and antioxidant effects	[524]
		Cyclophosphamide- induced nephrotoxicity in male Wistar rats	50 mg/kg; p.o.	Nephroprotective and antioxidant effects	[525]
		Diesel exhaust particles- induced nephrotoxicity in male Wistar rats	60 mg/kg; p.o.	Nephroprotective, anti-inflammatory, anti-autophagic and antioxidant effects	[526]
		Cisplatin- induced kidney injury in male Wistar rats	50 mg/kg; p.o.	Nephroprotective and antioxidant effects	[527]
		Methotrexate- induced kidney injury in male Sprague-Dawley rats	5 mg/kg; i.p.	Nephroprotective, anti-apoptotic and antioxidant effects	[528]
		Ochratoxin-induced nephrotoxicity in Cobb broiler chicks	0.5 g/kg of the diet; p.o.	Nephroprotective, antioxidant and anti-apoptotic effects by the suppression of PI3K/AKT pathway	[529]
		Acrylamide-induced nephrotoxicity in female Wistar rats	20, 40 mg/kg; p.o.	Nephroprotective and antioxidant effects	[530]
		LPS-induced kidney injury in male C57BL/6J mice	20, 40 mg/kg; i.p.	Nephroprotective, anti-inflammatory and inhibition of macrophage M1 polarization effects by the blockade of CD38 protein	[531]

		Ferric nitrilotriacetate- induced nephrotoxicity in male Wistar rats	15 mg/kg; p.o.	Nephroprotective and antioxidant effects.	[532]
		Cisplatin- induced kidney injury in male Fischer rats	50 mg/kg; i.p.	Nephroprotective, anti-inflammatory, and anti-apoptotic effects	[533]
		Cadmium- induced nephrotoxicity in male Wistar rats	50 mg/kg; p.o.	Nephroprotective, anti-inflammatory, and antioxidant effects	[534]
		Glycerol-induced myoglobinuric nephropathy in male Wistar rats	2 mg/kg; i.p.	Nephroprotective and antioxidant effects	[535]
		UUO-induced nephropathy in male Wistar rats	50 mg/kg; i.p.	Nephroprotective and anti-inflammatory effects via COX-2 expression inhibition	[536]
		Doxorubicin- induced kidney injury in male Sprague-Dawley rats	70 mg/kg; p.o.	Nephroprotective and antifibrotic effects. Reduction of TNF-α, cleaved caspase-3, and PPARα expression	[537]
		STZ-induced diabetic nephropathy in male Sprague Dawley rats	50 mg/kg; p.o.	Nephroprotective effect by the reduction of TGF-β1 and CTGF expression	[538]
		Nano zinc oxide particles-induced nephrotoxicity in Wistar rats	200 mg/kg; p.o.	Nephroprotective and anti-inflammatory effects by the reduction of inflammatory cytokines, VEGF and NO levels	[539]
		Cadmium-induced nephrotoxicity in male Wistar rats	50 mg/kg; p.o.	Nephroprotective and anti-inflammatory effects	[540]
		STZ-induced diabetic nephropathy in male Sprague Dawley rats	10 mg/kg; p.o.	Nephroprotective and antioxidant effects	[541]

		TCDD-induced nephrotoxicity in male Wistar rats	20 mg/kg; p.o.	Nephroprotective and antioxidant effects	[542]
		Rotenone-induced nephrotoxicity in male Wistar rats	5, 10, 20 mg/kg; s.c.	Nephroprotective and antioxidant effects	[543]
		Fructose-induced hyperuricemia and kidney dysfunction in male Sprague Dawley rats	50, 100 mg/kg; p.o.	Nephroprotective and hypouricemic effects by reversing dysregulations on renal specific transporters	[544]
		Viper snake venom-induced nephrotoxicity in male Wistar rats	10 mg/kg; i.p.	Nephroprotective, antioxidant and anti-inflammatory effects	[545]
		Cyclosporine- induced kidney injury in male Sprague Dawley rats	15 mg/kg; p.o.	Nephroprotective and antioxidant effects	[546]
		Doxorubicin- induced kidney injury in male Wistar rats	50 mg/kg; p.o.	Nephroprotective, antioxidant, anti-apoptotic and anti-inflammatory effects	[547]
		High salty-diet in Dahl salt-sensitive hypertensive rats	10 mg/kg; p.o.	Downregulation of ENaC expression in the kidney	[548]
		Sphere gold nanoparticles-induced nephrotoxicity in male Wistar rats	200 mg/kg; p.o.	Nephroprotective effects	[549]
		Doxorubicin- induced kidney injury in male Wistar rats	50 mg/kg; p.o.	Nephroprotective and anti-inflammatory effects	[550]
		Cyclosporine- induced kidney injury in Wistar rats	2 mg/kg; i.p.	Nephroprotective and antioxidant effects	[551]
		Chromium- induced nephrotoxicity in Wistar rats	50 mg/kg; i.p.	Nephroprotective and antioxidant effects	[552]

		Obesity- induced nephropathy in male Sprague Dawley rats	50 mg/kg; p.o.	Nephroprotective and anti-inflammatory effects	[553]
		STZ-induced diabetic nephropathy in C57BL/6J mice	10 mg/kg; p.o.	Nephroprotective, anti-apoptotic and antioxidant effects	[554]
		Cisplatin- induced kidney injury in male Wistar rats	50 mg/kg; p.o.	Nephroprotective, antifibrotic, anti-inflammatory and antioxidant effects	[555]
		Ferric nitrilotriacetate- induced nephrotoxicity in male Wistar rats	2 mg/kg; i.p.	Nephroprotective and antioxidant effects	[556]
		Glycerol- induced nephrotoxicity in male Sprague Dawley rats	20 mg/kg; s.c.	Reduction of plasma urea levels	[557]
		Methotrexate-induced kidney injury in male Sprague–Dawley rats	50 mg/kg; p.o.	Nephroprotective, anti-apoptotic and antioxidant effects	[558]
		Renal ischemia and reperfusion-induced damage in male Sprague Dawley rats	50 mg/kg; i.p.	Nephroprotective and antioxidant effects	[559]
		Cadmium-induced nephrotoxicity in male Sprague–Dawley rats	10, 50 mg/kg; p.o.	Nephroprotective effect by the regulation of lipid and amino acid metabolism, and enhancement of the antioxidant defense system	[560]
		Acetamiprid-induced nephrotoxicity in male Wistar rats	100 mg/kg; p.o.	Nephroprotective and anti-inflammatory effects. Modulation of renal cystatin C activity, and of ICAM, NF-kB, Nrf2 and TLR4 protein expression levels	[561]
		Immune-mediated lupus nephritis in BDF1 mice	30, 80 mg/kg; p.o.	Nephroprotective effect by the inhibition of CD4 T cell activation	[562]

		STZ-induced diabetic nephropathy in male Wistar rats	30 mg/kg; p.o.	Nephroprotective effect by the enhancement of the antioxidant defense system	[563]
Rosmarinic acid (**49**)	*A. capillus-veneris, C. indica, C. nutans, D. unguis-cati, J. gossypiifolia, L. paniculata, O. campechianum, T. rhomboidea*	Male Wistar rats	3 mg/kg; p.o.	Diuretic effect. Increase in urinary excretion of Na^+, Ca^{2+} and K^+	[18]
		Cadmium- induced nephrotoxicity in Swiss mice	50 mg/kg; p.o.	Nephroprotective, antioxidant and anti-inflammatory effects. Modulation of NF-κB pathway	[174]
		STZ-induced diabetic nephropathy in male Sprague Dawley rats	7.5, 15 mg/kg; p.o.	Nephroprotective and antifibrotic effects	[175]
		UUO-induced kidney fibrosis in male C57BL/6 mice	20 mg/kg; p.o.	Nephroprotective, anti-inflammatory and antifibrotic effects by the regulation of NLRP3 inflammasome	[177]
		Male diabetic C57BLKS/J mice	100 mg/kg; n.d.	Nephroprotective and anti-inflammatory effects. Restoration of the antioxidant status	[564]
		Cisplatin- induced kidney injury in male BALB/c mice	5, 10, 20 mg/kg; n.d.	Nephroprotective and anti-inflammatory effects by the modulation of Nrf2 signaling pathway. Restoration of the antioxidant status	[565]
		Cisplatin- induced kidney injury in male Swiss mice	100 mg/kg; p.o.	Nephroprotective and anti-inflammatory effects by the modulation of NLRP3 inflammasome	[566]

		Chlorpyrifos-induced nephrotoxicity in male Wistar rats	100 mg/kg; p.o.	Nephroprotective, anti-apoptotic and anti-inflammatory effects by the upregulation of Nrf2/HO-1 signaling and SIRT1	[567]
		Cisplatin- induced kidney injury in male BALB/cN mice	2, 5 mg/kg; p.o.	Nephroprotective, anti-apoptotic and anti-inflammatory effects. Restoration of the antioxidant status	[568]
		STZ-induced diabetic nephropathy in male Wistar rats	10 mg/kg; p.o.	Nephroprotective effect by the restoration of the antioxidant status	[569]
		Methotrexate- induced kidney injury in Wistar rats	100, 200 mg/kg; p.o.	Nephroprotective, anti-necrotic, antioxidant and anti-inflammatory effects.	[570]
		Chromium- induced nephrotoxicity in male Wistar rats	25 mg/kg; p.o.	Nephroprotective, antioxidant and anti-inflammatory effects. Upregulation of Nrf2 pathway	[571]
		STZ-induced diabetic nephropathy in male Wistar rats	75 mg/kg; p.o.	Nephroprotective effect by the prevention of podocyte detachment and inhibition of urinary nephrin and podocin excretion	[572]
		Gentamicin-induced kidney injury in male Sprague Dawley rats	50, 100 mg/kg; p.o.	Nephroprotective effect by the restoration of kidney antioxidant status	[573]
		Ethylene glycol and NH_4Cl-induced urolithiasis in male SHR	3 mg/kg; p.o.	Antilithic and anti-inflammatory effects	[574]
Rutin (**50**)	*A. edulis, A. cathartica, A. brasiliana, A. tenella, A. colubrina, A. coriacea, B. laevifolia, B. floribunda, B.*	Oxonate-induced hyperuricemia in male Swiss mice	15 mg/kg; p.o.	Hypouricemic and anti-inflammatory effects	[256]
		Cadmium and EtOH- induced kidney injury in male Wistar rats	100 mg/kg; p.o.	Nephroprotective effect	[81]

	floribunda, C. sylvestris, C. pachystachya, C. palmata, C. erosa, C. erecta, C. antisyphiliticus, C. cajucara, C. carthagenensis, C. americana, D. rugosa, D. unguis-cati, E. viscosa, E. bonariensis, E. involucrata, E. uniflora, E. hirta, G. integrifolia, G. ulmifolia, H. tomentosa, H. bonariensis, H. leucocephala, I. paraguariensis, I. suffruticosa, I. imperati, J. decurrens, L. paniculata, L. pinaster, M. velame, M. elliptica, O. campechianum, P. alata, P. amarus, P. niruri, P. sellowianus, P. tenellus, P. aduncum, P. peruviana, P. crassipes, P. guineense, S. australis, S. dulcis, S. paniculata, S. rhombifolia, S. guianensis, S. brasiliensis, S. campestris, S. paniculatum, S. viarum, S. chilensis, S. oleraceus, S. mombin, S. adstringens, T. aurea, T. esculenta, T. cacao, X. americana, X. aromatica	Spontaneous diabetic kidney disease in Db/db mice	100, 200 mg/kg; p.o.	Nephroprotective effect	[178]
		UUO-induced nephropathy in male Wistar rats	100 mg/kg; p.o.	Reduction of kidney interstitial fibrosis	[180]
		Fructose-induced hyperuricemia and renal dysfunction in male Sprague Dawley rats	50, 100 mg/kg; p.o.	Nephroprotective and hypouricemic effects by reversing the dysregulations on renal specific transporters	[544]
		Acetaminophen- induced kidney injury in Wistar rats	20 mg/kg; p.o.	Nephroprotective effect	[575]
		Valproate- induced kidney injury in male Sprague Dawley rats	50, 100 mg/kg; p.o.	Nephroprotective, anti-inflammatory, and antiapoptotic effects. Reduction of renal oxidative stress and autophagy.	[576]
		LPS and D-GalN- induced kidney injury in female Wistar rats	5, 10, and 20 mg/kg; p.o.	Nephroprotective and anti-inflammatory effects	[577]
		LPS-induced kidney injury in female C57BL/6 mice	50, 200 mg/kg; p.o.	Nephroprotective, anti-apoptotic and anti-inflammatory effects	[578]
		Vancomycin-induced kidney injury in male Wistar rats	150 mg/kg; p.o.	Nephroprotective, anti-apoptotic and anti-inflammatory effects	[579]
		Malathion-induced kidney injury in male Sprague Dawley rats	50, 100 mg/kg; p.o.	Nephroprotective, anti-inflammatory, anti-apoptotic, and anti-autophagic effects	[580]
		CCl_4-induced nephrotoxicity in male ICR mice	75, 150 mg/kg; p.o.	Nephroprotective, anti-inflammatory, anti-apoptotic, and anti-autophagic effects	[581]
		Perfluorooctanoic acid-induced kidney injury in male ICR mice	20 mg/kg; p.o.	Nephroprotective and regulation of lipid metabolism effects	[582]

		Carfilzomib-induced kidney injury in male Wistar rats	10, 20, 40 mg/kg; p.o.	Nephroprotective and anti-inflammatory effects	[583]
		STZ-induced diabetic nephropathy in male Wistar rats	100 mg/kg; p.o.	Nephroprotective effect. Modulation of metalloproteinases activity	[584]
		Renal ischemia and reperfusion-induced damage in Wistar rats	1g/kg; i.p.	Nephroprotective effects	[585]
		Cisplatin-induced kidney injury in male Wistar rats	30 mg/kg; p.o.	Nephroprotective and anti-inflammatory effects	[586]
		HCBD-induced kidney injury in female Wistar rats	100, 500, 1000 mg/kg; i.p.	Nephroprotective effect	[587]
		Subtotal nephrectomy-induced male Wistar rat	15, 45 mg/kg; p.o.	Nephroprotective effect	[588]
		Oxonate-induced hyperuricemia in male Kunming mice	50, 100 mg/kg; p.o.	Nephroprotective and hypouricemic effects	[589]
		Acrylamide-induced nephrotoxicity in female Wistar rats	40 mg/kg; p.o.	Nephroprotective effect	[590]
		Deltamethrin-induced kidney injury in male Sprague Dawley rats	25, 50 mg/kg; p.o.	Nephroprotective, anti-inflammatory and anti-apoptotic effects	[591]
		Gentamicin-induced kidney injury in male Sprague Dawley rats	150 mg/kg; p.o.	Nephroprotective, anti-inflammatory, anti-apoptotic, and anti-autophagic effects	[592]
		Mercury-induced nephrotoxicity in male Sprague Dawley rats	50, 100 mg/kg; p.o.	Nephroprotective, anti-inflammatory, anti-apoptotic effects	[593]

		STZ-induced diabetic nephropathy in male Sprague Dawley rats	10, 30, 90 mg/kg; p.o.	Nephroprotective effect	[594]
		$KBrO_3$-induced nephrotoxicity in male Sprague Dawley rats	50, 70 mg/kg; p.o.	Nephroprotective effect against oxidative renal damage	[595]
		Cisplatin-induced kidney injury in male Wistar rats	75, 150 mg/kg; p.o.	Nephroprotective, anti-inflammatory, anti-apoptotic effects	[596]
		Ethylene glycol- induced urolithiasis in male Wistar rats	20 mg/kg; p.o.	Antilithic, anti-inflammatory and antioxidant effects	[597]
Schaftoside (**71**)	*A. precatorius, C. arabicus, C. spiralis, P. emarginatus, V. polygama*	Ethylene glycol and NH_4Cl - induced urolithiasis in male Sprague Dawley rats	50 mg/kg; p.o.	Antilithic effect	[598]
Scopoletin (**51**)	*B. uniflora, H. brasiliense, P. rigida, P. americana, R. rosifolius, S. rhombifolia*	Vancomycin-induced kidney injury in male Wistar rats	50 mg/kg; i.p.	Nephroprotective and anti-inflammatory effects by the modulation of IkBα/p65 NF-κB and Nrf2 / HO-1 signaling pathways	[599]
		Oxonate-induced hyperuricemia in male ICR mice	50, 100, 200 mg/kg; i.p.	Hypouricemic effect by decreasing uric acid production and uricosuric mechanism	[600]
Scutellarein (**52**)	*A. conyzoides, S. dulcis*	Renal ischemia and reperfusion-induced damage in male Sprague Dawley rats	5, 10, 20 mg/kg; p.o.	Nephroprotective, anti-inflammatory, anti-apoptotic effects	[184]

Soyasaponin Bb (**72**)	*P. emarginatus*	Male DBA/2 FG-pcy (polycystic kidney disease) mice	181 mg/100 g of the diet; p.o.	Nephroprotective and inhibition of cyst growth	[601]
Stigmasterol (**73**)	*A. conyzoides, A. colubrina, B. pentandra, B. acutifolium, C. halicacabum, C. halicacabum, C. filiformis, C. rufescens, C. cajucara, D. brasiliensis, E. prostrata, E. bonariensis, E. prostrata, G. americana, G. viburnoides, H. crispa, H. radicans, I. diffusa, L. pinaster, M. glomerata, P. pellucida, P. sellowianus, P. umbellatum, P. sagittalis, P. crassipes, S. dulcis, S. occidentalis, S. rhombifolia, S. guianensis, S. brasiliensis, S. viarum, S. verticillata, S. mombin*	Oxonate-induced hyperuricemia in male Swiss mice	15 mg/kg; p.o.	Hypouricemic and anti-inflammatory effects	[256]
		Ethylene glycol- induced urolithiasis in Wistar rats	50, 100 mg/kg; p.o.	Antilithic effect	[419]
		STZ and NAD-induced diabetic nephropathy in male Wistar rats	5, 10 mg/kg; p.o.	Nephroprotective effect	[602]
Taxifolin (**53**)	*D. brasiliensis, E. precatoria, H. tomentosa, L. pisonis*	Acrylamide-induced nephrotoxicity in male Wistar rats	50 mg/kg; p.o.	Nephroprotective, antioxidant and anti-inflammatory effects	[603]
		High fat diet and STZ-induced diabetic nephropathy in male Sprague Dawley rats	100 mg/kg; p.o.	Nephroprotective effect by the restoration of the kidney biochemical markers	[185]
		Fructose-induced metabolic syndrome in male SHR	25, 50 mg/kg; p.o.	Nephroprotective, anti-fibrotic, anti-necrotic, anti-apoptotic and anti-inflammatory effects. Modulation of PI3K/AKT signaling pathway	[186]

		UUO-induced kidney fibrosis in male C57BL6 mice	50, 100, 200 mg/kg; p.o.	Anti-fibrotic effect by the reduction of oxidative stress and Smad3 phosphorylation via Nrf2 signaling.	[187]
		Cisplatin-induced kidney injury in male Swiss mice	25, 50 mg/kg; p.o.	Nephroprotective, anti-apoptotic, antioxidant and anti-inflammatory effects. Upregulation of Nrf2/HO-1 signaling	[604]
		STZ-induced diabetic nephropathy in male rats	10, 20 mg/kg; p.o.	Nephroprotective effect by the modulation of CAV-1/NF-κB pathway	[605]
		Acetaminophen- induced nephrotoxicity in male Wistar rats	50 mg/kg; p.o.	Nephroprotective effect by the protection against oxidative damage in kidney tissue	[606]
		Cadmium- induced nephrotoxicity in male mice	25, 50 mg/kg; p.o.	Nephroprotective, anti-apoptotic, antioxidant and anti-inflammatory effects. Upregulation of Nrf2/HO-1 signaling pathway	[607]
Theobromine (**54**)	*I. paraguariensis, T. cacao*	STZ-induced diabetic nephropathy in male SHR	5 mg/kg in the drinking water; p.o.	Nephroprotective and antifibrotic effects by the activation of Sirt-1	[608]
Ursolic acid (**55**)	*A. edulis, A. cathartica, E. prostrata, E. contortisiliquum, G. viburnoides, H. radicans, J. decurrens, P. coriacea, P. amarus, P. guineense, S. rhombifolia, S. verticillata*	Diabetic male BKS db/db mice	0.3% of the diet; p.o.	Nephroprotective, antifibrotic and anti-inflammatory effects by the regulation of ARAP1/AT1R signaling pathway	[189]
		CaOx- induced kidney injury in male Sprague Dawley rats	20, 40 mg/kg; p.o.	Nephroprotective, anti-inflammatory, antifibrotic, and anti-apoptotic effects. Modulation of Nrf2/HO-1 and TLR4/NF-κB signaling pathways	[190]
		UUO-induced nephropathy in male C57BL6 mice	50, 100 mg/kg; p.o.	Suppression of EMT and ECM processes	[191]
		Alloxan-induced diabetic nephropathy in male Swiss mice	50, 100 mg/kg; p.o.	Nephroprotective and anti-necrotic effects by the inhibition of AGE formation	[609]

		Gentamicin-induced kidney injury in Wistar rats	2, 5, 10 mg/kg	Nephroprotective and antioxidant effects	[610]
		Aristolochic acid-induced nephrotoxicity in zebrafish	1, 10, 20 ppm	Nephroprotective and anti-inflammatory effects. Improvement of blood circulation	[611]
		STZ-induced diabetic nephropathy in male Wistar rats	0.2% of the diet; p.o.	Nephroprotective effect by the inhibition of NF-κB activation and inhibition of the expression of P-selectin	[612]
		Adenine-induced hyperuricemic nephropathy in male Wistar rats	30 mg/kg; p.o.	Nephroprotective and antifibrotic effects	[613]
		Renal ischemia and reperfusion-induced damage in male Sprague Dawley rats	10 mg/kg; i.p.	Nephroprotective effect, modulation of STAT3 and NF-κB activities	[614]
		Cecal ligation and puncture injury-induced sepsis and nephropathy in ICR mice	2, 20 mg/kg; i.p.	Nephroprotective and anti-inflammatory effects by the inhibition of NF-κB activation	[615]
		STZ-induced diabetic nephropathy in male Balb/cA mice	0.1, 0.2 % of the diet; p.o.	Anti-glycative effects by the reduction of renal sorbitol dehydrogenase and aldole reductase activities	[616]
		UUO-induced nephropathy in male Sprague-Dawley rats	40 mg/kg; p.o.	Nephroprotective and antifibrotic effects by the activation of Nrf2/HO-1 signaling pathway.	[617]
		CCl_4-induced nephrotoxicity in male ICR mice	25, 50 mg/kg; p.o.	Nephroprotective and anti-inflammatory effects by the modulation of STAT3 and NF-κB signaling pathways	[618]
		LPS-induced kidney injury in Balb/cA mice	100 mg/kg; p.o.	Nephroprotective and anti-inflammatory effects by the modulation of autophagy pathway	[619]

		STZ-induced diabetic nephropathy in male Wistar rats	50 mg/kg; p.o.	Nephroprotective and antioxidant effects	[620]
		STZ-induced diabetic nephropathy in male Wistar rats	25 mg/kg; p.o.	Nephroprotective and anti-inflammatory effects by the modulation of TLR4 pathway	[621]
		STZ-induced diabetic nephropathy in male C57BL mice	0.01% of the diet; p.o.	Nephroprotective and antifibrotic effects by the inhibition of STAT-3, ERK1/2 and JNK pathways activation	[622]
		STZ-induced diabetic nephropathy in male Sprague Dawley rats	35 mg/kg; p.o.	Nephroprotective and anti-inflammatory effects	[623]
		Cisplatin-induced kidney injury in male Wistar rats	10 mg/kg; p.o.	Nephroprotective, anti-apoptotic and anti-inflammatory effects	[624]
Vanillic acid (**74**)	*C. ingrata, D. unguis-cati, E. precatoria, H. brasiliense, L. pisonis, O. monacantha, P. americana, P. aduncum, P. guineense, V. cymosa*	STZ-induced diabetic nephropathy in male Sprague Dawley rats	100 mg/kg; p.o.	Nephroprotective and anti-inflammatory effects. Restoration of the antioxidant status	[625]
		STZ and NAD-induced diabetic nephropathy in male Wistar rats	25, 50, 100 mg/kg; p.o.	Nephroprotective and anti-inflammatory effects by the downregulation of NF-κB, TNF-α, COX-2 and the up-regulation of Nrf-2 proteins in renal tissue	[626]
		STZ-induced diabetic nephropathy in male Sprague Dawley rats	50, 100 mg/kg; p.o.	Nephroprotective and anti-inflammatory effects, reduction of necroptosis of renal tissue	[627]
		High fat diet-induced induced diabetic nephropathy in male Wistar rats	50 mg/kg; p.o.	Nephroprotective effect by the improvement of the kidney antioxidant status	[628]
		Cisplatin-induced kidney injury in male Wistar rats	50, 100 mg/kg; p.o.	Nephroprotective, antioxidant and anti-inflammatory effects	[629]

		Methotrexate-induced kidney injury in male Wistar rats	100 mg/kg; p.o.	Nephroprotective, anti-apoptotic, antioxidant, and anti-inflammatory effects by the reduction of caspase-3 expression and overexpression of Bcl-2 and Nrf-2	[630]
Vitexin (**56**)	*A. precatorius, A. brasiliana, A. tenella, A. colubrina, C. pachystachya, C. erosa, C. cajucara, H. balsamifera, L. ferrea, L. paniculata, L. pinaster, N. theifera, P. alata, P. edulis, P. quadrangularis, P. pelucida, P. aduncum, P. marginatum, P. stratiotes, S. dulcis, S. erecta, T. grandiflorum*	Oxonate-induced hyperuricemia in male Swiss mice	15 mg/kg; p.o.	Hypouricemic effect	[256]
		Glyoxylate-induced nephrolithiasis in male C57BL/6 mice	20 mg/kg; p.o.	Antilithic, anti-apoptotic and anti-inflammatory effects	[196]
		Renal ischemia- reperfusion and UUO-induced nephropathy in male C57BL/6J mice	30 mg/kg; p.o.	Nephroprotective, antifibrotic and anti-inflammatory effects	[631]
		Cadmium-induced nephrotoxicity in male Sprague Dawley rats	30 mg/kg; p.o.	Nephroprotective effect	[632]
Wedelolactone (**57**)	*E. prostrata*	Cisplatin-induced kidney injury in male ICR mice	20 mg/kg; p.o.	Nephroprotective and anti-inflammatory effect	[198]
		Cisplatin-induced kidney injury in male C57BL/6J mice	30 mg/kg; p.o.	Nephroprotective, anti-apoptotic and anti-inflammatory effects	[633]

2K1C 2-Kidney, 1-clip surgery, *3-MCPD* 3-Chloropropane-1, 2-diol, *ACE* Angiotensin converting enzyme, *AGE* Advanced glycation end products, *AHR* Aryl hydrocarbon receptor, *Akt* Protein kinase B, *AlCl₃* Aluminium chloride, *AMPK* AMP-activated protein kinase, *AngII* Angiotensin II, *AQP4* Aquaporin 4, *ARAP1* Angiotensin II type 1 receptor-associated protein, *AT1R* Angiotensin II type 1 receptor, *Bax* B-cell lymphoma 2-associated X protein, *Bcl-2* B-cell lymphoma-2, *CAMKII* Calcium/calmodulin-dependent protein kinase II, *CaOx* Calcium oxalate, *CAV-1* Caveolin 1, CCl_4 Carbon tetrachloride, *CDKN1B* Cyclin-dependent kinase inhibitor 1B, *CFTR* Cystic fibrosis transmembrane conductance regulator, *COX-2* Cyclooxygenase-2, CTGF Connective tissue growth factor, *DDAH/NOS* Dimethylarginine dimethylamino hydrolase/nitric oxide synthase, *DEN N*-diethylnitrosamine, *D-GalN* D-galactosamine, *DMBA* 7,12-Dimethylbenz(a)anthracene, *DnBP* Di-n-butylphthalate, *DRP1* Dynamin-related protein 1, *ECM* Extracellular matrix, *EMT* Epithelial-to-mesenchymal transition, *ENaC* Epithelial Na^+ channel, *eNOS−/−* mice eNOS homozygous knockout mice, *ERK* Extracellular signal-regulated kinase, *EtOH* Ethanol, *EZH2* Enhancer of zeste homolog 2, *Fe-NTA* Ferric nitrilotriacetate, *FOXO3a* Forkhead boxO3, *GLP-1* Glucagon-like peptide 1, *GSK-3β* Glycogen synthase kinase-3β, *HCBD* Hexachlorobutadiene, *HIF-1α* Hypoxia-inducible factor 1α, *HMGB* Renal high mobility group box, HO-1 Heme oxygenase 1, *i.d.* Intraduodenal administration, *i.p.* Intraperitoneal administration, *i.v.* Intravenous, *ICAM-1* Intracellular adhesion molecule-1, IKKβ Inhibitor of nuclear factor kappa-B kinase subunit β, IL-1β Interleukin 1β, *IL-6* Interleukin 6, *ILK* Integrin-linked kinase, *iNOS* Inducible nitric

oxide synthase, *iRhom2* Inactive rhomboid 2, *ISG15* Interferon-stimulated gene 15, *JAK* Janus kinase, *JNK* c-Jun N-terminal kinase, *$K_2Cr_2O_7$* Potassium dichromate, *$KBrO_3$* Potassium bromate, *Keap1* Kelch-like ECH-associated protein 1, *KIM-1* Kidney injury molecule-1, *L-NAME* L-N^G-Nitro arginine methyl ester, *LPS* Lipopolysaccharide, *MAPK* Mitogen-activated protein kinase, *MCP-1* Monocyte chemoattractant protein-1, MCR Mineralocorticoid receptor, *mg/kg* Milligram/kilogram, *MMP* Matrix metalloproteinase, *MT* Metallothionein, *mTOR* Mammalian target of rapamycin, *n.d.* Not described, *NAD* Nicotinamide, *NaF* Sodium fluoride, *NDMA* *N*-nitrosodimethylamine, *NF-κB* Nuclear factor-κB, *NGAL* Neutrophil gelatinase-associated lipocalin, *NH_4Cl* Ammonium chloride, *NKD2* Naked keratinocyte homolog 2, *NLRP3* NOD-like receptor family pyrin domain containing 3, *NO* Nitric oxide, *NOD-2* Nucleotide-binding oligomerization domain containing 2, *NOX* NADPH oxidase, *NQO1* NAD(P)H dehydrogenase quinone 1, *Nrf2* Erythroid 2-related factor 2, *OAT* Organic anion transporters, *OH-1* Heme oxygenase 1, *OPA1* Optic atrophy 1, *p.o.* Oral administration, *PCK* Polycystic kidney disease rat model, *PCNA* Proliferating cell nuclear antigen, *PERK* Protein kinase RNA-like endoplasmic reticulum kinase, *PGC-1α* Peroxisome proliferator-activated receptor-γ coactivator 1, *PGE-2* Prostaglandin E-2, *PHN* Passive Heymann nephritis, *PI3K* Phosphoinositide 3-Kinase, *PINK1* PTEN-induced putative kinase 1, *PLA2* Phospholipase A2, *PON1* Paraoxonase 1, *PPAR* Peroxisome proliferator-activated receptor, *RIP3* Receptor-interacting protein kinase 3, *ROCK* Rho-kinase, *s.c.* Subcutaneous administration, *SARS-CoV-2* Severe acute respiratory syndrome coronavirus 2, *SHR* Spontaneous hypertensive rats, *SIRT1* Sirtuin 1, *SLPI* Secretory leukocyte peptidase inhibitor, *SMP30* Senescence marker protein30, *Src* Steroid receptor coactivator, *SREBP* Sterol regulatory element binding protein, *STAT3* Signal transducer and activator of transcription 3, *STZ* Streptozotocin, *TCDD* 2,3,7,8-Tetrachlorodibenzo-*p*-dioxin, *TGFBR1* Transforming growth factor-β1 receptor 1, *TGF-β1* Transforming growth factor-β1, *TIMP-1* Tissue inhibitor of metalloproteinases 1, *TiO_2* Titanium dioxide, *TLR4* Toll-like receptor 4, *TNF-α* Tumor necrosis factor alpha, *ULK1* Unc-51-like kinase 1, *URAT1* Renal urate transporter 1, *UUO* Unilateral ureteral obstruction, *VEGF* Vascular endothelial growth factor, *VKORC1* Vitamin K epoxide reductase complex subunit 1, *α*-SMA *α*-smooth muscle actin, *β-NAG* N-acetyl-β-D-glucosaminidase

References

[1] Cechinel-Zanchett, C.C.; Bolda Mariano, L.N.; Boeing, T.; Costa, J.C.; Da Silva, L.M.; Bastos, J.K.; Cechinel-Filho, V.; Souza, P. Diuretic and renal protective effect of kaempferol 3-*O*-alpha-L-rhamnoside (afzelin) in normotensive and hypertensive rats. *J Nat Prod.* **2020**, *83,* 1980-1989. https://doi.org.10.1021/acs.jnatprod.0c00274.

[2] Dou, F.; Liu, Y.; Liu, L.; Wang, J.; Sun, T.; Mu, F.; Guo, Q.; Guo, C.; Jia, N.; Liu, W.; Ding, Y.; Wen, A. Aloe-emodin ameliorates renal fibrosis via inhibiting PI3K/Akt/mTOR signaling pathway *in vivo* and *in vitro*. *Rejuvenation Res.* **2019**, *22*, 218-229. https://doi.org.10.1089/rej.2018.2104.

[3] Xu, W.; Zhang, H.; Zhang, Q.; Xu, J. β-Amyrin ameliorates diabetic nephropathy in mice and regulates the miR-181b-5p/HMGB2 axis in high glucose-stimulated HK-2 cells. *Environ Toxicol.* **2022**, *Mar;37(3),* 637-649. https://doi.org.10.1002/tox.23431.

[4] Wu, Q.; Li, W.; Zhao, J.; Sun, W.; Yang, Q.; Chen, C.; Xia, P.; Zhu, J.; Zhou, Y.; Huang, G.; Yong, C.; Zheng, M.; Zhou, E.; Gao, K. Apigenin ameliorates doxorubicin-induced renal injury via inhibition of oxidative stress and inflammation. *Biomed Pharmacother.* **2021**, *137*, 111308. https://doi.org.10.1016/j.biopha.2021.111308.

[5] Sharma, A.; Sinha, S.; Shrivastava, N. Apigenin and kaempferol as novel renoprotective agent against cisplatin-induced toxicity: an *in vitro* study. *Nat Prod Res.* **2022**, *36*, 6085-6090. https://doi.org.10.1080/14786419.2022.2045603.

[6] N. Li, Z. Wang, T. Sun, Y. Lei, X. Liu, Z. Li. Apigenin alleviates renal fibroblast activation through AMPK and ERK signaling pathways *in vitro*. *Curr Pharm Biotechnol.* **2020**, *21*, 1107-1118. https://doi.org.10.2174/1389201021666200320140908.

[7] Wang, X.; Wang, W.; Wang, J.Z.; Yang, C.; Liang, C.Z. Effect of apigenin on apoptosis induced by renal ischemia/reperfusion injury in vivo and in vitro. *Ren Fail.* **2018**, *40*, 498-505. https://doi.org.10.1080/0886022X.2018.1497517.

[8] Jeon, B.J.; Yang, H.M.; Lyu, Y.S.; Pae, H.O.; Ju, S.M.; Jeon, B.H. Apigenin inhibits indoxyl sulfate-induced endoplasmic reticulum stress and anti-proliferative pathways, CHOP and IL-6/p21, in human renal proximal tubular cells. *Eur Rev Med Pharmacol Sci.* **2015**, *19*, 2303-2310.

[9] Zhang, J.; Zhao, X.; Zhu, H.; Wang, J.; Ma, J.; Gu, M. Apigenin protects against renal Tubular Epithelial Cell Injury and Oxidative Stress by High Glucose via Regulation of NF-E2-Related Factor 2 (Nrf2). *Pathway. Med Sci Monit.* **2019**, *25,* 5280-5288. https://doi.org.10.12659/MSM.915038.

[10] Li, Y.; Zhao, Z.; Luo, J.; Jiang, Y.; Li, L.; Chen, Y.; Zhang, L.; Huang, Q.; Cao, Y.; Zhou, P.; Wu, T.; Pang, J. Apigenin ameliorates hyperuricemic nephropathy by inhibiting URAT1 and GLUT9 and relieving renal fibrosis via the Wnt/β-catenin pathway. *Phytomedicine.* **2021**, *87*, 153585. https://doi.org.10.1016/j.phymed.2021.153585

[11] Huo, X.; Meng, Q.; Wang, C.; Wu, J.; Zhu, Y.; Sun, P.; Ma, X.; Sun, H.; Liu, K. Targeting renal OATs to develop renal protective agent from traditional Chinese medicines: Protective effect of apigenin against imipenem-induced nephrotoxicity. *Phytother Res.* **2020**, *34*, 2998-3010. https://doi.org.10.1002/ptr.6727.

[12] Sun, M.Y.; Ye, H.J.; Zheng, C.; Jin, Z.J.; Yuan, Y.; Weng, H.B. Astragalin ameliorates renal injury in diabetic mice by modulating mitochondrial quality control via AMPK-dependent PGC1α pathway. *Acta Pharmacol Sin.* **2023**, *44*, 1676-1686. https://doi.org.10.1038/s41401-023-01064-z.

[13] Li, X.; Wang, X.; Liu, S.; Wang, J.; Liu, X.; Zhu, Y.; Zhang, L.; Li, R. Betulinic acid attenuates T-2 toxin-induced cytotoxicity in porcine kidney cells by blocking oxidative stress and endoplasmic reticulum stress. *Comp Biochem Physiol C Toxicol Pharmacol.* **2021**, *249*, 109124. https://doi.org.10.1016/j.cbpc.2021.109124.

[14] Liu, C.M.; Qi, X.L.; Yang, Y.F.; Zhang, X.D. Betulinic acid inhibits cell proliferation and fibronectin accumulation in rat glomerular mesangial cells cultured under high glucose condition. *Biomed Pharmacother*. **2016**, *80*, 338-342. DOI: 10.1016/j.biopha.2016.02.040.

[15] Li, J.; Yang, Y.; Wei, S.; Chen, L.; Xue, L.; Tian, H.; Tao, S. Bixin protects against kidney interstitial fibrosis through promoting STAT6 degradation. *Front Cell Dev Biol.* **2020**, *8*, 576988. https://doi.org.10.3389/fcell.2020.576988.

[16] Li, J.; Yang, Y.; Wei, S.; Chen, L.; Xue, L.; Tian, H.; Tao, S. Bixin confers prevention against ureteral obstruction-caused renal interstitial fibrosis through activation of the nuclear factor erythroid-2-related factor2 pathway in mice. *J Agric Food Chem.* **2020**, *68*, 8321-8329. https://doi.org.10.1021/acs.jafc.0c03674.

[17] Jeon, G.Y.; Nam, M.H.; Lee, K.W. Inhibitory effect of caffeic acid on advanced glycation end product-induced renal fibrosis in vitro: A potential therapeutic target. *J Food Sci.* **2021**, *86*, 579-586. https://doi.org.10.1111/1750-3841.15588.

[18] Moser, J.C.; Cechinel-Zanchett, C.C.; Mariano, L.N.B.; Boeing, T.; Silva, L.M.; Souza, P. Diuretic, natriuretic and Ca^{2+}-sparing effects induced by rosmarinic and caffeic acids in rats. *Rev. Bras. Farmacogn.* **2020**, *30*, 588–592. https://doi.org/10.1007/s43450-020-00075-9.

[19] Peerapen, P.; Thongboonkerd, V. Caffeine prevents kidney stone formation by translocation of apical surface annexin A1 crystal-binding protein into cytoplasm: in vitro evidence. *Sci Rep*. **2016**, *6*, 38536. DOI: 10.1038/srep38536.

[20] Nilnumkhum, A.; Kanlaya, R.; Yoodee, S.; Thongboonkerd, V. Caffeine inhibits hypoxia-induced renal fibroblast activation by antioxidant mechanism. *Cell Adh Migr.* **2019**, *13*, 260-272. https://doi.org.10.1080/19336918.2019.1638691.

[21] Kanlaya, R.; Subkod, C.; Nanthawuttiphan, S.; Thongboonkerd. V. Caffeine prevents oxalate-induced epithelial-mesenchymal transition of renal tubular cells by its anti-oxidative property through activation of Nrf2 signaling and suppression of Snail1 transcription factor. *Biomed Pharmacother.* **2021**, *141*, 111870. https://doi.org.10.1016/j.biopha.2021.111870.

[22] Li, X.; Wu, G.; Shang, P.; Bao, J.; Lu, J.; Yue, Z. Anti-nephrolithic potential of catechin in melamine-related urolithiasis via the inhibition of ROS, apoptosis, phospho-p38, and osteopontin in male Sprague-Dawley rats. *Free Radic Res.* **2015**, *49*, 1249-1258. https://doi.org.10.3109/10715762.2015.1061187.

[23] Zhai, W.; Zheng, J.; Yao, X.; Peng, B.; Liu, M.; Huang, J.; Wang, G.; Xu, Y. Catechin prevents the calcium oxalate monohydrate induced renal calcium crystallization in NRK-52E cells and the ethylene glycol induced renal stone formation in rat. *BMC Complement Altern Med.* **2013**, *13*, 228. https://doi.org.10.1186/1472-6882-13-228.

[24] Zhu, J.; Fu, M.; Gao, J.; Dai, G.; Guan, Q.; Du, C. Upregulation of thioredoxin reductase 1 expression by flavan-3-ols protects human kidney proximal tubular cells from hypoxia-induced Cell Death. *Antioxidants (Basel)*. **2022**, *11*, 1399. DOI: 10.3390/antiox11071399.

[25] Alim, Z.; Kilinç, N.; Şengül, B.; Beydemir, Ş. Inhibition behaviours of some phenolic acids on rat kidney aldose reductase enzyme: an in vitro study. *J Enzyme Inhib Med Chem.* **2017**, *32*, 277-284. https://doi.org.10.1080/14756366.2016.1250752.

[26] Zhang, J.; Hou, A.; Dong, J.; Zheng, S.; Yu, H.; Wang, X.; Jiang, H.; Yang, L. Screening out key compounds of Glechomae Herba for antiurolithic activity and quality control based on spectrum-effect relationships coupled with UPLC-QDA. *Biomed Pharmacother.* **2022**, *149*, 112829. https://doi.org.10.1016/j.biopha.2022.112829.

[27] Lin, C.H.; Tseng, H.F.; Hsieh, P.C.; Chiu, V.; Lin, T.Y.; Lan, C.C.; Tzeng, I.S.; Chao, H.N.; Hsu, C.C.; Kuo, C.Y. Nephroprotective role of chrysophanol in hypoxia/reoxygenation-induced renal cell damage via apoptosis, ER stress, and ferroptosis. *Biomedicines.* **2021**, *9*, 1283. https://doi.org.10.3390/biomedicines9091283.

[28] Gu, M.; Zhou, Y.; Liao, N.; Wei, Q.; Bai, Z.; Bao, N.; Zhu, Y.; Zhang, H.; Gao, L.; Cheng, X. Chrysophanol, a main anthraquinone from Rheum palmatum L. (rhubarb), protects against renal fibrosis by suppressing NKD2/NF-κB pathway. *Phytomedicine*. **2022**, *105*, 154381. https://doi.org.10.1016/j.bcp.2020.114079.

[29] Ma, S.; Xu, H.; Huang, W.; Gao, Y.; Zhou, H.; Li, X.; Zhang, W. Chrysophanol relieves cisplatin-induced nephrotoxicity *via* concomitant inhibition of oxidative stress, apoptosis, and inflammation. *Front Physiol.* **2021**, *12*, 706359. https://doi.org.10.3389/fphys.2021.706359.

[30] Dou, F.; Ding, Y.; Wang, C.; Duan, J.; Wang, W.; Xu, H.; Zhao, X.; Wang, J.; Wen, A. Chrysophanol ameliorates renal interstitial fibrosis by inhibiting the TGF-β/Smad signaling pathway. *Biochem Pharmacol.* **2020**, *180,* 114079. https://doi.org.10.1016/j.bcp.2020.114079.

[31] Gong, T. Protective effect of chrysophanol on canine renal cell injury induced by canine parvovirus. *Cell Mol Biol*. **2023**, *69*, 186-191. https://doi.org.10.14715/cmb/2023.69.5.29.

[32] Du, Y.W.; Li, X.K.; Wang, T.T.; Zhou, L.; Li, H.R.; Feng, L.; Ma, H.; Liu, H.B. Cyanidin-3-glucoside inhibits ferroptosis in renal tubular cells after ischemia/reperfusion injury via the AMPK pathway. *Mol Med.* **2023**, *29*, 42. https://doi.org.10.1186/s10020-023-00642-5.

[33] Xiong, Y.; Jian, J.; Yu, H.; Wu, J.; Mao, H.; Feng, R.; Wang, L.; Jian, Y.; Liu, X. Cyanidin-3-O-glucoside plays a protective role against renal ischemia/ reperfusion injury via the JAK/STAT pathway. *Acta Cir Bras*. **2023**, *38*, e381023. https://doi.org.10.1590/acb381023.

[34] Gao, S.; Chen, T.; Choi, M.Y.; Liang, Y.; Xue, J.; Wong, Y.S. Cyanidin reverses cisplatin-induced apoptosis in HK-2 proximal tubular cells through inhibition of ROS-mediated DNA damage and modulation of the ERK and AKT pathways. *Cancer Lett.* **2013**, *333*, 36-46. https://doi.org.10.1016/j.canlet.2012.12.029.

[35] Wei, J.; Wu, H.; Zhang, H.; Li, F.; Chen, S.; Hou, B.; Shi, Y.; Zhao, L.; Duan, H. Anthocyanins inhibit high glucose-induced renal tubular cell apoptosis caused by oxidative stress in db/db mice. *Int J Mol Med.* **2018**, *41*, 1608-1618. https://doi.org.10.3892/ijmm.2018.3378.

[36] Du, C.; Shi, Y.; Ren, Y.; Wu, H.; Yao, F.; Wei, J.; Wu, M.; Hou, Y.; Duan, H. Anthocyanins inhibit high-glucose-induced cholesterol accumulation and inflammation by activating LXRα pathway in HK-2 cells. *Drug Des Devel Ther.* **2015**, *9*, 5099-5113. https://doi.org.10.2147/DDDT.S90201.
[37] Bankoglu, E.E.; Broscheit, J.; Arnaudov, T.; Roewer, N.; Stopper, H. Protective effects of tricetinidin against oxidative stress inducers in rat kidney cells: A comparison with delphinidin and standard antioxidants. *Food Chem Toxicol.* **2018**, *121*, 549-557. https://doi.org.10.1016/j.fct.2018.09.058.
[38] Song, S.E.; Jo, H.J.; Kim, Y.W.; Cho, Y.J.; Kim, J.R.; Park, S.Y. Delphinidin prevents high glucose-induced cell proliferation and collagen synthesis by inhibition of NOX-1 and mitochondrial superoxide in mesangial cells. *J Pharmacol Sci.* **2016**, *130*, 235-243. https://doi.org.10.1016/j.jphs.2016.03.005.
[39] Li, M.T.; Liu, L.L.; Zhou, Q.; Huang, L.X.; Shi, Y.X.; Hou, J.B.; Lu, H.T.; Yu, B.; Chen, W.; Guo, ZY. *Phyllanthus niruri* L. exerts protective effects against the calcium oxalate-induced renal injury *via* ellagic acid. *Front Pharmacol.* **2022**, *13*, 891788. https://doi.org.10.3389/fphar.2022.891788.
[40] Chen, J.H.; Wu, P.T.; Chyau, C.C.; Wu, P.H.; Lin, H.H. The nephroprotective effects of *Hibiscus sabdariffa* leaf and ellagic acid *in vitro* and *in vivo* models of hyperuricemic nephropathy. *J Agric Food Chem*. **2023**, *71*, 382-397. https://doi.org.10.1021/acs.jafc.2c05720.
[41] Rao, A.R.; Veeresham, C.; Asres, K. In vitro and in vivo inhibitory activities of four Indian medicinal plant extracts and their major components on rat aldose reductase and generation of advanced glycation endproducts. *Phytother Res*. **2013**, *27*, 753-760. https://doi.org.10.1002/ptr.4786.
[42] Feng, L.; Lin, Z.; Tang, Z.; Zhu, L.; Xu, S.; Tan, X.; Wang, X.; Mai, J.; Tan, Q. Emodin improves renal fibrosis in chronic kidney disease by regulating mitochondrial homeostasis through the mediation of peroxisome proliferator-activated receptor-gamma coactivator-1 alpha (PGC-1α). *Eur J Histochem.* **2024**, *68*, 3917. https://doi.org.10.4081/ejh.2024.3917.
[43] Xu, L.; Gao, J.; Huang, D.; Lin, P.; Yao, D.; Yang, F.; Zhang, Y.; Yang, X.; Wu, M.; Ye, C. Emodin ameliorates tubulointerstitial fibrosis in obstructed kidneys by inhibiting EZH2. *Biochem Biophys Res Commun*. **2021**, *534*, 279-285. https://doi.org.10.1016/j.bbrc.2020.11.094.
[44] Liu, H.; Chen, W.D.; Hu, Y.L.; Yang, W.Q.; Hu, T.T.; Wang, H.L.; Zhang, Y.M. Emodin ameliorates high glucose-induced podocyte apoptosis via regulating AMPK/mTOR-mediated autophagy signaling pathway. *Chin J Integr Med.* **2023**, *29*, 801-808. https://doi.org.10.1007/s11655-022-3540-9.
[45] Wang, Y.; Liu, Q.; Cai, J.; Wu, P.; Wang, D.; Shi, Y.; Huyan, T.; Su, J.; Li, X.; Wang, Q.; Wang, H.; Zhang, F.; Bae, O.N.; Tie, L. Emodin prevents renal ischemia-reperfusion injury via suppression of CAMKII/DRP1-mediated mitochondrial fission. *Eur J Pharmacol.* **2022**, *916*, 174603. https://doi.org.10.1016/j.ejphar.2021.174603.
[46] Lu, H.; Xie, D.; Qu, B.; Li, M.; He, Y.; Liu, W. Emodin prevents renal ischemia-reperfusion injury via suppression of p53-mediated cell apoptosis based on network pharmacology. *Heliyon*. **2023**, *9*, e15682. https://doi.org.10.1016/j.heliyon.2023.e15682.
[47] Yuan, X.; Dai, B.; Yang, L.; Lin, B.; Lin, E.; Pan, Y. Emodin ameliorates renal injury in BXSB mice by modulating TNF-α/ICAM-1. *Biosci Rep.* **2020**, *40*, BSR20202551. https://doi.org.10.1042/BSR20202551.
[48] Waly, M.I.; Ali, B.H.; Al-Lawati, I.; Nemmar, A. Protective effects of emodin against cisplatin-induced oxidative stress in cultured human kidney (HEK 293) cells. *J Appl Toxicol.* **2013**, *33*,626-630. https://doi.org.10.1002/jat.1788.
[49] Yang, F.; Deng, L.; Li, J.; Chen, M.; Liu, Y.; Hu, Y.; Zhong, W. Emodin retarded renal fibrosis through regulating HGF and TGFβ-Smad signaling pathway. *Drug Des Devel Ther.* **2020**, *14*, 3567-3575. https://doi.org.10.2147/DDDT.S245847.
[50] Liu, W.; Gu, R.; Lou, Y.; He, C.; Zhang, Q.; Li, D. Emodin-induced autophagic cell death hinders epithelial-mesenchymal transition via regulation of BMP-7/TGF-β1 in renal fibrosis. *J Pharmacol Sci.* **2021**, *146*, 216-225. https://doi.org.10.1016/j.jphs.2021.03.009.

[51] Tian, N.; Gao, Y.; Wang, X.; Wu, X.; Zou, D.; Zhu, Z.; Han, Z.; Wang, T.; Shi, Y. Emodin mitigates podocytes apoptosis induced by endoplasmic reticulum stress through the inhibition of the PERK pathway in diabetic nephropathy. *Drug Des Devel Ther.* **2018**, *12*, 2195-2211. https://doi.org.10.2147/DDDT.S167405.

[52] Chen, T.; Zheng, L.Y.; Xiao, W.; Gui, D.; Wang, X.; Wang, N. Emodin ameliorates high glucose induced-podocyte epithelial-mesenchymal transition in-vitro and in-vivo. *Cell Physiol Biochem*. **2015**, *35*, 1425-1236. https://doi.org.10.1159/000373963.

[53] Liu, H.; Gu, L.B.; Tu, Y.; Hu, H.; Huang, Y.R.; Sun, W. Emodin ameliorates cisplatin-induced apoptosis of rat renal tubular cells in vitro by activating autophagy. *Acta Pharmacol Sin.* **2016**, *37*, 235-245. https://doi.org.10.1038/aps.2015.114.

[54] Chen, H.; Huang, R.S.; Yu, X.X.; Ye, Q.; Pan, L.L.; Shao, G.J.; Pan, J. Emodin protects against oxidative stress and apoptosis in HK-2 renal tubular epithelial cells after hypoxia/reoxygenation. *Exp Ther Med*. **2017**, *14*, 447-452. https://doi.org.10.3892/etm.2017.4473.

[55] Chan, T.M.; Leung, J.K.; Tsang, R.C.; Liu, Z.H.; Li, L.S.; Yung, S. Emodin ameliorates glucose-induced matrix synthesis in human peritoneal mesothelial cells. *Kidney Int.* **2003**, *64*, 519-533. https://doi.org.10.1046/j.1523-1755.2003.00113.x.

[56] Li, X.; Liu,W.; Wang, Q.; Liu, P.; Deng, Y.; Lan, T.; Zhang, X.; Qiu, B.; Ning, H.; Huang, H. Emodin suppresses cell proliferation and fibronectin expression via p38MAPK pathway in rat mesangial cells cultured under high glucose. *Mol Cell Endocrinol.* **2009**, *307*, 157-162. https://doi.org.10.1016/j.mce.2009.03.006.

[57] Yang, J.; Zeng, Z.; Wu, T.; Yang, Z.; Liu, B.; Lan, T. Emodin attenuates high glucose-induced TGF-β1 and fibronectin expression in mesangial cells through inhibition of NF-κB pathway. *Exp Cell Res*. **2013**, *319*, 3182-3189. https://doi.org.10.1016/j.yexcr.2013.10.006.

[58] Tanabe, K.; Tamura, Y.; Lanaspa, M.A.; Miyazaki, M.; Suzuki, N.; Sato, W.; Maeshima, Y.; Schreiner, G.F.; Villarreal, F.J.; Johnson, R.J.; Nakagawa, T. Epicatechin limits renal injury by mitochondrial protection in cisplatin nephropathy. *Am J Physiol Renal Physiol*. **2012**, *303*, F1264-74. https://doi.org.10.1152/ajprenal.00227.2012.

[59] Álvarez-Cilleros, D.; Martín, M.Á.; Ramos, S. (-)-Epicatechin and the Colonic 2,3-Dihydroxybenzoic Acid Metabolite Regulate Glucose Uptake, Glucose Production, and Improve Insulin Signaling in Renal NRK-52E Cells. *Mol Nutr Food Res.* **2018**, *62*. https://doi.org.10.1002/mnfr.201700470.

[60] Yokozawa, T.; Rhyu, D.Y.; Cho, E.J.; Aoyagi, K. Protective activity of (-)-epicatechin 3-O-gallate against peroxynitrite-mediated renal damage. *Free Radic Res.* **2003**, *37*, 561-571. https://doi.org.10.1080/1071576031000083134.

[61] Costa, S.; Utan, A.; Cervellati, R.; Speroni, E.; Guerra, M.C. Catechins: natural free-radical scavengers against ochratoxin A-induced cell damage in a pig kidney cell line (LLC-PK1). *Food Chem Toxicol.* **2007**, *45*, 1910-1917. https://doi.org.10.1016/j.fct.2007.04.008.

[62] Liang, Y.J.; Jian, J.H.; Liu, Y.C.; Juang, S.J.; Shyu, K.G.; Lai, L.P.; Wang, B.W.; Leu, J.G. Advanced glycation end products-induced apoptosis attenuated by PPARdelta activation and epigallocatechin gallate through NF-kappaB pathway in human embryonic kidney cells and human mesangial cells. *Diabetes Metab Res Rev.* **2010**, *26*, 406-416. https://doi.org.10.1002/dmrr.1100.

[63] Pan, H.; Chen, J.; Shen, K.; Wang, X.; Wang, P.; Fu, G.; Meng, H.; Wang, Y.; Jin, B. Mitochondrial modulation by Epigallocatechin 3-Gallate ameliorates cisplatin induced renal injury through decreasing oxidative/nitrative stress, inflammation and NF-kB in mice. *PLoS One.* **2015**, *10*, e0124775. https://doi.org.10.1371/journal.pone.0124775.

[64] Chang, S.N.; Haroon, M.; Dey, D.K.; Kang. S.C. Rhabdomyolysis-induced acute kidney injury and concomitant apoptosis induction via ROS-mediated ER stress is efficaciously counteracted by epigallocatechin gallate. *J Nutr Biochem*. **2022**, *110*, 109134. https://doi.org.10.1016/j.jnutbio.2022.109134.

[65] Wang, Y.; Liu, N.; Bian, X.; Sun, G.; Du, F.; Wang, B.; Su, X.; Li, D. Epigallocatechin-3-gallate reduces tubular cell apoptosis in mice with ureteral obstruction. *J Surg Res*. **2015**, *197*, 145-154. https://doi.org.10.1016/j.jss.2015.03.034.
[66] Chowdhury, S.; Ghosh, S.; Das, A.K.; Sil, P.C. Ferulic acid protects hyperglycemia-induced kidney damage by regulating oxidative insult, inflammation and autophagy. *Front Pharmacol*. **2019**, *10*, 27. https://doi.org.10.3389/fphar.2019.00027.
[67] Niu, L.; Wang, L.; He, X.; Fan, Q.; Chen, M.; Qiao, Y.; Huang, H.; Lai, S.; Wan, Q.; Zhang, Z.; He, M.; He, H. Renoprotective effects of ferulic acid mediated by AMPKα1 against lipopolysaccharide-induced damage. *Int Immunopharmacol.* **2023**, *115*, 109703. https://doi.org.10.1016/j.intimp.2023.109703.
[68] Wei, M.G.; Sun, W.; He, W.M.; Ni, L.; Yang, Y.Y. Ferulic acid attenuates TGF-β1-induced renal cellular fibrosis in NRK-52E cells by inhibiting Smad/ILK/Snail pathway. *Evid Based Complement Alternat Med*. (2015), 619720. https://doi.org.10.1155/2015/619720.
[69] Bunel, V.; Antoine, M.H.; Nortier, J.; Duez, P.; Stévigny, C. Nephroprotective effects of ferulic acid, Z-ligustilide and E-ligustilide isolated from Angelica sinensis against cisplatin toxicity in vitro. *Toxicol In Vitro.* **2015**, *29*, 458-467. https://doi.org.10.1016/j.tiv.2014.12.017.
[70] Wang, B.; Yang, L.N.; Yang, L.T.; Liang, Y.; Guo, F.; Fu, P.; Ma, L. Fisetin ameliorates fibrotic kidney disease in mice via inhibiting ACSL4-mediated tubular ferroptosis. *Acta Pharmacol Sin*. **2024**, *45*, 150-165. https://doi.org.10.1038/s41401-023-01156-w.
[71] Ren, Q.; Tao, S.; Guo, F.; Wang, B.; Yang, L.; Ma, L.; Fu, P. Natural flavonol fisetin attenuated hyperuricemic nephropathy via inhibiting IL-6/JAK2/STAT3 and TGF-β/SMAD3 signaling. *Phytomedicine*. **2021**, *87*, 153552. https://doi.org.10.1016/j.phymed.2021.153552.
[72] Dong, W.; Jia, C.; Li, J.; Zhou, Y.; Luo, Y.; Liu, J.; Zhao, Z.; Zhang, J.; Lin, S.; Chen, Y. Fisetin attenuates diabetic nephropathy-induced podocyte injury by inhibiting NLRP3 inflammasome. *Front Pharmacol*. **2022**, *13*, 783706. https://doi.org.10.3389/fphar.2022.783706.
[73] Ju, H.Y.; Kim, J.; Han, S.J. The flavonoid fisetin ameliorates renal fibrosis by inhibiting SMAD3 phosphorylation, oxidative damage, and inflammation in ureteral obstructed kidney in mice. *Kidney Res Clin Pract.* **2023**, *42*, 325-339. https://doi.org.10.23876/j.krcp.22.034.
[74] Ijima, S.; Saito, Y.; Nagaoka, K.; Yamamoto, S.; Sato, T.; Miura, N.; Iwamoto, T.; Miyajima, M.; Chikenji, T.S. Fisetin reduces the senescent tubular epithelial cell burden and also inhibits proliferative fibroblasts in murine lupus nephritis. *Front Immunol*. **2022**, *13*, 960601. https://doi.org.10.3389/fimmu.2022.960601.
[75] Ge, C.; Xu, M.; Qin, Y.; Gu, T.; Lou, D.; Li, Q.; Hu, L.; Nie, X.; Wang, M.; Tan, J. Fisetin supplementation prevents high fat diet-induced diabetic nephropathy by repressing insulin resistance and RIP3-regulated inflammation. *Food Funct*. **2019**, *10*, 2970-2985. https://doi.org.10.1039/c8fo01653d.
[76] Zhu, B.; Ni, Y.; Gong, Y.; Kang, X.; Guo, H.; Liu, X.; Li, J.; Wang, L. Formononetin ameliorates ferroptosis-associated fibrosis in renal tubular epithelial cells and in mice with chronic kidney disease by suppressing the Smad3/ATF3/SLC7A11 signaling. *Life Sci*. **2023**, *315*, 121331. https://doi.org.10.1016/j.lfs.2022.121331.
[77] Huang, D.; Wang, C.; Duan, Y.; Meng, Q.; Liu, Z.; Huo, X.; Sun, H.; Ma, X.; Liu, K. Targeting Oct2 and P53: Formononetin prevents cisplatin-induced acute kidney injury. *Toxicol Appl Pharmacol*. **2017**, *326*, 15-24. https://doi.org.10.1016/j.taap.2017.04.013.
[78] Huang, Q.; Chen, H.; Yin, K.; Shen, Y.; Lin, K.; Guo, X.; Zhang, X.; Wang, N.; Xin, W.; Xu, Y.; Gui, D. Formononetin attenuates renal tubular injury and mitochondrial damage in diabetic nephropathy partly via regulating Sirt1/PGC-1α pathway. *Front. Pharmacol*. **2022**, *13*, 901234. https://doi.org.10.3389/fphar.2022.901234.
[79] Lee, H.; Lee, D.; Kang, K.S.; Song, J.H.; Choi, Y.K. Inhibition of intracellular ROS accumulation by formononetin attenuates cisplatin-mediated apoptosis in LLC-PK1 cells. *Int. J. Mol. Sci.* **2018**, *19*, 813. https://doi.org.10.3390/ijms19030813.

[80] Zhou, D.; Wu, Y.; Yan, H.; Shen, T.; Li, S.; Gong, J.; Li, G.; Mai, H.; Wang, D.; Tan, X. Gallic acid ameliorates calcium oxalate crystal-induced renal injury via upregulation of Nrf2/HO-1 in the mouse model of stone formation. *Phytomedicine*. **2022**, *106*, 154429. https://doi.org.10.1016/j.phymed.2022.154429.
[81] Abarikwu, S.O.; Simple, G.; Onuoha, S.C.; Mokwenye, I.; Ayogu, J.F. Evaluation of the protective effects of quercetin and gallic acid against oxidative toxicity in rat's kidney and HEK-293 cells. *Toxicol Rep*. **2020**, *7*, 955-962. https://doi.org.10.1016/j.toxrep.2020.07.015.
[82] Hashemzaei, M.; Tabrizian, K.; Alizadeh, Z.; Pasandideh, S.; Rezaee, R.; Mamoulakis, C.; Tsatsakis, A.; Skaperda, Z.; Kouretas, D.; Shahraki, J. Resveratrol, curcumin and gallic acid attenuate glyoxal-induced damage to rat renal cells. *Toxicol Rep*. **2020**, *7*, 1571-1577. https://doi.org.10.1016/j.toxrep.2020.11.008.
[83] Cechinel-Zanchett, C.C.; Bolda Mariano, L.N.; Schlickmann, F.; Cechinel-Filho, V.; Souza, P. In vitro effects of 2 bioactive compounds, gallic acid and methyl gallate, on urolithiasis. *Actas Urol Esp (Engl Ed).* **2021**, *11*, S0210-4806(21)00093-0. https://doi.org.10.1016/j.acuro.2020.09.016.
[84] Ahad, A.; Ahsan, H.; Mujeeb, M.; Siddiqui, W.A. Gallic acid ameliorates renal functions by inhibiting the activation of p38 MAPK in experimentally induced type 2 diabetic rats and cultured rat proximal tubular epithelial cells. *Chem Biol Interact.* **2015**, *240*, 292-303. https://doi.org.10.1016/j.cbi.2015.08.026.
[85] Zhang, X.Z.; Lei, X.X.; Jiang, Y.L.; Zhao, L.M.; Zou, C.Y.; Bai, Y.J.; Li, Y.X.; Wang, R.; Li, Q.J.; Chen, Q.Z.; Fan, M.H.; Song, Y.T.; Zhang, W.Q.; Zhang, Y.; Li-Ling, J.; Xie, H.Q. Application of metabolomics in urolithiasis: the discovery and usage of succinate. *Signal Transduct Target Ther.* **2023**, *8*, 41. https://doi.org.10.1038/s41392-023-01311-z.
[86] Jia, J.; Xu, L.H.; Deng, C.; Zhong, X.; Xie, K.H.; Han, R.Y.; Su, H.W.; Tan, R.Z.; Wang, L. Hederagenin ameliorates renal fibrosis in chronic kidney disease through blocking ISG15 regulated JAK/STAT signaling. *Int Immunopharmacol.* **2023**, *118*, 110122. https://doi.org.10.1016/j.intimp.2023.110122.
[87] Xie, K.H.; Liu, X.H.; Jia, J.; Zhong, X.; Han, R.Y.; Tan, R.Z.; Wang, L. Hederagenin ameliorates cisplatin-induced acute kidney injury via inhibiting long non-coding RNA A330074k22Rik/Axin2/β-catenin signalling pathway. *Int Immunopharmacol.* **2022**, *112*, 109247. https://doi.org.10.1016/j.intimp.2022.109247.
[88] Yang, W.; He, L. The protective effect of hederagenin on renal fibrosis by targeting muscarinic acetylcholine receptor. *Bioengineered.* **2022**, *13*, 8689-8698. https://doi.org.10.1080/21655979.2022.2054596.
[89] Wu, F.; Li, S.; Zhang, N.; Huang, W.; Li, X.; Wang, M.; Bai, D.; Han, B. Hispidulin alleviates high-glucose-induced podocyte injury by regulating protective autophagy. *Biomed Pharmacother.* **2018**, *104*, 307-314. https://doi.org.10.1016/j.biopha.2018.05.017.
[90] Zhang, K.; Li, M.; Yin, K.; Wang, M.; Dong, Q.; Miao, Z.; Guan, Y.; Wu, Q.; Zhou, Y. Hyperoside mediates protection from diabetes kidney disease by regulating ROS-ERK signaling pathway and pyroptosis. *Phytother Res.* **2023**, *37*, 5871-5882. https://doi.org.10.1002/ptr.7993.
[91] Li, Z.; Liao, W.; Yin, X.; Liu, L.; Zhao, Z.; Lu, X.; Xu, F.; Lin, X.; Chen, Y.; Song, J.; He, Z.; Wei, Q.; Wu, W.; Wu, Y.; Yang, X. Hyperoside attenuates Cd-induced kidney injury via inhibiting NLRP3 inflammasome activation and ROS/MAPK/NF-κB signaling pathway in vivo and in vitro. *Food Chem Toxicol.* **2023**, *172*, 113601. https://doi.org.10.1016/j.fct.2023.113601.
[92] Chen, Y.; Ye, L.; Li, W.; Li, D.; Li, F. Hyperoside protects human kidney-2 cells against oxidative damage induced by oxalic acid. *Mol Med Rep.* **2018**, *18*, 486-494. https://doi.org.10.3892/mmr.2018.8948.
[93] Zhou, J.; Zhang, S.; Sun, X.; Lou, Y.; Bao, J.; Yu, J. Hyperoside ameliorates diabetic nephropathy induced by STZ via targeting the miR-499-5p/APC axis. *J Pharmacol Sci.* **2021**, *146*, 10-20. https://doi.org.10.1016/j.jphs.2021.02.005.

[94] Zhou, J.; Zhang, S.; Sun, X.; Lou, Y.; Yu, J. Hyperoside protects HK-2 cells against high glucose-induced apoptosis and inflammation *via* the miR-499a-5p/NRIP1 pathway. *Pathol Oncol Res.* **2021**, *27*, 629829. https://doi.org.10.3389/pore.2021.629829.
[95] Liu, B.; Tu, Y.; He, W.; Liu, Y.; Wu, W.; Fang, Q.; Tang, H.; Tang, R.; Wan, Z.; Sun, W.; Wan, Y. Hyperoside attenuates renal aging and injury induced by D-galactose via inhibiting AMPK-ULK1 signaling-mediated autophagy. *Aging*. **2018**, *10*, 4197-4212. https://doi.org. 10.18632/aging.101723.
[96] Zhang, L.; He, S.; Yang, F.; Yu, H.; Xie, W.; Dai, Q.; Zhang, D.; Liu, X.; Zhou, S.; Zhang, K. Hyperoside ameliorates glomerulosclerosis in diabetic nephropathy by downregulating miR-21. *Can J Physiol Pharmacol.* **2016**, *94*, 1249-1256. https://doi.org.10.1139/cjpp-2016-0066.
[97] An, X.; Zhang, L.; Yuan, Y.; Wang, B.; Yao, Q.; Li, L.; Zhang, J.; He, M.; Zhang, J. Hyperoside pre-treatment prevents glomerular basement membrane damage in diabetic nephropathy by inhibiting podocyte heparanase expression. *Sci Rep.* **2017**, *7*, 6413. https://doi.org.10.1038/s41598-017-06844-2.
[98] Wu, L.; Li, Q.; Liu, S.; An, X.; Huang, Z.; Zhang, B.; Yuan, Y.; Xing, C. Protective effect of hyperoside against renal ischemia-reperfusion injury *via* modulating mitochondrial fission, oxidative stress, and apoptosis. *Free Radic Res.* **2019**, *53*, 727-736. https://doi.org.10.1080/10715762.2019.1623883.
[99] Tian, H.; Liang, Q.; Shi, Z.; Zhao, H. Hyperoside ameliorates renal tubular oxidative damage and calcium oxalate deposition in rats through AMPK/Nrf2 Signaling Axis. *J Renin Angiotensin Aldosterone Syst.* **2023**, 5445548. https://doi.org.10.1155/2023/5445548.
[100] Chao, C.S.; Tsai, C.S.; Chang, Y.P.; Chen, J.M.; Chin, H.K.; Yang, S.C. Hyperin inhibits nuclear factor kappa B and activates nuclear factor E2-related factor-2 signaling pathways in cisplatin-induced acute kidney injury in mice. *Int Immunopharmacol.* **2016**, *40*, 517-523. https://doi.org.10.1016/j.intimp.2016.09.020.
[101] Zhou, L.; An, X.F.; Teng, S.C.; Liu, J.S.; Shang, W.B.; Zhang, A.H.; Yuan, Y.G.; Yu, J.Y. Pretreatment with the total flavone glycosides of Flos Abelmoschus manihot and hyperoside prevents glomerular podocyte apoptosis in streptozotocin-induced diabetic nephropathy. *J Med Food.* **2012**, *15*, 461-8. https://doi.org.10.1089/jmf.2011.1921.
[102] V. Murugaiyah, K.L. Chan, Antihyperuricemic lignans from the leaves of *Phyllanthus niruri*, Planta Med. 72 (2006) 1262-1267. https://doi.org.10.1055/s-2006-947224.
[103] Wang, L.; Xie, Y.; Xiao, B.; He, X.; Ying, G.; Zha, H.; Yang, C.; Jin, X.; Li, G.; Ping, L.; Wang, J.; Weng, Q. Isorhamnetin alleviates cisplatin-induced acute kidney injury via enhancing fatty acid oxidation. *Free Radic Biol Med.* **2024**, *212*, 22-33. https://doi.org.10.1016/j.freeradbiomed.2023.12.010.
[104] Qiu, S.; Sun, G.; Zhang, Y.; Li, X.; Wang, R. Involvement of the NF-κB signaling pathway in the renoprotective effects of isorhamnetin in a type 2 diabetic rat model. *Biomed Rep.* **2016**, *4*, 628-634. https://doi.org.10.3892/br.2016.636.
[105] Tseng, C.Y.; Yu, P.R.; Hsu, C.C.; Lin, H.H.; Chen, J.H. The effect of isovitexin on lipopolysaccharide-induced renal injury and inflammation by induction of protective autophagy. *Food Chem Toxicol.* **2023**, *172*, 113581. https://doi.org.10.1016/j.fct.2022.113581.
[106] Jiang, W.; Wang, R.; Liu, D.; Zuo, M.; Zhao, C.; Zhang, T.; Li, W. Protective effects of kaempferitrin on advanced glycation end products induce mesangial cell apoptosis and oxidative stress. *Int J Mol Sci.* **2018**, *19*, 3334. https://doi.org.10.3390/ijms19113334.
[107] Ji, X.; Cao, J.; Zhang, L.; Zhang, Z.; Shuai, W.; Yin, W. Kaempferol protects renal fibrosis through activating the BMP-7-Smad1/5 signaling pathway. *Biol Pharm Bull.* **2020**, *43*, 533-539. https://doi.org.10.1248/bpb.b19-01010.

[108] Wu, Q.; Chen, J.; Zheng, X.; Song, J.; Yin, L.; Guo, H.; Chen, Q.; Liu, Y.; Ma, Q.; Zhang, H.; Yang, Q. Kaempferol attenuates doxorubicin-induced renal tubular injury by inhibiting ROS/ASK1-mediated activation of the MAPK signaling pathway. *Biomed Pharmacother.* **2023**, *157*, 114087. https://doi.org.10.1016/j.biopha.2022.114087.

[109] Guan, Y.; Quan, D.; Chen, K.; Kang, L.; Yang, D.; Wu, H.; Yan, M.; Wu, S.; Lv, L.; Zhang, G. Kaempferol inhibits renal fibrosis by suppression of the sonic hedgehog signaling pathway. *Phytomedicine.* **2023**, *108*, 154246. https://doi.org.10.1016/j.phymed.2022.154246.

[110] Yuan, P.; Sun, X.; Liu, X.; Hutterer, G.; Pummer, K.; Hager, B.; Ye, Z.; Chen, Z. Kaempferol alleviates calcium oxalate crystal-induced renal injury and crystal deposition via regulation of the AR/NOX2 signaling pathway. *Phytomedicine.* **2021**, *86*, 153555. https://doi.org.10.1016/j.phymed.2021.153555.

[111] Zhang, N.; Zhao, S.; Hong, J.; Li, W.; Wang, X. Protective effects of kaempferol on D-ribose-induced mesangial cell injury. *Oxid Med Cell Longev.* **2019**, 7564207. https://doi.org.10.1155/2019/7564207.

[112] Sharma, D.; Gondaliya, P.; Tiwari, V.; Kalia, K. Kaempferol attenuates diabetic nephropathy by inhibiting RhoA/Rho-kinase mediated inflammatory signalling. *Biomed Pharmacother.* **2019**, *109*, 1610-1619. https://doi.org.10.1016/j.biopha.2018.10.195.

[113] Sharma, D.; Kumar Tekade, R.; Kalia, K. Kaempferol in ameliorating diabetes-induced fibrosis and renal damage: An in vitro and in vivo study in diabetic nephropathy mice model. *Phytomedicine.* **2020**, *76*, 153235. https://doi.org.10.1016/j.phymed.2020.153235.

[114] Li, Y.; Zheng, D.; Shen, D.; Zhang, X.; Zhao, X.; Liao, H. Protective effects of two safflower derived compounds, kaempferol and hydroxysafflor yellow A, on hyperglycaemic stress-induced podocyte apoptosis via modulating of macrophage M1/M2 polarization. *J Immunol Res.* **2020**, 2462039. https://doi.org.10.1155/2020/2462039.

[115] Guo, R.Z.; Li, J.; Pan, S.K.; Hu, M.Y.; Lv, L.X.; Feng, Q.; Qiao, Y.J.; Duan, J.Y.; Liu, D.W.; Liu, Z.S. Liquiritigenin, an active ingredient of liquorice, alleviates acute kidney injury by VKORC1-mediated ferroptosis inhibition. *Am J Chin Med.* **2024**, *28*, 1-20. https://doi.org.10.1142/S0192415X24500599.

[116] Zhou, M.; Dai, Y.; Ma, Y.; Yan, Y.; Hua, M.; Gao, Q.; Geng, X.; Zhou, Q. Protective effects of liquiritigenin against cisplatin-induced nephrotoxicity via NRF2/SIRT3-mediated improvement of mitochondrial function. *Molecules.* **2022**, *27*, 3823. https://doi.org.10.3390/molecules27123823.

[117] Zhu, X.; Shi, J.; Li, H. Liquiritigenin attenuates high glucose-induced mesangial matrix accumulation, oxidative stress, and inflammation by suppression of the NF-κB and NLRP3 inflammasome pathways. *Biomed Pharmacother.* **2018**, *106*, 976-982. https://doi.org. 10.1016/j.biopha.2018.07.045.

[118] Ding, T.; Yi, T.; Li, Y.; Zhang, W.; Wang, X.; Liu, J.; Fan, Y.; Ji, J.; Xu, L. Luteolin attenuates lupus nephritis by regulating macrophage oxidative stress via HIF-1α pathway. *Eur J Pharmacol.* **2023**, *953*, 175823. https://doi.org.10.1016/j.ejphar.2023.175823.

[119] Ding, J.; Wang, Y.; Wang, Z.; Hu, S.; Li, Z.; Le, C.; Huang, J.; Xu, X.; Huang, J.; Qiu, P. Luteolin ameliorates methamphetamine-induced podocyte pathology by inhibiting Tau phosphorylation in mice. *Evid Based Complement Alternat Med.* **2022**, 5909926. https://doi.org.10.1155/2022/5909926.

[120] Yu, Q.; Zhang, M.; Qian, L.; Wen, D.; Wu, G. Luteolin attenuates high glucose-induced podocyte injury via suppressing NLRP3 inflammasome pathway. *Life Sci.* **2019**, *225*, 1-7. https://doi.org.10.1016/j.lfs.2019.03.073.

[121] Liu, M.; Cheng, C.; Li, X.; Zhou, S.; Hua, J.; Huang, J.; Li, Y.; Yang, K.; Zhang, P.; Zhang,Y.; Tian, J. Luteolin alleviates ochratoxin A induced oxidative stress by regulating Nrf2 and HIF-1α pathways in NRK-52E rat kidney cells. *Food Chem Toxicol.* **2020**, *141*, 111436. https://doi.org.10.1016/j.fct.2020.111436.

[122] Su, Z.; Ye, J.; Qin, Z.; Ding, X. Protective effects of madecassoside against Doxorubicin induced nephrotoxicity in vivo and in vitro. *Sci Rep.* **2015**, *5*, 18314. https://doi.org.10.1038/srep18314.

[123] Chang, L.; Wang, Q.; Ju, J.; Li, Y.; Cai, Q.; Hao, L.; Zhou, Y. Magnoflorine ameliorates inflammation and fibrosis in rats with diabetic nephropathy by mediating the stability of lysine-specific demethylase 3A. *Front Physiol.* **2020**, *11*, 580406. https://doi.org.10.3389/fphys.2020.580406.

[124] Yokozawa, T.; Satoh, A.; Cho, E.J.; Kashiwada, Y.; Ikeshiro, Y. Protective role of Coptidis Rhizoma alkaloids against peroxynitrite-induced damage to renal tubular epithelial cells. *J Pharm Pharmacol.* **2005**, *57*, 367-374. https://doi.org.10.1211/0022357055470.

[125] P.K. Issac, M. Velayutham, A. Guru, G. Sudhakaran, R. Pachaiappan, J. Arockiaraj. Protective effect of morin by targeting mitochondrial reactive oxygen species induced by hydrogen peroxide demonstrated at a molecular level in MDCK epithelial cells. *Mol Biol Rep.* **2022**, *49*, 4269-4279. https://doi.org.10.1007/s11033-022-07261-z.

[126] Mo, J.S.; Choi, D.; Han, Y.R.; Kim, N.; Jeong, H.S. Morin has protective potential against ER stress induced apoptosis in renal proximal tubular HK-2 cells. *Biomed Pharmacother.* **2019**, 112, 108659. https://doi.org.10.1016/j.biopha.2019.108659.

[127] Ke, Y.Q.; Liu, C.; Hao, J.B.; Lu, L.; Lu, N.N.; Wu, Z.K.; Zhu, S.S.; Chen, X.L. Morin inhibits cell proliferation and fibronectin accumulation in rat glomerular mesangial cells cultured under high glucose condition. *Biomed Pharmacother.* **2016**, *84*, 622-627. https://doi.org.10.1016/j.biopha.2016.09.088.

[128] Singh, M.P.; Chauhan, A.K.; Kang, S.C. Morin hydrate ameliorates cisplatin-induced ER stress, inflammation and autophagy in HEK-293 cells and mice kidney via PARP-1 regulation. *Int Immunopharmacol.* **2018**, *56*, 156-167. https://doi.org.10.1016/j.intimp.2018.01.031.

[129] Lim, S.C.; Im, Y.B.; Bae, C.S.; Han, S.I.; Kim, S.E.; Han, H.K. Protective effect of morin on the imipenem-induced nephrotoxicity in rabbits. *Arch Pharm Res.* **2008**, *31*, 1060-1065. https://doi.org.10.1007/s12272-001-1270-x.

[130] Ponugoti, M.; Guntupalli, C.; Malothu, N. Morin hydrate mitigates calcium oxalate urolithiasis by inhibiting oxalate synthesis and modulating crystal formation. *Urolithiasis.* **2024**, *52*, 127. https://doi.org.10.1007/s00240-024-01628-6.

[131] Zeng, L.H.; Fung, K.P.; Wu, T.W. Morin hydrate protects cultured rat glomerular mesangial cells against oxyradical damage. *Life Sci.* **1994**, *55*, PL351-7. https://doi.org.10.1016/0024-3205(94)00760-8.

[132] Qi, X.; Wang, J.; Fei, F.; Gao, X.; Wu, X.; Shi, D.; Guo, C. Myricetin-loaded nanomicelles protect against cisplatin-induced acute kidney injury by inhibiting the DNA damage-cGAS-STING signaling pathway. *Mol Pharm.* **2023**, *20*, 136-146. https://doi.org. 10.1021/acs.molpharmaceut.2c00520.

[133] Yuan, N.; Diao, J.; Dong, J.; Yan, Y.; Chen, Y.; Yan, S.; Liu, C.; He, Z.; He, J.; Zhang, C.; Wang, H.; Wang, M.; He, F.; Xiao, W. Targeting ROCK1 in diabetic kidney disease: Unraveling mesangial fibrosis mechanisms and introducing myricetin as a novel antagonist. *Biomed Pharmacother.* **2024**, *171*, 116208. https://doi.org.10.1016/j.biopha.2024.116208.

[134] Zhang, B.; Wan, S.; Liu, H.; Qiu, Q.; Chen, H.; Chen, Z.; Wang, L.; Liu, X. Naringenin alleviates renal ischemia reperfusion injury by suppressing ER stress-induced pyroptosis and apoptosis through activating Nrf2/HO-1 signaling pathway. *Oxid Med Cell Longev.* **2022**, 5992436. https://doi.org.10.1155/2022/5992436.

[135] Yan, N.; Wen, L.; Peng, R.; Li, H.; Liu, H.; Peng, H.; Sun, Y.; Wu, T.; Chen, L.; Duan, Q.; Sun, Y.; Zhou, Q.; Wei, L.; Zhang, Z. Naringenin ameliorated kidney injury through Let-7a/TGFBR1 signaling in diabetic nephropathy. *J Diabetes Res.* **2016**, 8738760. https://doi.org.10.1155/2016/8738760.

[136] Yang, B.; Xin, M.; Liang, S.; Huang, Y.; Li, J.; Wang, C.; Liu, C.; Song, X.; Sun, J.; Sun W. Naringenin ameliorates hyperuricemia by regulating renal uric acid excretion via the PI3K/AKT signaling pathway and renal inflammation through the NF-κB signaling pathway. *J Agric Food Chem*. **2023**, *71*, 1434-1446. https://doi.org. 10.1021/acs.jafc.2c01513.

[137] Khan, M.F.; Mathur, A.; Pandey, V.K.; Kakkar, P. Naringenin alleviates hyperglycemia-induced renal toxicity by regulating activating transcription factor 4-C/EBP homologous protein mediated apoptosis. *J Cell Commun Signal.* **2022**, *16*, 271-291. https://doi.org.10.1007/s12079-021-00644-0.
[138] Kong, Z.L.; Che, K.; Hu, J.X.; Chen, Y.; Wang, Y.Y.; Wang, X.; Lü, W.S.; Wang, Y.G.; Chi, J.W. Orientin protects podocytes from high glucose induced apoptosis through mitophagy. *Chem Biodivers.* **2020**, *17*, e1900647. https://doi.org.10.1002/cbdv.201900647.
[139] Zhang, Y.; Yu, C.; Feng, Y. Pinocembrin ameliorates lipopolysaccharide-induced HK-2 cell apoptosis and inflammation by regulating endoplasmic reticulum stress. *Exp Ther Med.* **2022**, *24*, 513. https://doi.org.10.3892/etm.2022.11440.
[140] Yin, J.; Wang, K.; Zhu, X.; Lu, G.; Jin, D.; Qiu, J.; Zhou, F. Procyanidin B2 suppresses hyperglycemia-induced renal mesangial cell dysfunction by modulating CAV-1-dependent signaling. *Exp Ther Med.* **2022**, *24*, 496. https://doi.org.10.3892/etm.2022.11423.
[141] Li, D.; Zhao, T.; Meng, J.; Jing, Y.; Jia, F.; He, P. Procyanidin B2 inhibits high glucose-induced epithelial-mesenchymal transition in HK-2 human renal proximal tubular epithelial cells. *Mol Med Rep.* **2015**, *12*, 8148-8154. https://doi.org.10.3892/mmr.2015.4445.
[142] Bao, L.; Cai, X.; Zhang, Z.; Li, Y. Grape seed procyanidin B2 ameliorates mitochondrial dysfunction and inhibits apoptosis via the AMP-activated protein kinase-silent mating type information regulation 2 homologue 1-PPARγ co-activator-1α axis in rat mesangial cells under high-dose glucosamine. *Br J Nutr.* **2015**, *113*, 35-44. https://doi.org.10.1017/S000711451400347X.
[143] Cai, X.; Bao, L.; Ren, J.; Li, Y.; Zhang, Z. Grape seed procyanidin B2 protects podocytes from high glucose-induced mitochondrial dysfunction and apoptosis via the AMPK-SIRT1-PGC-1α axis in vitro. *Food Funct*. **2016**, *7*, 805-815. https://doi.org.10.1039/c5fo01062d.
[144] Ma, Y.; Chen, F.; Yang, S.; Chen, B.; Shi, J. Protocatechuic acid ameliorates high glucose-induced extracellular matrix accumulation in diabetic nephropathy. *Biomed Pharmacother.* **2018**, *98*, 18-22. https://doi.org.10.1016/j.biopha.2017.12.032.
[145] Lin, Y.; Zhong, L.; Li, H.; Xu, Y.; Li, X.; Zheng, D. Psoralen alleviates high glucose-induced HK-2 cell injury by inhibition of Smad 2 signaling via upregulation of microRNA 874. *BMC Pharmacol Toxicol.* **2020**, *21*, 52. https://doi.org.10.1186/s40360-020-00434-1.
[146] Feng, Q.; Yang, Y.; Qiao, Y.; Zheng, Y.; Yu, X.; Liu, F.; Wang, H.; Zheng, B.; Pan, S.; Ren, K.; Liu, D.; Liu, Z. Quercetin ameliorates diabetic kidney injury by inhibiting ferroptosis via activating Nrf2/HO-1 signaling pathway. *Am J Chin Med.* **2023**, *51*, 997-1018. https://doi.org.10.1142/S0192415X23500465.
[147] Wu, W.; Wang, W.; Liang, L.; Chen, J.; Wei, B.; Huang, X.R.; Wang, X.; Yu, X.; Lan, H.Y. Treatment with quercetin inhibits SARS-CoV-2 N protein-induced acute kidney injury by blocking Smad3-dependent G1 cell-cycle arrest. *Mol Ther.* **2023**, *31*, 344-361. https://doi.org. 10.1016/j.ymthe.2022.12.002.
[148] Wang, Q.; Wang, F.; Li, X.; Ma, Z.; Jiang, D. Quercetin inhibits the amphiregulin/EGFR signaling-mediated renal tubular epithelial-mesenchymal transition and renal fibrosis in obstructive nephropathy. *Phytother Res.* **2023**, *37*, 111-123. https://doi.org.10.1002/ptr.7599.
[149] Luo, M.; Liu, Z.; Hu, Z.; He, Q. Quercetin improves contrast-induced acute kidney injury through the HIF-1α/lncRNA NEAT1/HMGB1 pathway. *Pharm Biol.* **2022**, *60*, 889-898. https://doi.org.10.1080/13880209.2022.2058558.
[150] Widowati, W.; Prahastuti, S.; Tjokropranoto, R.; Onggowidjaja, P.; Kusuma, H.S.W.; Afifah, E.; Arumwardana, S.; Maulana, M.A.; Rizal, R. Quercetin prevents chronic kidney disease on mesangial cells model by regulating inflammation, oxidative stress, and TGF-β1/SMADs pathway. *PeerJ.* **2022**, *10*, e13257. https://doi.org.10.7717/peerj.13257.
[151] Ren, J.; Li, J.; Liu, X.; Feng, Y.; Gui, Y.; Yang, J.; He, W.; Dai, C. Quercetin inhibits fibroblast activation and kidney fibrosis involving the suppression of mammalian target of rapamycin and β-catenin signaling. *Sci Rep.* **2016**, *6*, 23968. https://doi.org.10.1038/srep23968.

[152] Liu, X.; Sun, N.; Mo, N.; Lu, S.; Song, E.; Ren, C.; Li, Z. Quercetin inhibits kidney fibrosis and the epithelial to mesenchymal transition of the renal tubular system involving suppression of the Sonic Hedgehog signaling pathway. *Food Funct.* **2019**, *10*, 3782-3797. https://doi.org.10.1039/c9fo00373h.
[153] Abharzanjani, F.; Hemmati, M. Protective effects of quercetin and resveratrol on aging markers in kidney under high glucose condition: in vivo and in vitro analysis. *Mol Biol Rep.* **2021**, *48*, 5435-5442. https://doi.org.10.1007/s11033-021-06550-3.
[154] Guo, S.; Sun, J.; Zhuang, Y. Quercetin alleviates lipopolysaccharide-induced inflammatory responses by up-regulation miR-124 in human renal tubular epithelial cell line HK-2. *Biofactors.* **2020**, *46*, 402-410. https://doi.org.10.1002/biof.1596.
[155] Lei, D.; Chengcheng, L.; Xuan, Q.; Yibing, C.; Lei, W.; Hao, Y.; Xizhi, L.; Yuan, L.; Xiaoxing,Y.; Qian, L. Quercetin inhibited mesangial cell proliferation of early diabetic nephropathy through the Hippo pathway. *Pharmacol Res.* **2019**, *146*, 104320. https://doi.org.10.1016/j.phrs.2019.104320.
[156] Andreucci, M.; Faga, T.; Pisani, A.; Serra, R.; Russo, D.; De Sarro, G.; Michael, A. Quercetin protects against radiocontrast medium toxicity in human renal proximal tubular cells. *J Cell Physiol.* **2018**, *233*, 4116-4125. https://doi.org.10.1002/jcp.26213.
[157] Ma, Z.; Wang, F.; Wang, H.; Sun, T.; Sun, W.; Xu, Q. Quercetin ameliorates renal tubulointerstitial transformation and renal fibrosis by regulating NLRP3 in obstructive nephropathy. *Minerva Med.* **2023**, *114*, 530-532. https://doi.org.10.23736/S0026-4806.22.08104-6.
[158] Chen, S.; Xu, T.; Xu, A.; Chu, J.; Luo, D.; Shi, G.; Li, S. Quercetin alleviates zearalenone-induced apoptosis and necroptosis of porcine renal epithelial cells by inhibiting CaSR/CaMKII signaling pathway. *Food Chem Toxicol.* **2023**, *182*, 114184. https://doi.org.10.1016/j.fct.2023.114184.
[159] Chen, B.L;. Wang, L.T.; Huang, K.H.; Wang, C.C.; Chiang, C.K.; Liu, S.H. Quercetin attenuates renal ischemia/reperfusion injury via an activation of AMP-activated protein kinase-regulated autophagy pathway. *J Nutr Biochem.* **2014**, *25*, 1226-1234. https://doi.org.10.1016/j.jnutbio.2014.05.013.
[160] Canuto, J.A.; Sampaio, T.L.; Silva, M.E.; Costa, M.D.R.; Almeida, I. M.; Magalhães, E.P.; Marinho, M.M.; Marinho, E. S.; Menezes, R.R.P.P.B.; Martins, A.M.C. Protective Effect of Quercetin on Renal Tubular Cells and the Involvement with the Renin-Angiotensin-Aldosterone Axis. Braz. Arch. Biol. *Technol.* **2021**, *64*, e21210202. https://doi.org/10.1590/1678-4324-2021210202
[161] Chen, P.; Shi, Q.; Xu, X.; Wang, Y.; Chen, W.; Wang, H. Quercetin suppresses NF-κB and MCP-1 expression in a high glucose-induced human mesangial cell proliferation model. *Int J Mol Med.* **2012**, *30*, 119-125. https://doi.org.10.3892/ijmm.2012.955.
[162] Lu, Q.; Ji, X.J.; Zhou, Y.X.; Yao, X.Q.; Liu, Y.Q.; Zhang, F.; Yin, X.X. Quercetin inhibits the mTORC1/p70S6K signaling-mediated renal tubular epithelial-mesenchymal transition and renal fibrosis in diabetic nephropathy. *Pharmacol Res.* **2015**, *99*, 237-247. https://doi.org.10.1016/j.phrs.2015.06.006.
[163] Lai, L.L.; Lu, H.Q.; Li, W.N.; Huang, H.P.; Zhou, H.Y.; Leng, E.N.; Zhang, Y.Y. Protective effects of quercetin and crocin in the kidneys and liver of obese Sprague-Dawley rats with Type 2 diabetes: Effects of quercetin and crocin on T2DM rats. *Hum Exp Toxicol.* **2021**, *40*, 661-672. https://doi.org.10.1177/0960327120954521.
[164] Ben Salem, I.; Prola, A.; Boussabbeh, M.; Guilbert, A.; Bacha, H.; Abid-Essefi, S.; Lemaire, C. Crocin and quercetin protect HCT116 and HEK293 cells from Zearalenone-induced apoptosis by reducing endoplasmic reticulum stress. *Cell Stress Chaperones.* **2015**, *20*, 927-938. https://doi.org.10.1007/s12192-015-0613-0.
[165] Chaudhary, S.; Ganjoo, P.; Raiusddin, S.; Parvez, S. Nephroprotective activities of quercetin with potential relevance to oxidative stress induced by valproic acid. *Protoplasma*. **2015**, *252*, 209-217. https://doi.org.10.1007/s00709-014-0670-8.
[166] Wang, L.; Lin, S.Q.; He, Y.L.; Liu, G.; Wang, Z.Y. Protective effects of quercetin on cadmium-induced cytotoxicity in primary cultures of rat proximal tubular cells. *Biomed Environ Sci.* **2013**, *26*, 258-267. https://doi.org.10.3967/0895-3988.2013.04.004.

[167] Tang, D.Q.; Wei, Y.Q.; Yin, X.X.; Lu, Q.; Hao, H.H.; Zhai, Y.P.; Wang, J.Y.; Ren, J. In vitro suppression of quercetin on hypertrophy and extracellular matrix accumulation in rat glomerular mesangial cells cultured by high glucose. *Fitoterapia.* **2011**, *82*, 920-926. https://doi.org. 10.1016/j.fitote.2011.05.001.
[168] Kuhlmann, M.K.; Burkhardt, G.; Horsch, E.; Wagner, M.; Köhler, H. Inhibition of oxidant-induced lipid peroxidation in cultured renal tubular epithelial cells (LLC-PK1) by quercetin. *Free Radic Res*. **1998**, *29*, 451-460. https://doi.org.10.1080/10715769800300501.
[169] Yokoo, T.; Kitamura, M. Unexpected protection of glomerular mesangial cells from oxidant-triggered apoptosis by bioflavonoid quercetin. *Am J Physiol*. **1997**, *273*, F206-12. https://doi.org.10.1152/ajprenal.1997.273.2.F206.
[170] Park, H.K.; Jeong, B.C.; Sung, M.K.; Park, M.Y.; Choi, E.Y.; Kim, B.S.; Kim, H.H.; Kim, J.I. Reduction of oxidative stress in cultured renal tubular cells and preventive effects on renal stone formation by the bioflavonoid quercetin. *J Urol.* **2008**, *179*, 1620-1626. https://doi.org. 10.1016/j.juro.2007.11.039.
[171] Kuhlmann, M.K.; Horsch, E.; Burkhardt, G.; Wagner, M.; Köhler, H. Reduction of cisplatin toxicity in cultured renal tubular cells by the bioflavonoid quercetin. *Arch Toxicol.* **1998**, *72*, 536-540. https://doi.org.10.1007/s002040050539.
[172] Ishikawa, Y.; Sugiyama, H.; Stylianou, E.; Kitamura, M. Bioflavonoid quercetin inhibits interleukin-1-induced transcriptional expression of monocyte chemoattractant protein-1 in glomerular cells via suppression of nuclear factor-kappaB. *J Am Soc Nephrol.* **1999**, *10*, 2290-2296. https://doi.org.10.1681/ASN.V10112290.
[173] Cai, H.D.; Su, S.L.; Qian, D.W.; Guo, S.; Tao, W.W.; Cong, X.D.; Tang, R.; Duan, J.A. Renal protective effect and action mechanism of Huangkui capsule and its main five flavonoids. *J Ethnopharmacol.* **2017**, *206*, 152-159. DOI: 10.1016/j.jep.2017.02.046.
[174] Joardar, S.; Dewanjee, S.; Bhowmick, S.; Dua, T.K.; Das, S.; Saha, A.; De Feo, V. Rosmarinic acid attenuates cadmium-induced nephrotoxicity via inhibition of oxidative stress, apoptosis, inflammation and fibrosis. *Int J Mol Sci.* **2019**, *20*, 2027. https://doi.org.10.3390/ijms20082027.
[175] Jiang, W.L.; Xu, Y.; Zhang, S.P.; Hou, J.; Zhu, H.B. Effect of rosmarinic acid on experimental diabetic nephropathy. *Basic Clin Pharmacol Toxicol.* **2012**, *110*, 390-395. https://doi.org.10.1111/j.1742-7843.2011.00828.x.
[176] Makino, T.; Ono, T.; Muso, E.; Yoshida, H.; Honda, G.; Sasayama, S. Inhibitory effects of rosmarinic acid on the proliferation of cultured murine mesangial cells. *Nephrol Dial Transplant*. **2000**, *15*, 1140-1145. https://doi.org.10.1093/ndt/15.8.1140.
[177] Hung, T.W.; Hsieh, Y.H.; Lee, H.L.; Ting, Y.H.; Lin, C.L.; Chao, W.W. Renoprotective effect of rosmarinic acid by inhibition of indoxyl sulfate-induced renal interstitial fibrosis via the NLRP3 inflammasome signaling. *Int Immunopharmacol.* **2024**, *135*, 112314. https://doi.org. 10.1016/j.intimp.2024.112314.
[178] Dong, R.; Zhang, X.; Liu, Y.; Zhao, T.; Sun, Z.; Liu, P.; Xiang, Q.; Xiong, J.; Du, X.; Yang, X.; Gui, D.; Xu. Y Rutin alleviates EndMT by restoring autophagy through inhibiting HDAC1 via PI3K/AKT/mTOR pathway in diabetic kidney disease. *Phytomedicine.* **2023**, *112*, 154700. https://doi.org.10.1016/j.phymed.2023.154700.
[179] Qu, S.; Dai, C.; Guo, H.; Wang, C.; Hao, Z.; Tang, Q.; Wang, H.; Zhang, Y. Rutin attenuates vancomycin-induced renal tubular cell apoptosis via suppression of apoptosis, mitochondrial dysfunction, and oxidative stress. *Phytother Res.* **2019**, *33*, 2056-2063. https://doi.org.10.1002/ptr.6391.
[180] Wang, X.; Zhao, X.; Feng, T.; Jin, G.; Li, Z. Rutin prevents high glucose-induced renal glomerular endothelial hyperpermeability by inhibiting the ROS/Rhoa/ROCK signaling pathway. *Planta Med.* **2016**, *82*, 1252-1257. https://doi.org.10.1055/s-0042-110859.
[181] Zhang, Y.; Wang, Q.; Wang, Y.D.; Sun, B.; Leng, X.W.; Li, Q.; Ren, L.Q. Effect of rutin on cisplatin-induced damage in human mesangial cells via apoptotic pathway. *Hum Exp Toxicol.* **2019**, *38*, 118-128. https://doi.org.10.1177/0960327118785233.

[182] Liang, Y.; Zeng, X.; Guo, J.; Liu, H.; He, B.; Lai, R.; Zhu, Q.; Zheng, Z. Scopoletin and umbelliferone from Cortex Mori as protective agents in high glucose-induced mesangial cell as *in vitro* model of diabetic glomerulosclerosis. *Chin J Physiol*. **2021**, *64*, 150-158. https://doi.org.10.4103/cjp.cjp_9_21.
[183] Kundu, S.; Ghosh, S.; Sahu, B.D. Scopoletin alleviates high glucose-induced toxicity in human renal proximal tubular cells via inhibition of oxidative damage, epithelial-mesenchymal transition, and fibrogenesis. *Mol Biol Rep*. **2024**, *51*, 620. https://doi.org. 10.1007/s11033-024-09579-2.
[184] Liu, D.; Zhang, C.; Hu, M.; Su, K. Scutellarein relieves the death and inflammation of tubular epithelial cells in ischemic kidney injury by degradation of COX-2 protein. *Int Immunopharmacol*. **2021**, *101*, 108193. https://doi.org.10.1016/j.intimp.2021.108193.
[185] Ding, T.; Wang, S.; Zhang, X.; Zai, W.; Fan, J.; Chen, W.; Bian, Q.; Luan, J.; Shen, Y.; Zhang, Y.; Ju, D.; Mei, X. Kidney protection effects of dihydroquercetin on diabetic nephropathy through suppressing ROS and NLRP3 inflammasome. *Phytomedicine*. **2018**, *41*, 45-53. https://doi.org.10.1016/j.phymed.2018.01.026.
[186] Gao, L.; Yuan, P.; Zhang, Q.; Fu, Y.; Hou, Y.; Wei, Y.; Zheng, X.; Feng, W. Taxifolin improves disorders of glucose metabolism and water-salt metabolism in kidney via PI3K/AKT signaling pathway in metabolic syndrome rats. *Life Sci*. **2020**, *263*, 118713. https://doi.org.10.1016/j.lfs.2020.118713.
[187] Wang, W.; Ma, B.L.; Xu, C.G.; Zhou, X.J. Dihydroquercetin protects against renal fibrosis by activating the Nrf2 pathway. *Phytomedicine*. **2020**, *69*, 153185. https://doi.org.10.1016/j.phymed.2020.153185.
[188] Julià, F.; Costa-Bauza, A.; Berga, F.; Grases, F. Effect of theobromine on dissolution of uric acid kidney stones. *World J Urol*. **2022**, *40*, 2105-2111. https://doi.org.10.1007/s00345-022-04059-3.
[189] Ma, T.K.; Xu, L.; Lu, L.X.; Cao, X.; Li, X.; Li, L.L.; Wang, X.; Fan, Q.L. Ursolic Acid Treatment Alleviates Diabetic Kidney Injury By Regulating The ARAP1/AT1R Signaling Pathway. *Diabetes Metab Syndr Obes*. **2019**, *12*, 2597-2608. https://doi.org.10.2147/DMSO.S222323.
[190] Jia, Z.; Li, W.; Bian, P.; Yang, L.; Liu, H.; Pan, D.; Dou, Z. Ursolic acid treats renal tubular epithelial cell damage induced by calcium oxalate monohydrate via inhibiting oxidative stress and inflammation. *Bioengineered*. **2021**, *12*, 5450-5461. https://doi.org.10.1080/21655979.2021.1955176.
[191] Xu, C.G.; Zhu, X.L.; Wang, W.; Zhou, X.J. Ursolic acid inhibits epithelial-mesenchymal transition in vitro and in vivo. *Pharm Biol*. **2019**, *57*, 169-175. https://doi.org.10.1080/13880209.2019.1577464.
[192] Xu, L.; Fan, Q.; Wang, X.; Li, L.; Lu, X.; Yue, Y.; Cao, X.; Liu, J.; Zhao, X.; Wang, L. Ursolic acid improves podocyte injury caused by high glucose. *Nephrol Dial Transplant*. **2017**, *32*, 1285-1293. https://doi.org.10.1093/ndt/gfv382.
[193] Lu, X.; Fan, Q.; Xu, L.; Li, L.; Yue, Y.; Xu, Y.; Su, Y.; Zhang, D.; Wang, L. Ursolic acid attenuates diabetic mesangial cell injury through the up-regulation of autophagy via miRNA-21/PTEN/Akt/mTOR suppression. *PLoS One*. **2015**, *10*, e0117400. https://doi.org.10.1371/journal.pone.0117400.
[194] Li, C.; Chen, W.; Zheng, L.; Zhang, B.; Yang, X.; Zhang, Q.; Wang, N.; Wang, Y.; Yang, J.; Sha, J.; Zhou, Z.; Li, X.; Li, Y.; Shen, X.L. Ameliorative effect of ursolic acid on ochratoxin A-induced renal cytotoxicity mediated by Lonp1/Aco2/Hsp75. *Toxicon*. **2019**, *168*, 141-146. https://doi.org.10.1016/j.toxicon.2019.07.014.
[195] Wang, E.M., Fan, Q.L.; Yue, Y.; Xu L. Ursolic acid attenuates high glucose-mediated mesangial cell injury by inhibiting the phosphatidylinositol 3-kinase/Akt/mammalian target of rapamycin (PI3K/Akt/mTOR) signaling pathway. *Med Sci Monit*. **2018**, *24*, 846-854. https://doi.org.10.12659/msm.907814.
[196] Ding, T.; Zhao, T.; Li, Y.; Liu, Z.; Ding, J.; Ji, B.; Wang, Y.; Guo, Z. Vitexin exerts protective effects against calcium oxalate crystal-induced kidney pyroptosis in vivo and in vitro. *Phytomedicine*. **2021**, *86*, 153562. https://doi.org.10.1016/j.phymed.2021.153562.

[197] Shen, P.; Yang, X.; Jiang, J.; Wang, X.; Liang, T.; He, L. Wedelolactone from Eclipta alba inhibits lipopolysaccharide-enhanced cell proliferation of human renal mesangial cells via NF-κB signaling pathway. *Am J Transl Res*. **2017**, *9*, 2132-2142.
[198] Wang, G.; Bi, Y.; Xiong, H.; Bo, T.; Han, L.; Zhou, L.; Zhang, C.; Zhang, Y. Wedelolactone protects against cisplatin-induced nephrotoxicity in mice via inhibition of organic cation transporter 2. *Hum Exp Toxicol*. **2021**, *40*, S447-S459. https://doi.org.10.1177/09603271211047915.
[199] Zhu, M.M.; Wang, L.; Yang, D.; Li, C.; Pang, S.T.; Li, X.H.; Li, R.; Yang, B.; Lian, Y.P.; Ma, L.; Lv, Q.L.; Jia, X.B.; Feng, L. Wedelolactone alleviates doxorubicin-induced inflammation and oxidative stress damage of podocytes by IκK/IκB/NF-κB pathway. *Biomed Pharmacother.* **2019**, *117*, 109088. https://doi.org.10.1016/j.biopha.2019.109088.
[200] Zhi, D.; Zhang, M.; Lin, J.; Liu, P.; Wang, Y.; Duan, M. Wedelolactone improves the renal injury induced by lipopolysaccharide in HK-2 cells by upregulation of protein tyrosine phosphatase non-receptor type 2. *J Int Med Res*. **2021**, *49*, 3000605211012665. https://doi.org.10.1177/03000605211012665.
[201] Wang, Y.; Liu, L.; Ge, M.; Cui, J.; Dong, X.; Shao. Y. Acacetin attenuates the pancreatic and hepatorenal dysfunction in type 2 diabetic rats induced by high-fat diet combined with streptozotocin. *J Nat Med*. **2023**, *77*, 446-454. https://doi.org.10.1007/s11418-022-01675-6.
[202] Jalili, C.; Akhshi, N.; Raissi, F.; Shiravi, A.; Alvani, A.; Vaezi, G.; Nedaei, S.E.; Ghanbari, A. Acacetin Alleviates Hepatitis Following Renal Ischemia-Reperfusion in Male Balb/C Mice by Antioxidants Regulation and Inflammatory Markers Suppression. *J Invest Surg*. **2021**, *34*, 495-503. https://doi.org.10.1080/08941939.2019.1656309.
[203] Shiravi, A.; Jalili, C.; Vaezi, G.; Ghanbari, A.; Alvani, A. Acacetin Attenuates Renal Damage-Induced by Ischemia-Reperfusion with Declining Apoptosis and Oxidative Stress in Mice. *Int J Prev Med*. **2020**, *11*, 22. https://doi.org.10.4103/ijpvm.IJPVM_512_18.
[204] Ijaz, M.; Akbar, A.; Ashraf, A.; Saad, A.; Alkahtane, A.; Riaz, M. Antioxidant, anti-inflammatory and anti-apoptotic effects of amentoflavone on gentamicin-induced kidney damage in rats. *J. King Saud Univ. Sci.* **2023**, *35*, 102791 https://doi.org/10.1016/j.jksus.2023.102791.
[205] Hashish, E.A.; Elgaml, S.A.; El-Fattah, A.; Shalaby, S.I.; Abdelaziz, S. β-Amyrin supplementation ameliorates the toxic effect of glycerol in the kidney of rat model. *Hum Exp Toxicol*. **2020**, *39*, 930-937. https://doi.org.10.1177/0960327120907136.
[206] Sahindokuyucu-Kocasari, F.; Akyol, Y.; Ozmen, O.; Erdemli-Kose, S.B.; Garli, S. Apigenin alleviates methotrexate-induced liver and kidney injury in mice. *Hum. Exp. Toxicol*. **2021**, *Oct;40(10)*, 1721-1731. https://doi.org.10.1177/09603271211009964.
[207] Almaghrabi,S.Y. Apigenin ameliorates hypercholesterolemic-induced kidney injury via modulating renal KIM-1, Fn1, and Nrf2 signaling pathways. *Eur Rev Med Pharmacol Sci*. **2023**, *27*, 1155-1169. https://doi.org.10.26355/eurrev_202302_31222.
[208] Hussein, M.M.; Althagafi, H.A.; Alharthi, F.; Albrakati, A.; Alsharif, K.F.; Theyab, A.; Kassab, R.B.; Mufti, A.H.; Algahtani, M.; Oyouni, A.A.A.; Baty, R.S.; Abdel Moneim, A.E.; Lokman, M.S. Apigenin attenuates molecular, biochemical, and histopathological changes associated with renal impairments induced by gentamicin exposure in rats. *Environ Sci Pollut Res Int*. **2022**, *29*, 65276-65288. https://doi.org.10.1007/s11356-022-20235-9.
[209] Fehaid, A.; Al-Ghamdi, M.S.; Alzahrani, K.J.; Theyab, A.; Al-Amer, O.M.; Al-Shehri, S.S.; Algahtani, M.; Oyouni, A.A.A.; Alnfiai, M.M.; Aly, M.H.; Alsharif, K.F.; Albrakati, A.; Kassab, R.B.; Althagafi, H.A.; Alharthi, F.; Moneim, A.E.A.; Lokman, M.S. Apigenin protects from hepatorenal damage caused by lead acetate in rats. *J Biochem Mol Toxicol*. **2023**, *37*, e23275. https://doi.org.10.1002/jbt.23275.
[210] He, X.; Li, C.; Wei, Z.; Wang, J.; Kou, J.; Liu, W.; Shi, M.; Yang, Z.; Fu, Y. Protective role of apigenin in cisplatin-induced renal injury. *Eur J Pharmacol*. **2016**, *789*, 215-221. https://doi.org.10.1016/j.ejphar.2016.07.003.

[211] Wang, T.; Zhang, Z.; Xie, M.; Li, S.; Zhang, J.; Zhou, J. Apigenin attenuates mesoporous silica nanoparticles-induced nephrotoxicity by activating FOXO3a. *Biol Trace Elem Res*. **2022**, *200*, 2793-2806. https://doi.org.10.1007/s12011-021-02871-3.
[212] Liu, T.; Gao, H.; Zhang, Y.; Wang, S.; Lu, M.; Dai, X.; Liu, Y.; Shi, H.; Xu, T.; Yin, J.; Gao, S.; Wang, L.; Zhang, D. Apigenin ameliorates hyperuricemia and renal injury through regulation of uric acid metabolism and JAK2/STAT3 signaling pathway. *Pharmaceuticals.* **2022**, *15*, 1442. https://doi.org.10.3390/ph15111442.
[213] Wei, X.; Gao, P.; Pu, Y.; Li, Q.; Yang, T.; Zhang, H.; Xiong, S.; Cui, Y.; Li, L.; Ma, X.; Liu, D.; Zhu, Z. Activation of TRPV4 by dietary apigenin antagonizes renal fibrosis in deoxycorticosterone acetate (DOCA)-salt-induced hypertension. *Clin Sci*. **2017**, *131*, 567-581. https://doi.org.10.1042/CS20160780.
[214] Azimi, A.; Eidi, A.; Mortazavi, P.; Rohani, A.H. Protective effect of apigenin on ethylene glycol-induced urolithiasis via attenuating oxidative stress and inflammatory parameters in adult male Wistar rats. *Life Sci*. **2021**, *279*, 119641. https://doi.org.10.1016/j.lfs.2021.119641.
[215] Hassan, S.M.; Khalaf, M.M.; Sadek, S.A.; Abo-Youssef, A.M. Protective effects of apigenin and myricetin against cisplatin-induced nephrotoxicity in mice. *Pharm Biol*. **2017**, *55*, 766-774. https://doi.org.10.1080/13880209.2016.1275704.
[216] Ali, A.A.; Mansour, A.B.; Attia, S.A. The potential protective role of apigenin against oxidative damage induced by nickel oxide nanoparticles in liver and kidney of male Wistar rat, Rattus norvegicus. *Environ Sci Pollut Res Int*. **2021**, *28*, 27577-27592. https://doi.org.10.1007/s11356-021-12632-3.
[217] Stiani, S.N.; Syahidah, F.M.; Fikriani, H.; Subarnas, A.; Rusdiana, T. Anticalculi Activity of Apigenin and Celery (*Apium graveolens* L.) Extract in Rats Induced by Ethylene Glycol-Ammonium Chloride. *J Pharm Bioallied Sci*. **2019**, *11*, S556-S561. https://doi.org.10.4103/jpbs.JPBS_202_19.
[218] Malik, S.; Suchal, K.; Khan, S.I.; Bhatia, J.; Kishore, K.; Dinda, A.K.; Arya, D.S. Apigenin ameliorates streptozotocin-induced diabetic nephropathy in rats via MAPK-NF-κB-TNF-α and TGF-β1-MAPK-fibronectin pathways. *Am J Physiol Renal Physiol*. **2017**, *313*, F414-F422. https://doi.org.10.1152/ajprenal.00393.2016.
[219] Zhong, Y.; Jin, C.; Wang, X.; Li, X.; Han, J.; Xue, W.; Wu, P.; Peng, X.; Xia, X. Protective effects of apigenin against 3-MCPD-induced renal injury in rat. *Chem Biol Interact*. **2018**, *296*, 9-17. https://doi.org.10.1016/j.cbi.2018.08.005.
[220] Ahmad, A.; Kumari, P.; Ahmad, M. Apigenin attenuates edifenphos-induced toxicity by modulating ROS-mediated oxidative stress, mitochondrial dysfunction and caspase signal pathway in rat liver and kidney. *Pestic Biochem Physiol*. **2019**, *159*, 163-172. https://doi.org. 10.1016/j.pestbp.2019.06.010.
[221] Khaled, M.; Salama, R.A.M.; Aboughalia, A.; Tarek, M.; Fawzy, N.M. Apigenin ameliorates genitourinary dysfunction in a type 1 diabetic rat model via Drp1 modulation. *Sci Rep*. 2024, *14*, 5754. https://doi.org.10.1038/s41598-024-56395-6.
[222] Abd El-Aal, S.A.; El-Sayyad, S.M.; El-Gazar, A.A.; Ibrahim, S.S.A; Essa, M.A.; Abostate, H.M.; Ragab, G.M. Boswellic acid and apigenin alleviate methotrexate-provoked renal and hippocampal alterations in rats: Targeting autophagy, NOD-2/NF-κB/NLRP3, and connexin-43. *Int Immunopharmacol*. **2024**, *134*, 112147. https://doi.org.10.1016/j.intimp.2024.112147.
[223] Xu, C.; Wang, W.; Xu, M.; Zhang J. Asiatic acid ameliorates tubulointerstitial fibrosis in mice with ureteral obstruction. Exp. *Therap. Med*. **2013**, *6*, 731-736. https://doi.org/10.3892/etm.2013.1197
[224] Kamble, S.M.; Patil, C.R. Asiatic acid ameliorates doxorubicin-induced cardiac and hepato-renal toxicities with Nrf2 transcriptional factor activation in rats. *Cardiovasc Toxicol*. **2018**, *18*, 131-141. https://doi.org.10.1007/s12012-017-9424-0.

[225] Chen, Y.N.; Wu, C.G.; Shi, B.M.; Qian, K.; Ding, Y. The protective effect of asiatic acid on podocytes in the kidney of diabetic rats. *Am J Transl Res*. **2018**, *10*, 3733-3741.
[226] Yang, C.; Guo, Y.; Huang, T.S.; Zhao, J.; Huang, X.J.; Tang, H.X.; An, N.; Pan, Q.; Xu, Y.Z.; Liu, H.F. Asiatic acid protects against cisplatin-induced acute kidney injury via anti-apoptosis and anti-inflammation. *Biomed Pharmacother*. **2018**, *107*, 1354-1362. https://doi.org.10.1016/j.biopha.2018.08.126.
[227] Zhang, Z.H., He, J.Q.; Zhao, Y.Y.; Chen, H.C.; Tan, N.H. Asiatic acid prevents renal fibrosis in UUO rats via promoting the production of 15d-PGJ2, an endogenous ligand of PPAR-γ. *Acta Pharmacol Sin*. **2020**, *41*, 373-382. https://doi.org.10.1038/s41401-019-0319-4.
[228] Qiu, W.; Zhang, X.; Pang, X.; Huang, J.; Zhou, S.; Wang, R.; Tang, Z.; Su, R. Asiatic acid alleviates LPS-induced acute kidney injury in broilers by inhibiting oxidative stress and ferroptosis via activation of the Nrf2 pathway. *Food Chem Toxicol*. **2022**, *170*, 113468. https://doi.org.10.1016/j.fct.2022.113468.
[229] Ji,Y.; Zhang, X.; Chen, J.; Song, S.; Fang, S.; Wang, Z.; Xu, S.; Xu, Y.; Liu, J.; Jiang, C.; Pan, K.; Zhang, J.; Wang, L.; Yin, Z. Asiatic acid attenuates tubular injury in diabetic kidney disease by regulating mitochondrial dynamics via the Nrf-2 pathway. *Phytomedicine*. **2023**, *109*, 154552. https://doi.org.10.1016/j.phymed.2022.154552.
[230] Kalidhindi, S.; Uddandrao, V.V.S.; Sasikumar, V.; Raveendran, N.; Ganapathy, S. Mitigating Perspectives of Asiatic Acid in the Renal Derangements of Streptozotocin-Nicotinamide Induced Diabetic Rats. *Cardiovasc Hematol Agents Med Chem*. **2020**, *18*, 37-44. https://doi.org.10.2174/1871525718666200131121419.
[231] Chen, X.C.; Huang, L.F.; Tang, J.X.; Wu, D.; An, N.; Ye, Z.N.; Lan, H.Y.; Liu, H.F.; Yang, C. Asiatic acid alleviates cisplatin-induced renal fibrosis in tumor-bearing mice by improving the TFEB-mediated autophagy-lysosome pathway. *Biomed Pharmacother*. **2023**, *165*, 115122. https://doi.org.10.1016/j.biopha.2023.115122.
[232] Wang, Z.; Liu, J.; Sun, W. Effects of asiaticoside on levels of podocyte cytoskeletal proteins and renal slit diaphragm proteins in adriamycin-induced rat nephropathy. *Life Sci*. **2013**, *93*, 352-358. https://doi.org.10.1016/j.lfs.2013.07.010.
[233] Tang, S.; Xie, X.; Wang, M.; Yang, L.; Wei, W. Protective effects of asiaticoside on renal ischemia reperfusion injury in vivo and in vitro. *Bioengineered*. **2022**, *13*, 10235-10243. https://doi.org.10.1080/21655979.2022.2061302.
[234] Tang, S.; Xie, X.; Wang, M.; Wei, W. Asiaticoside ameliorates renal ischemia/reperfusion injury by promoting CD4+CD25+FOXP3+ treg cell differentiation. *Heliyon*. 2023, *9*, e17390. https://doi.org.10.1016/j.heliyon.2023.e17390.
[235] Teixeira, R.G.S.; Pascual, R.; Lima-Araújo, K.G.; Brito, M.A.; Los Rios, C.; Carmo, A.F.; Gandía, L.; Silva, C.L.M.; Machado, T.B.; Santos, W.C. *In vitro* and *in silico* studies for barbinervic acid, a triterpene isolated from *Eugenia punicifolia* that inhibits vasopressor tone. *Nat Prod Res*. **2021**, *35*, 4870-4875. https://doi.org.10.1080/14786419.2020.
[236] Koc, K.; Geyikoglu, F.; Cakmak, O.; Koca, A.; Kutlu, Z.; Aysin, F.; Yilmaz, A.; Aşkın, H. The targets of β-sitosterol as a novel therapeutic against cardio-renal complications in acute renal ischemia/reperfusion damage. *Naunyn Schmiedebergs Arch Pharmacol*. **2021**, *394*, 469-479. https://doi.org.10.1007/s00210-020-01984-1.
[237] Sharmila, R.; Sindhu, G.; Arockianathan, P.M. Nephroprotective effect of β-sitosterol on N-diethylnitrosamine initiated and ferric nitrilotriacetate promoted acute nephrotoxicity in Wistar rats. *J Basic Clin Physiol Pharmacol*. **2016**, *27*, 473-482. https://doi.org.10.1515/jbcpp-2015-0085.
[238] Sharmila, R.; Sindhu, G. Modulation of angiogenesis, proliferative response and apoptosis by β-sitosterol in rat model of renal carcinogenesis. *Indian J Clin Biochem*. **2017**, *32*, 142-152. https://doi.org.10.1007/s12291-016-0583-8.

[239] Mohsin, N.; Akhtar, M.S.; Alkahtani, S.A.; Walbi, I.A.; Alhazmi, Y.; Alam, M.N.; Bhardwaj, A. Nephroprotective Effect of Bergapten Against Cyclophosphamide-Mediated Renal Stress, Inflammation, and Fibrosis in Wistar Rats: Probable Role of NF-kB and TGF-β1 Signaling Molecules. *ACS Omega*. **2024**, *9*, 18296-18303. https://doi.org.10.1021/acsomega.4c00124.
[240] Khataylou, Y.J.; Afshar, S.A.; Mirzakhani N. Betulinic acid reduces the complications of autoimmune diabetes on the body and kidney through effecting on inflammatory cytokines in C57BL/6 mice. *Vet Res Forum*. **2021**, *12*, 203-210. https://doi.org.10.30466/vrf.2019.101178.2409.
[241] Sharma, A.; Thakur, R.; Lingaraju, M.C.; Kumar, D.; Mathesh, K.; Telang, A.G.; Singh, T.U.; Kumar, D. Betulinic acid attenuates renal fibrosis in rat chronic kidney disease model. *Biomed Pharmacother*. **2017**, *89*, 796-804. https://doi.org.10.1016/j.biopha.2017.01.181.
[242] Adeleke, G.E.; Adaramoye, O.A. Betulinic acid abates N-nitrosodimethylamine-induced changes in lipid metabolism, oxidative stress, and inflammation in the liver and kidney of Wistar rats. *J Biochem Mol Toxicol*. **2021**, *35*, e22901. https://doi.org.10.1002/jbt.22901.
[243] Alherz, F.A.; Elekhnawy, E.; Selim, H.M.; El-Masry, T.A.; El-Kadem, A.H.; Hussein, I.A.; Negm, W.A. Protective role of betulinic acid against cisplatin-induced nephrotoxicity and its antibacterial potential toward uropathogenic bacteria. *Pharmaceuticals*. **2023**, *16*, 1180. https://doi.org.10.3390/ph16081180.
[244] Fan, R.; Hu, P.C.; Wang, Y.; Lin, H.Y.; Su, K.; Feng, X.S.; Wei, L.; Yang, F. Betulinic acid protects mice from cadmium chloride-induced toxicity by inhibiting cadmium-induced apoptosis in kidney and liver. *Toxicol Lett*. **2018**, *299*, 56-66. https://doi.org.10.1016/j.toxlet.2018.09.003.
[245] Huang, L.; Zhu, L.; Ou, Z.; Ma, C.; Kong, L.; Huang, Y.; Chen, Y.; Zhao, H.; Wen, L.; Wu, J.; Yuan, Z.; Yi, J. Betulinic acid protects against renal damage by attenuation of oxidative stress and inflammation via Nrf2 signaling pathway in T-2 toxin-induced mice. *Int Immunopharmacol*. **2021**, *101*, 108210. https://doi.org.10.1016/j.intimp.2021.108210.
[246] Berköz, M.; Çiftçi O. Boswellic Acid and Betulinic Acid Pre-treatments Can Prevent the Nephrotoxicity Caused by Cyclophosphamide Induction. *Dokl Biochem Biophys*. **2024**, *517*, 115-126. https://doi.org.10.1134/S1607672924600234
[247] Ekşioğlu-Demiralp, E.; Kardaş, E.R.; Ozgül, S.; Yağci, T.; Bilgin, H.; Sehirli, O.; Ercan, F.; Sener G. Betulinic acid protects against ischemia/reperfusion-induced renal damage and inhibits leukocyte apoptosis. *Phytother Res*. **2010**, *24*, 325-332. https://doi.org.10.1002/ptr.2929.
[248] Lingaraju, M.C.; Pathak, N.N.; Begum, J.; Balaganur, V.; Ramachandra, H.D.; Bhat, R.A.; Ram, M.; Singh, V.; Kandasamy, K.; Kumar, D.; Kumar, D.; Tandan, S.K. Betulinic acid attenuates renal oxidative stress and inflammation in experimental model of murine polymicrobial sepsis. *Eur J Pharm Sci*. **2015**, *70*, 12-21. https://doi.org.10.1016/j.ejps.2015.01.001.
[249] Sutariya, B.; Taneja, N.; Saraf, M. Betulinic acid, isolated from the leaves of Syzygium cumini (L.) Skeels, ameliorates the proteinuria in experimental membranous nephropathy through regulating Nrf2/NF-κB pathways. *Chem Biol Interact*. **2017**, *274*, 124-137. https://doi.org.10.1016/j.cbi.2017.07.011.
[250] Wang, S.; Yang, Z.; Xiong, F.; Chen, C.; Chao, X.; Huang, J.; Huang, H. Betulinic acid ameliorates experimental diabetic-induced renal inflammation and fibrosis via inhibiting the activation of NF-κB signaling pathway. *Mol Cell Endocrinol*. **2016**, *434*, 135-143. https://doi.org.10.1016/j.mce.2016.06.019.
[251] Adeleke, G.E.; Adaramoye, O.A. Modulatory role of betulinic acid in N-nitrosodimethylamine-induced hepatorenal toxicity in male Wistar rats. *Hum Exp Toxicol*. **2017**, *36*, 734-743. https://doi.org.10.1177/0960327116661399.
[252] Zhu, L.; Luo, C.; Ma, C.; Kong, L.; Huang, Y.; Yang, W.; Huang, C.; Jiang, W.; Yi, J. Inhibition of the NF-κB pathway and ERK-mediated mitochondrial apoptotic pathway takes part in the mitigative effect of betulinic acid on inflammation and oxidative stress in cyclophosphamide-triggered renal damage of mice. *Ecotoxicol Environ Saf*. **2022**, *246*, 114150. https://doi.org.10.1016/j.ecoenv.2022.114150.

[253] Xie, R.; Zhang, H.; Wang, X.Z.; Yang, X.Z.; Wu, S.N.; Wang, H.G.; Shen, P.; Ma, T.H. The protective effect of betulinic acid (BA) diabetic nephropathy on streptozotocin (STZ)-induced diabetic rats. *Food Funct*. **2017**, *8*, 299-306. https://doi.org.10.1039/c6fo01601d.
[254] Ahangarpour, A.; Oroojan, A.A.; Khorsandi, L.; Shabani, R.; Mojaddami, S. Preventive effects of betulinic acid on streptozotocinnicotinamide induced diabetic nephropathy in male mouse. *J Nephropathol.* **2016**, *5*, 128-133. https://doi.org. 10.15171/jnp.2016.24.
[255] Ma, J.Q.; Zhang, Y.J.; Tian, Z.K.; Liu, C.M. Bixin attenuates carbon tetrachloride induced oxidative stress, inflammation and fibrosis in kidney by regulating the Nrf2/TLR4/MyD88 and PPAR-γ/TGF-β1/Smad3 pathway. *Int Immunopharmacol*. **2021**, *90*, 107117. https://doi.org.10.1016/j.intimp.2020.107117.
[256] Martins de Sá Müller, C.; Coelho, G.B.; Paula Michel Araújo, M.C.; Saúde-Guimarães, DA. *Lychnophora pinaster* ethanolic extract and its chemical constituents ameliorate hyperuricemia and related inflammation. *J. Ethnopharmacol.* **2019**, *242*, 112040. https://doi.org.10.1016/j.jep.2019.112040.
[257] Yasir, F.; Wahab, A.T.; Choudhary, M.I. Protective effect of dietary polyphenol caffeic acid on ethylene glycol-induced kidney stones in rats. *Urolithiasis*. **2018**, *46*, 157-166. https://doi.org.10.1007/s00240-017-0982-1.
[258] Matboli, M.; Eissa, S.; Ibrahim, D.; Hegazy, M.G.A.; Imam, S.S.; Habib, E.K. Caffeic acid attenuates diabetic kidney disease via modulation of autophagy in a high-fat diet/streptozotocin- induced diabetic rat. *Sci Rep*. **2017**, *7*, 2263. https://doi.org.10.1038/s41598-017-02320-z.
[259] Chao, C.Y.; Mong, M.C.; Chan, K.C.; Yin, M.C. Anti-glycative and anti-inflammatory effects of caffeic acid and ellagic acid in kidney of diabetic mice. *Mol Nutr Food Res*. **2010**, *54*, 388-395. https://doi.org. 10.1002/mnfr.200900087.
[260] Kinra, M.; Arora, D.; Mudgal, J.; Pai, K.S.R.; Mallikarjuna Rao, C.; Nampoothiri, M. Effect of caffeic acid on ischemia-reperfusion-induced acute renal failure in rats. *Pharmacology.* **2019**, *103*, 315-319. https://doi.org.10.1159/000497474.
[261] Olayinka, E.T.; Ola, O.S.; Ore, A.; Adeyemo, O.A. Ameliorative effect of caffeic acid on capecitabine-induced hepatic and renal dysfunction: involvement of the antioxidant defense system. *Medicines*. **2017**, *78*. https://doi.org.10.3390/medicines4040078.
[262] Owumi, S.E.; Irozuru, C.E.; Arunsi, U.O.; Oyelere, A.K. Caffeic acid protects against DNA damage, oxidative and inflammatory mediated toxicities, and upregulated caspases activation in the hepatorenal system of rats treated with aflatoxin B1. *Toxicon*. **2022**, *207*, 1-12. https://doi.org.10.1016/j.toxicon.2021.12.021.
[263] Veeren, B.; Bringart, M.; Turpin, C.; Rondeau, P.; Planesse, C.; Ait-Arsa, I.; Gimié, F.; Marodon, C.; Meilhac, O.; Gonthier, M.P.; Diotel, N.; Bascands, J.L. Caffeic acid, one of the major phenolic acids of the medicinal plant *Antirhea borbonica*, reduces renal tubulointerstitial fibrosis. Biomedicines. **2021**, *9*, 358. https://doi.org.10.3390/biomedicines9040358.
[264] Gad, A.M. Study on the influence of caffeic acid against sodium valproate-induced nephrotoxicity in rats. *J Biochem Mol Toxicol.* **2018**, *32*, e22175. https://doi.org.10.1002/jbt.22175.
[265] Rieg, T.; Steigele, H.; Schnermann, J.; Richter, K.; Osswald, H.; Vallon, V. Requirement of intact adenosine A1 receptors for the diuretic and natriuretic action of the methylxanthines theophylline and caffeine. *J Pharmacol Exp Ther.* **2005**, *313*, 403-409. https://doi.org.10.1124/jpet.104.080432.
[266] Fenton, R.A.; Poulsen, S.B.; Mora Chavez, S.; Soleimani, M.; Busslinger, M.; Dominguez Rieg, J.A.; Rieg, T. Caffeine-induced diuresis and natriuresis is independent of renal tubular NHE3. *Am J Physiol Renal Physiol.* **2015**, *308*, F1409-20. https://doi.org.10.1152/ajprenal.00129.2015.
[267] Meca, R.; Balbo, B.E.; Ormanji, M.S.; Fonseca, J.M.; Iannuzzi, L.R.; Santana Costa, E.; Onuchic, L.F.; Heilberg, I.P. Caffeine accelerates cystic kidney disease in a Pkd1-deficient mouse model. *Cell Physiol Biochem.* **2019**, *52*, 1061-1074. https://doi.org.10.33594/000000072.

[268] Anwar, M.M.; Laila, I.M.I. Mitigative effect of caffeine against diclofenac-induced hepato-renal damage and chromosomal aberrations in male albino rats. *BMC Complement Med Ther.* **2022**, *22*, 327. https://doi.org.10.1186/s12906-022-03802-y.
[269] Khazaei, M.; Bayat, P.D.; Ghanbari, A.; Khazaei, S.; Feizian, M.; Khodaei, A.; Alian, H.A. Protective effects of subchronic caffeine administration on cisplatin induced urogenital toxicity in male mice. *Indian J Exp Biol.* **2012**, *50*, 638-644.
[270] Wongmekiat, O.; Peerapanyasut, W.; Kobroob, A. Catechin supplementation prevents kidney damage in rats repeatedly exposed to cadmium through mitochondrial protection. *Naunyn Schmiedebergs Arch Pharmacol.* **2018**, *391*, 385-394. https://doi.org.10.1007/s00210-018-1468-6.
[271] Khattab, S.A.; Hussien, W.F.; Raafat, N.; Ahmed Alaa El-Din, E. Modulatory effects of catechin hydrate on benzo[a]pyrene-induced nephrotoxicity in adult male albino rats. *Toxicol Res (Camb).* **2021**, *10*, 542-550. https://doi.org.10.1093/toxres/tfab029.
[272] Wang, H.; Liu, Z.; Liu, S.; Yang, R.; Wang, Y.; Gu, Y.; Wu, M.; Dong, R.; Chen, B. The therapeutic effect of catechin on nephrolithiasis induced by co-exposure to melamine and cyanuric acid in Sprague-Dawley rats. *Toxics*. **2023**, *11*, 799. https://doi.org.10.3390/toxics11090799.
[273] Sardana, A.; Kalra S, S.; Khanna, D.; Balakumar, P. Nephroprotective effect of catechin on gentamicin-induced experimental nephrotoxicity. *Clin Exp Nephrol.* **2015**, *19*, 178-184. https://doi.org.10.1007/s10157-014-0980-3.
[274] Zhu, D.; Wang, L.; Zhou, Q.; Yan, S.; Li, Z.; Sheng, J.; Zhang, W. (+)-Catechin ameliorates diabetic nephropathy by trapping methylglyoxal in type 2 diabetic mice. *Mol Nutr Food Res*. **2014**, *58*, 2249-2260. https://doi.org.10.1002/mnfr.201400533.
[275] Chander, V.; Singh, D.; Chopra, K. Catechin, a natural antioxidant protects against rhabdomyolysis-induced myoglobinuric acute renal failure. *Pharmacol Res.* **2003**, *48*, 503-509. https://doi.org.10.1016/s1043-6618(03)00207-x.
[276] Chopra, K.; Singh, D.; Chander, V. Nephrotoxicity and its prevention by catechin in ferric nitrilotriacetate promoted oxidative stress in rats. *Hum Exp Toxicol.* **2004**, *23*, 137-143. https://doi.org.10.1191/0960327104ht427oa.
[277] Anjaneyulu, M.; Tirkey, N.; Chopra, K. Attenuation of cyclosporine-induced renal dysfunction by catechin: possible antioxidant mechanism. *Ren Fail*. **2003**, *25*, 691-707. https://doi.org.10.1081/jdi-120024285.
[278] Amengual-Cladera, E.; Nadal-Casellas, A.; Gómez-Pérez, Y.; Gomila, I.; Prieto, R.M.; Proenza, AM.; Lladó, I. Phytotherapy in a rat model of hyperoxaluria: the antioxidant effects of quercetin involve serum paraoxonase 1 activation. *Exp Biol Med.* **2011**, *236*, 1133-1138. https://doi.org.10.1258/ebm.2011.011090.
[279] Yunus, J.; Salman M, M.; Lintin, G.B.R.; Muchtar, M.; Sari, D.C.R.; Arfian, N.; Romi, M.M. Chlorogenic acid attenuates kidney fibrosis via antifibrotic action of BMP-7 and HGF. *Med J Malaysia*. **2020**, *75*, 5-9.
[280] Arfian, N.; Wahyudi, D.A.P.; Zulfatina, I.B.; Citta, A.N.; Anggorowati, N.; Multazam, A.; Romi, M.M.; Sari, D.C.R. Chlorogenic acid attenuates kidney ischemic/reperfusion injury via reducing inflammation, tubular injury, and myofibroblast formation. *Biomed Res Int*. **2019**, 5423703. https://doi.org.10.1155/2019/5423703.
[281] Owumi, S.E.; Olusola, J.K.; Arunsi, U.O.; Oyelere, A.K. Chlorogenic acid abates oxido-inflammatory and apoptotic responses in the liver and kidney of Tamoxifen-treated rats. *Toxicol Res (Camb)*. **2021**, *10*, 345-353. https://doi.org.10.1093/toxres/tfab002.
[282] Feng, Y.; Yu, Y.H.; Wang, S.T.; Ren, J.; Camer, D.; Hua, Y.Z.; Zhang, Q.; Huang, J.; Xue, D.L.; Zhang, X.F.; Huang, X.F.; Liu, Y. Chlorogenic acid protects D-galactose-induced liver and kidney injury via antioxidation and anti-inflammation effects in mice. *Pharm Biol.* **2016**, *54*, 1027-1034. https://doi.org.10.3109/13880209.2015.1093510.

[283] Ye, H.Y.; Jin, J.; Jin, L.W.; Chen, Y.; Zhou, Z.H.; Li, Z.Y. Chlorogenic acid attenuates lipopolysaccharide-induced acute kidney injury by inhibiting TLR4/NF-κB signal pathway. *Inflammation*. **2017**, *40*, 523-529. https://doi.org.10.1007/s10753-016-0498-9.
[284] Domitrović, R. Cvijanović, O.; Šušnić, V.; Katalinić, N. Renoprotective mechanisms of chlorogenic acid in cisplatin-induced kidney injury. *Toxicology*. **2014**, *324*, 98-107. https://doi.org.10.1016/j.tox.2014.07.004.
[285] Zhang, T.; Chen, S.; Chen, L.; Zhang, L.; Meng, F.; Sha, S.; Ai, C.; Tai, J. Chlorogenic acid ameliorates lead-induced renal damage in mice. *Biol Trace Elem Res*. **2019**, *189*, 109-117. https://doi.org.10.1007/s12011-018-1508-6.
[286] Qu, S.; Dai, C.; Hao, Z.; Tang, Q.; Wang, H.; Wang, J.; Zhao, H. Chlorogenic acid prevents vancomycin-induced nephrotoxicity without compromising vancomycin antibacterial properties. *Phytother Res*. **2020**, *34*, 3189-3199. https://doi.org.10.1002/ptr.6765.
[287] Zhou, X.; Zhang, B.; Zhao, X.; Lin, Y.; Zhuang, Y.; Guo, J.; Wang, S. Chlorogenic acid prevents hyperuricemia nephropathy via regulating TMAO-related gut microbes and inhibiting the PI3K/AKT/mTOR pathway. *J Agric Food Chem*. **2022**, *70*, 10182-10193. https://doi.org.10.1021/acs.jafc.2c03099.
[288] Zhou, X.; Zhang, B.; Zhao, X.; Lin, Y.; Wang, J.; Wang, X.; Hu, N.; Wang, S. Chlorogenic acid supplementation ameliorates hyperuricemia, relieves renal inflammation, and modulates intestinal homeostasis. *Food Funct*. **2021**, *12*, 5637-5649. https://doi.org.10.1039/d0fo03199b.
[289] Al-Megrin, W.A.; Metwally, D.M.; Habotta, O.A.; Amin, H.K.; Abdel Moneim, A.E.; El-Khadragy, M. Nephroprotective effects of chlorogenic acid against sodium arsenite-induced oxidative stress, inflammation, and apoptosis. *J Sci Food Agric*. **2020**, *100*, 5162-5170. https://doi.org.10.1002/jsfa.10565.
[290] Ye, H.Y.; Li, Z.Y.; Zheng, Y.; Chen, Y.; Zhou, Z.H.; Jin, J. The attenuation of chlorogenic acid on oxidative stress for renal injury in streptozotocin-induced diabetic nephropathy rats. *Arch Pharm Res*. **2016**, *39*, 989-997. https://doi.org.10.1007/s12272-016-0771-3.
[291] Bao, L.; Li, J.; Zha, D.; Zhang, L.; Gao, P.; Yao, T.; Wu, X. Chlorogenic acid prevents diabetic nephropathy by inhibiting oxidative stress and inflammation through modulation of the Nrf2/HO-1 and NF-κB pathways. *Int Immunopharmacol*. **2018**, *54*, 245-253. https://doi.org.10.1016/j.intimp.2017.11.021.
[292] Cheng, D.; Li, H.; Zhou, J.; Wang, S. Chlorogenic acid relieves lead-induced cognitive impairments and hepato-renal damage via regulating the dysbiosis of the gut microbiota in mice. *Food Funct*. **2019**, *10*, 681-690. https://doi.org.10.1039/c8fo01755g.
[293] Toprak, T.; Sekerci, C.A.; Aydın, H.R.; Ramazanoglu, M.A.; Arslan, F.D.; Basok, B.I.; Kucuk, H.; Kocakgol, H.; Aksoy, H.Z.; Asci, S.S.; Tanıdır, Y. Protective effect of chlorogenic acid on renal ischemia/reperfusion injury in rats. *Arch Ital Urol Androl*. **2020**, *92*. https://doi.org.10.4081/aiua.2020.2.153.
[294] Ding, Y.; Li, X.; Liu, Y.; Wang, S.; Cheng, D. Protection Mechanisms Underlying Oral Administration of Chlorogenic Acid against Cadmium-Induced Hepatorenal Injury Related to Regulating Intestinal Flora Balance. *J Agric Food Chem*. **2021**, *69*, 1675-1683. https://doi.org.10.1021/acs.jafc.0c06698.
[295] Cariddi, L.N.; Escobar, F.M.; Sabini, M.C.; Campra, N.A.; Bagnis, G.; Decote-Ricardo, D.; Freire-de-Lima, C.G.; Mañas, F.; Sabini, L.I.; Dalcero, A.M. Phenolic acid protects of renal damage induced by ochratoxin A in a 28-days-oral treatment in rats. *Environ Toxicol Pharmacol*. **2016**, *43*, 105-111. https://doi.org.10.1016/j.etap.2016.03.004.
[296] Yuan, X.; Tang, W.; Lin, C.; He, H.; Li, L. Chrysophanol ameliorates oxidative stress and pyroptosis in mice with diabetic nephropathy through the Kelch-like ECH-associated protein 1/nuclear factor erythroid 2-related factor 2 signaling pathway. *Acta Biochim Pol*. **2023**, *70*, 891-897. https://doi.org.10.18388/abp.2020_6778.
[297] Babaeenezhad, E.; Nouryazdan, N.; Nasri, M.; Ahmadvand, H.; Moradi Sarabi, M. Cinnamic acid ameliorates gentamicin-induced liver dysfunctions and nephrotoxicity in rats through induction of antioxidant activities. *Heliyon*. **2021**, *7*, e07465. https://doi.org.10.1016/j.heliyon.2021.e07465.

[298] el-S.M, El-Sayed.; Abd El-Raouf, O.M.; Fawzy, H.M.; Manie, M.F. Comparative study of the possible protective effects of cinnamic acid and cinnamaldehyde on cisplatin-induced nephrotoxicity in rats. *J Biochem Mol Toxicol*. **2013**, *27*, 508-514. https://doi.org.10.1002/jbt.21515.

[299] Zabad, O.M.; Samra, Y.A.; Eissa, L.A. p-Coumaric acid alleviates experimental diabetic nephropathy through modulation of Toll like receptor-4 in rats. *Life Sci.* **2019**, *1*, 238, 116965. https://doi.org.10.1016/j.lfs.2019.116965.

[300] Navaneethan, D.; Rasool, M. p-Coumaric acid, a common dietary polyphenol, protects cadmium chloride-induced nephrotoxicity in rats. *Ren Fail*. **2014**, *36*, 244-251. https://doi.org.10.3109/0886022X.2013.835268.

[301] Ekinci Akdemir, F.N.; Albayrak, M.; Çalik, M.; Bayir, Y.; Gülçin, İ. The protective effects of p-coumaric acid on acute liver and kidney damages induced by cisplatin. *Biomedicines.* **2017**, *5*, 18. https://doi.org.10.3390/biomedicines5020018.

[302] Rafiee, Z.; Moaiedi, M.Z.; Gorji, A.V.; Mansouri, E. p-coumaric acid mitigates doxorubicin-induced nephrotoxicity through suppression of oxidative stress, inflammation and apoptosis. *Arch Med Res*. **2020**, *51*, 32-40. https://doi.org.10.1016/j.arcmed.2019.12.004.

[303] Mozaffari Godarzi, S.; Valizade Gorji, A.; Gholizadeh, B.; Mard, S.A.; Mansouri, E. Antioxidant effect of p-coumaric acid on interleukin 1-β and tumor necrosis factor-α in rats with renal ischemic reperfusion. *Nefrologia.* **2020**, *40*, 311-319. https://doi.org.10.1016/j.nefro.2019.10.003.

[304] Bal, S.S.; Leishangthem, G.D.; Sethi, R.S.; Singh, A. p-coumaric acid ameliorates fipronil induced liver injury in mice through attenuation of structural changes, oxidative stress and inflammation. *Pestic Biochem Physiol*. **2022**, *180*, 104997. https://doi.org. 10.1016/j.pestbp.2021.104997.

[305] Li, Y.X.; Lu, Y.P.; Tang, D.; Hu, B.; Zhang, Z.Y.; Wu, H.W.; Fan, L.J.; Cai, K.W.; Tang, C.; Zhang, Y.Q.; Hong, L.; Dong, J.J.; Guan, B.Z.; Yin, L.H.; Dai, Y.; Bai, W.B.; Zheng, Z.H.; Zhu, T. Anthocyanin improves kidney function in diabetic kidney disease by regulating amino acid metabolism. *J Transl Med.* **2022**, *20*, 510. https://doi.org.10.1186/s12967-022-03717-9.

[306] Qin, Y.; Zhai, Q.; Li, Y.; Cao, M.; Xu, Y.; Zhao, K.; Wang, T. Cyanidin-3-O-glucoside ameliorates diabetic nephropathy through regulation of glutathione pool. *Biomed Pharmacother.* **2018**, *103*, 1223-1230. https://doi.org.10.1016/j.biopha.2018.04.137.

[307] Sorrenti, V.; Di Giacomo, C.; Acquaviva, R.; Bognanno, M.; Grilli, E.; D'Orazio, N.; Galvano, F. Dimethylarginine dimethylaminohydrolase/nitric oxide synthase pathway in liver and kidney: protective effect of cyanidin 3-O-β-D-glucoside on ochratoxin-A toxicity. *Toxins*. **2012**, *4*, 353-363. https://doi.org.10.3390/toxins4050353.

[308] Zheng, H.X.; Qi, S.S.; He, J.; Hu, C.Y.; Han, H.; Jiang, H.; Li, X.S. Cyanidin-3-glucoside from black rice ameliorates diabetic nephropathy via reducing blood glucose, suppressing oxidative stress and inflammation, and regulating transforming growth factor β1/Smad expression. *J Agric Food Chem.* **2020**, *68*, 4399-4410. https://doi.org.10.1021/acs.jafc.0c00680.

[309] Di Giacomo, C.; Acquaviva, R.; Piva, A.; Sorrenti, V.; Vanella, L.; Piva, G.; Casadei, G.; La Fauci, L.; Ritieni, A.; Bognanno, M.; Di Renzo, L.; Barcellona, M.L.; Morlacchini, M.; Galvano, F. Protective effect of cyanidin 3-O-beta-D-glucoside on ochratoxin A-mediated damage in the rat. *Br J Nutr*. 2007, *98*, 937-943. https://doi.org.10.1017/S0007114507756908.

[310] Cuevas-Magaña, M.Y.; Vega-García, C.C.; León-Contreras, J.C.; Hernández-Pando, R.; Zazueta, C.; García-Niño, W.R. Ellagic acid ameliorates hexavalent chromium-induced renal toxicity by attenuating oxidative stress, suppressing TNF-α and protecting mitochondria. *Toxicol Appl Pharmacol*. **2022**, *454*, 116242. https://doi.org.10.1016/j.taap.2022.116242.

[311] Ayhanci, A.; Cengiz, M.; Mehtap Kutlu, H.; Vejselova, D. Protective effects of ellagic acid in D-galactosamine-induced kidney damage in rats. *Cytotechnol*. **2016**, *68*, 1763-1770. https://doi.org.10.1007/s10616-015-9928-z.

[312] Akkoyun, H.T.; Karadeniz, A. Investigation of the protective effect of ellagic acid for preventing kidney injury in rats exposed to nicotine during the fetal period. *Biotech Histochem*. **2016**, *91*, 108-115. https://doi.org.10.3109/10520295.2015.1078910.
[313] Chen, S.; Zhou, M.; Ying, X.; Zhou, C. Ellagic acid protects rats from chronic renal failure via MiR-182/FOXO3a axis. Mol *Immunol.* **2021**, *138*, 150-160. https://doi.org.10.1016/j.molimm.2021.08.007.
[314] Liu, Q.; Liang, X.; Liang, M.; Qin, R.; Qin, F.; Wang, X. Ellagic acid ameliorates renal ischemic-reperfusion injury through NOX4/JAK/STAT signaling pathway. *Inflammation*. **2020**, *43*, 298-309. https://doi.org.10.1007/s10753-019-01120-z.
[315] Sepand, M.R.; Ghahremani, M.H.; Razavi-Azarkhiavi, K.; Aghsami, M.; Rajabi, J.; Keshavarz-Bahaghighat, H.; Soodi, M. Ellagic acid confers protection against gentamicin-induced oxidative damage, mitochondrial dysfunction and apoptosis-related nephrotoxicity. *J Pharm Pharmacol.* **2016**, *68*, 1222-1232. https://doi.org.10.1111/jphp.12589.
[316] Naghibi, N.; Sadeghi, A.; Movahedinia, S.; Rahimi Naiini, M.; Rajizadeh, M.A.; Bahri, F.; Nazari-Robati, M. Ellagic acid ameliorates aging-induced renal oxidative damage through upregulating SIRT1 and NRF2. BMC Complement Med Ther. **2023**, *23*, 77. https://doi.org.10.1186/s12906-023-03907-y.
[317] Sun, Z.R.; Liu, H.R.; Hu, D.; Fan, M.S.; Wang, M.Y.; An, M.F.; Zhao, Y.L.; Xiang, Z.M.; Sheng, J. Ellagic acid exerts beneficial effects on hyperuricemia by inhibiting xanthine oxidase and NLRP3 inflammasome activation. *J Agric Food Chem*. **2021**, *69*, 12741-12752. https://doi.org.10.1021/acs.jafc.1c05239.
[318] Bhattacharjee, A.; Kulkarni, V.H.; Chakraborty, M.; Habbu, P.V.; Ray, A. Ellagic acid restored lead-induced nephrotoxicity by anti-inflammatory, anti-apoptotic and free radical scavenging activities. *Heliyon.* **2021**, *7*, e05921. https://doi.org.10.1016/j.heliyon.2021.e05921.
[319] El-Garhy, A.M.; Abd El-Raouf, O.M.; El-Sayeh, B.M.; Fawzy, H.M.; Abdallah, D.M. Ellagic acid antiinflammatory and antiapoptotic potential mediate renoprotection in cisplatin nephrotoxic rats. *J Biochem Mol Toxicol*. **2014**, *28*, 472-479. https://doi.org.10.1002/jbt.21587.
[320] Ateşşahín, A.; Ceríbaşi, A.O.; Yuce, A.; Bulmus, O.; Cikim, G. Role of ellagic acid against cisplatin-induced nephrotoxicity and oxidative stress in rats. *Basic Clin Pharmacol Toxicol*. **2007**, *100*, 121-126. https://doi.org.10.1111/j.1742-7843.2006.00015.x.
[321] Vijaya Padma, V.; Kalai Selvi, P.; Sravani, S. Protective effect of ellagic acid against TCDD-induced renal oxidative stress: modulation of CYP1A1 activity and antioxidant defense mechanisms. *Mol Biol Rep.* **2014**, *41*, 4223-4232. https://doi.org.10.1007/s11033-014-3292-5.
[322] Zhou, B.; Li, Q.; Wang, J.; Chen, P.; Jiang, S. Ellagic acid attenuates streptozocin induced diabetic nephropathy via the regulation of oxidative stress and inflammatory signaling. *Food Chem Toxicol.* **2019**, *Jan;123*, 16-27. https://doi.org.10.1016/j.fct.2018.10.036.
[323] Yüce, A.; Ateşşahin, A.; Ceribaşi, A.O. Amelioration of cyclosporine A-induced renal, hepatic and cardiac damages by ellagic acid in rats. *Basic Clin Pharmacol Toxicol*. **2008**, *103*, 186-191. https://doi.org.10.1111/j.1742-7843.2008.00284.x.
[324] Shabani, M.; Bayrami, D.; Moghadam, A.A.; Jamali, Z.; Salimi, A. Pretreatment of ellagic acid protects ifosfamide-induced acute nephrotoxicity in rat kidneys: A mitochondrial, histopathological and oxidative stress approaches. *Toxicol Rep.* **2023**, *10,* 441-447. https://doi.org.10.1016/j.toxrep.2023.04.005.
[325] Sharma, V.C.; Kaushik, A.; Dey, Y.N.; Srivastava, B.; Wanjari, M.; Pawar, S.; Chougule, S. Nephroprotective potential of Anogeissus latifolia Roxb. (Dhava) against gentamicin-induced nephrotoxicity in rats. *J Ethnopharmacol.* **2021**, *273*, 114001. https://doi.org.10.1016/j.jep.2021.114001.
[326] Rehman, M.U.; Tahir, M.; Ali, F.; Qamar, W.; Lateef, A.; Khan, R.; Quaiyoom, A. Oday-O-Hamiza, S. Sultana. Cyclophosphamide-induced nephrotoxicity, genotoxicity, and damage in kidney genomic DNA of Swiss albino mice: the protective effect of ellagic acid. *Mol Cell Biochem*. **2012**, *365*, 119-127. https://doi.org.10.1007/s11010-012-1250-x.

[327] Liu, J.; Sun, Y.; Zheng, H.; Wang, J.; Liu, L.; Song, B.; Zhang, H. Emodin attenuated the kidney damage of high-fat-diet mice via the upregulation of glucagon-like peptide-1 receptor. *Biomed Res Int*. **2021**, 6662704. https://doi.org.10.1155/2021/6662704.
[328] Liu, H.; Wang, Q.; Shi, G.; Yang, W.; Zhang, Y.; Chen, W.; Wan, S.; Xiong, F.; Wang, Z. Emodin ameliorates renal damage and podocyte injury in a rat model of diabetic nephropathy via regulating AMPK/mTOR-mediated autophagy signaling pathway. *Diabetes Metab Syndr Obes*. **2021**, *14*, 1253-1266. https://doi.org.10.2147/DMSO.S299375.
[329] Zeng, Y.Q.; Dai, Z.; Lu, F.; Lu, Z.; Liu, X.; Chen, C.; Qu, P.; Li, D.; Hua, Z.; Qu, Y.; Zou, C. Emodin via colonic irrigation modulates gut microbiota and reduces uremic toxins in rats with chronic kidney disease. *Oncotarget*. **2016**, *7*, 17468-17478. https://doi.org.10.18632/oncotarget.8160.
[330] Ali, B.H.; Al-Salam, S.; Al Husseini, I.S.; Al-Lawati, I.; Waly, M.; Yasin, J.; Fahim, M.; Nemmar, A. Abrogation of cisplatin-induced nephrotoxicity by emodin in rats. *Fundam Clin Pharmacol*. **2013**, *27*, 192-200. https://doi.org.10.1111/j.1472-8206.2011.01003.x.
[331] Jing, D.; Bai, H.; Yin, S. Renoprotective effects of emodin against diabetic nephropathy in rat models are mediated via PI3K/Akt/GSK-3β and Bax/caspase-3 signaling pathways. *Exp Ther Med*. **2017**, *14*, 5163-5169. https://doi.org.10.3892/etm.2017.5131.
[332] Prince, P.D; Fischerman, L.; Toblli, J.E.; Fraga, C.G.; Galleano, M. LPS-induced renal inflammation is prevented by (-)-epicatechin in rats. *Redox Biol*. **2017**, *11*, 342-349. https://doi.org.10.1016/j.redox.2016.12.023.
[333] Prince, P.D.; Lanzi, C.R.; Toblli, J.E.; Elesgaray, R.; Oteiza, P.I.; Fraga, C.G.; Galleano, M. Dietary (-)-epicatechin mitigates oxidative stress, NO metabolism alterations, and inflammation in renal cortex from fructose-fed rats. *Free Radic Biol Med*. **2016**, *90*, 35-46. https://doi.org.10.1016/j.freeradbiomed.2015.11.009.
[334] Montes-Rivera, J.; Arellano-Mendoza, M.; Nájera, N.; Del Valle-Mondragón, L.; Villarreal, F.; Rubio-Gayosso, I.; Perez-Duran, J.; Meaney, E.; Ceballos, G. Effect of (-)-epicatechin on the modulation of progression markers of chronic renal damage in a 5/6 nephrectomy experimental model. *Heliyon*. **2019**, *5*, e01512. https://doi.org.10.1016/j.heliyon.2019.e01512.
[335] Grases, F.; Prieto, R.M.; Fernandez-Cabot, R.A.; Costa-Bauzá, A.; Tur, F.; Torres, J.J. Effects of polyphenols from grape seeds on renal lithiasis. *Oxid Med Cell Longev*. **2015**, 813737. https://doi.org.10.1155/2015/813737.
[336] Malik, S.; Suchal, K.; Bhatia, J.; Gamad, N.; Dinda, A.K.; Gupta, Y.K.; Arya, D.S. Molecular mechanisms underlying attenuation of cisplatin-induced acute kidney injury by epicatechin gallate. *Lab Invest*. **2016**, *96*, 853-861. https://doi.org.10.1038/labinvest.2016.60.
[337] Peng, A.; Ye, T.; Rakheja, D.; Tu, Y.; Wang, T.; Du, Y.; Zhou, J.K.; Vaziri, N.D.; Hu, Z.; Mohan, C.; Zhou, X.J. The green tea polyphenol (-)-epigallocatechin-3-gallate ameliorates experimental immune-mediated glomerulonephritis. *Kidney Int*. **2011**, *80*, 601-611. https://doi.org.10.1038/ki.2011.121.
[338] Tsai, P.Y.; Ka, S.M.; Chang, J.M.; Chen, H.C.; Shui, H.A.; Li, C.Y.; Hua, K.F.; Chang, W.L.; Huang, J.J.; Yang, S.S.; Chen, A. Epigallocatechin-3-gallate prevents lupus nephritis development in mice via enhancing the Nrf2 antioxidant pathway and inhibiting NLRP3 inflammasome activation. *Free Radic Biol Med*. **2011**, *51*, 744-754. https://doi.org.10.1016/j.freeradbiomed.2011.05.016.
[339] Yamabe, N.; Yokozawa, T.; Oya, T.; Kim, M. Therapeutic potential of (-)-epigallocatechin 3-O-gallate on renal damage in diabetic nephropathy model rats. *J Pharmacol Exp Ther*. **2006**, *319*, 228-236. https://doi.org.10.1124/jpet.106.107029.
[340] Sahin, K.; Tuzcu, M.; Gencoglu, H.; Dogukan, A.; Timurkan, M.; Sahin, N.; Aslan, A.; Kucuk, O. Epigallocatechin-3-gallate activates Nrf2/HO-1 signaling pathway in cisplatin-induced nephrotoxicity in rats. *Life Sci*. **2010**, *87*, 240-245. https://doi.org.10.1016/j.lfs.2010.06.014.

[341] Ye, T.; Zhen, J.; Du, Y.; Zhou, J.K.; Peng, A.; Vaziri, N.D.; Mohan, C.; Xu, Y.; Zhou, X.J. Green tea polyphenol (-)-epigallocatechin-3-gallate restores Nrf2 activity and ameliorates crescentic glomerulonephritis. *PLoS One*. **2015**, *10*, e0119543. DOI: 10.1371/journal.pone.0119543.
[342] Zhou, P.; Yu, J.F.; Zhao, C.G.; Sui, F.X.; Teng, X.; Wu, Y.B. Therapeutic potential of EGCG on acute renal damage in a rat model of obstructive nephropathy. *Mol Med Rep.* **2013**, *7*, 1096-1102. DOI: 10.3892/mmr.2013.1296.
[343] F.T. Hammad, L. Lubbad. The effect of epigallocatechin-3-gallate on the renal dysfunction in the obstructed kidney in the rat. Int J Physiol Pathophysiol Pharmacol. (2017) Sep;9(4):119-126.
[344] Yang, R.; Chen, J.; Jia, Q.; Yang, X.; Mehmood, S. Epigallocatechin-3-gallate ameliorates renal endoplasmic reticulum stress-mediated inflammation in type 2 diabetic rats. *Exp Biol Med (Maywood)*. **2022**, *Aug;247(16)*, 1410-1419. https://doi.org.10.1177/15353702221106479.
[345] Chen, J.; Du, L.; Li, J.; Song, H. Epigallocatechin-3-gallate attenuates cadmium-induced chronic renal injury and fibrosis. *Food Chem Toxicol.* **2016**, *96,* 70-78. https://doi.org.10.1016/j.fct.2016.07.030.
[346] Nakagawa, T.; Yokozawa, T.; Sano, M.; Takeuchi, S.; Kim, M.; Minamoto, S. Activity of (-)-epigallocatechin 3-O-gallate against oxidative stress in rats with adenine-induced renal failure. *J Agric Food Chem*. **2004**, *52*, 2103-2107. https://doi.org.10.1021/jf030258j.
[347] Yoon, S.P.; Maeng, Y.H.; Hong, R.; Lee, B.R.; Kim, C.G.; Kim, H.L.; Chung, J.H.; Shin, B.C. Protective effects of epigallocatechin gallate (EGCG) on streptozotocin-induced diabetic nephropathy in mice. *Acta Histochem*. **2014**, *116*, 1210-1215. https://doi.org.10.1016/j.acthis.2014.07.003.
[348] Alam, M.A.; Sernia, C.; Brown, L. Ferulic acid improves cardiovascular and kidney structure and function in hypertensive rats. *J Cardiovasc Pharmacol.* **2013**, *61*, 240-249. https://doi.org.10.1097/FJC.0b013e31827cb600.
[349] Zhou, Q.; Gong, X.; Kuang, G.; Jiang, R.; Xie, T.; Tie, H.; Chen, X.; Li, K.; Wan, J.; Wang, B. Ferulic acid protected from kidney ischemia reperfusion injury in mice: possible mechanism through increasing adenosine generation via HIF-1α. *Inflammation.* **2018**, *41*, 2068-2078. https://doi.org.10.1007/s10753-018-0850-3.
[350] Erseçkin, V.; Mert, H.; İrak, K.; Yildirim, S.; Mert, N. Nephroprotective effect of ferulic acid on gentamicin-induced nephrotoxicity in female rats. *Drug Chem Toxicol.* **2022**, *45*, 663-669. https://doi.org.10.1080/01480545.2020.1759620.
[351] Nouri, A.; Ghatreh-Samani, K.; Amini-Khoei, H.; Mohammadi, A.; Heidarian, E.; Najafi, M. Ferulic acid prevents cyclosporine-induced nephrotoxicity in rats through exerting anti-oxidant and anti-inflammatory effects via activation of Nrf2/HO-1 signaling and suppression of NF-κB/TNF-α axis. *Naunyn Schmiedebergs Arch Pharmacol.* **2022**, *395*, 387-395. https://doi.org.10.1007/s00210-022-02212-8.
[352] Shokeir, A.A.; Hussein, A.A.; Soliman, S.A.; Kamal, M.M.; Abdel-Aziz, A.; Awadalla, A.; Rahim, M.A.; Barakat, N. Recoverability of renal functions after relief of partial ureteric obstruction of solitary kidney: impact of ferulic acid. *BJU Int.* **2012**, *110*, 904-911. https://doi.org.10.1111/j.1464-410X.2011.10848.x.
[353] Mir, S.M.; Ravuri, H.G.; Pradhan, R.K.; Narra, S.; Kumar, J.M.; Kuncha, M.; Kanjilal, S.; Sistla, R. Ferulic acid protects lipopolysaccharide-induced acute kidney injury by suppressing inflammatory events and upregulating antioxidant defenses in Balb/c mice. *Biomed Pharmacother*. **2018**, *100*, 304-315. https://doi.org.10.1016/j.biopha.2018.01.169.
[354] Ma, R.; He, Y.; Fang, Q. Xie, G. Qi, M. Ferulic acid ameliorates renal injury via improving autophagy to inhibit inflammation in diabetic nephropathy mice. *Biomed Pharmacother*. **2022**, *153*, 113424. https://doi.org.10.1016/j.biopha.2022.113424.

[355] Bami, E.; Ozakpınar, O.B.; Ozdemir-Kumral, Z.N.; Köroglu, K.; Ercan, F.; Cirakli, Z.; Sekerler, T.; Izzettin, F.V.; Sancar, M.; Okuyan, B. Protective effect of ferulic acid on cisplatin induced nephrotoxicity in rats. *Environ Toxicol Pharmacol.* **2017**, *54*, 105-111. https://doi.org.10.1016/j.etap.2017.06.026.
[356] Zhang, N.; Zhou, J.; Zhao, L.; Wang, O.; Zhang, L.; Zhou, F. Dietary ferulic acid ameliorates metabolism syndrome-associated hyperuricemia in rats *via* regulating uric acid synthesis, glycolipid metabolism, and hepatic injury. *Front Nutr.* **2022**, *9*, 946556. https://doi.org.10.3389/fnut.2022.946556.
[357] Manikandan, R.; Beulaja, M.; Thiagarajan, R.; Pandi, M.; Arulvasu, C.; Prabhu, N.M.; Saravanan, R.; Esakkirajan, M.; Palanisamy, S.; Dhanasekaran, G.; Nisha, R.G.; Devi, K.; Latha, M. Ameliorative effect of ferulic acid against renal injuries mediated by nuclear factor-kappaB during glycerol-induced nephrotoxicity in Wistar rats. *Ren Fail.* 2014, *36*, 154-165. https://doi.org.10.3109/0886022X.2013.835223.
[358] Ren, Q.; Guo, F.; Tao, S.; Huang, R.; Ma, L.; Fu, P. Flavonoid fisetin alleviates kidney inflammation and apoptosis via inhibiting Src-mediated NF-κB p65 and MAPK signaling pathways in septic AKI mice. *Biomed Pharmacother.* **2020**, *122*, 109772. https://doi.org.10.1016/j.biopha.2019.109772.
[359] Ren, Q.; Cheng, L.; Guo, F.; Tao, S.; Zhang, C.; Ma, L.; Fu, P. Fisetin improves hyperuricemia-induced chronic kidney disease via regulating gut microbiota-mediated tryptophan metabolism and aryl hydrocarbon receptor activation. *J Agric Food Chem*. **2021**, *69*, 10932-10942. https://doi.org.10.1021/acs.jafc.1c03449.
[360] Prem, P.N.; Kurian, G.A. Fisetin attenuates renal ischemia/reperfusion injury by improving mitochondrial quality, reducing apoptosis and oxidative stress. *Naunyn Schmiedebergs Arch Pharmacol*. **2022**, *395*, 547-561. https://doi.org.10.1007/s00210-022-02204-8.
[361] Chenxu, G.; Xianling, D.; Qin, K.; Linfeng, H.; Yan, S.; Mingxin, X.; Jun, T.; Minxuan, X. Fisetin protects against high fat diet-induced nephropathy by inhibiting inflammation and oxidative stress via the blockage of iRhom2/NF-κB signaling. *Int Immunopharmacol.* **2021**, *92*, 107353. https://doi.org.10.1016/j.intimp.2020.107353.
[362] Sahu, B.D.; Kalvala, A.K.; Koneru, M.; Mahesh Kumar, J.; Kuncha, M.; Rachamalla, S.S.; Sistla, R. Ameliorative effect of fisetin on cisplatin-induced nephrotoxicity in rats via modulation of NF-κB activation and antioxidant defense. *PLoS One.* **2014**, *9*, e105070. https://doi.org/10.1371/journal.pone.0105070.
[363] Berköz, M.; Yiğit, A.; Krośniak, M. Protective role of myricetin and fisetin against nephrotoxicity caused by lead acetate exposure through up-regulation of Nrf2/HO-1 signalling pathway. *Biol Trace Elem Res.* **2024**, *202*, 4032-4046. https://doi.org. 10.1007/s12011-023-03977-6.
[364] Oza, M.J.; Kulkarni, Y.A. Formononetin attenuates kidney damage in type 2 diabetic rats. *Life Sci.* **2019**, *219*, 109-121. https://doi.org.10.1016/j.lfs.2019.01.013.
[365] Hao, Y.; Miao, J.; Liu, W.; Peng, L.; Chen, Y.; Zhong Q. Formononetin protects against cisplatin-induced acute kidney injury through activation of the PPARα/Nrf2/HO-1/NQO1 pathway. *Int J Mol Med*. **2021**, *47*, 511-522. https://doi.org.10.3892/ijmm.2020.4805.
[366] Aladaileh, S.H.; Hussein, O.E.; Abukhalil, M.H.; Saghir, S.A.M.; Bin-Jumah, M.; Alfwuaires, M.A.; Germoush, M.O.; Almaiman, A.A.; Mahmoud, A.M. Formononetin upregulates Nrf2/HO-1 signaling and prevents oxidative stress, inflammation, and kidney injury in methotrexate-induced rats. *Antioxidants (Basel).* **2019**, *8*, 430. https://doi.org.10.3390/antiox8100430.
[367] Liu, L.; Hu, R.; You, H.; Li, J.; Liu, Y.; Li, Q.; Wu, X.; Huang, J.; Cai, X.; Wang, M.; Wei L. Formononetin ameliorates muscle atrophy by regulating myostatin-mediated PI3K/Akt/FoxO3a pathway and satellite cell function in chronic kidney disease. *J Cell Mol Med*. **2021**, *25,* 1493-1506. https://doi.org.10.1111/jcmm.16238.

[368] Althunibat, O.Y.; Abukhalil, M.H.; Aladaileh, S.H.; Qaralleh, H.; Al-Amarat, W.; Alfwuaires, M.A.; Algefare, A.I.; Namazi, N.I.; Melebary, S.J.; Babalghith, A.O.; Conte-Junior, C.A. Formononetin ameliorates renal dysfunction, oxidative stress, inflammation, and apoptosis and upregulates Nrf2/HO-1 signaling in a rat model of gentamicin-induced nephrotoxicity. *Front Pharmacol*. **2022**, *13*, 916732. https://doi.org.10.3389/fphar.2022.916732.
[369] Lv, J.; Zhuang, K.; Jiang, X.; Huang, H.; Quan, S. Renoprotective effect of formononetin by suppressing Smad3 expression in Db/Db mice. *Diabetes Metab Syndr Obes*. **2020**, *13,* 3313-3324. https://doi.org.10.2147/DMSO.S272147.
[370] Asci, H.; Ozmen, O.; Ellidag, H.Y.; Aydin, B.; Bas, E.; Yilmaz, N. The impact of gallic acid on the methotrexate-induced kidney damage in rats. *J Food Drug Anal*. **2017**, *25,* 890-897. https://doi.org.10.1016/j.jfda.2017.05.001.
[371] M.S. Garud, Kulkarni, Y.A. Gallic acid attenuates type I diabetic nephropathy in rats. *Chem Biol Interact*. **2018**, *282,* 69-76. https://doi.org.10.1016/j.cbi.2018.01.010.
[372] Nouri, A.; Heibati, F.; Heidarian, E. Gallic acid exerts anti-inflammatory, anti-oxidative stress, and nephroprotective effects against paraquat-induced renal injury in male rats. *Naunyn Schmiedebergs Arch Pharmacol*. **2021**, *394,* 1-9. https://doi.org.10.1007/s00210-020-01931-0.
[373] Mojadami, S.; Ahangarpour, A.; Mard, S.A.; Khorsandi, L. Diabetic nephropathy induced by methylglyoxal: gallic acid regulates kidney microRNAs and glyoxalase1-Nrf2 in male mice. *Arch Physiol Biochem*. **2023**, *129,* 655-662. https://doi.org.10.1080/13813455.2020.1857775.
[374] Doğan, D.; Meydan, İ.; Kömüroğlu, A.U. Protective effect of silymarin and gallic acid against cisplatin-induced nephrotoxicity and hepatotoxicity. *Int J Clin Pract*. **2022**, *2022*, 6541026. https://doi.org.10.1155/2022/6541026.
[375] Alhazmi, A.I.; El-Refaei, M.F.; Abdallah, E.A.A. Protective effects of gallic acid against nickel-induced kidney injury: impact of antioxidants and transcription factor on the incidence of nephrotoxicity. *Ren Fail*. **2024**, *46*, 2344656. https://doi.org.10.1080/0886022X.2024.2344656.
[376] Singh, J.P.; Singh, A.P.; Bhatti, R. Explicit role of peroxisome proliferator-activated receptor gamma in gallic acid-mediated protection against ischemia-reperfusion-induced acute kidney injury in rats. *J Surg Res*. **2014**, *187*, 631-639. https://doi.org.10.1016/j.jss.2013.11.1088.
[377] Gholamine, B.; Houshmand, G.; Hosseinzadeh, A.; Kalantar, M.; Mehrzadi, S.; Goudarzi, M. Gallic acid ameliorates sodium arsenite-induced renal and hepatic toxicity in rats. *Drug Chem Toxicol*. **2021**, *44,* 341-352. https://doi.org.10.1080/01480545.2019.1591434.
[378] Nabavi, S.M.; Habtemariam, S.; Nabavi, S.F.; Sureda, A.; Daglia, M.; Moghaddam, A.H.; Amani, M.A. Protective effect of gallic acid isolated from Peltiphyllum peltatum against sodium fluoride-induced oxidative stress in rat's kidney. *Mol Cell Biochem*. **2013**, *372,* 233-239. https://doi.org.10.1007/s11010-012-1464-y.
[379] Moradi, A.; Abolfathi, M.; Javadian, M.; Heidarian, E.; Roshanmehr, H.; Khaledi, M.; Nouri, A. Gallic acid exerts nephroprotective, anti-oxidative stress, and anti-inflammatory effects against diclofenac-induced renal injury in male rats. *Arch Med Res*. **2021**, *52*, 380-388. https://doi.org.10.1016/j.arcmed.2020.12.005.
[380] Reckziegel, P.; Dias, V.T.; Benvegnú, D.M.; Boufleur, N.; Barcelos, R.C.S.; Segat, H.J.; Pase, C.S.; Santos, C.M.M.; Flores, É.M.M.; Bürger, M.E. Antioxidant protection of gallic acid against toxicity induced by Pb in blood, liver and kidney of rats. *Toxicol Rep*. **2016**, *3,* 351-356. https://doi.org.10.1016/j.toxrep.2016.02.005.
[381] Eslamifar, Z.; Moridnia, A.; Sabbagh, S.; Ghaffaripour, R.; Jafaripour, L.; Behzadifard, M. Ameliorative effects of gallic acid on cisplatin-induced nephrotoxicity in rat variations of biochemistry, histopathology, and gene expression. *Biomed Res Int*. **2021**, 2195238. https://doi.org.10.1155/2021/2195238.

[382] Ghaznavi, H.; Fatemi, I.; Kalantari, H.; Hosseini Tabatabaei, S.M.T.; Mehrabani, M.; Gholamine, B.; Kalantar, M.; Mehrzadi, S.; Goudarzi. M. Ameliorative effects of gallic acid on gentamicin-induced nephrotoxicity in rats. *J Asian Nat Prod Res.* **2018**, *20,* 1182-1193. https://doi.org.10.1080/10286020.2017.1384819.

[383] Amini, N.; Badavi, M.; Mard, S.A.; Dianat, M.; Moghadam, M.T. The renoprotective effects of gallic acid on cisplatin-induced nephrotoxicity through anti-apoptosis, anti-inflammatory effects, and downregulation of lncRNA TUG1. *Naunyn Schmiedebergs Arch Pharmacol.* **2022**, *395*, 691-701. https://doi.org.10.1007/s00210-022-02227-1.

[384] Owumi, S.; Najophe, E.S.; Farombi, E.O.; Oyelere, A.K. Gallic acid protects against Aflatoxin B_1 -induced oxidative and inflammatory stress damage in rats kidneys and liver. *J Food Biochem.* **2020**, *44*, e13316. https://doi.org.10.1111/jfbc.13316.

[385] Obafemi, T.O. Gallic acid and hesperidin ameliorate electrolyte imbalances in $AlCl_3$-induced nephrotoxicity in Wistar rats. *Biochem Res Int.* **2022**, 6151684. https://doi.org.10.1155/2022/6151684.

[386] Saleh, S.M.M.; Mahmoud, A.B.; Al-Salahy, M.B.; Moustafa, F.A.M. Morphological, immunohistochemical, and biochemical study on the ameliorative effect of gallic acid against bisphenol A-induced nephrotoxicity in male albino rats. *Sci Rep.* **2023**, *13*, 1732. https://doi.org.10.1038/s41598-023-28860-1.

[387] Yousuf, M.J.; Vellaichamy, E. Protective activity of gallic acid against glyoxal -induced renal fibrosis in experimental rats. *Toxicol Rep.* **2015**, *2*, 1246-1254. https://doi.org.10.1016/j.toxrep.2015.07.007.

[388] Ahmadvand, H.; Yalameha, B.; Adibhesami, G.; Nasri, M.; Naderi, N.; Babaeenezhad, E.; Nouryazdan, N. The protective role of gallic acid pretreatment on renal ischemia-reperfusion injury in rats. Rep Biochem Mol Biol. **2019**, *8*, 42-48.

[389] Oyagbemi, A.A.; Akinrinde, A.S.; Adebiyi, O.E.; Jarikre, T.A.; Omobowale, T.O.; Ola-Davies, O.E.; Saba, A.B.; Emikpe, B.O.; Adedapo, A.A. Luteolin supplementation ameliorates cobalt-induced oxidative stress and inflammation by suppressing NF-κB/Kim-1 signaling in the heart and kidney of rats, Env. Toxicol. *Pharmacol*. **2020**, *80*, 103488. https://doi.org/10.1016/j.etap.2020.103488.

[390] Saeedavi, M.; Goudarzi, M.; Fatemi, I.; Basir, Z.; Noori, S.M.A.; Mehrzadi, S. Gentisic acid mitigates gentamicin-induced nephrotoxicity in rats. *Tissue Cell.* **2023**, *84*, 102191. https://doi.org.10.1016/j.tice.2023.102191.

[391] Noei Razliqi, R.; Ahangarpour, A.; Mard, S.A.; Khorsandi, L. Gentisic acid protects against diabetic nephropathy in nicotinamide-streptozotocin administered male mice by attenuating oxidative stress and inflammation: The role of miR-200a/Keap1/Nrf2 pathway, renin-angiotensin system (RAS) and NF-κB. *Chem Biol Interact.* **2023**, *380*, 110507. https://doi.org.10.1016/j.cbi.2023.110507.

[392] Das Neves, R.N.; Carvalho, F.; Carvalho, M.; Fernandes, E.; Soares, E.; Bastos, M.L.; Pereira, M.L. Protective activity of hesperidin and lipoic acid against sodium arsenite acute toxicity in mice. *Toxicol Pathol.* **2004**, *32*, 527-535. https://doi.org.10.1080/01926230490502566.

[393] Abd-Elhakim, Y.M.; Ghoneim, M.H.; Ebraheim, L.L.M.; Imam, T.S. Taurine and hesperidin rescues carbon tetrachloride-triggered testicular and kidney damage in rats via modulating oxidative stress and inflammation. *Life Sci.* **2020**, *254*, 117782. https://doi.org.10.1016/j.lfs.2020.117782.

[394] Tirkey, N.; Pilkhwal, S.; Kuhad, A.; Chopra, K. Hesperidin, a citrus bioflavonoid, decreases the oxidative stress produced by carbon tetrachloride in rat liver and kidney. *BMC Pharmacol.* 2005, 5, 2. https://doi.org.10.1186/1471-2210-5-2.

[395] Ali, Y.A.; Ahmed, O.M.; Soliman, H.A.; Abdel-Gabbar, M.; Al-Dossari, M.; El-Gawaad, N.S.A.; El-Nahass, E.S.; Ahmed, N.A. Rutin and hesperidin alleviate paclitaxel-induced nephrocardiotoxicity in Wistar rats *via* suppressing the oxidative stress and enhancing the antioxidant defense mechanisms. *Evid Based Complement Alternat Med.* **2023**, 5068304. https://doi.org.10.1155/2023/5068304.

[396] Nandakumar, N.; Balasubramanian, M.P. Hesperidin protects renal and hepatic tissues against free radical-mediated oxidative stress during DMBA-induced experimental breast cancer. *J Environ Pathol Toxicol Oncol.* **2011**, *30*, 283-300. https://doi.org.10.1615/jenvironpatholtoxicoloncol.v30.i4.20.

[397] Anandan, R.; Subramanian, P. Renal protective effect of hesperidin on gentamicin-induced acute nephrotoxicity in male Wistar albino rats. *Redox Rep.* **2012**, *17*, 219-226. https://doi.org.10.1179/1351000212Y.0000000019.

[398] Siddiqi, A.; Hasan, SK.; Nafees, S.; Rashid, S.; Saidullah, B.; Sultana, S. Chemopreventive efficacy of hesperidin against chemically induced nephrotoxicity and renal carcinogenesis via amelioration of oxidative stress and modulation of multiple molecular pathways. *Exp Mol Pathol.* **2015**, *99*, 641-653. https://doi.org.10.1016/j.yexmp.2015.11.012.

[399] Kim, K.; Leem, J. Hispidulin ameliorates endotoxin-induced acute kidney injury in mice. *Molecules.* **2022**, *27*, 2019. https://doi.org.10.3390/molecules27062019.

[400] Li, X.; Gu, Y.; Ren, L.; Cai, Q.; Qiu, Y.; He, J.; Qu, W.; Ji, W. Study of hispidulin in the treatment of uric acid nephropathy based on NF-κB signaling pathway. *Chem Biol Drug Des*. **2024**, *103*, e14367. https://doi.org.10.1111/cbdd.14367.

[401] Zhang, J.; Fu, H.; Xu, Y.; Niu, Y.; An, X. Hyperoside reduces albuminuria in diabetic nephropathy at the early stage through ameliorating renal damage and podocyte injury. *J Nat Med.* **2016**, *70*, 740-748. https://doi.org.10.1007/s11418-016-1007-z.

[402] Chunzhi, G.; Zunfeng, L.; Chengwei, Q.; Xiangmei, B.; Jingui, Y. Hyperin protects against LPS-induced acute kidney injury by inhibiting TLR4 and NLRP3 signaling pathways. *Oncotarget.* **2016**, *7*, 82602-82608. https://doi.org.10.18632/oncotarget.13010.

[403] Jian, J.; Yu-Qing, L.; Rang-Yue, H.; Xia, Z.; Ke-Huan, X.; Ying, Y.; Li, W.; Rui-Zhi, T. Isorhamnetin ameliorates cisplatin-induced acute kidney injury in mice by activating SLPI-mediated anti-inflammatory effect in macrophage. *Immunopharmacol Immunotoxicol.* **2024**, *46*, 319-329. https://doi.org.10.1080/08923973.2024.2329621.

[404] Yang, S.; Wu, H.; Li, Y.; Li, L.; Xiang, J.; Kang, L.; Yang, G.; Liang, Z. Inhibition of PFKP in renal tubular epithelial cell restrains TGF-β induced glycolysis and renal fibrosis. *Cell Death Dis.* **2023**, *14*, 816. https://doi.org.10.1038/s41419-023-06347-1.

[405] Wang, F.; Zhao, X.; Su, X.; Song, D.; Zou, F.; Fang, L. Isorhamnetin, the xanthine oxidase inhibitor from *Sophora japonica*, ameliorates uric acid levels and renal function in hyperuricemic mice. *Food Funct.* **2021**, *12*, 12503-12512. https://doi.org.10.1039/d1fo02719k.

[406] Liu, S.; Zhang, X.; Wang, J. Isovitexin protects against cisplatin-induced kidney injury in mice through inhibiting inflammatory and oxidative responses. *Int Immunopharmacol.* **2020**, *83*, 106437. https://doi.org.10.1016/j.intimp.2020.106437.

[407] Souza, P.; Silva, L.M.; Boeing, T.; Somensi, L.B.; Cechinel-Zanchett, C.C.; Campos, A.; Krueger, C.M.A.; Bastos, J.K.; Cechinel-Filho, V.; Andrade, S.F. Influence of prostanoids in the diuretic and natriuretic effects of extracts and kaempferitrin from *Bauhinia forficata* Link leaves in rats. *Phytother. Res.* **2017**, *31*, 1521-1528. https://doi.org.0.1002/ptr.5876.

[408] Luo, W.; Chen, X.; Ye, L.; Chen, X.; Jia, W.; Zhao, Y.; Samorodov, A.V.; Zhang, Y.; Hu, X.; Zhuang, F.; Qian, J.; Zheng, C.; Liang, G.; Wang, Y. Kaempferol attenuates streptozotocin-induced diabetic nephropathy by downregulating TRAF6 expression: The role of TRAF6 in diabetic nephropathy. *J Ethnopharmacol.* **2021**, *268*, 113553. https://doi.org.10.1016/j.jep.2020.113553.

[409] Xu, Z.; Wang, X.; Kuang, W.; Wang, S.; Zhao, Y. Kaempferol improves acute kidney injury via inhibition of macrophage infiltration in septic mice. *Biosci Rep.* **2023**, *43*, BSR20230873. https://doi.org.10.1042/BSR20230873.

[410] Sheng, H.; Zhang, D.; Zhang, J.; Zhang, Y.; Lu, Z.; Mao, W.; Liu, X.; Zhang, L. Kaempferol attenuated diabetic nephropathy by reducing apoptosis and promoting autophagy through AMPK/mTOR pathways. *Front Med.* **2022**, *9*, 986825. https://doi.org.10.3389/fmed.2022.986825.

[411] Park, M.J.; Lee, E.K.; Heo, H.S.; Kim, M.S.; Sung, B.; Kim, M.K.; Lee, J.; Kim, N.D.; Anton, S.; Choi, J.S.; Yu, B.P.; Chung, H.Y. The anti-inflammatory effect of kaempferol in aged kidney tissues: the involvement of nuclear factor-kappaB via nuclear factor-inducing kinase/IkappaB kinase and mitogen-activated protein kinase pathways. *J Med Food.* **2009**, *12*, 351-358. https://doi.org.10.1089/jmf.2008.0006.

[412] Alshehri, A.S. Kaempferol attenuates diabetic nephropathy in streptozotocin-induced diabetic rats by a hypoglycaemic effect and concomitant activation of the Nrf-2/Ho-1/antioxidants axis. *Arch Physiol Biochem.* **2023**, *129*, 984-997. https://doi.org.10.1080/13813455.2021.1890129.

[413] Alshehri, A.S.; El-Kott, A.F.; El-Kenawy, A.E.; Zaki, M.S.A.; Morsy, K.; Ghanem, R.A.; Salem, E.T.; Ebealy, E.R.; Khalifa, H.S.; Altyar, A.E.; AlGwaiz, H.I.M.; Ibrahim, E.H.; Mahmoud, M.S.; Dallak, M.A.; Abd-Ella, E.M. The ameliorative effect of kaempferol against CdCl2- mediated renal damage entails activation of Nrf2 and inhibition of NF-kB. *Environ Sci Pollut Res Int.* **2022**, *29*, 57591-57602. https://doi.org.10.1007/s11356-022-19876-7.

[414] Wang, Z.; Sun, W.; Sun, X.; Wang, Y.; Zhou, M. Kaempferol ameliorates cisplatin induced nephrotoxicity by modulating oxidative stress, inflammation and apoptosis via ERK and NF-κB pathways. *AMB Express.* **2020**, *10*, 58. https://doi.org.10.1186/s13568-020-00993-w.

[415] Ali, A.S.; Almalki, A.S.; Alharthy, B.T. Effect of kaempferol on tacrolimus-induced nephrotoxicity and calcineurin B1 expression level in animal model. *J Exp Pharmacol.* **2020**, *12*, 397-407. https://doi.org.10.2147/JEP.S265359.

[416] Vijayaprakash, S.; Langeswaran, K.; Kumar, S.G.; Revathy, R.; Balasubramanian, M.P. Nephroprotective significance of kaempferol on mercuric chloride induced toxicity in Wistar albino rats. Biomed. *Aging Pathol.* **2013**, *3*, 119–124. https://doi.org.10.1016/j.biomag.2013.05.004.

[417] Langeswaran, K.; Selvaraj, J.; Ponnulakshmi, R.; Mathaiyan, M.; Vijayaprakash, S. Protective effect of kaempferol on biochemical and histopathological changes in mercuric chloride induced nephrotoxicity in experimental rats. J. Biol. *Active Prod. Nat.* **2018**, *8*, 125-136. https://doi.org.10.1080/22311866.2018.1451386.

[418] Hongyan, L.; Suling, W.; Weina, Z.; Yajie, Z.; Jie, R. Antihyperuricemic effect of liquiritigenin in potassium oxonate-induced hyperuricemic rats. *Biomed Pharmacother.* **2016**, *84*, 1930-1936. https://doi.org.10.1016/j.biopha.2016.11.009.

[419] Manjula, K.; Rajendran, K.; Eevera, T.; Kumaran, S. Effect of *Costus igneus* stem extract on calcium oxalate urolithiasis in albino rats. *Urol Res.* **2012**, *40*, 499-510. https://doi.org.10.1007/s00240-012-0462-6.

[420] Dharsan, R.; Vishnu Priya, V.; Ponnulakshmi, R.; Gayathri, R.; Madhan, K.; Shyamaladevi, B.; Selvaraj, J. Attenuation of diabetic nephropathy by a plant sterol lupeol: A biochemical analysis. *Drug Inv. Today*. **2019**, 12, 1402-1405.

[421] Nagaraj, M.; Sunitha, S.; Varalakshmi, P. Effect of lupeol, a pentacyclic triterpene, on the lipid peroxidation and antioxidant status in rat kidney after chronic cadmium exposure. *J Appl Toxicol.* **2000**, *20*, 413-417. https://doi.org.10.1002/1099-1263(200009/10)20:5<413::AID-JAT706>3.0.CO;2-Y.

[422] Sudhahar, V.; Veena, C.K.; Varalakshmi, P. Antiurolithic effect of lupeol and lupeol linoleate in experimental hyperoxaluria. *J Nat Prod.* **2008**, *71*, 1509-1512. https://doi.org.10.1021/np0703141.

[423] Vidya, L.; Varalakshmi, P. Control of urinary risk factors of stones by betulin and lupeol in experimental hyperoxaluria. *Fitoterapia.* **2000**, *71*, 535-543. https://doi.org.10.1016/s0367-326x(00)00192-1.

[424] Malini, M.M.; Lenin, M.; Varalakshmi, P. Protective effect of triterpenes on calcium oxalate crystal-induced peroxidative changes in experimental urolithiasis. *Pharmacol Res.* **2000**, *41*, 413-418. https://doi.org.10.1006/phrs.1999.0601.

[425] Liu, Y.; Shi, B.; Li, Y.; Zhang, H. Protective effect of luteolin against renal ischemia/reperfusion injury via modulation of pro-inflammatory cytokines, oxidative stress and apoptosis for possible benefit in kidney transplant. *Med Sci Monit.* **2017**, *23*, 5720-5727. https://doi.org.10.12659/msm.903253.

[426] Kalbolandi, S.M.; Gorji, A.V.; Babaahmadi-Rezaei, H.; Mansouri, E. Luteolin confers renoprotection against ischemia-reperfusion injury via involving Nrf2 pathway and regulating miR320. *Mol Biol Rep.* **2019**, *46*, 4039-4047. DOI: 10.1007/s11033-019-04853-0.

[427] Xin, S.B.; Yan, H.; Ma, J.; Sun, Q.; Shen, L. Protective effects of luteolin on lipopolysaccharide-induced acute renal injury in mice. *Med Sci Monit.* **2016**, *22*, 5173-5180. https://doi.org.10.12659/msm.898177.

[428] Oyagbemi, A.A.; Adejumobi, O.A.; Ajibade, T.O.; Asenuga, E.R.; Afolabi, J.M.; Ogunpolu, B.S.; Falayi, O.O.; Hassan, F.O.; Nabofa, E.W.; Olutayo Omobowale, T.; Ola-Davies, O.E.; Saba, A.B.; Adedapo, A.A.; Oguntibeju, O.O.; Yakubu, M.A. Luteolin attenuates glycerol-induced acute renal failure and cardiac complications through modulation of Kim-1/NF-κB/Nrf2 signaling pathways. *J Diet Suppl.* **2020**, *18*, 543-565. https://doi.org.10.1080/19390211.2020.1811442.

[429] Domitrović, R.; Cvijanović, O.; Pugel, E.P.; Zagorac, G.B. Mahmutefendić H, Škoda M. Luteolin ameliorates cisplatin-induced nephrotoxicity in mice through inhibition of platinum accumulation, inflammation and apoptosis in the kidney. *Toxicology.* **2013**, *310*, 115-123. https://doi.org.10.1016/j.tox.2013.05.015.

[430] Kang, K.P.; Park, S.K.; Kim, D.H; Sung, M.J.; Jung, Y.J.; Lee, A.S.; Lee, J.E.; Ramkumar, K.M.; Lee, S.; Park, M.H.; Roh, S.G.; Kim, W. Luteolin ameliorates cisplatin-induced acute kidney injury in mice by regulation of p53-dependent renal tubular apoptosis. *Nephrol Dial Transplant.* **2011**, *26*, 814-822. https://doi.org.10.1093/ndt/gfq528.

[431] Owumi, S.E.; Lewu, D.O.; Arunsi, U.O.; Oyelere, A.K. Luteolin attenuates doxorubicin-induced derangements of liver and kidney by reducing oxidative and inflammatory stress to suppress apoptosis. *Hum Exp Toxicol.* **2021**, *40*, 1656-1672. https://doi.org.10.1177/09603271211006171.

[432] Dar, A.A.; Fehaid, A.; Alkhatani, S.; Alarifi, S.; Alqahtani, W.S.; Albasher, G.; Almeer, R.; Alfarraj, S.; Moneim, A.A. The protective role of luteolin against the methotrexate-induced hepato-renal toxicity via its antioxidative, anti-inflammatory, and anti-apoptotic effects in rats. *Hum Exp Toxicol.* **2021**, *40*, 1194-1207. https://doi.org.10.1177/0960327121991905.

[433] Arslan, B.Y.; Arslan, F.; Erkalp, K.; Alagöl, A.; Sevdi, M.S.; Yıldız, G.; Küçük, S.H.; Altınay, S. Luteolin ameliorates colistin-induced nephrotoxicity in the rat models. Ren Fail. **2016**, *38*, 1735-1740. https://doi.org.10.1080/0886022X.2016.1229995.

[434] Tan, X.; Liu, B.; Lu, J.; Li, S.; Baiyun, R.; Lv, Y.; Lu, Q.; Zhang, Z. Dietary luteolin protects against $HgCl_2$-induced renal injury via activation of Nrf2-mediated signaling in rat. *J Inorg Biochem.* **2018**, *179*, 24-31. DOI: 10.1016/j.jinorgbio.2017.11.010.

[435] Albarakati, A.J.A.; Baty, R.S.; Aljoudi, A.M.; Habotta, O.A.; Elmahallawy, E.K.; Kassab, R.B.; Abdel Moneim, A.E. Luteolin protects against lead acetate-induced nephrotoxicity through antioxidant, anti-inflammatory, anti-apoptotic, and Nrf2/HO-1 signaling pathways. *Mol Biol Rep.* **2020**, *47*, 2591-2603. https://doi.org.10.1007/s11033-020-05346-1.

[436] Awoyomi, O.V.; Adeoye, Y.D.; Oyagbemi, A.A.; Ajibade, T.O.; Asenuga, E.R.; Gbadamosi, I.T.; Ogunpolu, B.S.; Falayi, O.O.; Hassan, F.O.; Omobowale, T.O.; Arojojoye, O.A.; Ola-Davies, O.E.;Saba, A.B.; Adedapo, A.A.; Oguntibeju, O.O.; Yakubu, M.A. Luteolin mitigates potassium dichromate-

induced nephrotoxicity, cardiotoxicity and genotoxicity through modulation of Kim-1/Nrf2 signaling pathways. *Environ Toxicol*. **2021**, *36*, 2146-2160. https://doi.org.10.1002/tox.23329.
[437] Liu, Y.S.; Yang, Q.; Li, S.; Luo, L.; Liu, H.Y.; Li, X.Y.; Gao, Z.N. Luteolin attenuates angiotensin II-induced renal damage in apolipoprotein E-deficient mice. *Mol Med Rep.* **2021**, *23*, 157. https://doi.org.10.3892/mmr.2020.11796.
[438] Lin, Y.; Liu, P.G.; Liang, W.Q.; Hu, Y.J.; Xu, P.; Zhou, J.; Pu, J.B.; Zhang, H.J. Luteolin-4'-O-glucoside and its aglycone, two major flavones of *Gnaphalium affine* D. Don, resist hyperuricemia and acute gouty arthritis activity in animal models. *Phytomedicine.* **2018**, *41*, 54-61. https://doi.org.10.1016/j.phymed.2018.02.002.
[439] Yuan, H.; Zhao, Y.; Li, S.; Qin, J.; Yu, X. Madecassoside ameliorates cisplatin-induced nephrotoxicity by inhibiting activation of the mitogen activated protein kinase pathway. *Environ Toxicol.* **2023**, *38*, 1473-1483.https://doi.org.10.1002/tox.23777.
[440] Cheng, Y.; Lu, Z.; Mao, T.; Song, Y.; Qu, Y.; Chen, X.; Chen, K.; Liu, K.; Zhang, C. Magnoflorine ameliorates chronic kidney disease in high-fat and high-fructose-fed mice by promoting Parkin/PINK1-dependent mitophagy to inhibit NLRP3/caspase-1-mediated pyroptosis. *J Agric Food Chem.* **2024**, *72*, 12775-12787. https://doi.org.10.1021/acs.jafc.3c09634.
[441] Yang, Z.J.; Wang, H.R.; Wang, Y.I.; Zhai, Z.H.; Wang, L.W.; Li, L.; Zhang, C.; Tang, L. Myricetin attenuated diabetes-associated kidney injuries and dysfunction *via* regulating nuclear factor (erythroid derived 2)-like 2 and nuclear factor-κB signaling. *Front Pharmacol.* **2019**, *10*, 647. https://doi.org.10.3389/fphar.2019.00647.
[442] Xu, W.L.; Zhou, P.P.; Yu, X.; Tian, T.; Bao, J.J.; Ni, C.R.; Zha, M.; Wu, X.; Yu, J.Y. Myricetin induces M2 macrophage polarization to alleviate renal tubulointerstitial fibrosis in diabetic nephropathy *via* PI3K/Akt pathway. *World J Diabetes.* **2024**, *15*, 105-125. https://doi.org.10.4239/wjd.v15.i1.105.
[443] Yang, X.; Zhang, P.; Jiang, J.; Almoallim, H.S.; Alharbi, S.A.; Li, Y. Myricetin attenuates ethylene glycol-induced nephrolithiasis in rats via mitigating oxidative stress and inflammatory markers. *Appl Biochem Biotechnol.* **2024**, *196*, 5419–5434. https://doi.org.10.1007/s12010-023-04831-0.
[444] Ozcan, F.; Ozmen, A.; Akkaya, B.; Aliciguzel, Y.; Aslan, M. Beneficial effect of myricetin on renal functions in streptozotocin-induced diabetes. *Clin Exp Med.* **2012**, *12*, 265-272. DOI: 10.1007/s10238-011-0167-0.
[445] Kandasamy, N.; Ashokkumar, N. Protective effect of bioflavonoid myricetin enhances carbohydrate metabolic enzymes and insulin signaling molecules in streptozotocin-cadmium induced diabetic nephrotoxic rats. *Toxicol Appl Pharmacol.* **2014**, *279*, 173-185. https://doi.org.10.1016/j.taap.2014.05.014.
[446] Kandasamy, N.; Ashokkumar, N. Renoprotective effect of myricetin restrains dyslipidemia and renal mesangial cell proliferation by the suppression of sterol regulatory element binding proteins in an experimental model of diabetic nephropathy. *Eur J Pharmacol.* **2014**, *743*, 53-62. https://doi.org.10.1016/j.ejphar.2014.09.014.
[447] El-Haleem, M.R.; Kattaia, A.A.; El-Baset, S.A.; Mostafa Hel, S. Alleviative effect of myricetin on ochratoxin A-induced oxidative stress in rat renal cortex: histological and biochemical study. *Histol Histopathol.* **2016**, *31*, 441-451. https://doi.org.10.14670/HH-11-689.
[448] Gao, X.; Xu, J.; Jiang, L.; Liu, W.; Hong, H.; Qian, Y.; Li, S.; Huang, W.; Zhao, H.; Yang, Z.; Liu, Q.; Wei., Z. Morin alleviates aflatoxin B1-induced liver and kidney injury by inhibiting heterophil extracellular traps release, oxidative stress and inflammatory responses in chicks. *Poult Sci.* **2021**, *100*, 101513. https://doi.org.10.1016/j.psj.2021.101513.

[449] Wei, Z.; He, X.; Kou, J.; Wang, J.; Chen, L.; Yao, M.; Zhou, E.; Fu, Y.; Guo, C.; Yang, Z. Renoprotective mechanisms of morin in cisplatin-induced kidney injury. *Int Immunopharmacol.* **2015**, *28*, 500-506. https://doi.org.10.1016/j.intimp.2015.07.009.

[450] Shehata, A.M.; Fares, N.H.; Amin, B.H.; Mahmoud, A.A.; Mahmoud, Y.I. Morin attenuates sepsis-induced acute kidney injury by regulating inflammatory responses, oxidative stress and tubular regeneration (morin and sepsis-induced acute kidney injury). *Environ Toxicol Pharmacol.* **2024**, *111*, 104543. https://doi.org.10.1016/j.etap.2024.104543.

[451] Çomaklı, S.; Kandemir, FM.; Küçükler, S.; Özdemir, S. Morin mitigates ifosfamide induced nephrotoxicity by regulation of NF-kappaB/p53 and Bcl-2 expression. *Biotech Histochem.* **2022**, *97*, 423-432. https://doi.org.10.1080/10520295.2021.2021449.

[452] Wang, C.P.; Wang, X.; Zhang, X.; Shi, Y.W.; Liu, L.; Kong, L.D. Morin improves urate excretion and kidney function through regulation of renal organic ion transporters in hyperuricemic mice. *J Pharm Pharm Sci.* **2010**, *13*, 411-427. https://doi.org.10.18433/j3q30h.

[453] Singh, M.P.; Sharma, C.; Kang, S.C. Morin hydrate attenuates adenine-induced renal fibrosis via targeting cathepsin D signaling. *Int Immunopharmacol.* **2021**, *90*, 107234. https://doi.org.10.1016/j.intimp.2020.107234.

[454] Kandemir, F.M.; Yıldırım, S.; Kucukler, S.; Caglayan, C.; Darendelioğlu, E.; Dortbudak, M.B. Protective effects of morin against acrylamide-induced hepatotoxicity and nephrotoxicity: A multi-biomarker approach. *Food Chem Toxicol.* **2020**, *138*, 111190. https://doi.org.10.1016/j.fct.2020.111190.

[455] Kuzu, M.; Yıldırım, S.; Kandemir, F.M.; Küçükler, S.; Çağlayan, C.; Türk, E.; Dörtbudak, M.B. Protective effect of morin on doxorubicin-induced hepatorenal toxicity in rats. *Chem Biol Interact.* 2019, *308*, 89-100. https://doi.org.10.1016/j.cbi.2019.05.017.

[456] Khan, T.H.; Ganaie, M.A.; Alharthy, K.M.; Madkhali, H.; Jan, B.L.; Sheikh, I.A. Naringenin prevents doxorubicin-induced toxicity in kidney tissues by regulating the oxidative and inflammatory insult in Wistar rats. *Arch Physiol Biochem.* **2020**, *126*, 300-307. https://doi.org.10.1080/13813455.2018.1529799.

[457] Kahramanoğullari, M.; Erişir, M.; Yaman, M.; Parlak Ak, T. Effects of naringenin on oxidative damage and apoptosis in liver and kidney in rats subjected to chronic mercury chloride. *Environ Toxicol.* **2024**, *39*, 2937-2947. DOI: 10.1002/tox.24164.

[458] Mu, L.; Hu, G.; Liu, J.; Chen, Y.; Cui, W.; Qiao, L. Protective Effects of naringenin in a rat model of sepsis-triggered acute kidney injury via activation of antioxidant enzymes and reduction in urinary angiotensinogen. *Med Sci Monit.* **2019**, *25*, 5986-5991. https://doi.org.10.12659/MSM.916400.

[459] Oyagbemi, A.A.; Omobowale, T.O.; Adejumobi, O.A.; Owolabi, B.S.; Ogunpolu, A.M.; Falayi, O.O.; Hassan, F.O.; Ogunmiluyi, I.O.; Asenuga, E.R.; Ola-Davies, O.E.; Soetan, K.O.; Saba, A.B.; Adedapo, A.A.; Nkadimeng, S.M.; McGaw, L.J.; Oguntibeju, O.O.; Yakubu, M.A. Antihypertensive power of naringenin is mediated via attenuation of mineralocorticoid receptor (MCR)/ angiotensin converting enzyme (ACE)/ kidney injury molecule (Kim-1) signaling pathway. *Eur J Pharmacol.* **2020**, *880*, 173142. https://doi.org.10.1016/j.ejphar.2020.173142.

[460] Abrego-Peredo, A.; Romero-Ramírez, H.; Espinosa, E.; López-Herrera, G.; García-García, F.; Flores-Muñoz, M.; Sandoval-Montes, C.; Rodríguez-Alba, J.C. Naringenin mitigates autoimmune features in lupus-prone mice by modulation of T-cell subsets and cytokines profile. *PLoS One.* **2020**, *15*, e0233138. https://doi.org.10.1371/journal.pone.0233138.

[461] Fouad, A.A.; Albuali, W.H.; Zahran, A.; Gomaa, W. Protective effect of naringenin against gentamicin-induced nephrotoxicity in rats. *Environ Toxicol Pharmacol.* **2014**, *38*, 420-429. https://doi.org.10.1016/j.etap.2014.07.015.

[462] Gnanasoundari, M.; Pari. L. Impact of naringenin on oxytetracycline-mediated oxidative damage in kidney of rats. *Ren Fail.* **2006**, *28*, 599-605. https://doi.org.10.1080/08860220600843805.

[463] Hermenean, A.; Ardelean, A.; Stan, M.; Herman, H.; Mihali, C.V.; Costache, M.; Dinischiotu, A. Protective effects of naringenin on carbon tetrachloride-induced acute nephrotoxicity in mouse kidney. *Chem Biol Interact.* **2013**, *205*, 138-147. https://doi.org.10.1016/j.cbi.2013.06.016.

[464] Karuppagounder, V.; Arumugam, S.; Thandavarayan, R.A.; Pitchaimani, V.; Sreedhar, R.; Afrin, R.; Harima, M.; Suzuki, H.; Suzuki, K.; Nakamura, M.; Ueno, K.; Watanabe, K. Naringenin ameliorates daunorubicin induced nephrotoxicity by mitigating AT1R, ERK1/2-NFκB p65 mediated inflammation. *Int Immunopharmacol.* **2015**, *28*, 154-159. https://doi.org.10.1016/j.intimp.2015.05.050.

[465] Wang, Z.; Wang, S.; Zhao, J.; Yu, C.; Hu, Y.; Tu, Y.; Yang, Z.; Zheng, J.; Wang, Y.; Gao. Y. Naringenin ameliorates renovascular hypertensive renal damage by normalizing the balance of renin-angiotensin system components in rats. *Int J Med Sci.* **2019**, *16*, 644-653. https://doi.org.10.7150/ijms.31075.

[466] Wang, J.; Yang, Z.; Lin, L.; Zhao, Z.; Liu, Z.; Liu, X. Protective effect of naringenin against lead-induced oxidative stress in rats. *Biol Trace Elem Res.* **2012**, *146*, 354-359. https://doi.org.10.1007/s12011-011-9268-6.

[467] Calis, Z.; Dasdelen, D.; Baltaci, A.K.; Mogulkoc, R. Naringenin prevents renal injury in experimental hyperuricemia through suppressing xanthine oxidase, inflammation, apoptotic pathway, DNA damage, and activating antioxidant system. *Metab Syndr Relat Disord.* **2023**, *21*, 275-281. https://doi.org.10.1089/met.2023.0012.

[468] Roy, S.; Ahmed, F.; Banerjee, S.; Saha, U. Naringenin ameliorates streptozotocin-induced diabetic rat renal impairment by downregulation of TGF-β1 and IL-1 via modulation of oxidative stress correlates with decreased apoptotic events. *Pharm Biol.* **2016**, *54*, 1616-1627. https://doi.org.10.3109/13880209.2015.1110599.

[469] Khaled, S.S.; Soliman, H.A.; Abdel-Gabbar, M.; Ahmed, N.A.; Attia, K.A.H.A.; Mahran, H.A.; El-Nahass, E.S.; Ahmed, O.M. The preventive effects of naringin and naringenin against paclitaxel-induced nephrotoxicity and cardiotoxicity in male Wistar rats. *Evid Based Complement Alternat Med.* **2022**, *30*, 8739815. https://doi.org.10.1155/2022/8739815.

[470] Tsai, S.J.; Huang, C.S.; Mong, M.C.; Kam, W.Y.; Huang, H.Y.; Yin, M.C. Anti-inflammatory and antifibrotic effects of naringenin in diabetic mice. *J Agric Food Chem.* **2012**, *60*, 514-521. https://doi.org.10.1021/jf203259h.

[471] Renugadevi, J.; Prabu, S.M. Naringenin protects against cadmium-induced oxidative renal dysfunction in rats. *Toxicology.* **2009**, *256*, 128-134. https://doi.org.10.1016/j.tox.2008.11.012.

[472] Badary, O.A.; Abdel-Maksoud, S.; Ahmed, W.A.; Owieda, G.H. Naringenin attenuates cisplatin nephrotoxicity in rats. *Life Sci.* **2005**, *76*, 2125-2135. https://doi.org.10.1016/j.lfs.2004.11.005.

[473] An, F.; Yang, G.; Tian, J.; Wang, S. Antioxidant effects of the orientin and vitexin in *Trollius chinensis* Bunge in D-galactose-aged mice. *Neural Regen Res.* **2012**, *7*, 2565-2575. https://doi.org.10.3969/j.issn.1673-5374.2012.33.001.

[474] Ijaz, M.U.; Aziz, S.; Faheem, M.; Abbas, K.; Nasir, S.; Naz, H.; Ali, A.; Rehman, T.; Imran, M. Orientin attenuates cisplatin-induced renal toxicity by reducing oxidative stress and inflammation. *Pak. Vet. J.* **2022**, *41*, 2074-7764.

[475] Murugaiyah, V.; Chan, K.L. Mechanisms of antihyperuricemic effect of *Phyllanthus niruri* and its lignin constituents. *J. Ethnopharmacol.* **2009**, *124*, 233–239.

[476] S. Promsan, K. Jaikumkao, A. Pongchaidecha, N. Chattipakorn, V. Chatsudthipong, P. Arjinajarn, W. Pompimon, A. Lungkaphin. Pinocembrin attenuates gentamicin-induced nephrotoxicity in rats. Can J Physiol Pharmacol. (2016) Aug;94(8):808-818. https://doi.org.10.1139/cjpp-2015-0468.

[477] Tonum, K.; Chabang, N.; Fongsupa, S.; Chantawarin, S.; Jiarpinitnun, C.; Tuchinda, P.; Soodvilai. S. Pinostrobin inhibits renal CFTR-mediated Cl- secretion and retards cyst growth in cell-derived cyst and polycystic kidney disease rats. *J Pharmacol Sci.* **2022**, *148*, 369-376. https://doi.org.10.1016/j.jphs.2022.02.003.

[478] Liu, J.X.; Yang, C.; Liu, Z.J.; Su, H.Y.; Zhang, W.H.; Pan, Q.; Liu, H.F. Protection of procyanidin B2 on mitochondrial dynamics in sepsis associated acute kidney injury via promoting Nrf2 nuclear translocation. *Aging (Albany NY).* **2020**, *12*, 15638-15655. https://doi.org.10.18632/aging.103726.

[479] Zhang, Z.; Li, B.Y.; Li, X.L.; Cheng, M.; Yu, F.; Lu, W.D.; Cai, Q.; Wang, J.F.; Zhou, R.H.; Gao, H.Q.; Shen, L. Proteomic analysis of kidney and protective effects of grape seed procyanidin B2 in db/db mice indicate MFG-E8 as a key molecule in the development of diabetic nephropathy. *Biochim Biophys Acta.* **2013**, *1832*, 805-816. https://doi.org.10.1016/j.bbadis.2013.02.022.

[480] He, J.; Sun, M.; Tian, S. Procyanidin B2 prevents lupus nephritis development in mice by inhibiting NLRP3 inflammasome activation. *Innate Immun.* **2018**, *24*, 307-315. https://doi.org.10.1177/1753425918780985.

[481] Deng, Z.J.; Zhao, J.F.; Huang, F.; Sun, G.L.; Gao, W.; Lu, L.; Xiao, Q. Protective effect of procyanidin B2 on acute liver injury induced by aflatoxin B 1 in rats. *Biomed Environ Sci.* **2020**, *33*, 238-247. https://doi.org.10.3967/bes2020.033.

[482] Zhou, Y.; Li, B.Y.; Li, X.L.; Wang, Y.J.; Zhang, Z.; Pei, F.; Wang, Q.Z.; Zhang, J.; Cai, Y.W.; Cheng, M.; Gao, H.Q. Restoration of mimecan expression by grape seed procyanidin B2 through regulation of nuclear factor-kappa B in mice with diabetic nephropathy. *Iran J Kidney Dis.* **2016**, *10*, 325-331.

[483] Salama, A.A.A.; Elgohary, R.; Fahmy, M.I. Protocatechuic acid ameliorates lipopolysaccharide-induced kidney damage in mice via downregulation of TLR-4-mediated IKBKB/NF-κB and MAPK/Erk signaling pathways. *J Appl Toxicol.* **2023**, *43*, 1119-1129. https://doi.org.10.1002/jat.4447.

[484] Kassab, R.B.; Theyab, A.; Al-Ghamdy, A.O.; Algahtani, M.; Mufti, A.H.; Alsharif, K.F.; Abdella, E.M.; Habotta, O.A.; Omran, M.M.; Lokman, M.S.; Bauomy, A.A.; Albrakati, A.; Baty, R.S.; Hassan, K.E.; Alshiekheid, M.A.; Abdel Moneim, A.E.; Elmasry, H.A. Protocatechuic acid abrogates oxidative insults, inflammation, and apoptosis in liver and kidney associated with monosodium glutamate intoxication in rats. *Environ Sci Pollut Res Int.* **2022**, *29*, 12208-12221. https://doi.org.10.1007/s11356-021-16578-4.

[485] Molehin, O.R.; Adeyanju, A.A.; Adefegha, S.A.; Oyeyemi, A.O.; Idowu, K.A. Protective mechanisms of protocatechuic acid against doxorubicin-induced nephrotoxicity in rat model. *J Basic Clin Physiol Pharmacol.* **2019**, *30*. https://doi.org.10.1515/jbcpp-2018-0191.

[486] Safaeian, L.; Emami, R.; Hajhashemi, V.; Haghighatian, Z. Antihypertensive and antioxidant effects of protocatechuic acid in deoxycorticosterone acetate-salt hypertensive rats. *Biomed Pharmacother.* **2018**, *100*, 147-155. https://doi.org.10.1016/j.biopha.2018.01.107.

[487] Owumi, S.E.; Ajijola, I.J.; Agbeti, O.M. Hepatorenal protective effects of protocatechuic acid in rats administered with anticancer drug methotrexate. *Hum Exp Toxicol.* **2019**, *38*, 1254-1265. https://doi.org.10.1177/0960327119871095.

[488] Lee, T.W.; Bae, E.; Kim, J.H.; Jung, M.H.; Park, D.J. Psoralen alleviates renal fibrosis by attenuating inflammasome-dependent NLRP3 activation and epithelial-mesenchymal transition in a mouse unilateral ureteral obstruction model. *Int J Mol Sci.* **2023**, *24*, 13171. https://doi.org.10.3390/ijms241713171.

[489] Dungca, N.T. Protective effect of the methanolic leaf extract of *Eclipta alba* (L.) Hassk. (Asteraceae) against gentamicin-induced nephrotoxicity in Sprague Dawley rats. *J Ethnopharmacol.* **2016**, *184*, 18-21. https://doi.org.10.1016/j.jep.2016.03.002.

[490] ameliorates kidney injury and fibrosis by modulating M1/M2 macrophage polarization. *Biochem Pharmacol.* **2018**, *154*, 203-212. https://doi.org.10.1016/j.bcp.2018.05.007.

[491] Liu, T.; Yang, Q.; Zhang, X.; Qin, R.; Shan, W.; Zhang, H.; Chen, X. Quercetin alleviates kidney fibrosis by reducing renal tubular epithelial cell senescence through the SIRT1/PINK1/mitophagy axis. *Life Sci.* **2020**, *257*, 118116. https://doi.org.10.1016/j.lfs.2020.118116.
[492] Tan, R.Z.; Wang, C.; Deng, C.; Zhong, X.; Yan, Y.; Luo Y.; Lan, H.Y.; He, T.; Wang, L. Quercetin protects against cisplatin-induced acute kidney injury by inhibiting Mincle/Syk/NF-κB signaling maintained macrophage inflammation. *Phytother Res.* **2020**, *34*, 139-152. https://doi.org.10.1002/ptr.6507.
[493] Yufang, W., Mingfang, L., Nan, H., Tingting, W. Quercetin-targeted AKT1 regulates the Raf/MEK/ERK signaling pathway to protect against doxorubicin-induced nephropathy in mice. *Tissue Cell.* **2023**, 85, 102229. https://doi.org.10.1016/j.tice.2023.102229.
[494] Liu, C.M.; Sun, Y.Z.; Sun, J.M.; Ma, J.Q.; Cheng. C. Protective role of quercetin against lead-induced inflammatory response in rat kidney through the ROS-mediated MAPKs and NF-κB pathway. *Biochim Biophys Acta*. **2012**, *1820*, 1693-1703. https://doi.org.10.1016/j.bbagen.2012.06.011.
[495] Wang, C.; Pan, Y.; Zhang, Q.Y.; Wang, F.M.; Kong, L.D. Quercetin and allopurinol ameliorate kidney injury in STZ-treated rats with regulation of renal NLRP3 inflammasome activation and lipid accumulation. *PLoS One*. **2012**, *7*, e38285. https://doi.org.10.1371/journal.pone.0038285.
[496] González-Esquivel, A.E.; Charles-Niño, C.L.; Pacheco-Moisés, F.P.; Ortiz, G.G.; Jaramillo-Juárez, F.; Rincón-Sánchez, A.R. Beneficial effects of quercetin on oxidative stress in liver and kidney induced by titanium dioxide (TiO2) nanoparticles in rats. *Toxicol Mech Methods.* **2015**, *25*, 166-175. https://doi.org.10.3109/15376516.2015.1006491.
[497] Tu, H.; Ma, D.; Luo, Y.; Tang, S.; Li, Y.; Chen, G.; Wang, L.; Hou, Z.; Shen, C.; Lu, H.; Zhuang, X.; Zhang, L. Quercetin alleviates chronic renal failure by targeting the PI3k/Akt pathway. *Bioengineered*. **2021**, *12*, 6538-6558. https://doi.org.10.1080/21655979.2021.1973877.
[498] Sangai, N.P.; Verma, R.J.; Trivedi, M.H. Testing the efficacy of quercetin in mitigating bisphenol A toxicity in liver and kidney of mice. *Toxicol Ind Health.* **2014**, *30*, 581-597. https://doi.org.10.1177/0748233712457438.
[499] Nabavi, S.M.; Nabavi, S.F.; Habtemariam, S.; Moghaddam, A.H.; Latifi, A.M. Ameliorative effects of quercetin on sodium fluoride-induced oxidative stress in rat's kidney. *Ren Fail.* **2012**, *34*, 901-906. https://doi.org.10.3109/0886022X.2012.687347.
[500] Hou, Y.; Zeng, Y.; Li, S.; Qi, L.; Xu, W.; Wang, H.; Zhao, X.; Sun, C. Effect of quercetin against dichlorvos induced nephrotoxicity in rats. *Exp Toxicol Pathol.* **2014**, *66*, 211-218. https://doi.org.10.1016/j.etp.2014.01.007.
[501] Santos ,M.; Poletti, P.T.; Favero, G.; Stacchiotti, A.; Bonomini, F.; Montanari, C.C.; Bona, S.R.; Marroni, N.P.; Rezzani, R.; Veronese, F.V. Protective effects of quercetin treatment in a pristane-induced mouse model of lupus nephritis. *Autoimmunity.* **2018**, *51*, 69-80. https://doi.org.10.1080/08916934.2018.1442828.
[502] Bo, L.; Liu, Y.; Jia, S.; Liu, Y.; Zhang, M.; Li, S.; Zhao, X.; Sun, C. Metabonomics analysis of quercetin against the nephrotoxicity of acrylamide in rats. *Food Funct.* **2018**, *9*, 5965-5974. https://doi.org.10.1039/c8fo00902c.
[503] Gonçalves, G.F.B.; Silva, M.E.M.; Sampaio, F.J.B.; Pereira-Sampaio, M.A.; Souza, D.B. Quercetin as a nephroprotector after warm ischemia: histomorphometric evaluation in a rodent model. *Int Braz J Urol*. **2021**, *47*, 796-802. https://doi.org.10.1590/S1677-5538.IBJU.2020.0358.
[504] El-Far, A.H.; Lebda, M.A.; Noreldin, A.E.; Atta, M.S.; Elewa, Y.H.A.; Elfeky, M.; Mousa, S.A. Quercetin attenuates pancreatic and renal D-galactose-induced aging-related oxidative alterations in rats. *Int J Mol Sci.* **2020**, *21*, 4348. https://doi.org.10.3390/ijms21124348.
[505] Liu, Y.; Dai, E.; Yang, J. Quercetin suppresses glomerulosclerosis and TGF-β signaling in a rat model. *Mol Med Rep*. **2019**, *19*, 4589-4596. https://doi.org.10.3892/mmr.2019.10118.

[506] Abdelhalim, M.A.K.; Qaid, H.A.; Al-Mohy, Y.; Al-Ayed, M.S. Effects of quercetin and arginine on the nephrotoxicity and lipid peroxidation induced by gold nanoparticles in vivo. *Int J Nanomedicine.* **2018**, *13*, 7765-7770. https://doi.org.10.2147/IJN.S183281.

[507] Özyurt, H.; Çevik, Ö.; Özgen, Z.; Özden, A.S.; Çadırcı, S.; Elmas, M.A.; Ercan, F.; Gören, M.Z.; Şener, G. Quercetin protects radiation-induced DNA damage and apoptosis in kidney and bladder tissues of rats. *Free Radic Res.* **2014**, *48*, 1247-1255. https://doi.org.10.3109/10715762.2014.945925.

[508] Zhang, Y.; Gao, Z.; Liu, J.; Xu, Z. Protective effects of baicalin and quercetin on an iron-overloaded mouse: comparison of liver, kidney and heart tissues. *Nat Prod Res.* **2011**, *25*, 1150-1160. https://doi.org.10.1080/14786419.2010.495070.

[509] Soliman, M.M.; Gaber, A.; Alsanie, W.F.; Mohamed, W.A.; Metwally, M.M.M.; Abdelhadi, A.A.; Elbadawy, M.; Shukry, M. Gibberellic acid-induced hepatorenal dysfunction and oxidative stress: Mitigation by quercetin through modulation of antioxidant, anti-inflammatory, and antiapoptotic activities. *J Food Biochem.* **2022**, *46*, e14069. https://doi.org.10.1111/jfbc.14069.

[510] Guzel, A.; Yunusoglu, S.; Calapoglu, M.; Candan, I.A.; Onaran, I.; Oncu, M.; Ergun, O.; Oksay, T. Protective effects of quercetin on oxidative stress-induced tubular epithelial damage in the experimental rat hyperoxaluria model. *Medicina (Kaunas).* **2021**, *57*, 566. https://doi.org.10.3390/medicina57060566.

[511] Hou, Y.; Ding, T.; Guan, Z.; Wang, J.; Yao, R.; Yu, Z.; Zhao. X. Untargeted metabolomics reveals the preventive effect of quercetin on nephrotoxicity induced by four organophosphorus pesticide mixtures. *Food Chem Toxicol.* **2023**, *175*, 113747. https://doi.org.10.1016/j.fct.2023.113747.

[512] Baran, M.; Yay, A.; Onder, G.O.; Canturk Tan, F.; Yalcin, B.; Balcioglu, E.; Yıldız, O.G. Hepatotoxicity and renal toxicity induced by radiation and the protective effect of quercetin in male albino rats. *Int J Radiat Biol.* **2022**, *98*, 1473-1483. https://doi.org.10.1080/09553002.2022.2033339.

[513] Shin, Y.J.; Kim, J.J.; Kim, Y.J.; Kim, W.H.; Park, E.Y.; Kim, I.Y.; Shin, H.S.; Kim, K.S.; Lee, E.K.; Chung, K.H.; Lee, B.M.; Kim, H.S. Protective effects of quercetin against $HgCl_2$-induced nephrotoxicity in Sprague-Dawley rats. *J Med Food.* **2015**, *18*, 524-534. https://doi.org.10.1089/jmf.2014.3242.

[514] Rathi, V.; Tiwari, I.; Kulshreshtha, R.; Sagi, S.S.K. Hypobaric hypoxia induced renal injury in rats: Prophylactic amelioration by quercetin supplementation. *PLoS One.* **2023**, *24;18*, e0279304. https://doi.org.10.1371/journal.pone.0279304.

[515] Owumi, S.E.; Danso, O.F.; Effiong, M.E. Dietary quercetin abrogates hepatorenal oxidative damage associated with dichloromethane exposure in rats. *Acta Biochim Pol.* **2019**, *66*, 201-206. https://doi.org.10.18388/abp.2018_2771.

[516] Al-Rasheed, N.M.; Fadda, L.M.; Attia, H.A.; Ali, H.M.; Al-Rasheed, N.M. Quercetin inhibits sodium nitrite-induced inflammation and apoptosis in different rats organs by suppressing Bax, HIF1-α, TGF-β, Smad-2, and AKT pathways. *J Biochem Mol Toxicol.* **2017**, *31*. https://doi.org.10.1002/jbt.21883.

[517] Alam, M.M.; Meerza, D.; Naseem, I. Protective effect of quercetin on hyperglycemia, oxidative stress and DNA damage in alloxan induced type 2 diabetic mice. *Life Sci.* **2014**, *109*, 8-14. https://doi.org.10.1016/j.lfs.2014.06.005.

[518] Guan,T.; Xin, Y.; Zheng, K.; Wang, R.; Zhang, X.; Jia, S.; Li, S.; Cao, C.; Zhao, X. Metabolomics analysis of the effects of quercetin on renal toxicity induced by cadmium exposure in rats. *Biometals.* **2021**, *34*, 33-48. https://doi.org.10.1007/s10534-020-00260-2.

[519] Chang, X.Y.; Cui, L.; Wang, X.Z.; Zhang, L.; Zhu, D.; Zhou, X.R.; Hao, L.R. Quercetin attenuates vascular calcification through suppressed oxidative stress in adenine-induced chronic renal failure rats. *Biomed Res Int.* **2017**, 5716204. https://doi.org.10.1155/2017/5716204.

[520] Kahraman, A.; Erkasap, N.; Serteser, M.; Köken, T. Protective effect of quercetin on renal ischemia/reperfusion injury in rats. *J Nephrol.* **2003**, *16*, 219-224.

[521] Chen, J.; Zhang, H.; Yang, Y.; Chen, B. Quercetin regulates vascular endothelium function in chronic renal failure via modulation of Eph/Cav-1 signaling. *Drug Dev Res.* **2022**, *83*, 1167-1175. https://doi.org.10.1002/ddr.21940.

[522] Morales, A.I.; Vicente-Sánchez, C.; Jerkic, M.; Santiago, J.M.; Sánchez-González, P.D.; Pérez-Barriocanal, F.; López-Novoa, J.M. Effect of quercetin on metallothionein, nitric oxide synthases and cyclooxygenase-2 expression on experimental chronic cadmium nephrotoxicity in rats. *Toxicol Appl Pharmacol.* **2006**, *210*, 128-135. https://doi.org.10.1016/j.taap.2005.09.006.

[523] Hu, Q.H.; Zhang, X.; Wang, X.; Jiao, R.Q.; Kong, L.D. Quercetin regulates organic ion transporter and uromodulin expression and improves renal function in hyperuricemic mice. *Eur J Nutr.* **2012**, 51, 593-606. https://doi.org.10.1007/s00394-011-0243-y.

[524] Abdel-Raheem, I.T.; Abdel-Ghany, A.A.; Mohamed, G.A. Protective effect of quercetin against gentamicin-induced nephrotoxicity in rats. *Biol Pharm Bull.* **2009**, *32*, 61-67. https://doi.org.10.1248/bpb.32.61.

[525] Ebokaiwe, A.P.; Obasi, D.O.; Njoku, R.C.C.; Osawe, S.; Olusanya, O.; Kalu W.O. Cyclophosphamide instigated hepatic-renal oxidative/inflammatory stress aggravates immunosuppressive indoleamine 2,3-dioxygenase in male rats: Abatement by quercetin. *Toxicology.* **2021**, *464*, 153027. https://doi.org.0.1016/j.tox.2021.153027.

[526] Morsi, A.A.; Fouad, H.; Alasmari, W.A.; Faruk, E.M. The biomechanistic aspects of renal cortical injury induced by diesel exhaust particles in rats and the renoprotective contribution of quercetin pretreatment: Histological and biochemical study. *Environ Toxicol.* **2022**, *37*, 310-321. https://doi.org.10.1002/tox.23399.

[527] Behling, E.B.; Sendão, M.C.; Francescato, H.D.; Antunes, L.M.; Costa, R.S.; Bianchi, M.L.P. Comparative study of multiple dosage of quercetin against cisplatin-induced nephrotoxicity and oxidative stress in rat kidneys. *Pharmacol Rep.* **2006**, *58*, 526-532.

[528] Erboga, M.; Aktas, C.; Erboga, Z.F.; Donmez, Y.B.; Gurel, A. Quercetin ameliorates methotrexate-induced renal damage, apoptosis and oxidative stress in rats. *Ren Fail.* **2015**, *37*, 1492-1497. https://doi.org.10.3109/0886022X.2015.1074521.

[529] Abdelrahman, R.E.; Khalaf, A.A.A.; Elhady, M.A.; Ibrahim, M.A.; Hassanen, E.I.; Noshy, P.A. Antioxidant and antiapoptotic effects of quercetin against ochratoxin A-induced nephrotoxicity in broiler chickens. *Environ Toxicol Pharmacol.* **2022**, *96*, 103982. https://doi.org.10.1016/j.etap.2022.103982.

[530] Uthra, C.; Shrivastava, S.; Jaswal, A.; Sinha, N.; Reshi, M.S.; Shukla, S. Therapeutic potential of quercetin against acrylamide induced toxicity in rats. *Biomed Pharmacother.* **2017**, *86*, 705-714. https://doi.org.10.1016/j.biopha.2016.12.065.

[531] Shu, B.; Feng, Y.; Gui, Y.; Lu, Q.; Wei, W.; Xue, X.; Sun, X.; He, W.; Yang, J.; Dai, C. Blockade of CD38 diminishes lipopolysaccharide-induced macrophage classical activation and acute kidney injury involving NF-κB signaling suppression. *Cell Signal.* **2018**, *42*, 249-258. https://doi.org.10.1016/j.cellsig.2017.10.014.

[532] Eybl, V.; Kotyzová, D.; Cerná, P.; Koutensky. J. Effect of melatonin, curcumin, quercetin, and resveratrol on acute ferric nitrilotriacetate (Fe-NTA)-induced renal oxidative damage in rats. *Hum Exp Toxicol.* **2008**, *27*, 347-353. https://doi.org.10.1177/0960327108094508.

[533] Sanchez-Gonzalez, P.D.; Lopez-Hernandez, F.J.; Perez-Barriocanal, F.; Morales, A.I.; Lopez-Novoa, J.M. Quercetin reduces cisplatin nephrotoxicity in rats without compromising its anti-tumour activity. *Nephrol Dial Transplant.* **2011**, *26*, 3484-3495. https://doi.org.10.1093/ndt/gfr195.

[534] Alshammari, G.M.; Al-Qahtani, W.H.; AlFaris, N.A.; Albekairi, N.A.; Alqahtani, S.; Eid, R. Yagoub, A.E.A.; Al-Harbi, L.N.; Yahya. M.A. Quercetin alleviates cadmium chloride-induced renal damage in rats by suppressing endoplasmic reticulum stress through SIRT1-dependent deacetylation of Xbp-1s and eIF2α. *Biomed Pharmacother.* **2021**, *141*, 111862. https://doi.org.10.1016/j.biopha.2021.111862.

[535] Chander, V.; Singh, D.; Chopra, K. Reversal of experimental myoglobinuric acute renal failure in rats by quercetin, a bioflavonoid. *Pharmacology.* **2005**, *73*, 49-56. https://doi.org.10.1159/000081074.

[536] Carlsen, I.; Frøkiær, J.; Nørregaard, R. Quercetin attenuates cyclooxygenase-2 expression in response to acute ureteral obstruction. *Am J Physiol Renal Physiol.* **2015**, *308*, F1297-305. https://doi.org.10.1152/ajprenal.00514.2014.
[537] Peng, C.C.; Hsieh, C.L.; Ker, Y.B.; Wang, H.Y.; Chen, K.C.; Peng, R.Y. Selected nutraceutic screening by therapeutic effects on doxorubicin-induced chronic kidney disease. *Mol Nutr Food Res*. **2012**, *56*, 1541-1558. https://doi.org.10.1002/mnfr.201200178.
[538] Lai, P.B.; Zhang, L.; Yang, L.Y. Quercetin ameliorates diabetic nephropathy by reducing the expressions of transforming growth factor-β1 and connective tissue growth factor in streptozotocin-induced diabetic rats. *Ren Fail.* **2011**, *34*, 83-87. https://doi.org.10.3109/0886022X.2011.623564.
[539] Faddah, L.M.; Abdel Baky, N.A.; Al-Rasheed, N.M.; Al-Rasheed, N.M.; Fatani, A.J.; Atteya, M. Role of quercetin and arginine in ameliorating nano zinc oxide-induced nephrotoxicity in rats. *BMC Complement Altern Med.* **2012**, *12*, 60. https://doi.org.10.1186/1472-6882-12-60.
[540] Renugadevi, J.; Prabu, S.M. Quercetin protects against oxidative stress-related renal dysfunction by cadmium in rats. *Exp Toxicol Pathol.* **2010**, *62*, 471-481. https://doi.org.10.1016/j.etp.2009.06.006.
[541] Anjaneyulu, M.; Chopra, K. Quercetin, an anti-oxidant bioflavonoid, attenuates diabetic nephropathy in rats. *Clin Exp Pharmacol Physiol.* **2004**, *31*, 244-248. https://doi.org.0.1111/j.1440-1681.2004.03982.x.
[542] Ciftci, O.; Ozdemir, I.; Vardi, N.; Beytur, A.; Oguz, F. Ameliorating effects of quercetin and chrysin on 2,3,7,8-tetrachlorodibenzo- p-dioxin-induced nephrotoxicity in rats. *Toxicol Ind Health.* **2012**, *28*, 947-954. https://doi.org.10.1177/0748233711430978.
[543] Crown, O.O.; Ogundele, O.O.; Akinmoladun, A.C.; Famusiwa, C.D.; Josiah, S.S.; Olaleye, M.T.; Akindahunsi, A.A. Effects of catechin, quercetin and taxifolin on redox parameters and metabolites linked with renal health in rotenone-toxified rats. *Niger J Physiol Sci.* **2019**, *34*, 1-10.
[544] Hu, Q.H.; Wang, C.; Li, J.M.; Zhang, D.M.; Kong, L.D. Allopurinol, rutin, and quercetin attenuate hyperuricemia and renal dysfunction in rats induced by fructose intake: renal organic ion transporter involvement. Am *J Physiol Renal Physiol.* **2009**, *297*, F1080-91. https://doi.org.10.1152/ajprenal.90767.2008.
[545] Al-Asmari, A.K.; Khan, H.A.; Manthiri, R.A.; Al-Khlaiwi, A.A.; Al-Asmari, B.A.; Ibrahim, K.E. Protective effects of a natural herbal compound quercetin against snake venom-induced hepatic and renal toxicities in rats. *Food Chem Toxicol.* **2018**, *118*, 105-110. https://doi.org.10.1016/j.fct.2018.05.016.
[546] Zal, F.; Mostafavi-Pour, Z.; Vessal, M. Comparison of the effects of vitamin E and/or quercetin in attenuating chronic cyclosporine A-induced nephrotoxicity in male rats. *Clin Exp Pharmacol Physiol.* **2007**, *34*, 720-724. https://doi.org.10.1111/j.1440-1681.2007.04623.x.
[547] Heeba, G.H.; Mahmoud, M.E. Dual effects of quercetin in doxorubicin-induced nephrotoxicity in rats and its modulation of the cytotoxic activity of doxorubicin on human carcinoma cells. *Environ Toxicol.* **2016**, *31*, 624-636. https://doi.org.10.1002/tox.22075.
[548] Aoi, W.; Niisato, N.; Miyazaki, H.; Marunaka, Y. Flavonoid-induced reduction of ENaC expression in the kidney of Dahl salt-sensitive hypertensive rat. *Biochem Biophys Res Commun.* **2004**, *315*, 892-896.https://doi.org.10.1016/j.bbrc.2004.01.150.
[549] Abdelrahman, F.A.F.; El-Sayed, S.A.; Abuel-Atta, A.A.; Ghonimi, W.A.M. Nephrotoxicity induced by different diameters of sphere gold nanoparticles with special emphasis on the nephroprotective role of quercetin. *Open Vet J.* **2023**, *13*, 723-731. https://doi.org.10.5455/OVJ.2023.v13.i6.7.
[550] Khalil, S.R.; Mohammed, A.T.; Abd El-Fattah, A.H.; Zaglool, A.W. Intermediate filament protein expression pattern and inflammatory response changes in kidneys of rats receiving doxorubicin chemotherapy and quercetin. *Toxicol Lett.* **2018**, *288*, 89-98. https://doi.org.10.1016/j.toxlet.2018.02.024.
[551] Satyanarayana, P.S.; Singh, D.; Chopra, K. Quercetin, a bioflavonoid, protects against oxidative stress-related renal dysfunction by cyclosporine in rats. *Methods Find Exp Clin Pharmacol.* **2001**, *23*, 175-181. https://doi.org.10.1358/mf.2001.23.4.634641.

[552] Becerra-Torres, S.L.; Rodríguez-Vázquez, M.L.; Medina-Ramírez, I.E.; Jaramillo-Juárez, F. The flavonoid quercetin protects and prevents against potassium dichromate-induced systemic peroxidation of lipids and diminution in renal clearance of para-aminohippuric acid and inulin in the rat. *Drug Chem Toxicol.* **2009**, *32*, 88-91. https://doi.org.0.1080/01480540802449951.
[553] Gelen, V.; Şengül, E.; Gedikli, S.; Gür, C.; Özkanlar, S. Therapeutic effect of quercetin on renal function and tissue damage in the obesity induced rats. *Biomed Pharmacother*. **2017**, *89*, 524-528. https://doi.org.10.1016/j.biopha.2017.02.057.
[554] Gomes, I.B.; Porto, M.L.; Santos, M.C.; Campagnaro, B.P.; Pereira, T.M.; Meyrelles, S.S.; Vasquez, E.C. Renoprotective, anti-oxidative and anti-apoptotic effects of oral low-dose quercetin in the C57BL/6J model of diabetic nephropathy. *Lipids Health Dis.* **2014**, *13*, 184. https://doi.org.0.1186/1476-511X-13-184.
[555] Francescato, H.D.; Coimbra, T.M.; Costa, R.S.; Bianchi, M.L.P. Protective effect of quercetin on the evolution of cisplatin-induced acute tubular necrosis. *Kidney Blood Press Res.* **2004**, *27*, 148-58. https://doi.org.10.1159/000078309.
[556] D. Singh, V. Chander, K. Chopra. Quercetin, a bioflavonoid, attenuates ferric nitrilotriacetate-induced oxidative renal injury in rats. Drug Chem Toxicol. (2004) May;27(2):145-156. https://doi.org.10.1081/dct-120030729.
[557] Nikolić, J.; Cvetković, T.; Sokolović, D. Role of quercetin on hepatic urea production in acute renal failure. *Ren Fail.* **2003**, 25, 149-155. https://doi.org.10.1081/jdi-120018716.
[558] Yuksel, Y.; Yuksel, R.; Yagmurca, M.; Haltas, H.; Erdamar, H.; Toktas, M.; Ozcan, O. Effects of quercetin on methotrexate-induced nephrotoxicity in rats. *Hum Exp Toxicol.* **2017**, *36*, 51-61. https://doi.org.10.1177/0960327116637414.
[559] Inal, M.; Altinişik, M.; Bilgin, M.D. The effect of quercetin on renal ischemia and reperfusion injury in the rat. *Cell Biochem Funct.* **2002**, *20*, 291-296. https://doi.org.10.1002/cbf.953.
[560] Jia, S.; Guan, T.; Zhang, X.; Liu, Y.; Liu, Y.; Zhao, X. Serum metabonomics analysis of quercetin against the toxicity induced by cadmium in rats. *J. Biochem. Mol. Toxicol.* **2020**, *34*, e22448. https://doi.org.10.1002/jbt.22448
[561] Alhusaini, A.; Fadda, L.M.; Ali, H.M.; Hasan, I.H.; Ali, R.A.; Zakaria, E.A. Mitigation of acetamiprid - induced renotoxicity by natural antioxidants via the regulation of ICAM, NF-kB and TLR 4 pathways. *Pharmacol Rep.* **2019**, *71*, 1088-1094. https://doi.org.10.1016/j.pharep.2019.06.008.
[562] Li, W.; Li, H.; Zhang, M.; Wang, M.; Zhong, Y.; Wu, H.; Yang, Y.; Morel, L.; Wei, Q. Quercitrin ameliorates the development of systemic lupus erythematosus-like disease in a chronic graft-versus-host murine model. *Am J Physiol Renal Physiol.* **2016**, *311*, F217-26. https://doi.org.10.1152/ajprenal.00249.2015.
[563] Babujanarthanam, R.; Kavitha, P.; Mahadeva Rao, U.S.; Pandian, M.R. Quercitrin a bioflavonoid improves the antioxidant status in streptozotocin: induced diabetic rat tissues. *Mol Cell Biochem.* **2011**, *358*, 121-129. https://doi.org.10.1007/s11010-011-0927-x.
[564] Chen, J.; Zhang, Q.; Guo, J.; Gu, D.; Liu, J.; Luo, P.; Bai, Y.; Chen, J.; Zhang, X.; Nie, S.; Chen, C.; Feng, Y.; Wang, J. Single-cell transcriptomics reveals the ameliorative effect of rosmarinic acid on diabetic nephropathy-induced kidney injury by modulating oxidative stress and inflammation. *Acta Pharm Sin B.* **2024**, *14*, 1661-1676. https://doi.org.10.1016/j.apsb.2024.01.003.
[565] Xiang, Y.; Ji, M.; Wu, L.; Lv, L.; Liang, Q.; Deng, R.; Deng, Z.; Liu, X.; Ren, L.; Feng, X.; He, J. Rosmarinic acid prevents cisplatin-induced liver and kidney injury by inhibiting inflammatory responses and enhancing total antioxidant capacity, thereby Activating the Nrf2 signaling pathway. *Molecules.* **2022**, *27*, 7815. https://doi.org.10.3390/molecules27227815.

[566] Akhter, J.; Khan, J.; Baghel, M.; Beg, M.M.A.; Goswami, P.; Afjal, M.A.; Ahmad, S.; Habib, H.; Najmi, A.K.; Raisuddin, S. NLRP3 inflammasome in rosmarinic acid-afforded attenuation of acute kidney injury in mice. *Sci Rep.* **2022**, *12*, 1313. https://doi.org.10.1038/s41598-022-04785-z.

[567] Abduh, M.S.; Alruhaimi, R.S.; Alqhtani, H.A.; Hussein, O.E.; Abukhalil, M.H.; Kamel, E.M.; Mahmoud. A.M. Rosmarinic acid mitigates chlorpyrifos-induced oxidative stress, inflammation, and kidney injury in rats by modulating SIRT1 and Nrf2/HO-1 signaling. Life Sci. (2023) Jan;313:121281. https://doi.org.10.1016/j.lfs.2022.121281.

[568] Domitrović, R.; Potočnjak, I.; Crnčević-Orlić, Z.; Škoda, M. Nephroprotective activities of rosmarinic acid against cisplatin-induced kidney injury in mice. *Food Chem Toxicol.* **2014**, *66*, 321-328. https://doi.org.10.1016/j.fct.2014.02.002.

[569] Mushtaq, N.; Schmatz, R.; Ahmed, M.; Pereira, L.B.; Costa, P.; Reichert, K.P.; Dalenogare, D.; Pelinson, L.P.; Vieira, J.M.; Stefanello, N.; Oliveira, L.S.; Mulinacci, N.; Bellumori, M.; Morsch, V.M.; Schetinger, M.R. Protective effect of rosmarinic acid against oxidative stress biomarkers in liver and kidney of streptozotocin-induced diabetic rats. *J Physiol Biochem.* **2015**, *71*, 743-751. https://doi.org.10.1007/s13105-015-0438-4.

[570] Jafaripour, L.; Naserzadeh, R.; Alizamani, E.; Javad Mashhadi, S.M.; Moghadam, E.R.; Nouryazdan, N.; Ahmadvand, H. Effects of rosmarinic acid on methotrexate-induced nephrotoxicity and hepatotoxicity in Wistar rats. *Indian J Nephrol.* **2021**, *31*, 218-224. https://doi.org.10.4103/ijn.IJN_14_20.

[571] Khalaf, A.A.; Hassanen, E.I.; Ibrahim, M.A.; Tohamy, A.F.; Aboseada, M.A.; Hassan, H.M.; Zaki, A.R. Rosmarinic acid attenuates chromium-induced hepatic and renal oxidative damage and DNA damage in rats. *J Biochem Mol Toxicol.* **2020**, *34*, e22579. https://doi.org.10.1002/jbt.22579.

[572] Samsu, N.; Soeharto, S.; Rifai, M.; Rudijanto, A. Rosmarinic acid monotherapy is better than the combination of rosmarinic acid and telmisartan in preventing podocyte detachment and inhibiting the progression of diabetic nephropathy in rats. *Biologics*. **2019**, *13*, 179-190. https://doi.org.10.2147/BTT.S214820.

[573] Tavafi, M.; Ahmadvand, H. Effect of rosmarinic acid on inhibition of gentamicin induced nephrotoxicity in rats. *Tissue Cell.* **2011**, *43*, 392-397. https://doi.org.10.1016/j.tice.2011.09.001.

[574] Macarini, A.F.; Mariano, L.N.B.; Zanovello, M.; Silva, R.C.V.; Corrêa, R.; Souza, P. Protective role of rosmarinic acid in experimental urolithiasis: understanding its impact on renal parameters. *Pharmaceuticals.* **2024**, *17*, 702. https://doi.org.10.3390/ph17060702.

[575] Parameshappa, B.; Ali Basha, M.S.; Sen, S.; Chakraborty, R.; Kumar, G.V.; Sagar, G.V.; Sowmya, L.; Raju, K.K.; Sesh Kumar, P.K.; Lakshmi, A.V. Acetaminophen-induced nephrotoxicity in rats: protective role of *Cardiospermum halicacabum*. *Pharm Biol.* **2012**, *50*, 247-253. https://doi.org.10.3109/13880209.2011.596843.

[576] Kandemir, F.M.; Ileriturk, M.; Gur, C. Rutin protects rat liver and kidney from sodium valproate-induce damage by attenuating oxidative stress, ER stress, inflammation, apoptosis and autophagy. *Mol Biol Rep.* **2022**, *49*, 6063-6074. https://doi.org.10.1007/s11033-022-07395-0.

[577] Rakshit, S.; Shukla, P.; Verma, A.; Kumar Nirala, S.; Bhadauria, M. Protective role of rutin against combined exposure to lipopolysaccharide and D-galactosamine-induced dysfunctions in liver, kidney, and brain: Hematological, biochemical, and histological evidences. *J Food Biochem.* **2021**, *45*, e13605. https://doi.org.10.1111/jfbc.13605.

[578] Khajevand-Khazaei, M.R.; Mohseni-Moghaddam, P.; Hosseini, M.; Gholami, L.; Baluchnejadmojarad, T.; Roghani, M. Rutin, a quercetin glycoside, alleviates acute endotoxemic kidney injury in C57BL/6 mice via suppression of inflammation and up-regulation of antioxidants and SIRT1. *Eur J Pharmacol.* **2018**, *833*, 307-313. https://doi.org.10.1016/j.ejphar.2018.06.019.

[579] Qu, S.; Dai, C.; Lang, F.; Hu, L.; Tang, Q.; Wang, H.; Zhang, Y.; Hao, Z. Rutin attenuates vancomycin-induced nephrotoxicity by ameliorating oxidative stress, apoptosis, and inflammation in rats. *Antimicrob Agents Chemother.* **2018**, *63*, e01545-18. https://doi.org.10.1128/AAC.01545-18.
[580] Gur, C.; Kandemir, F.M. Molecular and biochemical investigation of the protective effects of rutin against liver and kidney toxicity caused by malathion administration in a rat model. *Environ Toxicol.* **2023**, *38*, 555-565. https://doi.org.10.1002/tox.23700.
[581] Ma, J.Q.; Liu, C.M.; Yang, W. Protective effect of rutin against carbon tetrachloride-induced oxidative stress, inflammation and apoptosis in mouse kidney associated with the ceramide, MAPKs, p53 and calpain activities. *Chem Biol Interact.* **2018**, *286*, 26-33. https://doi.org.10.1016/j.cbi.2018.03.003.
[582] Ma, X.; Ren, X.; Zhang, X.; Wang, G.; Liu, H.; Wang, L. Rutin ameliorate PFOA induced renal damage by reducing oxidative stress and improving lipid metabolism. *J Nutr Biochem.* **2023**, *123*, 109501. https://doi.org.10.1016/j.jnutbio.2023.109501.
[583] Al-Harbi, N.O.; Imam, F.; Al-Harbi, M.M.; Al-Shabanah, O.A.; Alotaibi, M.R.; As Sobeai, H.M.; Afzal, M.; Kazmi, I.; Al Rikabi, A.C. Rutin inhibits carfilzomib-induced oxidative stress and inflammation via the NOS-mediated NF-κB signaling pathway. *Inflammopharmacology.* **2019**, *27*, 817-827. https://doi.org.10.1007/s10787-018-0550-5.
[584] Kamalakkannan, N.; Stanely Mainzen Prince, P. The influence of rutin on the extracellular matrix in streptozotocin-induced diabetic rat kidney. *J Pharm Pharmacol.* **2006**, *58*, 1091-1098. https://doi.org.10.1211/jpp.58.8.0010.
[585] Korkmaz, A.; Kolankaya, D. Protective effect of rutin on the ischemia/reperfusion induced damage in rat kidney. *J Surg Res.* **2010**, *164*, 309-315. https://doi.org.10.1016/j.jss.2009.03.022.
[586] Alhoshani, A.R.; Hafez, M.M.; Husain, S.; Al-Sheikh, A.M.; Alotaibi, M.R.; Al Rejaie, S.S.; Alshammari, M.A.; Almutairi, M.M.; Al-Shabanah, O.A. Protective effect of rutin supplementation against cisplatin-induced nephrotoxicity in rats. *BMC Nephrol*. **2017**, *18*, 194. https://doi.org.10.1186/s12882-017-0601-y.
[587] Sadeghnia, H.R.; Yousefsani, B.S.; Rashidfar, M.; Boroushaki, M.T.; Asadpour, E.; Ghorbani, A. Protective effect of rutin on hexachlorobutadiene-induced nephrotoxicity. *Ren Fail.* **2013**, *35*, 1151-1155. https://doi.org.10.3109/0886022X.2013.815546.
[588] Han, Y.; Lu, J.S.; Xu, Y.; Zhang, L.; Hong, B.F. Rutin ameliorates renal fibrosis and proteinuria in 5/6-nephrectomized rats by anti-oxidation and inhibiting activation of TGFβ1-smad signaling. *Int J Clin Exp Pathol.* **2015**, *8*, 4725-4734.
[589] Chen, Y.S.; Hu, Q.H.; Zhang, X.; Zhu, Q.; Kong, L.D. Beneficial effect of rutin on oxonate-induced hyperuricemia and renal dysfunction in mice. *Pharmacol.* **2013**, *92*, 75-83. https://doi.org.10.1159/000351703.
[590] Uthra, C.; Reshi, M.S.; Jaswal, A.; Yadav, D.; Shrivastava, S.; Sinha, N.; Shukla, S. Protective efficacy of rutin against acrylamide-induced oxidative stress, biochemical alterations and histopathological lesions in rats. Toxicol Res. **2022**, *11*, 215-225. https://doi.org.10.1093/toxres/tfab125.
[591] Küçükler, S.; Kandemir, F.M.; Özdemir, S.; Çomaklı, S.; Caglayan, C. Protective effects of rutin against deltamethrin-induced hepatotoxicity and nephrotoxicity in rats via regulation of oxidative stress, inflammation, and apoptosis. *Environ Sci Pollut Res Int.* **2021**, 28, 62975-62990. https://doi.org.10.1007/s11356-021-15190-w.
[592] Kandemir, F.M.; Ozkaraca, M.; Yildirim, B.A.; Hanedan, B.; Kirbas, A.; Kilic, K.; Aktas, E.; Benzer, F. Rutin attenuates gentamicin-induced renal damage by reducing oxidative stress, inflammation, apoptosis, and autophagy in rats. *Ren Fail.* **2015**, *37*, 518-525. https://doi.org.10.3109/0886022X.2015.1006100.

[593] Caglayan, C.; Kandemir, F.M.; Yildirim, S.; Kucukler, S.; Eser, G. Rutin protects mercuric chloride-induced nephrotoxicity via targeting of aquaporin 1 level, oxidative stress, apoptosis and inflammation in rats. *J Trace Elem Med Biol.* **2019**, *54*, 69-78. https://doi.org.10.1016/j.jtemb.2019.04.007.
[594] H.H. Hao, Z.M. Shao, Tang, D.Q.; Lu, Q.; Chen, X.; Yin, X.X.; Wu, J.; Chen, H. Preventive effects of rutin on the development of experimental diabetic nephropathy in rats. *Life Sci.* **2012**, *91*, 959-967. https://doi.org.10.1016/j.lfs.2012.09.003.
[595] Khan, R.A.; Khan, M.R.; Sahreen, S. Protective effects of rutin against potassium bromate induced nephrotoxicity in rats. *BMC Complement Altern Med*. **2012**, *12*, 204. https://doi.org.10.1186/1472-6882-12-204.
[596] Arjumand, W.; Seth, A.; Sultana, S. Rutin attenuates cisplatin induced renal inflammation and apoptosis by reducing NFκB, TNF-α and caspase-3 expression in wistar rats. *Food Chem Toxicol.* **2011**, *49*, 2013-2021. https://doi.org.10.1016/j.fct.2011.05.012.
[597] Ghodasara, J.; Pawar, A.; Deshmukh, C.; Kuchekar, B. Inhibitory effect of rutin and curcumin on experimentally-induced calcium oxalate urolithiasis in rats. *Pharmacognosy Res.* **2010**, *2*, 388-392. https://doi.org.10.4103/0974-8490.75462.
[598] Liu, R.; Meng, C.; Zhang, Z.; Ma, H.; Lv, T.; Xie, S.; Liu, Y.; Wang, C. Comparative metabolism of schaftoside in healthy and calcium oxalate kidney stone rats by UHPLC-Q-TOF-MS/MS method. *Anal Biochem.* **2020**, *597*, 113673. https://doi.org.10.1016/j.ab.2020.113673.
[599] Khalaf, M.M.; Hassan, S.M.; Sayed, A.M.; Abo-Youssef, A.M. Ameliorate impacts of scopoletin against vancomycin-induced intoxication in rat model through modulation of Keap1-Nrf2/HO-1 and IκBα-P65 NF-κB/P38 MAPK signaling pathways: Molecular study, molecular docking evidence and network pharmacology analysis. *Int Immunopharmacol.* **2022**, *102*, 108382. https://doi.org.10.1016/j.intimp.2021.108382.
[600] Ding, Z.; Dai, Y.; Wang, Z. Hypouricemic action of scopoletin arising from xanthine oxidase inhibition and uricosuric activity. *Planta Med.* **2005**, *71*, 183-185. https://doi.org.10.1055/s-2005-837789.
[601] Philbrick, D.J.; Bureau, D.P.; Collins, F.W.; Holub, B.J. Evidence that soyasaponin Bb retards disease progression in a murine model of polycystic kidney disease. *Kidney Int*. **2003**, *63*, 1230-1239. https://doi.org.10.1046/j.1523-1755.2003.00869.x.
[602] Kishore, L.; Kaur, N.; Singh, R. Renoprotective effect of Bacopa monnieri via inhibition of advanced glycation end products and oxidative stress in STZ-nicotinamide-induced diabetic nephropathy. *Ren Fail.* **2016**, *38*, 1528-1544. https://doi.org.10.1080/0886022X.2016.1227920.
[603] Bedir, F.; Kocatürk, H.; Yapanoğlu, T.; Gürsul, C.; Arslan, R.; Mammadov, R.; Çoban, A.; Altuner, D.; Suleyman, H. Protective effect of taxifolin against prooxidant and proinflammatory kidney damage associated with acrylamide in rats. *Biomed Pharmacother.* **2021**, *139*, 11660. https://doi.org.10.1016/j.biopha.2021.111660.
[604] Alanezi, A.A.; Almuqati, A.F.; Alfwuaires, M.A.; Alasmari, F.; Namazi, N.I.; Althunibat, O.Y.; Mahmoud, A.M. Taxifolin prevents cisplatin nephrotoxicity by modulating Nrf2/HO-1 pathway and mitigating oxidative stress and inflammation in mice. *Pharmaceuticals.* **2022**, *15*, 1310. https://doi.org.10.3390/ph15111310.
[605] Zhao, Y.; Huang, W.; Wang, J.; Chen, Y.; Huang, W.; Zhu, Y. Taxifolin attenuates diabetic nephropathy in streptozotocin-induced diabetic rats. *Am J Transl Res.* **2018**, *10*, 1205-1210.
[606] Topal, I.; Özdamar, M.Y.; Catakli, T.; Malkoc, İ.; Hacimuftuoglu, A.; Mamoulakis, C.; Tsatsakis, A.; Tsarouhas, K.; Tsitsimpikou, C.; Taghizadehghalehjoughi, A. Renoprotective effect of taxifolin in paracetamol-induced nephrotoxicity: emerging evidence from an animal model. *J Clin Med.* **2023**, *12*, 876. https://doi.org.10.3390/jcm12030876.

[607] Algefare, A.I. Renoprotective and oxidative stress-modulating effects of taxifolin against cadmium-induced nephrotoxicity in mice. *Life.* **2022**, *12*, 1150. https://doi.org.0.3390/life12081150.
[608] Papadimitriou, A.; Silva, K.C.; Peixoto, E.B.; Borges, C.M.; J.M.; Lopes de Faria, J.B. Theobromine increases NAD^+/Sirt-1 activity and protects the kidney under diabetic conditions. *Am J Physiol Renal Physiol.* **2015**, *308*, F209-25. https://doi.org.10.1152/ajprenal.00252.2014.
[609] Mazumder, K.; Biswas, B.; Al Mamun, A.; Billah, H.; Abid, A.; Sarkar, K.K.; Saha, B.; Azom, S.; Kerr, P.G. Investigations of AGEs' inhibitory and nephroprotective potential of ursolic acid towards reduction of diabetic complications. *J Nat Med.* **2022**, *76*, 490-503. https://doi.org.10.1007/s11418-021-01602-1.
[610] Pai, P.G.; Chamari Nawarathna, S.; Kulkarni, A.; Habeeba, U.; Reddy, C.S.; Teerthanath, S.; Shenoy, J.P. Nephroprotective effect of ursolic acid in a murine model of gentamicin-induced renal damage. *ISRN Pharmacol*. **2012**, 410902. DOI: 10.5402/2012/410902.
[611] Ding, Y.J.; Sun, C.Y.; Wen, C.C.; Chen, Y.H. Nephroprotective role of resveratrol and ursolic acid in aristolochic acid intoxicated zebrafish. *Toxins (Basel).* **2015**, *7*, 97-109. https://doi.org.10.3390/toxins7010097.
[612] Ling, C.; Jinping, L.; Xia, L.; Renyong, Y. Ursolic acid provides kidney protection in diabetic rats. *Curr Ther Res Clin Exp.* **2013**, *75*, 59-63. https://doi.org.10.1016/j.curtheres.2013.07.001.
[613] Thakur, R.; Sharma, A.; Lingaraju, M.C.; Begum, J.; Kumar, D.; Mathesh, K.; Kumar, P.; Singh, T.U.; Kumar, D. Ameliorative effect of ursolic acid on renal fibrosis in adenine-induced chronic kidney disease in rats. *Biomed Pharmacother.* **2018**, *101*, 972-980. https://doi.org. 10.1016/j.biopha.2018.02.143.
[614] Peng, J.; Ren, X.; Lan, T.; Chen, Y.; Shao, Z.; Yang, C. Renoprotective effects of ursolic acid on ischemia/reperfusion-induced acute kidney injury through oxidative stress, inflammation and the inhibition of STAT3 and NF-κB activities. *Mol Med Rep.* **2016**, *14*, 3397-3402. DOI: 10.3892/mmr.2016.5654.
[615] Zhang, Z.; Zhang, H.; Chen, R.; Wang, Z. Oral supplementation with ursolic acid ameliorates sepsis-induced acute kidney injury in a mouse model by inhibiting oxidative stress and inflammatory responses. *Mol Med Rep*. **2018**, *17*, 7142-7148. https://doi.org.10.3892/mmr.2018.8767.
[616] Wang, Z.H.; Hsu, C.C.; Huang, C.N.; Yin, M.C. Anti-glycative effects of oleanolic acid and ursolic acid in kidney of diabetic mice. *Eur J Pharmacol.* **2010**, *628*, 255-260. https://doi.org.10.1016/j.ejphar.2009.11.019.
[617] Pei, J.; Wu, M.; Cai, S.; Peng, J.; Zhan, X.; Wang, D.; Wang, W.; An, N. The protective effect of ursolic acid on unilateral ureteral obstruction in rats by activating the Nrf2/HO-1 antioxidant signaling pathway. *Comput Intell Neurosci.* **2022**, *25*, 3690524. https://doi.org.10.1155/2022/3690524.
[618] Ma, J.Q.; Ding, J.; Xiao, Z.H.; Liu, C.M. Ursolic acid ameliorates carbon tetrachloride-induced oxidative DNA damage and inflammation in mouse kidney by inhibiting the STAT3 and NF-κB activities. *Int Immunopharmacol.* **2014**, *21*, 389-395. https://doi.org.10.1016/j.intimp.2014.05.022.
[619] Zhao, J.; Zheng, H.; Sui, Z.; Jing, F.; Quan, X.; Zhao, W.; Liu, G. Ursolic acid exhibits anti-inflammatory effects through blocking TLR4-MyD88 pathway mediated by autophagy. *Cytokine.* **2019**, *123*, 154726. https://doi.org.10.1016/j.cyto.2019.05.013.
[620] Bacanli, M.; Aydin, S.; Anlar, H.G.; Çal, T.; Ündeğer Bucurgat, Ü.; Ari, N.; Başaran, AA.; Başaran, N. Protective effects of ursolic acid in the kidneys of diabetic rats. *Turk J Pharm Sci.* **2018**, *15*, 166-170. https://doi.org.10.4274/tjps.49469.
[621] Li, J.; Li, N.; Yan, S.; Liu, M.; Sun, B.; Lu, Y.; Shao, Y. Ursolic acid alleviates inflammation and against diabetes-induced nephropathy through TLR4-mediated inflammatory pathway. *Mol Med Rep.* **2018**, *18*, 4675-4681. https://doi.org.10.3892/mmr.2018.9429.
[622] Zhou, Y.; Li, J.S.; Zhang, X.; Wu, Y.J.; Huang, K.; Zheng, L. Ursolic acid inhibits early lesions of diabetic nephropathy. *Int J Mol Med.* **2010**, *26*, 565-570. https://doi.org.10.3892/ijmm_00000500.

[623] Xu, H.L.; Wang, X.T.; Cheng, Y.; Zhao, J.G.; Zhou, Y.J.; Yang, J.J.; Qi, M.Y. Ursolic acid improves diabetic nephropathy via suppression of oxidative stress and inflammation in streptozotocin-induced rats. *Biomed Pharmacother.* **2018**, *105*, 915-921. https://doi.org.10.1016/j.biopha.2018.06.055.
[624] Tripathi, P.; Alshahrani, S. Mitigation of ILβ-1, ILβ-6, TNF-α, and markers of apoptosis by ursolic acid against cisplatin-induced oxidative stress and nephrotoxicity in rats. *Hum Exp Toxicol.* **2021**, *40*, S397-S405. https://doi.org.10.1177/09603271211045953.
[625] Alamri, E.S.; El Rabey, H.A. The protective effects of vanillic acid and vanillic acid-coated silver nanoparticles (AgNPs) in streptozotocin-induced diabetic rats. *J Diabetes Res.* **2024**, 4873544. https://doi.org.10.1155/2024/4873544.
[626] Singh, B.; Kumar, A.; Singh, H.; Kaur, S.; Arora, S.; Singh, B. Protective effect of vanillic acid against diabetes and diabetic nephropathy by attenuating oxidative stress and upregulation of NF-κB, TNF-α and COX-2 proteins in rats. *Phytother Res.* **2022**, *36*, 1338-1352. https://doi.org.10.1002/ptr.7392.
[627] Kumari, S.; Kamboj, A.; Wanjari, M.; Sharma, A.K. Nephroprotective effect of vanillic acid in STZ-induced diabetic rats. *J Diabetes Metab Disord.* **2021**, *20*, 571-582. https://doi.org.0.1007/s40200-021-00782-7.
[628] Vinothiya, K.; Ashokkumar, N. Modulatory effect of vanillic acid on antioxidant status in high fat diet-induced changes in diabetic hypertensive rats. *Biomed Pharmacother.* **2017**, *87*, 640-652. https://doi.org.10.1016/j.biopha.2016.12.134.
[629] Sindhu, G.; Nishanthi, E.; Sharmila, R. Nephroprotective effect of vanillic acid against cisplatin induced nephrotoxicity in wistar rats: a biochemical and molecular study. *Environ Toxicol Pharmacol.* **2015**, *39*, 392-404. https://doi.org.10.1016/j.etap.2014.12.008.
[630] Amini, N.; Shoshtari, M.H.; Nejaddehbashi, F.; Dianat, M.; Badavi, M. Dose-dependent renoprotective effect of vanillic acid on methotrexate-induced nephrotoxicity via its anti-apoptosis, antioxidant, and anti-inflammatory properties. *Naunyn Schmiedebergs Arch Pharmacol.* **2024**, *397*, 4195-4204. https://doi.org.10.1007/s00210-023-02866-y.
[631] Song, J.; Wang, H.; Sheng, J.; Zhang, W.; Lei, J.; Gan, W.; Cai, F.; Yang, Y. Vitexin attenuates chronic kidney disease by inhibiting renal tubular epithelial cell ferroptosis via NRF2 activation. *Mol Med.* **2023**, *29*, 147. https://doi.org.10.1186/s10020-023-00735-1.
[632] Umar Ijaz, M.; Batool, M.; Batool, A.; Al-Ghanimd, K.A.; Zafar, S.; Ashraf, A.; Al-Misned, F.; Ahmed, Z.; Shahzadi, S.; Samad, A.; Atique, U.; Al-Mulhm, N.; Mahboob, S. Protective effects of vitexin on cadmium-induced renal toxicity in rats. *Saudi J Biol Sci.* **2021**, *28*, 5860-5864. https://doi.org.10.1016/j.sjbs.2021.06.040.
[633] Miao, N.; Wang, B.; Xu, D.; Wang, Y.; Gan, X.; Zhou, L.; Xue, H.; Zhang, W.; Wang, X.; Lu, L. Caspase-11 promotes cisplatin-induced renal tubular apoptosis through a caspase-3-dependent pathway. *Am J Physiol Renal Physiol.* **2018**, *314*, F269-F279. https://doi.org.10.1152/ajprenal.00091.2017.

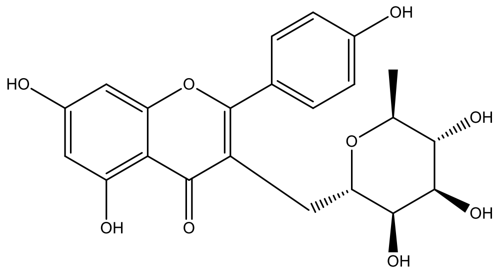

afzelin **(1)**

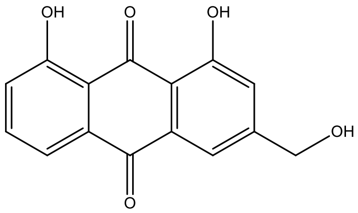

aloe-emodin **(2)**

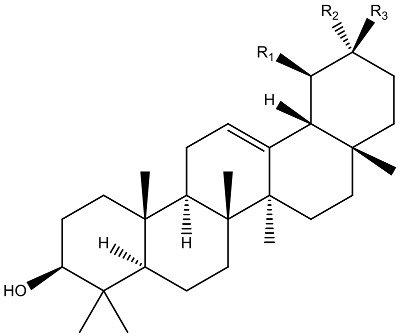

a-amyrin ($R_1 = R_2 = CH_3$ R_3 = H) **(3a)**
b-amyrin (R_1 = H $R_2 = R_3 = CH_3$) **(3b)**

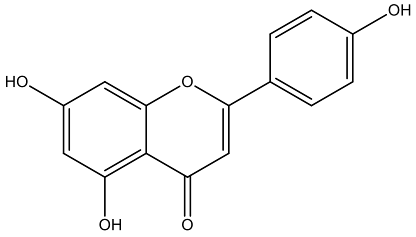

apigenin **(4)**

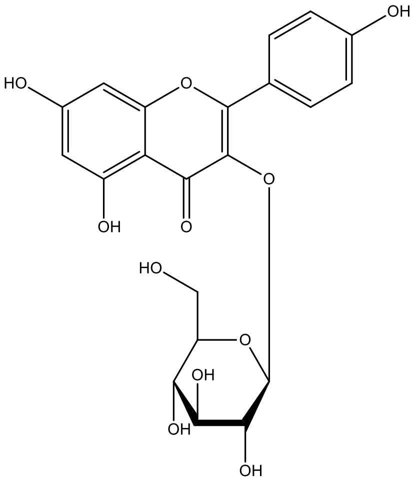

astragalin **(5)**

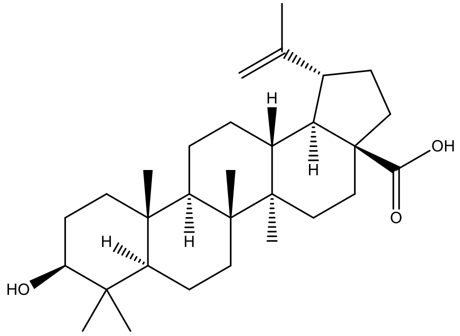

betulinic acid **(6)**

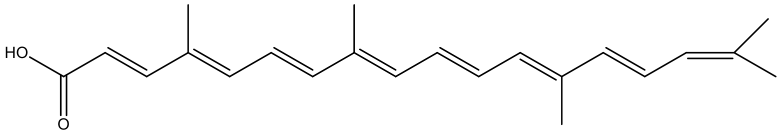

bixin **(7)**

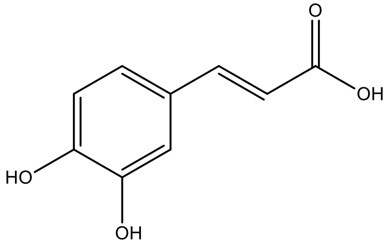

caffeic acid **(8)**

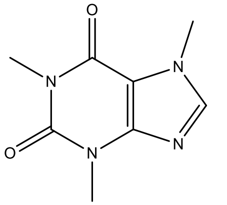

caffeine **(9)**

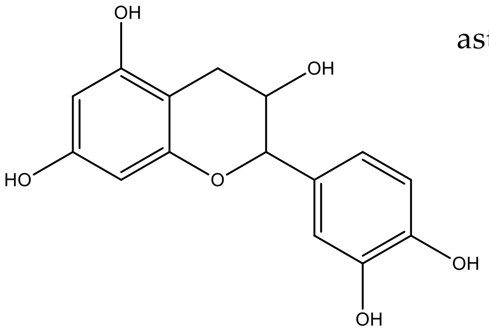

catechin **(10)**

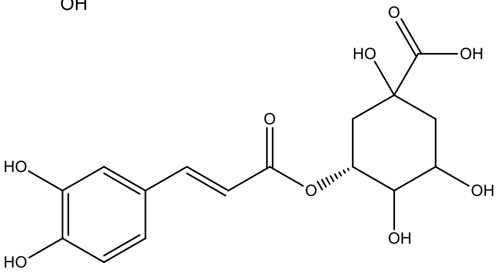

chlorogenic acid **(11)**

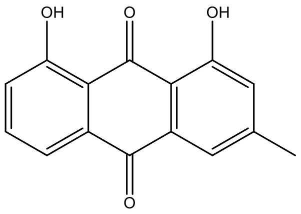

chrysophanol **(12)**

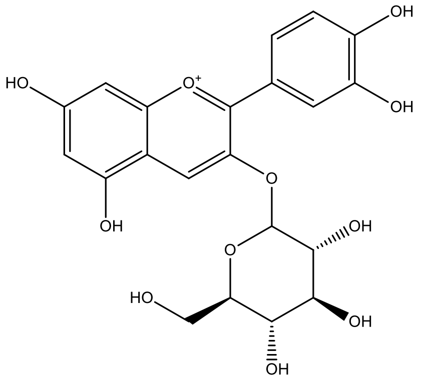

cyanidin-3-*O*-glucoside **(13)**

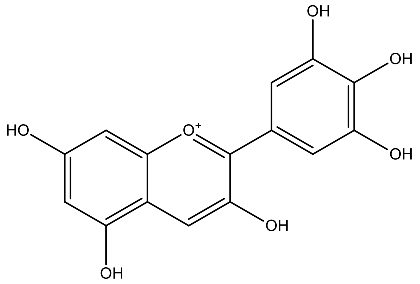

delphinidin **(14)**

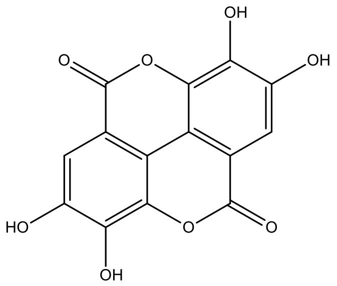

ellagic acid **(15)**

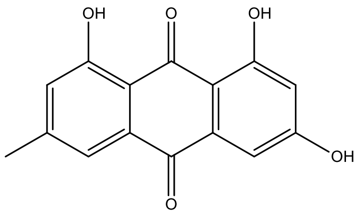

emodin **(16)**

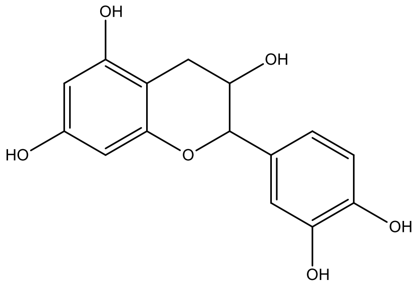

epicatechin **(17)**

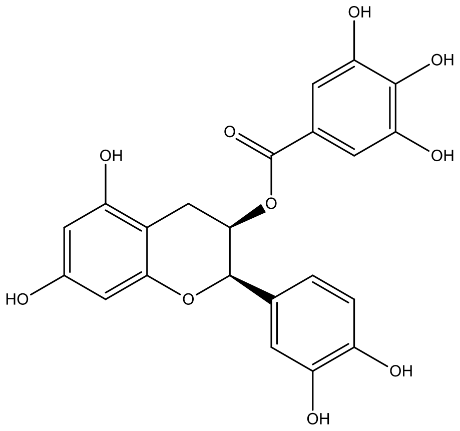

epicatechin gallate **(18)**

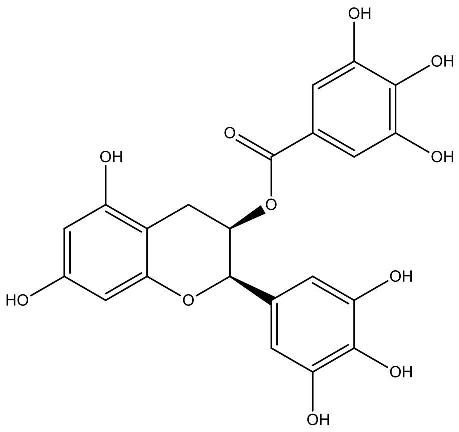

epigallocatechin-3-gallate **(19)**

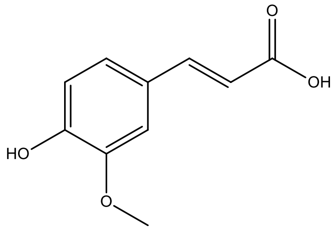

ferulic acid **(20)**

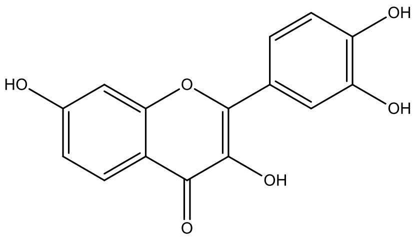

fisetin **(21)**

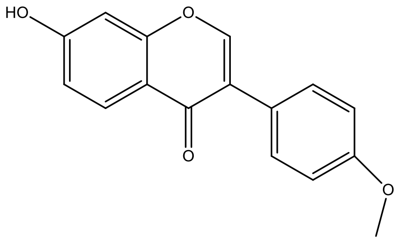

formononetin **(22)**

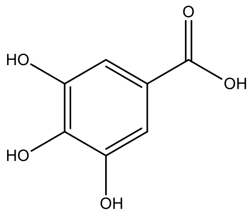

gallic acid **(23)**

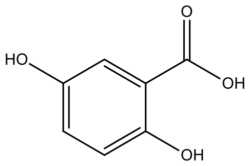

gentisic acid **(24)**

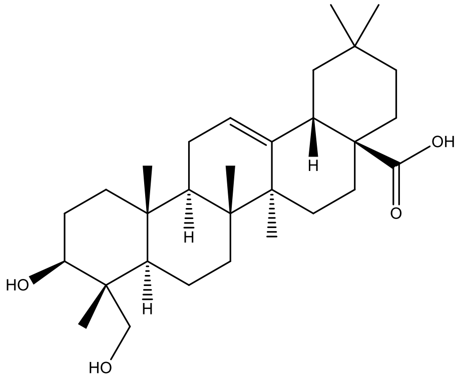

hederagenin **(25)**

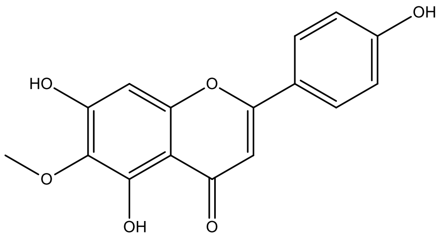

hispidulin **(26)**

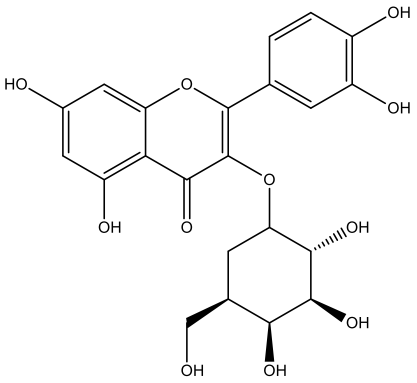

hyperoside **(27)**

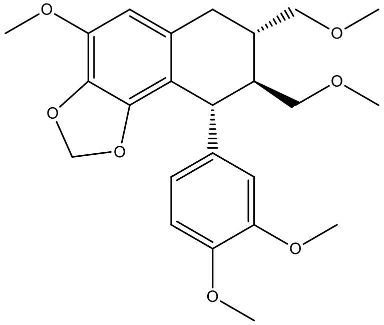

hypophyllanthin **(28)**

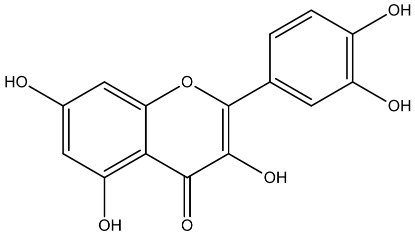

isorhamnetin **(29)**

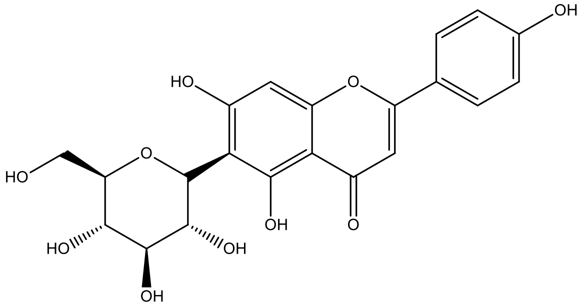

isovitexin **(30)**

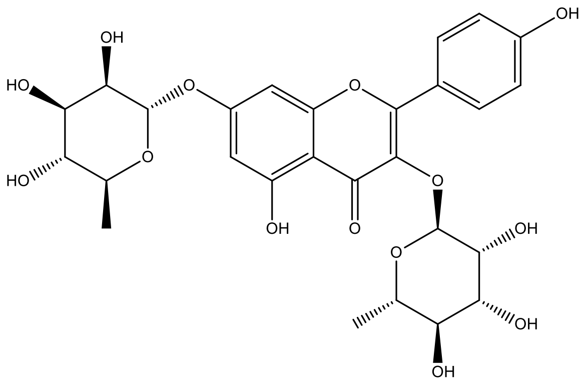

kaempferitrin **(31)**

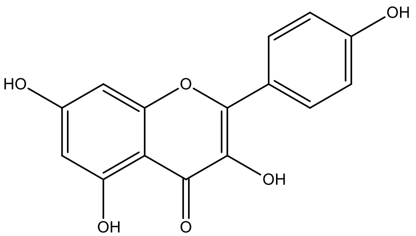

kaempferol **(32)**

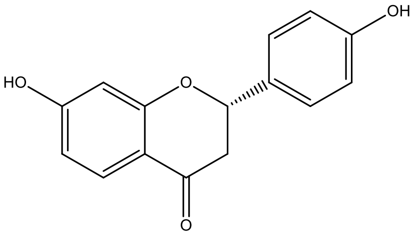

liquiritigenin **(33)**

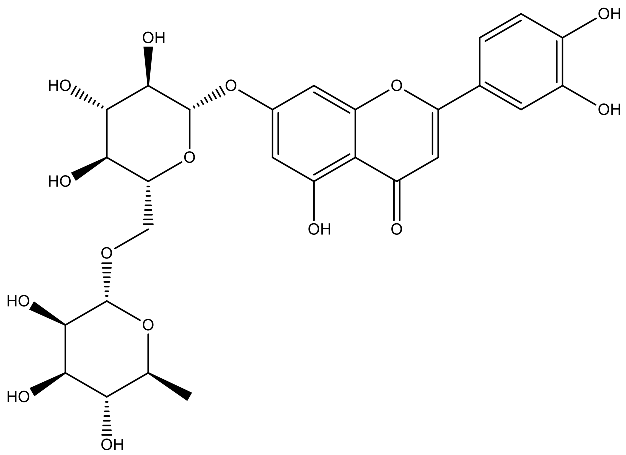

luteolin **(34)**

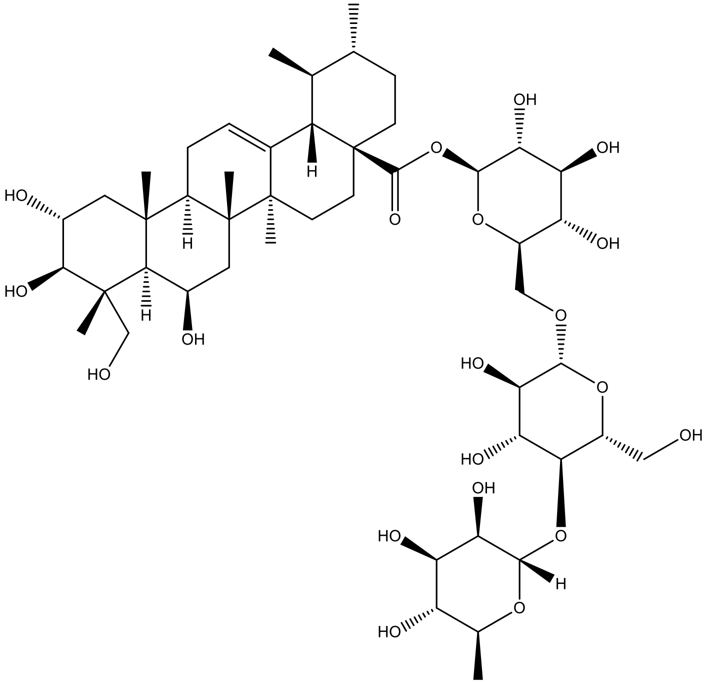

madecassoside **(35)**

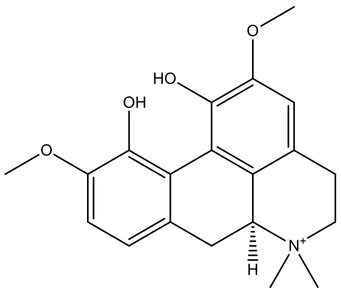

magnoflorine **(36)**

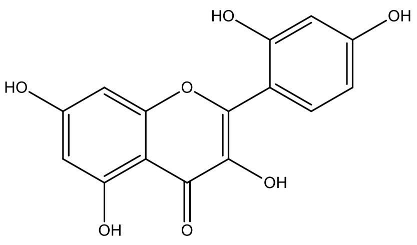

morin **(37)**

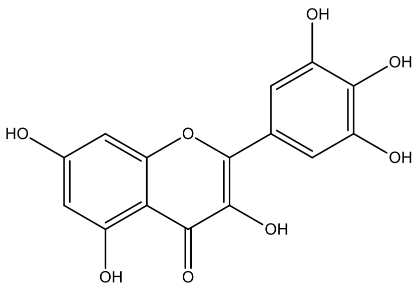

myricetin **(38)**

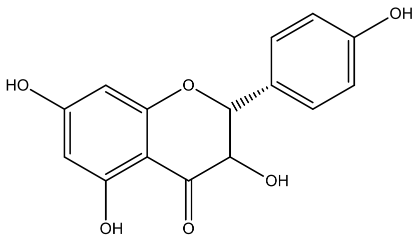

naringenin **(39)**

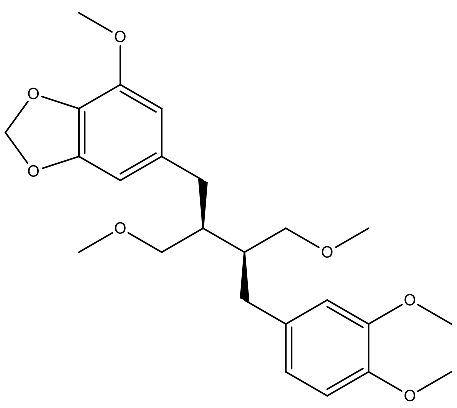

niranthin **(40)**

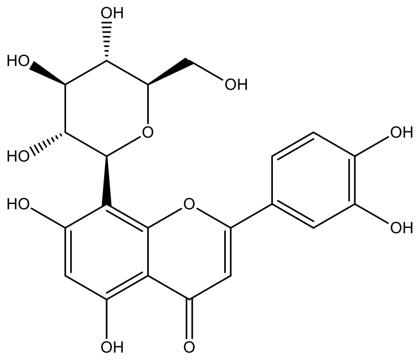

orientin **(41)**

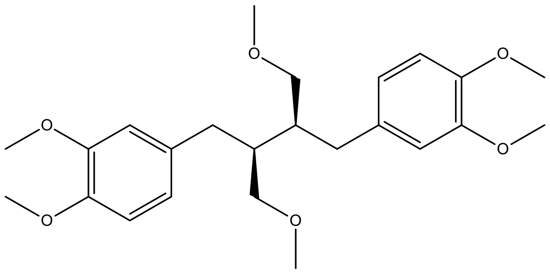

phyllanthin **(42)**

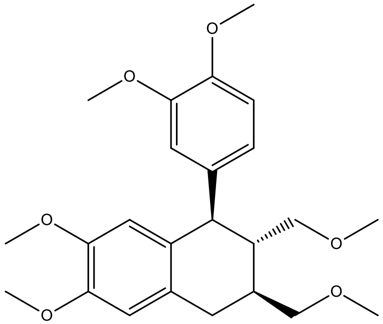

phyltetralin **(43)**

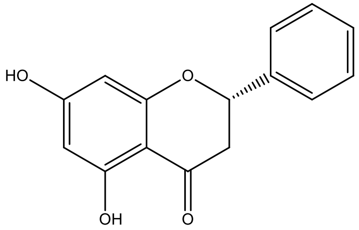

pinocembrin **(44)**

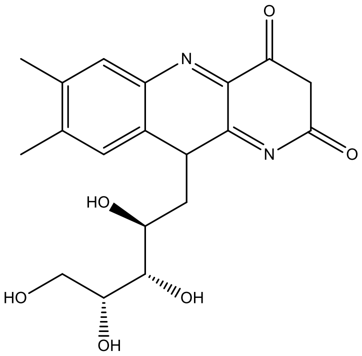

procyanidin B2 **(45)**

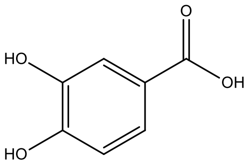

protocatechuic acid **(46)**

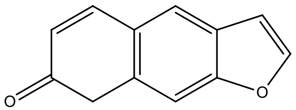

psoralen **(47)**

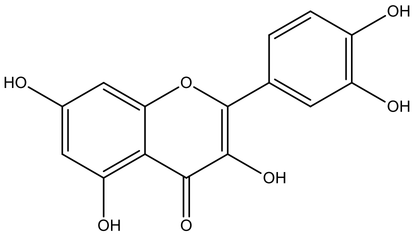

quercetin **(48)**

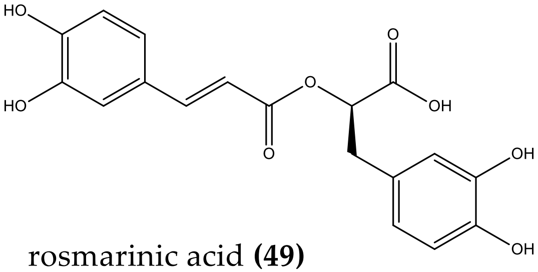

rosmarinic acid **(49)**

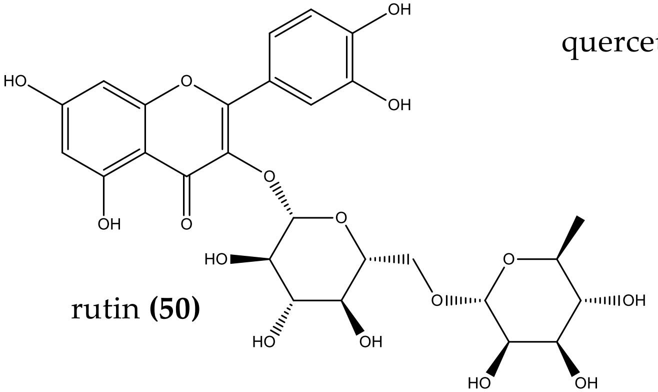

rutin **(50)**

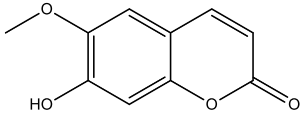

scopoletin **(51)**

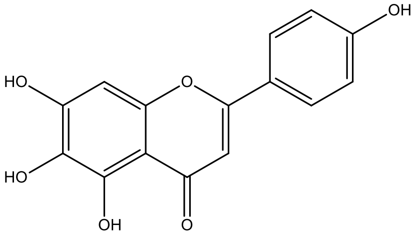

scutellarein **(52)**

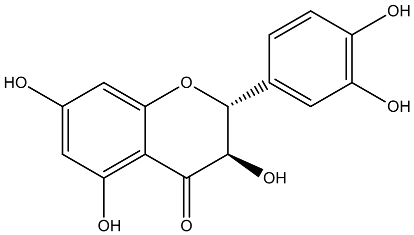

taxifolin **(53)**

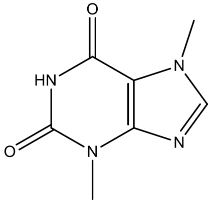

theobromine **(54)**

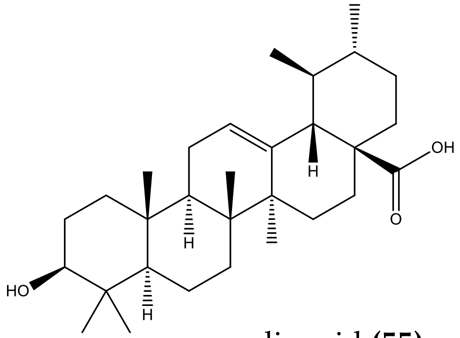

ursolic acid **(55)**

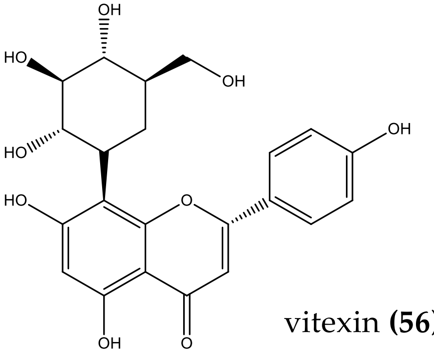

vitexin **(56)**

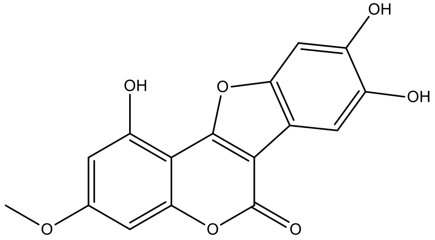

wedelolactone **(57)**

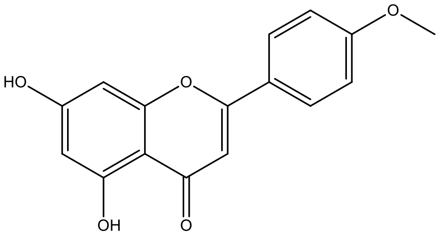

acacetin **(58)**

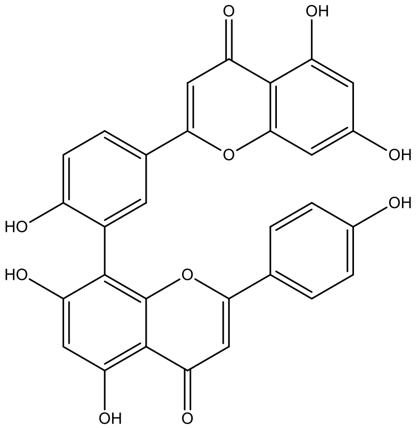

amentoflavone **(59)**

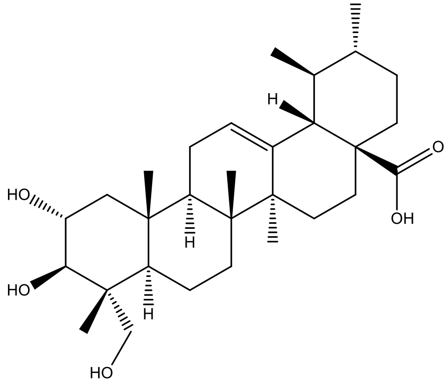

asiatic acid **(60)**

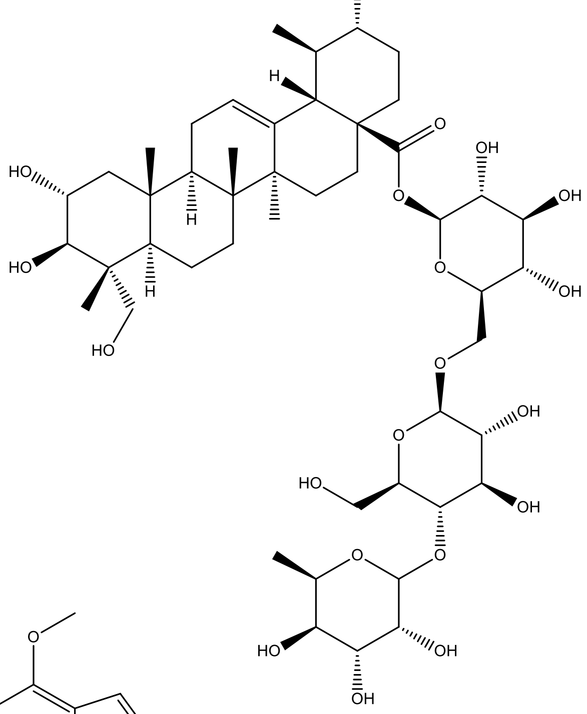

asiaticoside **(61)**

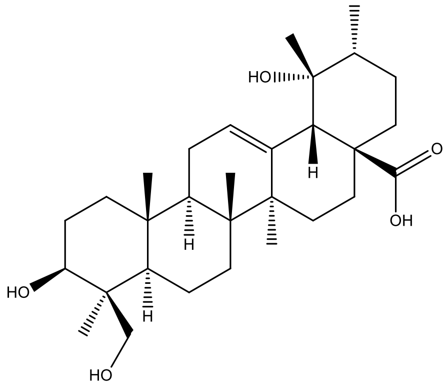

barbinervic acid **(62)**

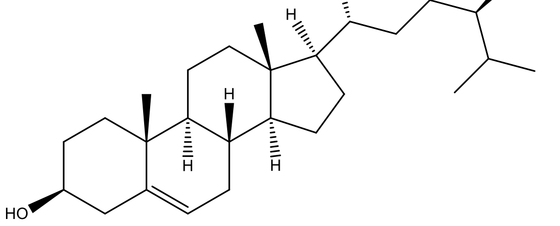

β-sitosterol **(63)**

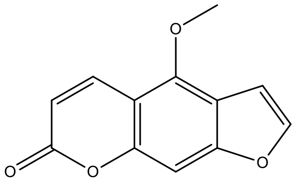

bergapten **(64)**

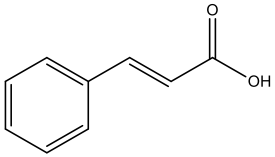

cinnamic acid **(65)**

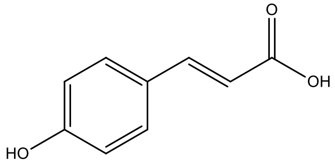

p-coumaric acid **(66)**

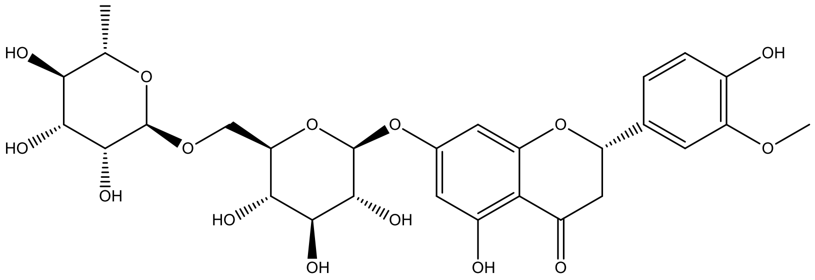

hesperidin **(67)**

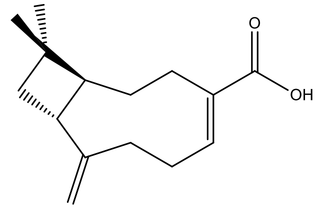

lychnophoric acid **(68)**

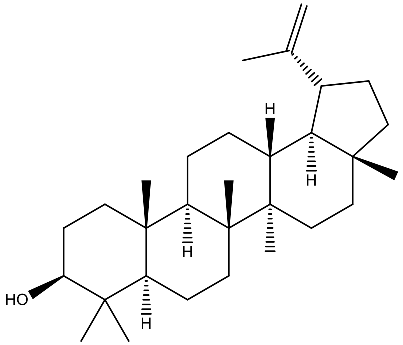

lupeol **(69)**

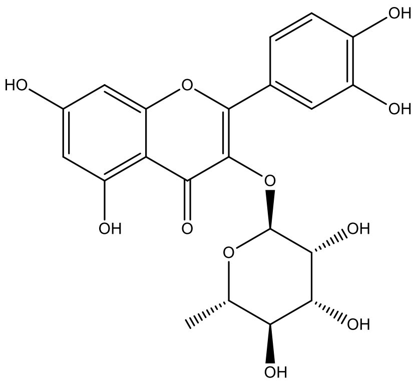

quercitrin **(70)**

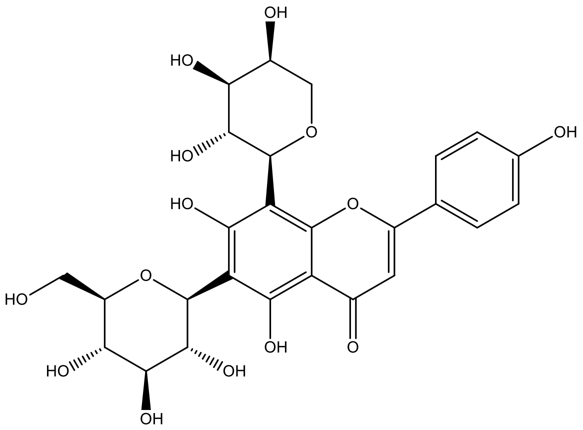

schaftoside **(71)**

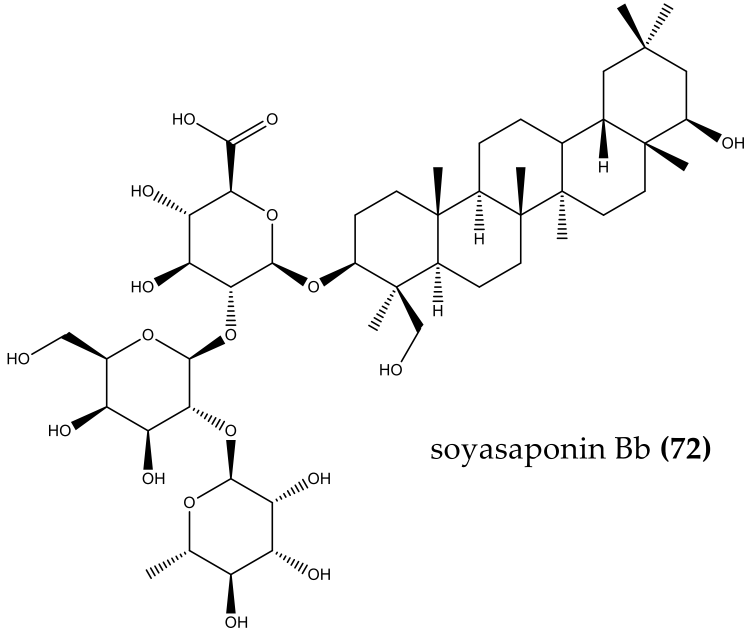

soyasaponin Bb **(72)**

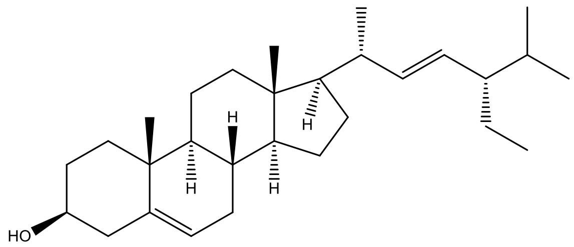

stigmasterol **(73)**

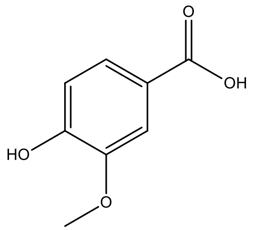

vanillic acid **(74)**

Figure S1. Molecular structures of secondary metabolites with nephroprotective activity evaluated in vitro and in vivo, shown in Tables S1 and S2 of the supporting information.
